# Are We Accurately Predicting Mortality in Renal Cancer? A Systematic Review of Prognostic Models

**DOI:** 10.3390/jcm14165851

**Published:** 2025-08-19

**Authors:** Laura Martinez-Cayuelas, Pau Sarrio-Sanz, Blanca Lumbreras, Vicente F. Gil-Guillen, Jesus Romero-Maroto, Luis Gomez-Perez

**Affiliations:** 1 Urology Services, University Hospital of San Juan de Alicante, Nacional Street n-332. s/n., 03550 Alicante, San Juan de Alicante, Spain; martinezcayuelaslaura@gmail.com (L.M.-C.); blumbreras@umh.es (B.L.); vte.gil@gmail.com (V.F.G.-G.); jromeroma@coma.es (J.R.-M.); l.gomez@umh.es (L.G.-P.); 2 Publich Health, Science History and Gynaecology Department, Miguel Hernández University, 03550 Alicante, San Juan de Alicante, Spain; 3 CIBER of Epidemiology and Public Health (CIBERESP), Instituto de Salud Carlos III, C/Monforte de Lemos 3-5 Pabellon 11 Planta 0, 28029 Madrid, Spain; 4 Clinical Medicine Department, Miguel Hernández University, 03202 Alicante, San Juan de Alicante, Spain; 5 Pathology and Surgery Department, Miguel Hernández University, 03550 Alicante, San Juan de Alicante, Spain; 6 Urology Services, University and General Hospital of Elche, 03203 Alicante, Elche, Spain

**Keywords:** kidney cancer, nomogram, risk score, cancer specific survival, mortality

## Abstract

**Background/Objectives**: Renal cancer has a heterogeneous characteristic. Prognostic models can enable a better evaluation of the prognosis. This study aimed to analyze the clinical applicability and risk of bias of prognostic models described in the literature for predicting cancer-specific mortality in renal cancer patients who have undergone nephrectomy. **Methods**: A systematic review (PROSPERO [CRD42021243529]) of all scientific articles that evaluate prognostic models for cancer-specific mortality due to renal cancer was performed. Descriptive analysis and application of the Checklist for Critical Appraisal and Data Extraction for Systematic Reviews of Prediction Modelling Studies (CHARMS) and the Prediction Model Risk Of Bias Assessment Tool (PROBAST) were used. **Results**: Most of the 40 reviewed studies used retrospective cohort designs, mainly based on hospital records or the SEER database, and focused on patients undergoing nephrectomy. While 50% developed models without validation, the rest included internal or external validation methods, with nomograms being the most common format for presenting results. Cox regression was the main modeling technique, although problems such as poor treatment of missing data, inadequate reporting of events per variable, and limited assessment of model assumptions were prevalent. According to the PROBAST assessment, all studies showed a high risk of bias, particularly in the scope of analysis, and only 40% had good applicability. **Conclusions**: All the studies analyzed were found to have a high risk of bias, and only 40% demonstrated good applicability. Hence, it is necessary to develop cancer-specific prognostic models for renal cancer based on the CHARMS and PROBAST frameworks.

## 1. Introduction

Renal cancer is the third most common urological tumor [1,2]. It is the sixth most common cancer in Europe (fifth in men and eleventh in women), where it accounts for 2.7% of cancer-related deaths (3.1% in men and 2.2% in women) [3], and one of the ten most prevalent cancers in the United States [3,4].

Renal cancer prognosis mainly depends on histology and the stage at which it is detected. Numerous factors have been investigated as possible predictors of the development of the disease. Therefore, they have been incorporated into prognostic models so that patients can be classified by risk in order to provide them with the most appropriate individualized management [1,5].

Prognostic models, which indicate the probability of an event occurring [6,7], are designed to support decision-making in the management of a patient with a specific condition [8]. The literature reveals a growing interest in this topic, with the publication of numerous prognostic models [7]. However, few of them are currently applied in clinical practice. The Checklist for Critical Appraisal and Data Extraction for Systematic Reviews of Prediction Modelling Studies (CHARMS), which provides guidelines for conducting systematic reviews of predictive models [7,9], was published in 2014 to overcome this lack of applicability.

In 2019, the Prediction Model Risk of Bias Assessment Tool (PROBAST) statement was published to evaluate the applicability and risk of bias of predictive models developed and/or externally validated [10,11]. Systematic reviews of predictive models have been conducted for other cancers. In the case of bladder cancer [12], the studies included in the review showed a high risk of bias and failed to demonstrate the applicability of the models involved. They concluded that the design and result reporting need to be improved in prognostic studies and that further external validations are required. Similarly, Lawlor et al. [13] reviewed models for predicting treatment response in patients with metastatic prostate cancer and concluded that most of the models identified required further evaluation and validation before they could be implemented in clinical practice.

In the case of kidney cancer, no synthesis of the evidence has been conducted to date, despite the urgent need to develop prognostic models. In addition, it is necessary to assess the applicability and risk of bias of the currently available models.

Therefore, the aim of this study is to carry out a systematic review of existing prognostic models for kidney cancer-specific mortality and to evaluate both their applicability and risk of bias.

## 2. Materials and Methods

### 2.1. Protocol

The protocol for this systematic review was registered in the PROSPERO database (CRD42021243529).

The study was carried out in accordance with the guidelines of the Preferred Reporting Items for Systematic Reviews and Meta-Analyses (PRISMA) statement [14].

We applied the 11 items on the CHARMS [7] in order to collect the most relevant information and to identify potential methodological errors. We assessed the risk of bias and the applicability of the models using the PROBAST tool [11].

### 2.2. Study Design, Search Criteria, and Inclusion and Exclusion Criteria

A systematic review was conducted of all articles that developed a predictive model for kidney tumor-specific mortality in patients undergoing total or partial nephrectomy. Articles that reported external validation of the predictive model were included, but not those that conducted only external validation without prior development of the prognostic model. Articles in English and Spanish were included.

Given that the aim was to select models with application to routine clinical practice, studies that incorporated genetic markers in the model or those involving patients receiving only systemic treatment were excluded. Cancer mortality models for all outcomes (short-, medium-, and long-term) were considered.

The literature search was conducted in the Medline (via PubMed), Scopus, and Embase databases, including all articles published from their inception until 4 May 2025. A search was also conducted on the medRxiv preprint server. The full search algorithms are included in Supplementary Material S1.

Additionally, a manual search of the reference lists of the selected articles was performed, complemented by a targeted search on Google Scholar to identify gray literature, including non-peer-reviewed sources that fulfilled the inclusion criteria established for the review. Once the articles were selected, the two primary investigators independently reviewed titles and abstracts to identify those that met the inclusion criteria. In cases of uncertainty, a third reviewer was consulted to resolve discrepancies. The same independent review procedure was applied to the full texts of the articles selected based on their abstracts.

### 2.3. Data Extraction

For each of the selected articles, two investigators extracted the data recommended by the CHARMS [7].

### 2.4. Risk of Bias and Applicability

The risk of bias and applicability of the study were assessed using the PROBAST tool [11]. The potential biases were analyzed in the following domains: participants, predictors, outcome, and analysis. Applicability was assessed in participants, predictors, and outcomes. For each domain, the interpretation and scoring criteria were applied in accordance with the guidance provided by Moons [15], which details how to assess each item and assign scores based on the reported results. A study was considered to have a high risk of bias or low applicability if at least one domain was rated as high risk or low applicability. To decide an overall judgment of risk of bias and applicability, the ‘worst score counts’ approach was employed, whereby the global rating for each article corresponded to the lowest score assigned across the evaluated domains.

### 2.5. Statistical Analysis

A descriptive analysis of CHARMS and PROBAST was conducted [7,11], including the frequencies and percentages of the main characteristics of the models and the results of the domains.

## 3. Results

### 3.1. Results of the Literature Search

**Figure 1:**
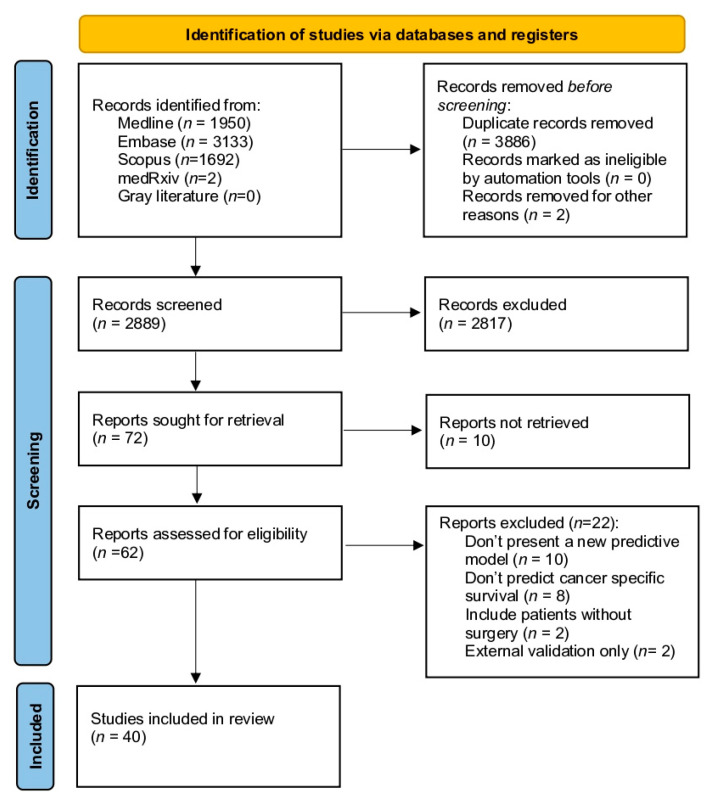
PRISMA flowchart for the systematic review of predictive models in cancer-specific mortality for renal cancer in patients treated with total or partial nephrectomy.

Figure 1 shows the flow diagram of the systematic review. A total of 6777 articles were evaluated (3133 from Embase, 1950 from PubMed, 1692 from Scopus, and 2 from medRxiv), and 2889 articles were selected for full-text review. Finally, 40 studies were included for subsequent analysis [2,5,8,16,17,18,19,20,21,22,23,24,25,26,27,28,29,30,31,32,33,34,35,36,37,38,39,40,41,42,43,44,45,46,47,48,49,50,51].

### 3.2. Characteristics of the Studies According to CHARMS

The CHARMS tables are included in Supplementary Material S2.

### 3.3. Source of Data and Participants

Almost all the studies applied a retrospective cohort design, except two (5%) that applied a prospective design [40,43].

Data sources were obtained from hospital records in 60% of the studies [5,8,19,21,25,26,28,31,32,34,35,36,38,39,40,41,42,43,44,45,46,48,51], and 40% utilized data from the SEER (Surveillance, Epidemiology, and End Results) database of the United States National Cancer Institute [2,16,17,18,20,22,23,24,27,29,30,33,37,49,50,52].

Notably, 50% of the articles only developed the model without conducting validation. Of these, 17 studies presented a single predictive model [8,21,25,26,33,34,36,37,41,42,44,45,46,47,48,49,51], two studies presented separate models for preoperative and postoperative prediction [31,35], and one study created three models for different histopathological subtypes (papillary, chromophobe, and clear cell) [32]. The remaining articles presented both model development and validation [5,16,17,18,19,20,22,23,24,27,29,30,38,39,40,43,50,52], except for studies by Zhou et al. and Guo et al., which included two distinct validation cohorts—one assessing one-year follow-up and the other three-year follow-up [2,28].

All the studies included patients who had undergone nephrectomy, 22.5% of them included patients with metastatic disease, and 77.5% involved patients without metastasis [2,17,19,20,25,31,35,42,44]. Among the non-metastatic patients who underwent surgery, six groups were identified: those including all histological subtypes (the largest group, 48.4%) [8,21,23,28,30,34,37,40,41,43,44,46,47,50,52], those restricted to the papillary subtype (12.9%) [16,27,38,49], the chromophobe subtype (9.6%) [18,22,29], the clear cell subtype (19.3%) [5,18,33,39,45,48], the ductal carcinoma (6.4%) [36,51] and patients with grade 4 cancer only (3.2%) [26].

Treatment was included as a candidate predictor in 47.5% of the studies [2,16,17,18,20,22,23,24,26,27,28,29,30,31,33,35,36,50,52] but not in the remaining 52.5% [5,8,19,21,25,32,34,37,38,39,40,41,42,43,44,45,46,48,49,51].

### 3.4. Outcome to Be Predicted

The primary outcome in all studies was cancer-specific mortality, with prediction times ranging from 6 months to 15 years. The most common prediction periods were one year [5,16,17,20,22,23,25,26,28,30,31,40,41,42,43,44,45,51], three years [2,5,8,16,17,18,20,21,22,23,24,25,26,27,28,29,30,31,33,34,41,42,44,45,47,49,50,51,52], and five years [2,5,8,16,17,18,21,22,23,24,26,27,28,29,30,31,32,33,36,37,38,39,40,41,42,43,44,45,46,47,48,49,50,51,52].

Blinding procedures were not reported in any of the included studies. Mortality data were primarily obtained from death certificates. In 17.5% of the studies, the data collection methodology was explicitly described [31,34,36,38,40,43,47], while in the remaining 82.5%, data were obtained from medical records.

### 3.5. Candidate Predictors

The candidate predictors varied across the articles, including sociodemographic data (age, sex, and race), patient-related factors (systemic symptoms), and tumor-related factors (size, TNM classification, lymphovascular invasion, necrosis, and grade, among the most common).

Continuous variables were categorized in 77.5% of the studies [2,5,8,21,25,26,27,28,29,30,31,32,33,34,36,37,39,42,44,45,46,48,49,50,52], modeled as linear predictors in 20% of the studies [35,36,38,40,43,51], and unspecified in one study [41].

Information on blinding was available in only two studies: Lyon et al. [31] and Frank et al. [45] reported that the histopathological analysis was performed under blinded conditions.

### 3.6. Sample Size

The sample size across studies ranged from 133 to 42,890 patients. The number of events was not reported in 50% of the studies [2,5,8,16,17,19,20,21,22,23,24,26,27,28,29,30,33,34,38,50]. Among the studies that did report this information, the number of events ranged from 22 to 1373. The number of events per variable (EPV) ranged from 1.15 to 98 and was unknown in 50% of articles [2,5,8,16,17,19,20,21,22,23,24,26,27,28,29,30,33,34,38,50]. Among those that did report EPV, 75% fell below the threshold of 20 [18,31,32,35,36,39,41,42,44,46,47,48,49,51,52]; only 25% were above 20 [25,32,37,40,43,45].

### 3.7. Missing Data

The procedure for handling missing data was not reported in 27.5% of studies [19,31,32,35,36,41,44,46,47,48]. The majority, 70%, performed a complete case analysis [2,5,8,16,17,18,20,21,22,23,24,25,26,27,28,29,30,33,37,38,39,40,43,45,49,50,51,52], and only one article imputed missing values [34].

### 3.8. Model Development

The most commonly used statistical model was Cox regression, applied in 85% of the studies [2,5,8,16,17,19,20,21,23,24,26,27,28,29,30,31,32,33,34,36,38,39,40,41,42,43,44,45,46,47,48,50,51,52]. Among the remaining studies, four (10%) applied a competing risk model [22,25,37,49], one study (Zheng et al.) used a shrinkage model [18], and another (Margulis et al.) used logistic regression [35].

The proportional hazard assumption was only tested in three studies [36,40,43].

In most cases (82.5%), the selection of predictors for multivariate analysis was based on previous univariate analysis [2,5,8,16,17,20,21,22,23,24,25,26,27,28,29,30,31,32,33,34,39,40,42,43,44,45,46,47,48,49,50,51,52]; least absolute shrinkage was used in two studies (5%) [18,19]; full model approach was also applied in two studies (5%) [36,38]; and stepwise variable selection was adopted in one study (Margulis et al.) [35].

Multivariate analysis most commonly relied on the full model approach [2,5,8,16,17,18,19,20,21,22,23,24,25,26,27,28,29,30,32,33,34,35,37,39,40,42,43,46,47,48,50,51,52]. Seven studies used the backward stepwise methodology [31,32,36,38,44,45,49]. In their 2018 study, Leibovich et al. [32] developed three models: backward selection was used for the clear cell model, the full model was used for the papillary subtype, and no explicit selection method was reported for the chromophobe model. Hsiao et al. used both the full model and the stepdown method [34]. Only Zheng et al. [18] and Laukhtina et al. [19] performed the shrinkage method.

### 3.9. Model Performance

Discrimination was mostly performed using the c-index [2,5,8,16,17,18,19,20,21,22,23,24,25,26,27,28,29,30,31,32,33,34,36,38,39,40,41,43,45,48,50,51,52] and area under the curve [2,5,8,16,17,18,19,20,21,22,23,24,27,28,29,33,49,50,52]. Regarding calibration, the most commonly used techniques were curves (42.5%) [5,8,16,18,21,22,23,24,25,27,28,29,33,35,48,50,52] followed by plots (32.4%) [2,17,19,26,30,31,34,38,40,41,43,49,51]. Kutikov et al. used quintile calibration [37].

Classification measures were not reported in most studies (57.5%). Among those that did include such metrics, decision curve analysis was the most commonly used (42.5%) [2,5,8,16,17,19,20,21,22,23,24,29,30,31,35,50,52]. Huang et al. [17] used net reclassification improvement and integrated discrimination improvement, in addition to decision curve analysis. In their 2020 study, Zhou et al. [28] used sensitivity and specificity.

### 3.10. Model Evaluation

Bootstrapping was the most commonly used internal validation method, applied in 60% of the studies [8,16,19,20,21,23,24,25,26,28,30,31,32,33,34,36,38,40,41,43,45,49,51,52]. Sixteen studies did not use this method, but 56.25% of these included a validation cohort [2,5,17,18,22,27,29,39,50].

### 3.11. Results

Most of the studies presented a nomogram (62.5%) [5,8,16,18,19,20,21,22,26,27,29,30,33,34,35,37,38,40,41,43,48,49,50,51,52], while nine (22.5%) used a risk score [31,32,36,39,42,44,45,46,47]. Six articles (15%) presented both a nomogram and a risk score [2,17,23,24,25,28]. No studies presented baseline survival.

All the articles that included a development group and one or more validation groups made comparisons between them.

### 3.12. Interpretation and Discussion

Most of the studies presented exploratory results (87.5%) [2,5,8,16,17,18,19,21,22,23,24,25,26,27,28,31,32,33,34,35,36,37,38,39,41,42,44,45,46,47,48,49,50,51,52]; only five presented confirmatory results [20,29,30,40,43]. All studies compared their models with others. Likewise, all discussed strengths and limitations, with the exception of Leibovich et al. in their 2003 study [44].

Virtually all articles highlighted the need for additional external validations, with the exceptions of Lu et al. [20], Chen et al. [29], Zhou et al. [30], and Leibovich et al. [44].

### 3.13. PROBAST Analysis

The results of the PROBAST analysis are included in Supplementary Material S3.

The risk of bias and concerns regarding the applicability of the assessed studies using PROBAST are summarized in Table 1.

In the participant domain, 60% of the studies were rated as having a high risk of bias or nonapplicability. In the analysis domain, all studies were assessed to have a high risk of bias [2,5,8,16,17,18,19,20,21,22,23,24,25,26,27,28,29,30,31,32,33,34,35,36,37,38,39,40,41,42,43,44,45,46,47,48,49,50,51]. Common reasons for high risk of bias in this domain were small sample sizes, categorization of continuous variables, inadequate handling of missing data, and the calibration of the models.

As a result, in the overall section of bias assessment, all studies [2,5,8,16,17,18,19,20,21,22,23,24,25,26,27,28,29,30,31,32,33,34,35,36,37,38,39,40,41,42,43,44,45,46,47,48,49,50,51] were classified as high risk of bias. In terms of applicability, only 40% of the studies demonstrated good applicability [16,18,22,23,24,25,28,34,35,41,45,47,48,50,52].

## 4. Discussion

This study provides a summary of all currently available prognostic models for cancer-specific mortality in renal cancer. It presents highly relevant information for application in clinical practice and for the development of clinical management guidelines. After applying PROBAST criteria, we observed that 100% of the studies had a high risk of bias, and only 40% presented adequate applicability in clinical practice [16,18,22,23,24,25,28,34,35,41,45,47,48,50,52].

To our knowledge, this is the first systematic review focused on the prognosis of renal cancer. Thus, we cannot compare our results with previous reviews. However, similar reviews have been conducted in other fields, such as that by Sarrio et al. [12] in bladder cancer; Beneyto et al. [6] in sepsis, Lawlor et al. in metastatic prostate cancer [13]; and Palazon et al. [10] and Russo et al. [53] in oropharyngeal cancer, among others. The methodology used in those reviews was like ours, as were the results. Three systematic reviews revealed a high risk of bias of 100% in the included studies [6,10,12], whereas this was 92.8% in Lawlor et al. [13] but only 33.3% in Russo et al. [53]. The applicability was more variable, with higher values in the case of Palazón et al. [10], Sarrio et al. [12], and Lawlor et al. [13], with rates of 83.3%, 64.2%, and 71.4%, respectively, whereas this was 42.8% in Beneyto et al. [6] (a value very similar to our value of 40%) and much lower in the case of Russo et al. [53], with only 16.6%.

External validation was performed in 50% of the studies [2,5,16,17,18,19,20,22,23,24,27,28,29,30,38,39,40,43,50,52], slightly less than in the reviews by Beneyto et al. [6] (64.28%), Palazon et al. [10] (66.6%), and Russo et al. [53] (66.6%), and slightly higher than in Sarrio et al. [12] (26%). This external validation is very important for assessing clinical applicability in specific healthcare areas.

Only two of the studies included in our review (5%) applied a prospective design [40,43], and none of the studies analyzed in the review by Russo et al. [53] adopted a prospective approach [53]. Although other systematic reviews have reported higher proportions of prospective studies, none have exceeded 50%[6,10,12]. This highlights the ongoing reliance on retrospective data and underscores the importance of promoting the design and implementation of high-quality prospective research in this field.

The EPV was above 20 in only 12.5% of the included studies, a proportion comparable to those reported by Palazon et al. [10] and Sarrio et al. [12] (16.6% and 21.4%, respectively). None of the studies in our review applied the shrinkage method to adjust regression coefficients (nor did those included in other systematic reviews, such as Russo et al.)[53].

The handling of missing data was inadequate in most reviews, including ours, either due to the performance of complete case analyses or because, in most cases, the issue was not mentioned. Only 2.5% of the studies applied appropriate imputation methods consistent with PROBAST recommendations, a finding in line with previously reported rates ranging from 0% to 16% [34].

Regarding the presentation of prediction tools, 62.5% of the studies included a nomogram, 22.5% reported a risk score, and 15% provided both. These approaches are consistent with those commonly used in the literature, along with mathematical formulas and point-based scoring systems (see the reviews by Beneyto et al. [6] and Palazon et al. [10]).

Overall, our results align with those of previous reviews of prognostic models that show a lack of compliance with CHARMS and PROBAST recommendations. This highlights the need to develop new prognostic models that are both methodologically rigorous and externally validated, thereby enhancing their clinical utility. Given the consistently high risk of bias observed across all models included in this review, none can be recommended for clinical application at present.

The development of prognostic models that conform to CHARMS and PROBAST guidelines—and undergo external validation—would provide clinicians with reliable tools to estimate cancer-specific mortality in patients with renal cancer after nephrectomy, allowing more informed decisions to be made during follow-up.

The main strength of this systematic review lies in its methodology. All the studies in the selection process were independently reviewed by three researchers, who also independently collected the different information from each individual study. Furthermore, we evaluated the probability of bias and applicability using two published and validated tools: CHARMS and PROBAST [7,11].

This review is not without limitations. We may have missed some relevant studies from other databases, which could be considered in future studies by adapting the search algorithm to cover all relevant sources. Language barriers could also be a limitation, as articles in languages unfamiliar to the primary researchers but relevant to the review may not have been considered. However, given that most scientific publications are currently in English, this limitation is probably small.

## 5. Conclusions

This systematic review analyzed a total of 40 prognostic models for renal cancer-specific mortality based on the CHARMS and PROBAST criteria. All the studies analyzed were found to be at high risk of bias, and only 40% demonstrated good applicability. Consequently, none were considered suitable for routine clinical practice.

The least frequently met criteria were participants and analysis. Further development of renal cancer-specific prognostic models based on the CHARMS and PROBAST frameworks is needed. In addition, incorporating prospective models and performing external validations would improve their suitability for clinical application.

## Figures and Tables

**Table 1:** Risk of bias and concern regarding the applicability of the included studies (PROBAST). Abbreviations: PROBAST, Prediction Model Risk of Bias Assessment Tool; ROB, risk of bias. +: low ROB/low concern regarding applicability; -, high ROB/high concern regarding applicability.

Study	ROB				Applicability			Overall			ROB				Applicability			Overall	
	Participants	Predictors	Outcome	Analysis	Participants	Predictors	Outcome	ROB	Applicability	Study	Participants	Predictors	Outcome	Analysis	Participants	Predictors	Outcome	ROB	Applicability
**Chen et al., 2025**	+	+	+	-	+	+	+	-	+	**Zhou et al., 2018**	-	+	+	-	-	+	+	-	-
**Ni et al., 2024**	-	+	+	-	-	+	+	-	-	**Leibovich et al., 2018**	-	+	+	-	-	+	+	-	-
**Guo et al., 2024**	-	+	+	-	-	+	+	-	-	**Zhang et al., 2018**	-	+	+	-	-	+	+	-	-
**Zhanghuang et al., 2022**	+	+	+	-	+	+	+	-	+	**Hsiao et al., 2015**	+	+	+	-	+	+	+	-	+
**Tang et al., 2022**	+	+	+	-	+	+	+	-	+	**May et al., 2013**	-	+	+	-	-	+	+	-	-
**Wang et al., 2022**	+	+	+	-	+	+	+	-	+	**Kutikov et al. 2012**	-	+	+	-	-	+	+	-	-
**Ni et al., 2022**	-	+	+	-	-	+	+	-	-	**Klatte et al., 2010**	-	+	+	-	-	+	+	-	-
**Lu et al., 2022**	-	+	+	-	-	+	+	-	-	**Iimura et al., 2008**	-	+	+	-	-	+	+	-	-
**Laukhtina et al., 2021**	-	+	+	-	-	+	+	-	-	**Karakiewicz et al., 2008**	-	+	+	-	-	+	+	-	-
**Zheng et al., 2022**	+	+	+	-	+	+	+	-	+	**Kanao et al., 2008**	+	+	+	-	+	+	+	-	+
**Huang et al., 2022**	+	+	+	-	-	+	+	-	-	**Karakiewicz et al., 2007**	-	+	+	-	-	+	+	-	-
**Zhanghuang et al., 2022**	+	+	+	-	+	+	+	-	+	**Frank et al., 2002**	+	+	+	-	+	+	+	-	+
**Guo et al., 2021**	+	+	+	-	+	+	+	-	+	**Velis et al., 2017**	-	+	+	-	-	+	+	-	-
**Tian et al., 2021**	+	-	+	-	+	+	+	-	+	**Peng et al., 2016**	+	+	+	-	+	+	+	-	+
**Xiao et al., 2021**	-	-	+	-	-	+	+	-	-	**Peng et al., 2018**	+	+	+	-	+	+	+	-	+
**Su et al., 2021**	-	+	+	-	-	+	+	-	-	**Wu et al., 2020**	-	+	+	-	+	+	+	-	+
**Zhu et al., 2020**	-	+	+	-	-	+	+	-	-	**Lyon et al., 2020**	-	+	+	-	-	+	+	-	-
**Yan et al., 2020**	-	+	+	-	-	+	+	-	-	**Margulis et al., 2012**	?	+	+	-	+	+	+	-	+
**Zhou et al., 2020**	+	+	+	-	+	+	+	-	+	**Cho et al., 2008**	-	+	+	-	-	+	+	-	-
**Chen et al., 2020**	-	+	+	-	-	+	+	-	-	**Leibovich et al., 2003**	-	+	+	-	-	+	+	-	-

## Supplementary Materials

The following supporting information can be downloaded at: https://www.mdpi.com/article/10.3390/jcm14165851/s1.

## Abbreviations

The following abbreviations are used in this manuscript:

PROBAST	Prediction Model Risk of Bias Assessment Tool
PRISMA	Preferred Reporting Items for Systematic Reviews and Meta-Analyses
CHARMS	Checklist for Critical Appraisal and Data Extraction for Systematic Reviews of Prediction Modelling Studies

## References

[1] Lorente D., Trilla E., Meseguer A., Planas J., Placer J., Celma A., Salvador C., Regis L., Morote J. (2017). Systematic review of renal carcinoma prognostic factors. Actas Urol. Esp..

[2] Guo Q., Li S., Zhu J., Wang Z., Li Z., Wang J., Wen R., Li H. (2024). Development and validation of prognostic nomograms for adult with papillary renal cell carcinoma: A retrospective study. Clinics.

[3] Dyba T., Randi G., Bray F., Martos C., Giusti F., Nicholson N., Gavin A., Flego M., Neamtiu L., Dimitrova N. (2021). The European cancer burden in 2020: Incidence and mortality estimates for 40 countries and 25 major cancers. Eur. J. Cancer.

[4] Chowdhury N., Drake C.G. (2020). Kidney Cancer: An Overview of Current Therapeutic Approaches. Urol. Clin. North Am..

[5] Chen W., Tanaka H., Kobayashi M., Fukuda S., Nakayama A., Meagher M.F., Greenwald R., Schmeusser B., Nicase E., Waseda Y. (2025). Development and validation of nomograms and integrated software incorporating preoperative C-reactive protein level for prognostic prediction of nonmetastatic clear cell renal cell carcinoma: Results from the International Marker Consortium for Renal Cancer (INMARC) Registry. World J. Urol..

[6] Beneyto-Ripoll C., Palazón-Bru A., Llópez-Espinós P., Martínez-Díaz A.M., Gil-Guillén V.F., de Los Ángeles Carbonell-Torregrosa M. (2021). A critical appraisal of the prognostic predictive models for patients with sepsis: Which model can be applied in clinical practice?. Int. J. Clin. Pract..

[7] Moons K.G.M., de Groot J.A.H., Bouwmeester W., Vergouwe Y., Mallett S., Altman D.G., Reitsma J.B., Collins G.S. (2014). Critical appraisal and data extraction for systematic reviews of prediction modelling studies: The CHARMS checklist. PLoS Med..

[8] Ni J., Yao X., Song W., Zhang H., Zhang H., Wang Y., Zhang Y., Wang G., Wang K., Mao W. (2024). Prognostic value of preoperative combined neutrophil, monocyte, and lymphocyte scores in patients with renal cell carcinoma undergoing laparoscopic nephrectomy: A retrospective study. Cancer Med..

[9] Palazón-Bru A., Martín-Pérez F., Mares-García E., Beneyto-Ripoll C., Gil-Guillén V.F., Pérez-Sempere Á., Carbonell-Torregrosa M.Á. (2020). A general presentation on how to carry out a CHARMS analysis for prognostic multivariate models. Stat. Med..

[10] Palazón-Bru A., Mares-García E., López-Bru D., Mares-Arambul E., Folgado-de la Rosa D.M., Carbonell-Torregrosa M.D.L.Á., Gil-Guillén V.F. (2020). A critical appraisal of the clinical applicability and risk of bias of the predictive models for mortality and recurrence in patients with oropharyngeal cancer: Systematic review. Head Neck.

[11] Wolff R.F., Moons K.G.M., Riley R.D., Whiting P.F., Westwood M., Collins G.S., Reitsma J.B., Kleijnen J., Mallett S. (2019). PROBAST: A Tool to Assess the Risk of Bias and Applicability of Prediction Model Studies. Ann. Intern. Med..

[12] Sarrió-Sanz P., Martinez-Cayuelas L., Lumberas B., Sánchez-Caballero L., Palazón-Bru A., Gil-Guillén V.F., Gómez-Pérez L. (2022). Mortality prediction models after radical cystectomy for bladder tumour: A systematic review and critical appraisal. Eur. J. Clin. Investig..

[13] Lawlor A., Lin C., Gómez Rivas J., Ibáñez L., Abad López P., Willemse P.-P., Imran Omar M., Remmers S., Cornford P., Rajwa P. (2024). Predictive Models for Assessing Patients’ Response to Treatment in Metastatic Prostate Cancer: A Systematic Review. Eur. Urol. Open Sci..

[14] Moher D., Liberati A., Tetzlaff J., Altman D.G., PRISMA Group (2009). Preferred reporting items for systematic reviews and meta-analyses: The PRISMA statement. Ann. Intern. Med..

[15] Moons K.G.M., Wolff R.F., Riley R.D., Whiting P.F., Westwood M., Collins G.S., Reitsma J.B., Kleijnen J., Mallett S. (2019). PROBAST: A Tool to Assess Risk of Bias and Applicability of Prediction Model Studies: Explanation and Elaboration. Ann. Intern. Med..

[16] Zhanghuang C., Wang J., Zhang Z., Jin L., Tan X., Mi T., Liu J., Li M., He D. (2021). A Web-Based Prediction Model for Cancer-Specific Survival of Elderly Patients With Clear Cell Renal Cell Carcinoma: A Population-Based Study. Front. Public Health.

[17] Huang G., Liao J., Cai S., Chen Z., Qin X., Ba L., Rao J., Zhong W., Lin Y., Liang Y. (2022). Development and validation of a prognostic nomogram for predicting cancer-specific survival in patients with metastatic clear cell renal carcinoma: A study based on SEER database. Front. Oncol..

[18] Zheng J., Li S., Zhao Y., Tao Z., Li L., Li Z., Li M., Chen X. (2022). Nomograms for predicting overall and cancer-specific survival of patients with chromophobe renal cell carcinoma after nephrectomy: A retrospective SEER-based study. BMJ Open.

[19] Laukhtina E., Schuettfort V.M., D’Andrea D., Pradere B., Quhal F., Mori K., Sari Motlagh R., Mostafaei H., Katayama S., Grossmann N.C. (2022). Selection and evaluation of preoperative systemic inflammatory response biomarkers model prior to cytoreductive nephrectomy using a machine-learning approach. World J. Urol..

[20] Lu Z., He W., Zhou J., Yang C., Xiang R. (2022). Construction and validation of a novel prognostic nomogram for patients with metastatic renal cell carcinoma: A SEER-based study. J. Int. Med. Res..

[21] Ni J., Wang Y., Zhang H., Wang K., Song W., Luo M., Che J., Geng J., Xu Y., Yao X. (2022). Combination of preoperative plasma fibrinogen and neutrophil-to-lymphocyte ratio to predict the prognosis for patients undergoing laparoscopic nephrectomy for renal cell carcinoma. Am. J. Cancer Res..

[22] Wang J., Zhanghuang C., Tan X., Mi T., Liu J., Jin L., Li M., Zhang Z., He D. (2022). Development and Validation of a Competitive Risk Model in Elderly Patients With Chromophobe Cell Renal Carcinoma: A Population-Based Study. Front. Public Health.

[23] Tang J., Wang J., Pan X., Liu X., Zhao B. (2022). A Web-Based Prediction Model for Cancer-Specific Survival of Middle-Aged Patients With Non-metastatic Renal Cell Carcinoma: A Population-Based Study. Front. Public Health.

[24] Zhanghuang C., Wang J., Yao Z., Li L., Xie Y., Tang H., Zhang K., Wu C., Yang Z., Yan B. (2022). Development and Validation of a Nomogram to Predict Cancer-Specific Survival in Elderly Patients With Papillary Renal Cell Carcinoma. Front. Public Health.

[25] Wu K., Liu Z., Shao Y., Li X. (2020). Nomogram Predicting Survival to Assist Decision-Making of Metastasectomy in Patients With Metastatic Renal Cell Carcinoma. Front. Oncol..

[26] Zhu J., Liu Z., Zhang Z., Fan Y., Chen Y., He Z., Zhou L., Jin J., Shen C., Yu W. (2020). Development and internal validation of nomograms for the prediction of postoperative survival of patients with grade 4 renal cell carcinoma (RCC). Transl. Androl. Urol..

[27] Yan H., Wei X., Wu A., Sha Y., Li X., Qi F. (2020). Nomograms for predicting overall and cancer-specific survival in patients with papillary renal cell carcinoma: A population-based study using SEER database. Transl. Androl. Urol..

[28] Zhou Y., Zhang R., Ding Y., Wang Z., Yang C., Tao S., Liang C. (2020). Prognostic nomograms and Aggtrmmns scoring system for predicting overall survival and cancer-specific survival of patients with kidney cancer. Cancer Med..

[29] Chen C., Geng X., Liang R., Zhang D., Sun M., Zhang G., Hou J. (2021). Nomograms-based prediction of overall and cancer-specific survivals for patients with chromophobe renal cell carcinoma. Exp. Biol. Med..

[30] Zhou W., Huang C., Yuan N. (2018). Prognostic nomograms based on log odds of positive lymph nodes for patients with renal cell carcinoma: A retrospective cohort study. Int. J. Surg..

[31] Lyon T.D., Gershman B., Shah P.H., Thompson R.H., Boorjian S.A., Lohse C.M., Costello B.A., Cheville J.C., Leibovich B.C. (2018). Risk prediction models for cancer-specific survival following cytoreductive nephrectomy in the contemporary era. Urol. Oncol. Semin. Orig. Investig..

[32] Leibovich B.C., Lohse C.M., Cheville J.C., Zaid H.B., Boorjian S.A., Frank I., Thompson R.H., Parker W.P. (2018). Predicting Oncologic Outcomes in Renal Cell Carcinoma After Surgery. Eur. Urol..

[33] Zhang G., Wu Y., Zhang J., Fang Z., Liu Z., Xu Z., Fan Y. (2018). Nomograms for predicting long-term overall survival and disease-specific survival of patients with clear cell renal cell carcinoma. OncoTargets Ther..

[34] Hsiao W., Herrel L.A., Yu C., Kattan M.W., Canter D.J., Carthon B.C., Ogan K., Master V.A. (2015). Nomograms incorporating serum C-reactive protein effectively predict mortality before and after surgical treatment of renal cell carcinoma. Int. J. Urol..

[35] Margulis V., Shariat S.F., Rapoport Y., Rink M., Sjoberg D.D., Tannir N.M., Abel E.J., Culp S.H., Tamboli P., Wood C.G. (2013). Development of accurate models for individualized prediction of survival after cytoreductive nephrectomy for metastatic renal cell carcinoma. Eur. Urol..

[36] May M., Ficarra V., Shariat S.F., Zigeuner R., Chromecki T., Cindolo L., Burger M., Gunia S., Feciche B., Wenzl V. (2013). Impact of clinical and histopathological parameters on disease specific survival in patients with collecting duct renal cell carcinoma: Development of a disease specific risk model. J. Urol..

[37] Kutikov A., Egleston B.L., Canter D., Smaldone M.C., Wong Y.N., Uzzo R.G. (2012). Competing risks of death in patients with localized renal cell carcinoma: A comorbidity based model. J. Urol..

[38] Klatte T., Remzi M., Zigeuner R.E., Mannweiler S., Said J.W., Kabbinavar F.F., Haitel A., Waldert M., de Martino M., Marberger M. (2010). Development and external validation of a nomogram predicting disease specific survival after nephrectomy for papillary renal cell carcinoma. J. Urol..

[39] Iimura Y., Saito K., Fujii Y., Kumagai J., Kawakami S., Komai Y., Yonese J., Fukui I., Kihara K. (2009). Development and external validation of a new outcome prediction model for patients with clear cell renal cell carcinoma treated with nephrectomy based on preoperative serum C-reactive protein and TNM classification: The TNM-C score. J. Urol..

[40] Karakiewicz P.I., Suardi N., Capitanio U., Jeldres C., Ficarra V., Cindolo L., de la Taille A., Tostain J., Mulders P.F.A., Bensalah K. (2009). A preoperative prognostic model for patients treated with nephrectomy for renal cell carcinoma. Eur. Urol..

[41] Kanao K., Mizuno R., Kikuchi E., Miyajima A., Nakagawa K., Ohigashi T., Nakashima J., Oya M. (2009). Preoperative prognostic nomogram (probability table) for renal cell carcinoma based on TNM classification. J. Urol..

[42] Cho K.S., Choi Y.D., Kim S.J., Kim C.I., Chung B.H., Seong D.H., Lee D.H., Cho J.S., Cho I.R., Hong S.J. (2008). A comprehensive prognostic stratification for patients with metastatic renal clear cell carcinoma. Yonsei Med. J..

[43] Karakiewicz P.I., Briganti A., Chun F.K.H., Trinh Q.-D., Perrotte P., Ficarra V., Cindolo L., De la Taille A., Tostain J., Mulders P.F.A. (2007). Multi-institutional validation of a new renal cancer-specific survival nomogram. J. Clin. Oncol..

[44] Leibovich B.C., Han Kryu Bui M.H.T., Pantuck A.J., Dorey F.J., Figlin R.A., Belldegrun A. (2003). Scoring algorithm to predict survival after nephrectomy and immunotherapy in patients with metastatic renal cell carcinoma: A stratification tool for prospective clinical trials. Cancer.

[45] Frank I., Blute M.L., Cheville J.C., Lohse C.M., Weaver A.L., Zincke H. (2002). An outcome prediction model for patients with clear cell renal cell carcinoma treated with radical nephrectomy based on tumor stage, size, grade and necrosis: The SSIGN score. J. Urol..

[46] Velis J.M., Ancizu F.J., Hevia M., Merino I., García A., Doménech P., Algarra R., Tienza A., Pascual J.I., Robles J.E. (2017). Risk models for patients with localised renal cell carcinoma. Actas Urol. Esp..

[47] Peng D., He Z.S., Li X.S., Tang Q., Zhang L., Yang K.-W., Yu X.-T., Zhang C.-J., Zhou L.-Q. (2017). A Novel Predictor of Survival with Renal Cell Carcinoma After Nephrectomy. J. Endourol..

[48] Peng D., Zhang C.J., Tang Q., Zhang L., Yang K.-W., Yu X.-T., Gong Y., Li X.-S., He Z.-S., Zhou L.-Q. (2018). Prognostic significance of the combination of preoperative hemoglobin and albumin levels and lymphocyte and platelet counts (HALP) in patients with renal cell carcinoma after nephrectomy. BMC Urol..

[49] Su X., Hou N.N., Yang L.J., Li P.-X., Yang X.-J., Hou G.-D., Gao X.-L., Ma S.-J., Guo F., Zhang R. (2021). The first competing risk survival nomogram in patients with papillary renal cell carcinoma. Sci. Rep..

[50] Tian S., Sun S., Mao W., Qian S., Zhang L., Zhang G., Xu B., Chen M. (2021). Development and Validation of Prognostic Nomogram for Young Patients with Kidney Cancer. Int. J. Gen. Med..

[51] Xiao R., Liu C., He W., Ma L. (2021). Prognostic Factors and a Nomogram Predicting Overall Survival and Cancer-Specific Survival for Patients with Collecting Duct Renal Cell Carcinoma. BioMed Res. Int..

[52] Guo P., Wang Y., Han Y., Wei D., Zhao J., Li M., Jiang Y., Luo Y. (2021). Development and validation of a nomogram to predict postoperative cancer-specific survival of patients with nonmetastatic T3a renal cell carcinoma. Urol. Oncol. Semin. Orig. Investig..

[53] Russo D., Mariani P., Caponio V.C.A., Lo Russo L., Fiorillo L., Zhurakivska K., Lo Muzio L., Laino L., Troiano G. (2021). Development and Validation of Prognostic Models for Oral Squamous Cell Carcinoma: A Systematic Review and Appraisal of the Literature. Cancers.

