# Supplementary material for: Are We Accurately Predicting Mortality in Renal Cancer? A Systematic Review of Prognostic Models
Source: J Clin Med. 2025 Aug 19;14(16):5851. doi: 10.3390/jcm14165851 (PMC12387891; doi:10.3390/jcm14165851)
Supplement: Supplementary file 1 [file jcm-14-05851-s001.zip › jcm-3792165-Supplementary Material 1.pdf]

Supplemental Material 1

EMBASE research

(‘kidney cancer’:ti,ab,kw OR ‘renal cancer’:ti,ab,kw OR ‘kidney tumor’/exp OR ‘kidney cancer’/exp) AND (‘statistical model’/exp OR ‘statistical model’:ti,ab,kw OR ‘nomogram’/exp OR ‘nomogram’:ti,ab,kw OR ‘predictive model’:ti,ab,kw OR ‘scoring system’/exp OR ‘scoring system’:ti,ab,kw OR ‘points system’:ti,ab,kw OR ‘risk score’:ti,ab,kw OR ‘prediction model’:ti,ab,kw) AND (‘recurrent disease’/exp OR ‘recurrence’:ti,ab,kw OR ‘survival’/exp OR ‘survival’:ti,ab,kw OR ‘mortality’/exp OR ‘mortality’:ti,ab,kw OR ‘death’/exp OR ‘death’:ti,ab,kw)

Filters applied: Article, English, Spanish.

Until 4/05/2025

Medline research

("Kidney Neoplasms"[mesh] OR "kidney cancer"[title/abstract] OR "renal cancer"[title/abstract]) AND ("Models, Statistical"[Mesh] OR "Nomograms"[Mesh] OR "predictive model"[title/abstract] OR "nomogram"[title/abstract] OR "scoring system"[title/abstract] OR "points system"[title/abstract] OR "risk score"[title/abstract] OR "prediction model"[title]) AND ("mortality"[title/abstract] OR "mortality"[mesh] OR "death"[title/abstract] OR "death"[mesh] OR "survival"[title/abstract] OR "survival"[mesh] OR "recurrence"[title/abstract] OR "recurrence"[mesh])

Filters applied: Abstract, English, Spanish.

Until 4/05/2025
