# Supplementary material for: Are We Accurately Predicting Mortality in Renal Cancer? A Systematic Review of Prognostic Models
Source: J Clin Med. 2025 Aug 19;14(16):5851. doi: 10.3390/jcm14165851 (PMC12387891; doi:10.3390/jcm14165851)
Supplement: Supplementary file 1 [file jcm-14-05851-s001.zip › jcm-3792165-Supplementary Material 2.pdf]

Table S1: Relevant items extracted from the included studies based on the Critical Appraisal and Data Extraction for Systematic Reviews of Prediction Modelling Studies checklist (Part 1 / 14).

| **CHARMS items** | **References** | | |
|---|---|---|---|
| | **Chen et al., 2025** | **Ni et al., 2024** | **Guo et al., 2024** |
| **Source of data** | Retrospective cohort study | Retrospective cohort study | Retrospective cohort study |
| **Participants** | Consecutive inclusion of patients<br>The total records were randomly split into development and validation cohorts in a ratio of 7:3<br>Patients with N0M0 ccRCC treated with partial or total nephrectomy<br>Treatment is not included as a candidate predictor<br>Baseline data: 2000-2019 | Consecutive inclusion of patients<br>All the participants were used to develop the model<br>Patients with N0M0 RCC treated with partial or total laparoscopic nephrectomy<br>Treatment is not included as a candidate predictor<br>Baseline data: 2014-2019 | SEER data base and external validation on the author's hospital<br>Consecutive inclusion of patients<br><br>All patients were randomly assigned to a training cohort (70%) or a validation cohort (30%). Then an external validation was done with another cohort<br>Papillary RCC<br>Treatment is included as a candidate predictor<br><br>Baseline data: 2004-2015 |
| **Outcome to be predicted** | 1-, 3- and 5-years CSS<br>Measurement: medical records<br>Blinding unknown | 3 and 5-year CSS<br>Measurement: medical records<br>Blinding unknown | 3,5 and 8 -year CSS<br>Measurement: medical records<br>Blinding unknown |
| **Candidate predictors** | Predictors: age, sex, body mass index, tumor location, T stage, necrosis, Fuhrman grade and | Predictors: age, sex, body mass index, hypertension, diabetes, cardiovascular disease, smoking, TNM stage, Fuhrman grade, AJCC | Predictors: age, sex, race, laterality, marital status, months from diagnosis, grade, size, family income, AJCC stage, operation strategy, |

| | | | |
|---|---|---|---|
| | preoperative CRP.<br>Measurement: clinical records at baseline (diagnosis)<br>Blinding of measurement unknown but all of them are objective<br>Continuous predictors: categorizations | stage and PBIS score.<br>Measurement: clinical records at baseline (diagnosis or after the treatment)<br>Blinding of measurement: unknown, but all of them are objective<br>Continuous predictors: categorizations | TNM stage, chemotherapy and radiotherapy<br>Measurement: clinical records at baseline (diagnosis or after the treatment)<br>Blinding of measurement unknown<br>Continuous predictors: categorizations |
| **Sample size** | N= 1599 (E= unknown) to develop the model and 685 to validate it (E= unknown)<br>EPV= unknown | N= 438 (E=36).<br>EPV = 36/21 = 1.71 | N= 752 (E= unknown) to develop the model, 322 (E= unknown) for internal validation and 107 (E= unknown) for external validation. (E= unknown)<br>EPV= unknown |
| **Missing data** | Completed case analysis | Completed case analysis | Completed case analysis |
| **Model development** | Cox proportional hazards models<br>Assumptions were not tested<br>Method for selection of predictors for inclusion in multivariable modeling: based on univariate analysis<br>Method for selection of predictors during multivariable modeling: full model<br>No shrinkage or penalization | Cox proportional hazards models<br>Assumptions were not tested<br>Method for selection of predictors for inclusion in multivariable modeling: based for univariate<br>Method for selection of predictors during multivariable modeling: full model<br>No shrinkage or penalization | Cox proportional hazards models<br>Assumptions were not tested<br>Method for selection of predictors for inclusion in multivariable modeling: based on univariate<br>Method for selection of predictors during multivariable modeling: full model<br>No shrinkage or penalization |
| **Model performance** | Discrimination: c- index and AUC<br>Calibration: calibration curves<br>Classification measures: decision curves analysis | Discrimination: c-index and AUC<br>Calibration: calibration curves<br>Classification measures: decision curves analysis | Discrimination: c- index and AUC<br>Calibration: calibration plots<br>Classification measures: decision curves analysis |
| **Model** | Internal: none | Internal: Bootstrapping | Internal: none |

| | | | |
|---|---|---|---|
| **evaluation** | External: none | | External: none |
| **Results** | Indicated: model coefficients, c- index, AUC, decision curves analysis and calibration curves<br>Not indicated: baseline survival<br>Presentation: nomogram<br>The authors did compare the distribution of the predictors for development and validation data sets | Indicated: model coefficients, c-index, AUC and calibration curves<br>Not indicated: baseline survival<br>Presentation: nomogram | Indicated: model coefficients, c-index, AUC, calibration plots, decision curves analysis<br>Not indicated: baseline survival<br>Presentation: nomogram and risk score<br>The authors did compare the distribution of the predictors for development and validation data sets |
| **Interpretation and discussion** | Exploratory results<br>Comparison with previous models and explanation for the predictors of the final models<br>They analyzed strengths and limitations<br>They discussed generalizability in other areas<br>They stated the need of carrying out an external validation | Exploratory results<br>Comparison with previous models and explanation for the predictors of the final models<br>They analyzed strengths and limitations<br>They discussed generalizability in other areas<br>They stated the need of carrying out an external validation | Exploratory results<br>Comparison with previous models and explanation for the predictors of the final models<br>They analyzed strengths and limitations<br>They discussed generalizability in other areas<br>They stated the need of carrying out an external validation |

Abbreviations: CHARMS, Critical Appraisal and Data Extraction for Systematic Reviews of Prediction Modelling Studies; ccRCC: clear cell Renal Cancer; CRP: C-reactive protein; AJCC: American Joint Committee on Cancer; PBIS: peripheral blood immune score. SEER: Surveillance, Epidemiology and End Results database; CSS: cancer specific survival; AJCC, American Joint Committee on Cancer; E, total number of events; EPV, events-per-variable ratio; N, total number of patients; AUC, area under the curve.

EPV was calculated using the predictors selected during multivariable modeling.

Table 1: Relevant items extracted from the included studies based on the Critical Appraisal and Data Extraction for Systematic Reviews of Prediction Modelling Studies checklist (Part 2/ 14).

| CHARMS items | References | | |
|---|---|---|---|
| | **Tian et al., 2021** | **Zhanghuang et al., 2022** | **Huang el at., 2022** |
| **Source of data** | Retrospective cohort study | Retrospective cohort study | Retrospective cohort study |
| **Participants** | SEER data base<br>The total records were randomly split into development and validation cohorts in a ratio of 2:1<br>Patients diagnosed early onset (age < 46 years old) with renal cancer treated with partial or total nephrectomy<br>Treatment is included as a candidate predictor<br>Baseline data: 2004-2015 | SEER data base<br>Consecutive inclusion of patients<br><br>All patients were randomly assigned to a training cohort (70%) or a validation cohort (30%)<br><br>Elderly patients with ccRCC<br><br>Treatment is included as a candidate predictor<br><br>Baseline data: 2004-2018 | SEER data base<br>Consecutive inclusion of patients<br><br>All patients were randomly assigned to a training cohort (70%) or a validation cohort (30%)<br><br>Metastatic patients with ccRCC<br><br>Treatment is included as a candidate predictor<br><br>Baseline data: 2010-2015 |
| **Outcome to be predicted** | 3 and 5 years CCS<br>Measurement: medical records<br>Blinding unknown | 1, 3 and 5-year CCS<br>Measurement: medical records<br>Blinding unknown | 1,3 and 5 -year CCS<br>Measurement: medical records<br>Blinding unknown |
| **Candidate predictors** | Predictors: age, race, grade, gender, AJCC stage, TNM stage, histology, SEER stage, | Predictors: age, sex, race, marriage, tumor size, year of diagnosis, laterality, grade, TNM stage | Predictors: age, sex, race, laterality, TN stage, surgery, bone, brain, liver and lung metastases |

| | | | |
|---|---|---|---|
| | chemotherapy and radiotherapy<br>Measurement: clinical records at baseline (diagnosis and after the treatment)<br>Blinding of measurement unknown but all of them are objective<br>Continuous predictors: categorizations | and surgery<br>Measurement: clinical records at baseline (diagnosis or after the treatment)<br>Blinding of measurement: unknown, but all of them are objective<br>Continuous predictors: linear | and chemotherapy, radiotherapy and histological grade<br>Measurement: clinical records at baseline (diagnosis or after the treatment)<br>Blinding of measurement unknown<br>Continuous predictors: categorizations |
| **Sample size** | N= 8350 (E= unknown) to develop the model and 4176 to validate it (E= unknown)<br>EPV= unknown | N= 23412 (E= unknown) to develop the model and 10097 to validate it (E= unknown)<br>EPV= unknown | N= 1253 (E= unknown) to develop the model and 537 to validate it (E= unknown)<br>EPV= unknown |
| **Missing data** | Completed case analysis | Completed case analysis | Completed case analysis |
| **Model development** | Cox proportional hazards models<br>Assumptions were not tested<br>Method for selection of predictors for inclusion in multivariable modeling: based on univariate analysis<br>Method for selection of predictors during multivariable modeling: full model<br>No shrinkage or penalization | Cox proportional hazards models<br>Assumptions were not tested<br>Method for selection of predictors for inclusion in multivariable modeling: based for univariate<br>Method for selection of predictors during multivariable modeling: full model<br>No shrinkage or penalization | Cox proportional hazards models<br>Assumptions were not tested<br>Method for selection of predictors for inclusion in multivariable modeling: based on univariate and considering their influence on patient prognosis in terms of professional significance<br>Method for selection of predictors during multivariable modeling: full model<br>No shrinkage or penalization |
| **Model performance** | Discrimination: c- index and AUC<br>Calibration: calibration curves<br>Classification measures: decision curves analysis | Discrimination: c-index and AUC<br>Calibration: calibration curves<br>Classification measures: decision curves analysis | Discrimination: c- index and AUC<br>Calibration: calibration plots<br>Classification measures: decision curves analysis, net reclassification improvement and integrated discrimination improvement |
| **Model** | Internal: none | Internal: Bootstrapping | Internal: none |

| | | | |
|---|---|---|---|
| **evaluation** | External: none | External: Bootstrapping | External: none |
| **Results** | Indicated: model coefficients, c- index AUC, decision curves analysis and calibration curves<br>Not indicated: baseline survival<br>Presentation: nomogram<br>The authors did compare the distribution of the predictors for development and validation data sets | Indicated: model coefficients, c-index, AUC and calibration curves<br>Not indicated: baseline survival<br>Presentation: nomogram<br>The authors did compare the distribution of the predictors for development and validation data sets | Indicated: model coefficients, c-index, AUC, calibration plots, decision curves analysis, net reclassification improvement and integrated discrimination improvement<br>Not indicated: baseline survival<br>Presentation: nomogram and risk score<br>The authors did compare the distribution of the predictors for development and validation data sets |
| **Interpretation and discussion** | Exploratory results<br>Comparison with previous models and explanation for the predictors of the final models<br>They analyzed strengths and limitations<br>They discussed generalizability in other areas<br>They stated the need of carrying out an external validation | Exploratory results<br>Comparison with previous models and explanation for the predictors of the final models<br>They analyzed strengths and limitations<br>They discussed generalizability in other areas<br>They stated the need of carrying out an external validation | Exploratory results<br>Comparison with previous models and explanation for the predictors of the final models<br>They analyzed strengths and limitations<br>They discussed generalizability in other areas<br>They stated the need of carrying out an external validation |

Abbreviations: CHARMS, Critical Appraisal and Data Extraction for Systematic Reviews of Prediction Modelling Studies; ccRCC: clear cell Renal Cancer; SEER: Surveillance, Epidemiology and End Results database; CSS: cancer specific survival; AJCC, American Joint Committee on Cancer; E, total number of events; EPV, events-per-variable ratio; N, total number of patients; AUC, area under the curve.

EPV was calculated using the predictors selected during multivariable modeling.

Table 1: Relevant items extracted from the included studies based on the Critical Appraisal and Data Extraction for Systematic Reviews of Prediction Modelling Studies checklist (Part 3 / 14).

| **CHARMS items** | **References** | | |
|---|---|---|---|
| | **Zheng et al., 2022** | **Laukhtina et al., 2021** | **Lu et al., 2022** |
| **Source of data** | Retrospective cohort study | Retrospective cohort study | Retrospective cohort study |
| **Participants** | SEER data base<br>The patients were randomly divided into two cohorts, including the training cohort with 70% and the validation cohort with 30% of patients. And also, they validated them in another single-centre external validation cohort from China<br><br>chRCC patients after nephrectomy<br><br>Treatment is included as a candidate predictor<br><br>Baseline data: development and internal validation 2010-2015 and external validation 2010-2020 | Consecutive inclusion of patients<br><br>All patients were randomly assigned to a training cohort (65%) or a validation cohort (35%)<br><br>mRCC patients treated with CN at tertiary centers in the USA and Europe<br><br>Treatment is not included as a candidate predictor<br><br>Baseline data: not indicated | SEER data base<br>Consecutive inclusion of patients<br><br>Randomly divided into the training cohort and validation group with a ratio of 1:1.<br><br>Metastatic patients with RCC<br><br>Treatment is included as a candidate predictor<br><br>Baseline data: 2010-2015 |
| **Outcome to be predicted** | 3 and 5 years CCS<br>Measurement: medical records and by | 2 year CCS<br>Measurement: medical records | 1,2 and 3 -year CCS<br>Measurement: medical records |

| | | | |
|---|---|---|---|
| | pathology results<br>Blinding unknown but clinical data and follow-up information were collected by two independent researchers | Blinding unknown | Blinding unknown |
| **Candidate predictors** | Predictors: age at diagnosis, race, sex, grade, TNM stage, SEER stage, surgery method, tumour size and marital status.<br>Measurement: clinical records at baseline (diagnosis and after the treatment)<br>Blinding of measurement unknown but all of them are objective<br>Continuous predictors: categorizations | Predictors: age, hemoglobin, N and M stage, histologic, album-globulin ratio, systemic immune- inflammation index and De Ritis ratio<br>Measurement: clinical records at baseline (diagnosis or after the treatment)<br>Blinding of measurement: unknown, but all of them are objective<br>Continuous predictors: categorizations | Predictors: age, race, sex, laterality, grade, histological type, tumor size, TN stage, metastatic sites, median household income, residential area and surgery<br>Measurement: clinical records at baseline (diagnosis or after the treatment)<br>Blinding of measurement unknown<br>Continuous predictors: categorizations |
| **Sample size** | N= 1970 (E= 37) to develop the model, 840 to internal validation (E= 19) and 124 for Chinese external validation (E=5)<br><br>EPV develop the model = 37/24=1.54<br>EPV internal validation = 19/24=0.79<br>EPV external validation = 5/24=0.2 | N= 400 (E= unknown) to develop the model and 213 to validate it (E= unknown)<br>EPV= unknown | N= 601 (E= unknown) to develop the model and 600 to validate it (E= unknown)<br>EPV= unknown |
| **Missing data** | Completed case analysis | Not indicated | Completed case analysis |
| **Model development** | Least absolute shrinkage model<br>Assumptions were not tested<br>Method for selection of predictors for inclusion in multivariable modeling: The least absolute shrinkage and selection operator regression | Cox proportional hazards models<br>Assumptions were not tested<br>Method for selection of predictors for inclusion in multivariable modeling: The absolute shrinkage and selection operator (LASSO) | Cox proportional hazards models<br>Assumptions were not tested<br>Method for selection of predictors for inclusion in multivariable modeling: based on univariate<br>Method for selection of predictors during |

| | | | |
|---|---|---|---|
| | was performed to screen the prognostic factors<br><br>Method for selection of predictors during multivariable modeling: full model<br>The least absolute shrinkage and selection operator was used | approach<br>Method for selection of predictors during multivariable modeling: full model<br>The least absolute shrinkage and selection operator was used | multivariable modeling: full model<br>No shrinkage or penalization |
| **Model performance** | Discrimination: c- index and AUC<br>Calibration: calibration curves<br>Classification measures: none | Discrimination: c-index and AUC<br>Calibration: calibration plots<br>Classification measures: decision curves analysis | Discrimination: c- index and AUC<br>Calibration: none<br>Classification measures: decision curves analysis |
| **Model evaluation** | Internal: none<br>External: none | Internal: Bootstrapping<br>External: Bootstrapping | Internal: Bootstrapping<br>External: Bootstrapping |
| **Results** | Indicated: model coefficients, c- index AUC and calibration curves<br>Not indicated: baseline survival<br>Presentation: nomogram<br>The authors did compare the distribution of the predictors for development and validation data sets | Indicated: model coefficients, c-index, AUC, calibration curves and decision curves analysis<br>Not indicated: baseline survival<br>Presentation: nomogram<br>The authors did compare the distribution of the predictors for development and validation data sets | Indicated: model coefficients, c-index, AUC and decision curves analysis<br>Not indicated: baseline survival<br>Presentation: nomogram<br>The authors did compare the distribution of the predictors for development and validation data sets |
| **Interpretation and discussion** | Exploratory results<br>Comparison with previous models and explanation for the predictors of the final models | Exploratory results<br>Comparison with previous models and explanation for the predictors of the final models | Confirmatory results<br>Comparison with previous models and explanation for the predictors of the final models |

| | | | |
|---|---|---|---|
| | They analyzed strengths and limitations<br>They discussed generalizability in other areas<br>They stated the need of carrying out another external validation | They analyzed strengths and limitations<br>They discussed generalizability in other areas<br>They stated the need of carrying out an external validation | They analyzed strengths and limitations<br>They discussed generalizability in other areas<br>They didn´t stated the need of carrying out an external validation |

Abbreviations: CHARMS, Critical Appraisal and Data Extraction for Systematic Reviews of Prediction Modelling Studies; mRCC: metastatic Clear Cell renal carcinoma; chRCC: chromophobe Renal Cancer; RCC: renal cell carcinoma; SEER: Surveillance, Epidemiology and End Results database; CSS: cancer specific survival; E, total number of events; EPV, events-per-variable ratio; N, total number of patients; AUC, area under the curve.

EPV was calculated using the predictors selected during multivariable modeling.

Table 1: Relevant items extracted from the included studies based on the Critical Appraisal and Data Extraction for Systematic Reviews of Prediction Modelling Studies checklist (Part 4 / 14).

| **CHARMS items** | **References** | | |
|---|---|---|---|
| | **Ni et al., 2022** | **Wang et al., 2022** | **Tang et al., 2022** |
| **Source of data** | Retrospective cohort study | Retrospective cohort study | Retrospective cohort study |
| **Participants** | Consecutive inclusion of patients<br>All the participants were used to develop the model<br>Patients with RCC and laparoscopic nephrectomy<br>Treatment is not included as a candidate predictor<br>Baseline data: 2014-2019 | SEER data base<br>The total records were randomly split into development and validation cohorts in a ratio of 70/30.<br>Elderly patients diagnosed with chromophobe renal cell cancer<br>Treatment is included as a candidate predictor<br>Baseline data: 2004-2018 | SEER data base<br>The total records were randomly split into development and validation cohorts in a ratio of 70/30.<br>Middle-aged patients diagnosed with non metastatic renal cell cancer<br>Treatment is included as a candidate predictor<br>Baseline data: 2010-2018 |
| **Outcome to be predicted** | 3- and 5-year CCS<br>Measurement: medical records<br>Blinding unknown | 1, 3 and 5 years CCS<br>Measurement: medical records<br>Blinding unknown | 1-, 3- and 5 -year CCS<br>Measurement: medical records<br>Blinding unknown |
| **Candidate predictors** | Predictors: age, gender, BMI, hypertension, diabetes, cardiovascular disease, smoking, TNM stage, Fuhrman grade, fibrinogen, NLR, PLR and SII.<br>Measurement: clinical records at baseline (diagnosis)<br>Blinding of measurement unknown, but all of | Predictors: age, sex, race, marital status, year of diagnosis, tumor size, laterality, grade, TNM stage and surgery<br>Measurement: clinical records at baseline (diagnosis)<br>Blinding of measurement unknown but all of | Predictors: age, race, sex, marital status, year of diagnosis, tumor size, T stage, grade, laterality, histology type and surgery<br>Measurement: clinical records at baseline (diagnosis)<br>Blinding of measurement unknown |

| | | | |
|---|---|---|---|
| | them are objective<br>Continuous predictors: categorizations | them are objective<br>Continuous predictors: linear | Continuous predictors: linear |
| **Sample size** | N= 425 (E= unknown)<br>EPV= unknown | N= 2474 (E= unknown) to develop the model and 1048 to validate it (E= unknown)<br>EPV= unknown | N= 18990 (E= unknown) to develop the model, 8083 to validate it (E= unknown)<br>EPV= unknown |
| **Missing data** | Complete-case analysis | Complete-case analysis | Complete-case analysis |
| **Model development** | Cox proportional hazards regression model<br>Assumptions were not tested<br>Method for selection of predictors for inclusion in multivariable modeling: based on univariate analysis<br>Method for selection of predictors during multivariable modeling: full model<br>No shrinkage or penalization | Competing risk model<br>Assumptions were not tested<br>Method for selection of predictors for inclusion in multivariable modeling: based on univariate analysis<br>Method for selection of predictors during multivariable modeling: full model<br>No shrinkage or penalization | Cox proportional hazards models<br>Assumptions were not tested<br>Method for selection of predictors for inclusion in multivariable modeling: based on univariate analysis<br>Method for selection of predictors during multivariable modeling: full model<br>No shrinkage or penalization |
| **Model performance** | Discrimination: C-index and AUC<br>Calibration: calibration curves<br>Classification measures: DCA | Discrimination: c- index and AUC<br>Calibration: calibration curves<br>Classification measures: DCA | Discrimination: c- index and AUC<br>Calibration: calibration curves<br>Classification measures: DCA |
| **Model evaluation** | Internal: bootstrapping | Internal: none<br>External: none | Internal: bootstrapping<br>External: bootstrapping |
| **Results** | Indicated: model coefficients, C-index, calibration curves, AUC and DCA<br>Not indicated: baseline survival<br>Presentation: nomogram | Indicated: model coefficients, c- index, AUC, calibration curves and DCA<br>Not indicated: baseline survival<br>Presentation: nomogram<br>The authors did compare the distribution of the predictors for development and validation data | Indicated: model coefficients, AUC, c-index, calibration curves and DCA<br>Not indicated: baseline survival<br>Presentation: nomogram and risk groups<br>The authors did compare the distribution of the predictors for development and validation data |

| | | | |
|---|---|---|---|
| | | sets | sets |
| **Interpretation and discussion** | Exploratory results<br>Comparison with previous models and explanation for the predictors of the final models<br>They analyzed strengths and limitations<br>They discussed generalizability in other areas<br>They stated the need of carrying out an external validation | Exploratory results<br>Comparison with previous models and explanation for the predictors of the final models<br>They analyzed strengths and limitations<br>They discussed generalizability in other areas<br>They stated the need of carrying out an external validation | Exploratory results<br>Comparison with previous models and explanation for the predictors of the final models<br>They analyzed strengths and limitations<br>They discussed generalizability in other areas<br>They stated the need of carrying out an external validation |

Abbreviations: CHARMS, Critical Appraisal and Data Extraction for Systematic Reviews of Prediction Modelling Studies; RCC: clear cell Renal Cancer; SEER: Surveillance, Epidemiology and End Results database; CSS: cancer specific survival; BMI: body mass index; NLR: neutrophile to lymphocyte ratio; PLR: platelets to lymphocyte ratio; SII: systemic immune-inflammation index; E, total number of events; EPV, events-per-variable ratio; N, total number of patients; AUC, area under the curve; DCA: applying decision curve analysis.

EPV was calculated using the predictors selected during multivariable modeling.

Table 1: Relevant items extracted from the included studies based on the Critical Appraisal and Data Extraction for Systematic Reviews of Prediction Modelling Studies checklist (Part 4 / 14).

| **CHARMS items** | ***Zhanghuang* et al., 2022** | **Guo et al., 2021** |
|---|---|---|
| **Source of data** | Retrospective cohort study | Retrospective cohort study |
| **Participants** | SEER data base<br>The total records were randomly split into development and validation cohorts in a ratio of 70/30<br>Elderly patients diagnosed with papillary renal cell cancer<br>Treatment is included as a candidate predictor<br>Baseline data: 2004-2018 | SEER data base<br>The total records were randomly split into development and validation cohorts in a ratio of 7/3<br>Patients with T3aN0-1M0 clear-cell, papillary and chromophobe RCC were included<br>Treatment is included as a candidate predictor<br>Baseline data: 2010-2018 |
| **Outcome to be predicted** | 1, 3- and 5-years CCS<br>Measurement: medical records<br>Blinding unknown | 3 and 5 years CCS<br>Measurement: medical records<br>Blinding unknown |
| **Candidate predictors** | Predictors: age, sex, race, marital status, year of diagnosis, laterality, grade, TNM stage, surgery, chemotherapy and radiotherapy<br>Measurement: clinical records at baseline (diagnosis)<br>Blinding of measurement unknown but all of them are objective | Predictors: age, sex, race, laterality, tumor size, surgery type, grade, histological type, sarcomatoid differentiation, TN stage and nodes removed<br>Measurement: clinical records at baseline (diagnosis)<br>Blinding of measurement unknown but all of |

| | | |
|---|---|---|
| | Continuous predictors: categorizations | them are objective<br>Continuous predictors: categorization |
| **Sample size** | N= 9250 (E= unknown) to develop the model and 3855 to validate it (E= unknown)<br>EPV= unknown | N= 4055 (E= 491) to develop the model and 1736 to validate it (E= 203)<br>EPV develop the model= 491/28=17,53<br>EPV internal validation= 203/28=7,25 |
| **Missing data** | Complete-case analysis | Complete-case analysis |
| **Model development** | Cox proportional hazards models<br>Assumptions were not tested<br>Method for selection of predictors for inclusion in multivariable modeling: based on univariate analysis<br>Method for selection of predictors during multivariable modeling: full model<br>No shrinkage or penalization | Cox proportional hazards models<br>Assumptions were not tested<br>Method for selection of predictors for inclusion in multivariable modeling: based on univariate analysis<br>Method for selection of predictors during multivariable modeling: full model<br>No shrinkage or penalization |
| **Model performance** | Discrimination: c- index and AUC<br>Calibration: calibration curves<br>Classification measures: DCA | Discrimination: c- index and AUC<br>Calibration: calibration curves<br>Classification measures: DCA |
| **Model evaluation** | Internal: bootstrapping<br>External: bootstrapping | Internal: bootstrapping<br>External: bootstrapping |
| **Results** | Indicated: model coefficients, c- index, AUC, calibration curves and DCA<br>Not indicated: baseline survival<br>Presentation: nomogram and risk score<br>The authors did compare the distribution of the | Indicated: model coefficients, c- index, AUC, calibration curves and DCA<br>Not indicated: baseline survival<br>Presentation: nomogram<br>The authors did compare the distribution of the |

| | | |
|---|---|---|
| | predictors for development and validation data sets | predictors for development and validation data sets |
| **Interpretation and discussion** | Exploratory results<br>Comparison with previous models and explanation for the predictors of the final models<br>They analyzed strengths and limitations<br>They discussed generalizability in other areas<br>They stated the need of carrying out an external validation | Exploratory results<br>Comparison with previous models and explanation for the predictors of the final models<br>They analyzed strengths and limitations<br>They discussed generalizability in other areas<br>They stated the need of carrying out an external validation |

Abbreviations: CHARMS, Critical Appraisal and Data Extraction for Systematic Reviews of Prediction Modelling Studies; RCC: clear cell Renal Cancer; SEER: Surveillance, Epidemiology and End Results database; CSS: cancer specific survival; E, total number of events; EPV, events-per-variable ratio; N, total number of patients; AUC, area under the curve; DCA: applying decision curve analysis.

EPV was calculated using the predictors selected during multivariable modeling.

Table 1: Relevant items extracted from the included studies based on the Critical Appraisal and Data Extraction for Systematic Reviews of Prediction Modelling Studies checklist (Part 6 / 14).

| **CHARMS items** | **References** | | |
|---|---|---|---|
| | **Leibovich et al., 2003** | **Su et al., 2021** | **Xiao el at., 2021** |
| **Source of data** | Retrospective cohort study | Retrospective cohort study | Retrospective cohort study |
| **Participants** | Consecutive inclusion of patients<br>All the patients were used to develop the model<br>Patients diagnosed with renal metastases at presentation who were treated with radical nephrectomy and immunotherapy<br><br>Treatment is not included as a candidate predictor<br><br>Baseline data: 1989-2000 | SEER data base<br>Consecutive inclusion of patients<br>All the patients were used to develop the model<br><br>Patients diagnosed with papillary renal cancer treated with a partial or total nephrectomy<br><br>Treatment is not included as a candidate predictor<br><br>Baseline data: 2010-2016 | Consecutive inclusion of patients<br>All the patients were used to develop the model<br><br>Patients diagnosed with collecting duct renal cell cancer treated with a partial or total nephrectomy<br><br>Treatment is included as a candidate predictor<br><br>Baseline data: 2004-2015 |
| **Outcome to be predicted** | 1,3 and 5- year CSS<br>Measurement: medical records<br>Blinding unknown | 2, 3 and 5-year CSS<br>Measurement: medical records<br>Blinding unknown | 1,3 and 5 -year CSS<br>Measurement: medical records<br>Blinding unknown |
| **Candidate predictors** | Predictors: age, gender, TN stage, tumor size, grade, ECOG, sarcomatoid histology, location of metastasis, thyroid-stimulating hormone and | Predictors: age, sex, raze, tumor side, pathological grade, T and N stage and bone, liver or lung metastases<br>Measurement: clinical records at baseline | Predictors: age at diagnosis, race, gender, tumor laterality, year of diagnosis, marital status, tumor grade, tumor size, AJCC stage, TNM stage, surgical type, radiotherapy and |

| | | | |
|---|---|---|---|
| | constitutional symptoms<br><br>Measurement: clinical records at baseline (treatment)<br>Blinding of measurement unknown<br>Continuous predictors: categorizations | (diagnosis or after the treatment)<br>Blinding of measurement: unknown, but all of them are objective<br>Continuous predictors: categorizations | chemotherapy<br>Measurement: clinical records at baseline (diagnosis or after the treatment)<br>Blinding of measurement unknown<br>Continuous predictors: linear |
| **Sample size** | N= 173 (E= 123)<br>EPV= 123/19=6.47 | N= 5993 (E= 298)<br>EPV= 298/17=17.53 | N= 324 (E= 208)<br>EPV= 208/23=9.04 |
| **Missing data** | No indicated | Completed case analysis | Completed case analysis |
| **Model development** | Cox proportional hazards models<br>Assumptions were not tested<br>Method for selection of predictors for inclusion in multivariable modeling: based on univariate<br>Method for selection of predictors during multivariable modeling: stepwise backward selection<br>No shrinkage or penalization | Competing risk model<br>Assumptions were not tested<br>Method for selection of predictors for inclusion in multivariable modeling: based for univariate<br>Method for selection of predictors during multivariable modeling: stepwise backward selection<br>No shrinkage or penalization | Cox proportional hazards models<br>Assumptions were not tested<br>Method for selection of predictors for inclusion in multivariable modeling: based on univariate<br>Method for selection of predictors during multivariable modeling: full model<br>No shrinkage or penalization |
| **Model performance** | Discrimination: none<br>Calibration: not indicated<br>Classification measures: none | Discrimination: AUC<br>Calibration: calibration plots<br>Classification measures: none | Discrimination: c- index<br>Calibration: calibration plots<br>Classification measures: none |
| **Model evaluation** | Not indicated | Bootstrapping | Bootstrapping |
| **Results** | Indicated: model coefficients and calibration<br>Not indicated: baseline survival<br>Presentation: risk score | Indicated: model coefficients, AUC and calibration plots<br>Not indicated: baseline survival | Indicated: model coefficients, c-index and calibration plots<br>Not indicated: baseline survival |

| | | | |
|---|---|---|---|
| | | Presentation: nomogram | Presentation: nomogram |
| **Interpretation and discussion** | Exploratory results<br>Comparison with previous models and explanation for the predictors of the final models<br>They didn´t analyzed strengths and limitations<br>They didn´t discussed generalizability in other areas<br>They didn´t state the need of carrying out an external validation | Exploratory results<br>Comparison with previous models and explanation for the predictors of the final models<br>They analyzed strengths and limitations<br>They discussed generalizability in other areas<br>They stated the need of carrying out an external validation | Exploratory results<br>Comparison with previous models and explanation for the predictors of the final models<br>They analyzed strengths and limitations<br>They discussed generalizability in other areas<br>They stated the need of carrying out an external validation |

Abbreviations: CHARMS, Critical Appraisal and Data Extraction for Systematic Reviews of Prediction Modelling Studies; SEER: Surveillance, Epidemiology and End Results database; CSS: cancer specific survival; AJCC: American Joint Committee on Cancer; E, total number of events; EPV, events-per-variable ratio; N, total number of patients; AUC, area under the curve.

EPV was calculated using the predictors selected during multivariable modeling.

Table 1: Relevant items extracted from the included studies based on the Critical Appraisal and Data Extraction for Systematic Reviews of Prediction Modelling Studies checklist (Part 7 / 14).

| CHARMS items | References | | |
|---|---|---|---|
| | **Zhu et al., 2020** | **Yan et al., 2020** | **Zhou et al., 2020** |
| **Source of data** | Retrospective cohort study | Retrospective cohort study | Retrospective cohort study |
| **Participants** | Consecutive inclusion of patients<br>All the participants were used to develop the model<br>Patients diagnosed of grade 4 renal cancer and treated with partial or radical nephrectomy<br>Targeted therapy is included as a candidate predictor<br>Baseline data: 2013-2018 | SEER data base<br>The total records were randomly split into development and validation cohorts in a ratio of 7:3<br>Patients diagnosed with papillary renal cancer<br>Surgery, chemotherapy, radiotherapy and lymph node removal are included as a candidate predictor<br>Baseline data: 2010-2014 | SEER data base<br><br>Consecutive inclusion of patients in three cohort: one to develop, another to validate 1 year nomogram and one more to validate three year nomogram<br><br>Patients diagnosed with renal cancer<br>Surgery is included as a candidate predictor<br>Baseline data: 2005-2015 to develop the model; 2012-2013 to validate 1 year nomogram and 2014-2015 to validate three year nomogram |
| **Outcome to be predicted** | 1, 3- and 5-year CSS<br>Measurement: medical records<br>Blinding unknown | 3 and 5 years CSS<br>Measurement: medical records<br>Blinding unknown | 1-, 3- and 5 -year CSS<br>Measurement: medical records<br>Blinding unknown |
| **Candidate predictors** | Predictors: Aspartate aminotransferase, fibrinogen, systemic symptom, adrenal invasion, tumor size, targeted therapy, sarcomatous differentiation, metastasis, lymph | Predictors: age, sex, laterality, race, TNM, surgery, lymph node removal, RT, chemotherapy, marital status and insurance | Predictors: age, gender, marital status, grade, T stage, M stage, surgery, race, N stage and pathology<br>Measurement: clinical records at baseline |

| | | | |
|---|---|---|---|
| | node metastatis, RCC/non RCC, tumor thrombus, vascular invasion, necrosis, IMDC risk group, MSKCC risk group<br>Measurement: clinical records at baseline (diagnosis)<br>Blinding of measurement unknown, but all of them are objective<br>Continuous predictors: categorizations | status<br>Measurement: clinical records at baseline (diagnosis)<br>Blinding of measurement unknown but all of them are objective<br>Continuous predictors: categorizations | (diagnosis)<br>Blinding of measurement unknown<br>Continuous predictors: categorizations |
| **Sample size** | N= 133 (E= unknown)<br>EPV= unknown | N= 3404 (E= unknown) to develop the model and 1456 to validate it (E= unknown)<br>EPV= unknown | N= 42890 (E= unknown) to develop the model, 13094 to validate three year model (E= unknown) and 14497 to validate one year model and it (E= unknown)<br>EPV= unknown |
| **Missing data** | Complete-case analysis | Complete-case analysis | Complete-case analysis |
| **Model development** | Cox proportional hazards regression model<br>Assumptions were not tested<br>Method for selection of predictors for inclusion in multivariable modeling: based on univariate analysis<br>Method for selection of predictors during multivariable modeling: full model<br>No shrinkage or penalization | Cox proportional hazards models<br>Assumptions were not tested<br>Method for selection of predictors for inclusion in multivariable modeling: based on univariate analysis<br>Method for selection of predictors during multivariable modeling: full model<br>No shrinkage or penalization | Cox proportional hazards models<br>Assumptions were not tested<br>Method for selection of predictors for inclusion in multivariable modeling: based on univariate analysis<br>Method for selection of predictors during multivariable modeling: full model<br>No shrinkage or penalization |
| **Model performance** | Discrimination: C-index<br>Calibration: calibration plots<br>Classification measures: none | Discrimination: c- index and AUC<br>Calibration: calibration curves<br>Classification measures: none | Discrimination: c- index and AUC<br>Calibration: calibration curves<br>Classification measures: sensitivity and |

| | | | |
|---|---|---|---|
| | | | specificity |
| **Model evaluation** | Internal: bootstrapping | Internal: none<br>External: none | Internal: bootstrapping<br>External: bootstrapping |
| **Results** | Indicated: model coefficients, C-index and calibration plots<br>Not indicated: baseline survival<br>Presentation: nomogram | Indicated: model coefficients, c- index AUC, and calibration curves<br>Not indicated: baseline survival<br>Presentation: nomogram<br>The authors did compare the distribution of the predictors for development and validation data sets | Indicated: model coefficients, AUC, c-index, calibration curves<br>Not indicated: baseline survival<br>Presentation: nomogram and risk groups<br>The authors did compare the distribution of the predictors for development and validation data sets |
| **Interpretation and discussion** | Exploratory results<br>Comparison with previous models and explanation for the predictors of the final models<br>They analyzed strengths and limitations<br>They discussed generalizability in other areas<br>They stated the need of carrying out an external validation | Exploratory results<br>Comparison with previous models and explanation for the predictors of the final models<br>They analyzed strengths and limitations<br>They discussed generalizability in other areas<br>They stated the need of carrying out an external validation | Exploratory results<br>Comparison with previous models and explanation for the predictors of the final models<br>They analyzed strengths and limitations<br>They discussed generalizability in other areas<br>They stated the need of carrying out an external validation |

Abbreviations: CHARMS= Critical Appraisal and Data Extraction for Systematic Reviews of Prediction Modelling Studies; CSS = cancer specific survival; RCC= renal cell cancer; E= total number of events; IMDC= International Metastatic RCC Database Consortium; MSKCC= The Memorial Sloan–Kettering Cancer Center; EPV= events-per-variable ratio; RT= radiotherapy N= total number of patients; AUC= area under the curve. EPV was calculated using the predictors selected during multivariable modeling.

Table 1: Relevant items extracted from the included studies based on the Critical Appraisal and Data Extraction for Systematic Reviews of Prediction Modelling Studies checklist (Part 8 / 14).

| **CHARMS items** | **Chen et al., 2020** | **Zhou et al., 2018** |
|---|---|---|
| **Source of data** | Retrospective cohort study | Retrospective cohort study |
| **Participants** | SEER data base<br><br>Consecutive inclusion of patients in two cohorts resulting in 60% (2901 chRCC patients) of the subjects in the training group and 40% (1934 chRCC patients) in the validation group<br><br>Patients diagnosed with chromophobe renal cancer<br><br>Surgery, chemotherapy, radiotherapy are included as a candidate predictor<br>Baseline data: 2004-2015 | SEER data base<br>The match cohort was randomly divided into two equally groups including development and validation cohort, to construct and validate the nomogram, respectively<br>Patients diagnosed with renal cancer treated with a partial or total nephrectomy<br><br>Radiotherapy is included as a candidate predictor<br><br>Baseline data: 2005-2015 |
| **Outcome to be predicted** | 3- and 5-year CSS<br>Measurement: medical records<br>Blinding unknown | 1-, 3- and 5- years CSS<br>Measurement: medical records<br>Blinding unknown |
| **Candidate predictors** | Predictors: gender, age, marital status, race, grade, AJCC stage, TNM stage, SEER stage, surgery, radiotherapy and chemotherapy | Predictors: insurance, histological grade, SEER stage, T stage, radiation and log odds of positive lymph nodes |

| | | |
|---|---|---|
| | Measurement: clinical records at baseline (diagnosis)<br>Blinding of measurement unknown, but all of them are objective<br>Continuous predictors: categorizations | Measurement: clinical records at baseline (diagnosis)<br>Blinding of measurement unknown but all of them are objective<br>Continuous predictors: categorizations |
| **Sample size** | N= 2901 (E= unknown) to develop the model and 1934 to validate it (E= unknown)<br>EPV= unknown | N= 1199 (E= ¿) to develop the model and 1199 to validate it (E=¿)<br>EPV= ¿/12 |
| **Missing data** | Complete-case analysis | Complete-case analysis |
| **Model development** | Cox proportional hazards regression model<br>Assumptions were not tested<br>Method for selection of predictors for inclusion in multivariable modeling: based on univariate analysis<br>Method for selection of predictors during multivariable modeling: full model<br>No shrinkage or penalization | Cox proportional hazards models<br>Assumptions were not tested<br>Method for selection of predictors for inclusion in multivariable modeling: based on univariate analysis<br>Method for selection of predictors during multivariable modeling: full model<br>No shrinkage or penalization |
| **Model performance** | Discrimination: c-index and AUC<br>Calibration: calibration curves<br>Classification measures: decision curve analysis | Discrimination: c- index<br>Calibration: calibration plots<br>Classification measures: decision curve analysis |
| **Model evaluation** | Internal: none<br>External: none | Internal: bootstrapping<br>External: bootstrapping |
| **Results** | Indicated: model coefficients, C-index, AUC, decision curve analysis and calibration plots<br>Not indicated: baseline survival | Indicated: model coefficients, c- index, calibration plots<br>Not indicated: baseline survival |

| | | |
|---|---|---|
| | Presentation: nomogram<br>The authors did compare the distribution of the predictors for development and validation data sets | Presentation: nomogram<br>The authors did compare the distribution of the predictors for development and validation data sets |
| **Interpretation and discussion** | Confirmatory results<br>Comparison with previous models and explanation for the predictors of the final models<br>They analyzed strengths and limitations<br>They not discussed generalizability in other areas<br>They didn´t state the need of carrying out an external validation | Confirmatory results<br>Comparison with previous models and explanation for the predictors of the final models<br>They analyzed strengths and limitations<br>They not discussed generalizability in other areas<br>They didn´t state the need of carrying out an external validation |

Abbreviations: CHARMS, Critical Appraisal and Data Extraction for Systematic Reviews of Prediction Modelling Studies; chRCC: chromophobe renal cell cancer; SEER: Surveillance, Epidemiology and End Results database; CSS: cancer specific survival; AJCC: American joint committee on cancer; E, total number of events; EPV, events-per-variable ratio; N, total number of patients; AUC, area under the curve.

EPV was calculated using the predictors selected during multivariable modeling.

Table 1: Relevant items extracted from the included studies based on the Critical Appraisal and Data Extraction for Systematic Reviews of Prediction Modelling Studies checklist (Part 9 / 14).

| **CHARMS items** | **References** | | |
|---|---|---|---|
| | **Peng et al., 2016** | **Peng et al., 2018** | **Wu et al., 2020** |
| **Source of data** | Retrospective cohort study | Retrospective cohort study | Retrospective cohort study |
| **Participants** | Consecutive inclusion of patients<br>All the patients were used to develop the model<br><br>Patients diagnosed with renal cancer treated with a partial or total nephrectomy<br><br>Treatment is not included as a candidate predictor<br>Baseline data: not indicated | Consecutive inclusion of patients<br>All the patients were used to develop the model<br><br>Patients diagnosed with clear renal cell cancer treated with radical nephrectomy<br><br>Treatment is not included as a candidate predictor<br>Baseline data: 2001-2010 | Consecutive inclusion of patients<br>All the patients were used to develop the model<br><br>Patients diagnosed with metastatic renal cell cancer treated with cytoreductive nephrectomy and without metastasis treatment<br><br>Treatment is not included as a candidate predictor<br>Baseline data: 2010-2015 |
| **Outcome to be predicted** | 5 years CSS<br>Measurement: medical records and death certificates<br>Blinding unknown | 3 and 5-years CSS<br>Measurement: medical records<br>Blinding unknown | 1 and 3-year CSS<br>Measurement: medical records<br>Blinding unknown |
| **Candidate predictors** | Predictors: Fuhrman grade, TNM stage, tumor thrombus, sarcomatous differentiation, lymphovascular invasion, necrosis, hypoalbuminemia, serum calcium, alkaline phosphatase, neutrophil count, anemia, | Predictors: age, gender, histological subtype, ASA grade, Fuhrman grade, TNM, sarcomatoid component, lymphovascular invasion, necrosis, hypoalbuminemia, anemia, neutrophil -to-lymphocyte count, platelet-to-lymphocyte count | Predictors: age, sex, race, laterality, TN stage, bone, brain, liver or lung metastases and grade<br><br>Measurement: clinical records at baseline |

| | | | |
|---|---|---|---|
| | neutrophil to lymphocyte ratio, lymphocyte to monocyte ratio and fibrinogen-cholesterol score<br><br>Measurement: clinical records at baseline (diagnosis)<br>Blinding of measurement unknown but all of them are objective<br>Continuous predictors: categorization | and HALP (hemoglobin x albumin x lymphocyte/platelet)<br>Measurement: clinical records at baseline(diagnosis)<br>Blinding of measurement: unknown, but all of them are objective<br>Continuous predictors: categorization | (diagnosis)<br><br>Blinding of measurement unknown<br><br>Continuous predictors: categorization |
| **Sample size** | N= 1360 (E= 139)<br>EPV= 139/17=8.17 | N= 1360 (E= 139)<br>EPV=139/16=8.68 | N= 2332 (E=1373)<br>EPV= 1373/14 = 98 |
| **Missing data** | Not indicated | Not indicated | Complete case analysis |
| **Model development** | Cox proportional hazards models<br>Assumptions were not tested<br>Method for selection of predictors for inclusion in multivariable modeling: based on univariate analysis<br>Method for selection of predictors during multivariable modeling: full model<br>No shrinkage or penalization | Cox proportional hazards models<br>Assumptions were not tested<br>Method for selection of predictors for inclusion in multivariable modeling: based on univariate analysis<br>Method for selection of predictors during multivariable modeling: full model<br>No shrinkage or penalization | Competing risk analyses<br>Assumptions were not tested<br>Method for selection of predictors for inclusion in multivariable modeling: based on univariate analysis<br>Method for selection of predictors during multivariable modeling: full model<br>No shrinkage or penalization |
| **Model performance** | Discrimination: none<br>Calibration: none<br>Classification measures: none | Discrimination: c- index<br>Calibration: calibration curves<br>Classification measures: none | Discrimination: c-index<br>Calibration: calibration curves<br>Classification measures: none |
| **Model evaluation** | None | None | Bootstrapping |

| | | | |
|---|---|---|---|
| **Results** | Indicated: model coefficients<br>Not indicated: baseline survival<br>Presentation: risk score | Indicated: model coefficients, c-index and calibration curves<br>Not indicated: baseline survival<br>Presentation: nomogram | Indicated: model coefficients, c-index and calibration curves<br>Not indicated: baseline survival<br>Presentation: nomogram and risk score |
| **Interpretation and discussion** | Exploratory results<br>Comparison with previous models and explanation for the predictors of the final models<br>They analyzed strengths and limitations<br>They discussed generalizability in other areas<br>They stated the need of carrying out an external validation | Exploratory results<br>Comparison with previous models and explanation for the predictors of the final models<br>They analyzed strengths and limitations<br>They discussed generalizability in other areas<br>They stated the need of carrying out an external validation | Exploratory results<br>Comparison with previous models and explanation for the predictors of the final models<br>They analyzed strengths and limitations<br>They discussed generalizability in other areas<br>They stated the need of carrying out an external validation |

Abbreviations: CHARMS, Critical Appraisal and Data Extraction for Systematic Reviews of Prediction Modelling Studies; CSS: cancer specific survival; E, total number of events; EPV, events-per-variable ratio; N, total number of patients.

EPV was calculated using the predictors selected during multivariable modeling.

Table 1: Relevant items extracted from the included studies based on the Critical Appraisal and Data Extraction for Systematic Reviews of Prediction Modelling Studies checklist (Part 10 / 14).

| **CHARMS items** | **References** | | |
|---|---|---|---|
| | **Leibovich et al., 2018** | **Zhang et al., 2018** | **Hsiao et al., 2015** |
| **Source of data** | Retrospective cohort study | Retrospective cohort study | Retrospective cohort study |
| **Participants** | The Mayo Clinic Nephrectomy Registry<br>Consecutive inclusion of patients<br>All the patients were used to develop three models: papillary, chromophobe and clear cell renal cancer<br>Patients diagnosed with non-metastatic renal cancer treated with a partial or total nephrectomy<br>Treatment is not included as a candidate predictor<br>Baseline data: 1980-2010 | SEER data base<br>All the patients were used to develop the model<br>Patients diagnosed with clear cell renal cancer treated with a partial or total nephrectomy<br>Surgery is included as a candidate predictor<br>Baseline data: 2004-1014 | Consecutive inclusion of patients<br>All the patients were used to develop the model<br>Patients diagnosed with renal cancer treated with a partial or total nephrectomy<br>Treatment is not included as a candidate predictor<br>Baseline data: 2005-2012 |
| **Outcome to be predicted** | 5-, 10- and 15-year CSS<br>Measurement: medical records<br>Blinding unknown | 3-, 5- and 10-year CSS<br>Measurement: medical records<br>Blinding unknown | 3-year CSS<br>Measurement: death certificates and cause of death<br>Blinding unknown |
| **Candidate predictors** | Predictors: age, year of surgery, sex, race, presence of symptoms at diagnosis, smoking status, BMI, ECOG, Charlson, preoperative glomerular filtration rate, preoperative | Predictors: race, sex, age, marital status, grade, TNM, laterality and nephrectomy<br>Measurement: clinical records at baseline | Predictors: preoperative and postoperative CRP levels, time between CRP level and surgery, maximum tumor diameter, age at surgery, TNM stages, RCC grade, angiolymphatic invasion, |

| | | | |
|---|---|---|---|
| | hemoglobin and surgical details (approach, adjacent organs resection and lymph node dissection status), surgical margin, histologic subtype, grade, coagulative necrosis, sarcomatoid differentiation, rhabdoid differentiation, size, perinephric fat invasion, tumor thrombus, extension beyond Gerota´s fascia and lymph node status.<br>Measurement: clinical records at baseline (treatment)<br>Blinding of measurement unknown, but all of them are objective<br>Continuous predictors: categorizations | (diagnosis)<br>Blinding of measurement unknown, but all of them are objective<br>Continuous predictors: categorizations | presence of necrosis and histological subtype<br>Measurement: clinical records at baseline (diagnosis)<br>Blinding of measurement: unknown, but all of them are objective<br>Continuous predictors: categorizations |
| **Sample size** | Clear cell: N=2726 (E= 635); EPV= 635/19= 33.42<br>Papillary: N= 607 (E= 45); EPV= 45/19= 2.36<br>Chromophobe: N= 222 (E= 22); EPV= 22/19= 1.15 | N= 35151 (E= unknown)<br>EPV= unknown | N= 516 (E= 53)<br>EPV= unknown |
| **Missing data** | Not indicated | Complete-case analysis | Missing values in the predictors were multiply imputed |
| **Model development** | Cox proportional hazards regression model and competing risk model<br>Assumptions were not tested<br>Method for selection of predictors for inclusion in multivariable modeling: based on univariate | Cox proportional hazards regression model<br>Assumptions were not tested<br>Method for selection of predictors for inclusion in multivariable modeling: based on univariate | Cox proportional hazards models and competing risk model<br>Assumptions were not tested<br>Method for selection of predictors for inclusion in multivariable modeling: based on univariate |

| | | | |
|---|---|---|---|
| | analysis<br>Method for selection of predictors during multivariable modeling: backward selection (clear cell model), full model (papillary) and not performed (chromophobe)<br>No shrinkage or penalization | analysis<br>Method for selection of predictors during multivariable modeling: full model<br>No shrinkage or penalization | analysis<br>Method for selection of predictors during multivariable modeling: full model and stepdown method<br>No shrinkage or penalization |
| **Model performance** | Discrimination: c-index<br>Calibration: none<br>Classification measures: none | Discrimination: c-index and AUC<br>Calibration: calibration curves<br>Classification measures: none | Discrimination: c- index<br>Calibration: calibration plots<br>Classification measures: none |
| **Model evaluation** | Bootstrapping | Bootstrapping | Bootstrapping |
| **Results** | Indicated: model coefficients, C-index<br>Not indicated: baseline survival<br>Presentation: risk score | Indicated: model coefficients, C-index, AUC, and calibration curves<br>Not indicated: baseline survival<br>Presentation: nomogram | Indicated: model coefficients, c- index, calibration plots<br>Not indicated: baseline survival<br>Presentation: nomogram |
| **Interpretation and discussion** | Exploratory results<br>Comparison with previous models and explanation for the predictors of the final models<br>They analyzed strengths and limitations<br>They discussed generalizability in other areas<br>They stated the need of carrying out an external validation | Exploratory results<br>Comparison with previous models and explanation for the predictors of the final models<br>They analyzed strengths and limitations<br>They discussed generalizability in other areas<br>They stated the need of carrying out an external validation | Exploratory results<br>Comparison with previous models and explanation for the predictors of the final models<br>They analyzed strengths and limitations<br>They discussed generalizability in other areas<br>They stated the need of carrying out an external validation |

Abbreviations: CHARMS, Critical Appraisal and Data Extraction for Systematic Reviews of Prediction Modelling Studies; SEER: Surveillance, Epidemiology and End Results database; CSS: cancer specific survival; ECOG: eastern cooperative oncology group scale;  BMI: body mass index; CRP: C-reactive protein; E, total number of events; EPV, events-per-variable ratio; N, total number of patients; AUC, area under the curve.

EPV was calculated using the predictors selected during multivariable modeling.

Table 1: Relevant items extracted from the included studies based on the Critical Appraisal and Data Extraction for Systematic Reviews of Prediction Modelling Studies checklist (Part 11 / 14).

| **CHARMS items** | **References** | | |
|---|---|---|---|
| | **Lyon et al., 2018** | **Margulis et al., 2012** | **Cho et al., 2008** |
| **Source of data** | Retrospective cohort study | Retrospective cohort study | Retrospective cohort study |
| **Participants** | Consecutive inclusion of patients<br>All the patients were used to develop two models: preoperative and postoperative models<br>Patients diagnosed with metastatic renal cancer treated with partial or total nephrectomy<br>Treatment is included as a candidate predictor<br>Baseline data: 1990-2010 | Consecutive inclusion of patients<br>All the patients were used to develop two models: preoperative and postoperative models<br>Patients diagnosed with metastatic renal cancer treated with cytoreductive nephrectomy<br>Treatment is included as a candidate predictor<br>Baseline data: 1991-2008 | Consecutive inclusion of patients<br>All the patients were used to develop the model<br>Patients diagnosed with metastatic renal cancer treated with nephrectomy and at least one cycle of immunotherapy or a combination<br>Treatment is not included as a candidate predictor<br>Baseline data: 1995-2004 |
| **Outcome to be predicted** | 1, 3 and 5-years CSS<br>Measurement:<br>"If a patient has died, a death certificate is ordered to determine the cause of death" "If the death certificate does not support this, the medical history is reviewed by a urologist to determine the cause of death, a method that has also been employed by others". If a death certificate cannot be obtained, cause of death is verified with the patient's family or local | 6 and 12-months CSS<br>Measurement: medical records<br>Blinding unknown | 1, 3 and 5-years CSS<br>Measurement: medical records<br>Blinding unknown |

| | | | |
|---|---|---|---|
| | physician"<br><br>Blinding unknown | | |
| **Candidate predictors** | Predictors: year of surgery, age, sex, symptoms, smoking, ECOG, Charlson, BMI, calcium, haemoglobine, radiographic lymphadenopathy, radiographic evidence of hemorrhage, necrosis, calcification, extrarenal extension, neovascularity, adrenal involvement, cystic, tumor thrombus, neoadjuvant therapy, number of metastasis sites, location of metastases, type of surgery, concurrent metastasectomy, tumor size, lymph node dissection, adverse RCC histologic suptype, T and N stage, grade, coagulative tumor necrosis and sarcomatoid differentiation<br><br>Measurement: clinical records at baseline (treatment)<br>Blinding of measurement: the pathologist was blinded to patient outcome.<br>Continuous predictors: categorizations | Predictors:<br>- Preoperative model: number of metastatic organ sites, ECOG, time from diagnosis to surgery, preoperative glomerular filtration rate, alkaline phosphatase, lactate dehydrogenase, corrected calcium, albumin, total and fractionated white blood cells, hemoglobin, platelets, hematocrit and Motzer criteria<br>- Postoperative model: included the preoperative variables, as well as pathologic TN stage, lymph node density, lymphovascular invasion, tumor grade, operating room time, concomitant retroperitoneal lymphadenectomy, and receipt of a blood transfusion during surgery.<br>Measurement: clinical records at baseline and after the treatment<br>Blinding of measurement unknown, but all of them are objective<br>Continuous predictors: linear | Predictors: gender, age, constitutional symptoms at presentation, local symptoms at presentation, ECOG, hematocrit, platelet, alkaline phosphatase, aspartate aminotransferase, alanine aminotransferase, and calcium level, T stage, tumor size, nuclear grade, sarcomatoid differentiation, tumor necrosis, N stage, timing of metastasis, site of metastatic disease (lung, bone, brain, and liver), and number of metastatic sites<br><br>Measurement: clinical records at baseline (diagnosis and after the treatment)<br>Blinding of measurement: unknown, but all of them are objective<br>Continuous predictors: categorizations |
| **Sample size** | N = 313 (E = 279) | N preoperative = 601 (E= 215)<br>N postoperative = 574 (E= 215) | N= 197 (E= 127)<br>EPV= 127/24=5.29 |

| | | | |
|---|---|---|---|
| | EPV = 279/46 = 5.06 | EPV Preoperative model: 215/21=10.24<br>EPV Postoperative model: 215/30=7.16 | |
| **Missing data** | Not indicated | Not indicated | Not indicated |
| **Model development** | Cox proportional hazards regression model<br>Assumptions were not tested<br>Method for selection of predictors for inclusion in multivariable modeling: based on univariate analysis<br>Method for selection of predictors during multivariable modeling: backward stepwise selection<br>No shrinkage or penalization | Logistic regression analysis<br>Assumptions were not tested<br>Method for selection of predictors for inclusion in multivariable modeling: forward stepwise variable selection process<br>Method for selection of predictors during multivariable modeling: full model<br>No shrinkage or penalization | Cox proportional hazards models<br>Assumptions were not tested<br>Method for selection of predictors for inclusion in multivariable modeling: based on univariate analysis<br>Method for selection of predictors during multivariable modeling: full model<br>No shrinkage or penalization |
| **Model performance** | Discrimination: c-index<br>Calibration: calibration plots<br>Classification measures: decision curves analysis | Discrimination: none<br>Calibration: calibration curves<br>Classification measures: decision curves analysis | Discrimination: none<br>Calibration: none<br>Classification measures: none |
| **Model evaluation** | Bootstrapping | Overfit using 10-fold crossvalidation | Not indicated |
| **Results** | Indicated: model coefficients, C-index, calibration plots and decision curves analysis<br>Not indicated: baseline survival<br>Presentation: two risks score | Indicated: model coefficients, decision curves analysis and calibration curves<br>Not indicated: baseline survival<br>Presentation: two nomograms | Indicated: model coefficients<br>Not indicated: baseline survival<br>Presentation: risk score |
| **Interpretation and discussion** | Exploratory results<br>Comparison with previous models and explanation for the predictors of the final | Exploratory results<br>Comparison with previous models and explanation for the predictors of the final | Exploratory results<br>Comparison with previous models and explanation for the predictors of the final |

| | | | |
|---|---|---|---|
| | models<br>They analyzed strengths and limitations<br>They discussed generalizability in other areas<br>They stated the need of carrying out an external validation | models<br>They analyzed strengths and limitations<br>They discussed generalizability in other areas<br>They stated the need of carrying out an external validation | models<br>They analyzed strengths and limitations<br>They discussed generalizability in other areas<br>They stated the need of carrying out an external validation |

Abbreviations: CHARMS, Critical Appraisal and Data Extraction for Systematic Reviews of Prediction Modelling Studies; RCC: clear cell Renal Cancer; SEER: Surveillance, Epidemiology and End Results database; CSS: cancer specific survival; BMI: body mass index; ECOG: eastern cooperative oncology group scale; E, total number of events; EPV, events-per-variable ratio; N, total number of patients.

EPV was calculated using the predictors selected during multivariable modeling.

Table 1: Relevant items extracted from the included studies based on the Critical Appraisal and Data Extraction for Systematic Reviews of Prediction Modelling Studies checklist (Part 12 / 14).

| **CHARMS items** | **References** | | |
|---|---|---|---|
| | **May et al., 2013** | **Kutikov et al., 2012** | **Klatte el at., 2010** |
| **Source of data** | Retrospective cohort study | Retrospective cohort study | Retrospective cohort study |
| **Participants** | Consecutive inclusion of patients<br>All the patients were used to develop the model<br>Patients diagnosed with collecting duct renal cancer treated with a partial or total nephrectomy<br><br>Treatment is included as a candidate predictor<br><br>Baseline data: 1992-2010 | SEER data base<br>Consecutive inclusion of patients<br>All the patients were used to develop the model<br><br>Patients diagnosed with renal cancer treated with a partial or total nephrectomy older than 66 years and with a medicare coverage for on years before and after cancer diagnosis<br><br>Treatment is not included as a candidate predictor<br><br>Baseline data: 1995-2005 | Consecutive inclusion of patients<br>Two cohorts were used to develop the model and one cohort for external validation<br>Patients diagnosed with papillary renal cancer treated with a partial or total nephrectomy<br><br>Treatment is not included as a candidate predictor<br><br>Baseline data: 1984-2008 |
| **Outcome to be predicted** | 5- year CSS<br>Measurement: medical records and death certificates<br>Blinding unknown | 5-year CSS<br>Measurement: medical records<br>Blinding unknown | 5 -year CSS<br>Measurement: medical records and death certificates<br>Blinding unknown |
| **Candidate** | Predictors: age, sex, symptoms, ASA score, | Predictors: race, gender, tumor size, age and | Predictors: symptoms, TNM stage, grade, tumor |

| | | | |
|---|---|---|---|
| **predictors** | type of surgery, tumor size, TNM stage, Fuhrman grade, adrenal involvement, positive surgical margins, LVI, sarcomatoid differentiation and tumor necrosis<br>Measurement: clinical records at baseline (treatment)<br>Blinding of measurement unknown<br>Continuous predictors: linear and categorizations | Charlson score<br>Measurement: clinical records at baseline (diagnosis)<br>Blinding of measurement: unknown, but all of them are objective<br>Continuous predictors: categorizations | size, type of papillary tumor, vascular invasion and necrosis<br>Measurement: clinical records at baseline (diagnosis)<br>Blinding of measurement unknown<br>Continuous predictors: linear |
| **Sample size** | N= 95 (E= 53)<br>EPV= 53/16= 3.3 | N= 6665 (E= 466)<br>EPV= 466/9= 51.7 | N= 258 (E= unknown) to develop the model and N= 177 (E= unknown) to validate the model<br>EPV= unknown |
| **Missing data** | No indicated | Complete-case analysis | Completed case analysis |
| **Model development** | Cox proportional hazards models<br>Assumptions were tested<br>Method for selection of predictors for inclusion in multivariable modeling: full model<br>Method for selection of predictors during multivariable modeling: stepwise backward selection<br>No shrinkage or penalization | Competing risk model<br>Assumptions were not tested<br>Method for selection of predictors for inclusion in multivariable modeling: not indicated<br>Method for selection of predictors during multivariable modeling: full model<br>No shrinkage or penalization | Cox proportional hazards models<br>Assumptions were not tested<br>Method for selection of predictors for inclusion in multivariable modeling: full model<br>Method for selection of predictors during multivariable modeling: stepwise backward selection<br>No shrinkage or penalization |
| **Model performance** | Discrimination: c- index<br>Calibration: none<br>Classification measures: none | Discrimination: none<br>Calibration: quintile calibration<br>Classification measures: none | Discrimination: c- index<br>Calibration: calibration plots<br>Classification measures: none |
| **Model** | Bootstrapping | None | Internal: Bootstrapping |

| | | | |
|---|---|---|---|
| **evaluation** | | | External: none |
| **Results** | Indicated: model coefficients and c-index<br>Not indicated: baseline survival<br>Presentation: risk score | Indicated: model coefficients and quintile calibration<br>Not indicated: baseline survival<br>Presentation: nomogram | Indicated: model coefficients, c-index and calibration plots<br>Not indicated: baseline survival<br>Presentation: nomogram.<br>The authors compared the distribution of the predictors for validation and derivation data sets |
| **Interpretation and discussion** | Exploratory results<br>Comparison with previous models and explanation for the predictors of the final models<br>They analyzed strengths and limitations<br>They discussed generalizability in other areas<br>They stated the need of carrying out an external validation | Exploratory results<br>Comparison with previous models and explanation for the predictors of the final models<br>They analyzed strengths and limitations<br>They discussed generalizability in other areas<br>They stated the need of carrying out an external validation | Exploratory results<br>Comparison with previous models and explanation for the predictors of the final models<br>They analyzed strengths and limitations<br>They discussed generalizability in other areas<br>They stated the need of carrying out an external validation |

Abbreviations: CHARMS, Critical Appraisal and Data Extraction for Systematic Reviews of Prediction Modelling Studies; SEER: Surveillance, Epidemiology and End Results database; CSS: cancer specific survival; BMI: body mass index; ASA score: American society of anesthesiologist physical status; LVI: lymphovascular invasion; E, total number of events; EPV, events-per-variable ratio; N, total number of patients.

EPV was calculated using the predictors selected during multivariable modeling.

Table 1: Relevant items extracted from the included studies based on the Critical Appraisal and Data Extraction for Systematic Reviews of Prediction Modelling Studies checklist (Part 13 / 14).

| CHARMS items | References | | |
|---|---|---|---|
| | **Iimura et al., 2008** | **Karakiewicz et al., 2008** | **Kanao et al., 2008** |
| **Source of data** | Retrospective cohort study | Prospective cohort study | Retrospective cohort study |
| **Participants** | Consecutive inclusion of patients in two cohorts: one to develop and another to validate<br><br>Patients diagnosed with clear cell renal cancer and treated with partial or radical nephrectomy<br><br>Treatment is not included as a candidate predictor<br>Baseline data: 1993-2006 | Multicentric study<br><br>Consecutive inclusion of patients in two cohorts: one to develop (patients from 5 hospitals) and another to validate (patients from 7 hospitals)<br><br>Patients diagnosed with renal cancer treated with partial or radical nephrectomy<br><br>Treatment is not included as a candidate predictor<br>Baseline data: 1984-2006 | All the patients were used to develop the model<br><br>Patients diagnosed with renal cancer treated with partial or radical nephrectomy<br><br>Treatment is not included as a candidate predictor<br>Baseline data: 1985-2003 |
| **Outcome to be predicted** | 5-year CSS<br>Measurement: medical records<br>Blinding unknown | 1, 2, 5- and 10-years CSS<br>Measurement: medical records and death certificates<br>Blinding unknown | 1-, 3- and 5 -year CSS<br>Measurement: medical records<br>Blinding unknown |
| **Candidate** | Predictors: age, sex, CRP, TNM stage, size, | Predictors: age, gender, clinical stage, presence | Predictors: TNM stage |

| | | | |
|---|---|---|---|
| **predictors** | nuclear grade and tumor necrosis<br>Measurement: clinical records at baseline (diagnosis)<br>Blinding of measurement unknown, but all of them are objective<br>Continuous predictors: categorizations | of metastases, tumor size and symptoms<br>Measurement: clinical records at baseline (diagnosis)<br>Blinding of measurement unknown but all of them are objective<br>Continuous predictors: linear | Measurement: clinical records at baseline (diagnosis)<br>Blinding of measurement unknown<br>Continuous predictors: none |
| **Sample size** | N= 249 (E= 46) to develop the model and 290 to validate it (E= unknown)<br>EPV= 46/12=3.83 | N= 2474 (E= 535) to develop the model and 1972 to validate it (E= 272)<br>EPV= 535/9=59.4 | N= 545 (E= 60)<br>EPV= 60/7=8.57 |
| **Missing data** | Complete-case analysis | Complete-case analysis | Not indicated |
| **Model development** | Cox proportional hazards regression model<br>Assumptions were not tested<br>Method for selection of predictors for inclusion in multivariable modeling: based on univariate analysis<br>Method for selection of predictors during multivariable modeling: full model<br>No shrinkage or penalization | Cox proportional hazards models and competing risks models<br>Assumptions were tested<br>Method for selection of predictors for inclusion in multivariable modeling: based on univariate analysis<br>Method for selection of predictors during multivariable modeling: full model<br>No shrinkage or penalization | Cox proportional hazards models<br>Assumptions were not tested<br>Method for selection of predictors for inclusion in multivariable modeling: not indicated<br>Method for selection of predictors during multivariable modeling: not indicated<br>No shrinkage or penalization |
| **Model performance** | Discrimination: C-index<br>Calibration: none<br>Classification measures: none | Discrimination: c- index<br>Calibration: calibration plots<br>Classification measures: none | Discrimination: c- index<br>Calibration: calibration plots<br>Classification measures: none |
| **Model evaluation** | Internal: none<br>External: none | Internal: bootstrapping<br>External: none | Internal: bootstrapping |
| **Results** | Indicated: model coefficients and C-index | Indicated: model coefficients, c- index and | Indicated: model coefficients, c-index and |

| | | | |
|---|---|---|---|
| | Not indicated: baseline survival<br>Presentation: risk score<br>The authors did compare the distribution of the predictors for development and validation data sets | calibration curves<br>Not indicated: baseline survival<br>Presentation: nomogram<br>The authors did compare the distribution of the predictors for development and validation data sets | calibration curves<br>Not indicated: baseline survival<br>Presentation: nomogram |
| **Interpretation and discussion** | Exploratory results<br>Comparison with previous models and explanation for the predictors of the final models<br>They analyzed strengths and limitations<br>They discussed generalizability in other areas<br>They stated the need of carrying out an external validation | Confirmatory results<br>Comparison with previous models and explanation for the predictors of the final models<br>They analyzed strengths and limitations<br>They not discussed generalizability in other areas<br>They stated the need of carrying out an external validation | Exploratory results<br>Comparison with previous models and explanation for the predictors of the final models<br>They analyzed strengths and limitations<br>They discussed generalizability in other areas<br>They stated the need of carrying out an external validation |

Abbreviations: CHARMS, Critical Appraisal and Data Extraction for Systematic Reviews of Prediction Modelling Studies; CSS: cancer specific survival; CRP: C-reactive protein; E, total number of events; EPV, events-per-variable ratio; N, total number of patients.

EPV was calculated using the predictors selected during multivariable modeling.

Table 1: Relevant items extracted from the included studies based on the Critical Appraisal and Data Extraction for Systematic Reviews of Prediction Modelling Studies checklist (Part 14 / 14).

| **CHARMS items** | **References** | | |
|---|---|---|---|
| | **Karakiewicz et al., 2007** | **Frank et al., 2002** | **Velis et al., 2017** |
| **Source of data** | Prospective cohort study | Retrospective cohort study | Retrospective cohort study |
| **Participants** | Multicentric study<br><br>Consecutive inclusion of patients in two cohorts: one to develop (patients from 5 hospitals) and another to validate (patients from 6 hospitals)<br>Patients diagnosed with renal cancer treated with a partial or total nephrectomy<br><br>Treatment is not included as a candidate predictor<br>Baseline data: not indicated | Consecutive inclusion of patients<br>All the patients were used to develop the model<br><br>Patients diagnosed with clear renal cell cancer treated with radical nephrectomy<br><br>Treatment is not included as a candidate predictor<br>Baseline data: 1970-1998 | Consecutive inclusion of patients<br>All the patients were used to develop the model<br><br>Patients diagnosed with renal cell N0 M0 cancer treated with radical or partial nephrectomy<br><br>Treatment is not included as a candidate predictor<br><br>Baseline data: 1990-2012 |
| **Outcome to be predicted** | 1, 2, 5 and 10- years CSS<br>Measurement: medical records and death certificates<br>Blinding unknown | 1,3, 5, 7 and 10-years CSS<br>Measurement: medical records<br>Blinding unknown | 5 and 10-year CSS<br>Measurement: medical records<br>Blinding unknown |
| **Candidate predictors** | Predictors: TNM stage, age, sex, tumor size and symptoms, Fuhrman grade and histologic | Predictors: age, gender, smoking, recent onset hypertension, symptomatic, tumor size, TNM, | Predictors: haemoglobine, haematuria, tumor size, tumor grade, collecting system invasion, |

| | | | |
|---|---|---|---|
| | subtype<br>Measurement: clinical records at baseline (diagnosis)<br>Blinding of measurement unknown but all of them are objective<br>Continuous predictors: linear | nuclear grade, histological tumor necrosis, sarcomatoid component, cystic architecture, multifocality and positive surgical margins.<br>Measurement: clinical records at baseline(diagnosis)<br>Blinding of measurement: microscopic slides from all tumor specimens were reviewed by a urological pathologist without knowledge of patient outcome<br>Continuous predictors: categorization | multifocality, tumor necrosis, sarcomatoid component, T stage, perirenal fat invasion, venous thrombus, lymphovascular invasion<br>Measurement: clinical records at baseline (diagnosis)<br>Blinding of measurement unknown<br>Continuous predictors: categorization |
| **Sample size** | N= 2530 (E= 598) to develop the model and 1377 to validate it (E= 168)<br>EPV= 598/16=37.37 | N= 1801 (E= 652)<br>EPV=652/19=34.31 | N= 596 (E=57)<br>EPV= 57/12= 4.25 |
| **Missing data** | Complete-case analysis | Completed- case analysis | Not indicated |
| **Model development** | Cox proportional hazards models and competing risks models<br>Assumptions were tested<br>Method for selection of predictors for inclusion in multivariable modeling: based on univariate analysis<br>Method for selection of predictors during multivariable modeling: full model<br>No shrinkage or penalization | Cox proportional hazards models and<br>Assumptions were not tested<br>Method for selection of predictors for inclusion in multivariable modeling: based on univariate analysis<br>Method for selection of predictors during multivariable modeling: stepwise selection<br>No shrinkage or penalization | Cox proportional hazards models<br>Assumptions were not tested<br>Method for selection of predictors for inclusion in multivariable modeling: based on univariate analysis<br>Method for selection of predictors during multivariable modeling: full model<br>No shrinkage or penalization |
| **Model performance** | Discrimination: c- index<br>Calibration: calibration plots | Discrimination: c- index<br>Calibration: none | Discrimination: none<br>Calibration: none |

| | | | |
|---|---|---|---|
| | Classification measures: none | Classification measures: none | Classification measures: none |
| **Model evaluation** | Internal: bootstrapping<br>External: none | Bootstrapping | none |
| **Results** | Indicated: model coefficients, c- index and calibration curves<br>Not indicated: baseline survival<br>Presentation: nomogram<br>The authors did compare the distribution of the predictors for development and validation data sets | Indicated: model coefficients and c-index<br>Not indicated: baseline survival<br>Presentation: risk score | Indicated: model coefficients<br>Not indicated: baseline survival<br>Presentation: risk score |
| **Interpretation and discussion** | Confirmatory results<br>Comparison with previous models and explanation for the predictors of the final models<br>They analyzed strengths and limitations<br>They not discussed generalizability in other areas<br>They stated the need of carrying out an external validation<br>They stated the need of carrying out an external validation | Exploratory results<br>Comparison with previous models and explanation for the predictors of the final models<br>They analyzed strengths and limitations<br>They discussed generalizability in other areas<br>They stated the need of carrying out an external validation<br>They stated the need of carrying out an external validation | Exploratory results<br>Comparison with previous models and explanation for the predictors of the final models<br>They analyzed strengths and limitations<br>They discussed generalizability in other areas<br>They stated the need of carrying out an external validation<br>They stated the need of carrying out an external validation |

Abbreviations: CHARMS, Critical Appraisal and Data Extraction for Systematic Reviews of Prediction Modelling Studies; CSS: cancer specific survival; E, total number of events; EPV, events-per-variable ratio; N, total number of patients.

EPV was calculated using the predictors selected during multivariable modeling.
