# Supplementary material for: Are We Accurately Predicting Mortality in Renal Cancer? A Systematic Review of Prognostic Models
Source: J Clin Med. 2025 Aug 19;14(16):5851. doi: 10.3390/jcm14165851 (PMC12387891; doi:10.3390/jcm14165851)
Supplement: Supplementary file 1 [file jcm-14-05851-s001.zip › jcm-3792165-Supplementary Material 3.pdf]

PROBAST

(Prediction model study Risk Of Bias Assessment Tool)

Published in Annals of Internal Medicine (freely available):

1. PROBAST: A Tool to Assess the Risk of Bias and Applicability of Prediction Model Studies
2. PROBAST: A Tool to Assess Risk of Bias and Applicability of Prediction Model Studies: Explanation and Elaboration

What does PROBAST assess?

PROBAST assesses both the *risk of bias* and *concerns regarding applicability* of a study that evaluates (develops, validates or updates) a multivariable diagnostic or prognostic prediction model. It is designed to assess primary studies included in a systematic review.

Bias occurs if systematic flaws or limitations in the design, conduct or analysis of a primary study distort the results. For the purpose of prediction modelling studies, we have defined *risk of bias* to occur when shortcomings in the study design, conduct or analysis lead to systematically distorted estimates of a model's predictive performance or to an inadequate model to address the research question. Model predictive performance is typically evaluated using calibration, discrimination and sometimes classification measures, and these are likely inaccurately estimated in studies with high risk of bias. *Applicability* refers to the extent to which the prediction model from the primary study matches your systematic review question, for example in terms of the participants, predictors or outcome of interest.

A primary study may include the development and/or validation or update of more than one prediction model. A PROBAST assessment should be completed for each distinct model that is developed, validated or updated (extended) for making individualised predictions. Where a publication assesses multiple prediction models, only complete a PROBAST assessment for those models that meet the inclusion criteria for your systematic review. Please note that subsequent use of the term "model" includes derivatives of models, such as simplified risk scores, nomograms, or recalibrations of models.

PROBAST is not designed for all multivariable diagnostic or prognostic studies. For example, studies using multivariable models to identify predictors associated with an outcome but not attempting to develop a model for making individualised predictions are not covered by PROBAST.

PROBAST includes four steps.

Step	Task	When to complete
**1**	Specify your systematic review question(s)	Once per systematic review
**2**	Classify the type of prediction model evaluation	Once for each model of interest in each publication being assessed, for each relevant outcome
**3**	Assess risk of bias and applicability	Once for each development and validation of each distinct prediction model in a publication
**4**	Overall judgment	Once for each development and validation of each distinct prediction model in a publication

If this is your first time using PROBAST, we strongly recommend reading the detailed explanation and elaboration (E&E, see link above) paper and to check the examples on www.probast.org

Step 1: Specify your systematic review question

State your systematic review question to facilitate the assessment of the applicability of the evaluated models to your question. *The following table should be completed once per systematic review.*

Criteria	Specify your systematic review question
Intended use of model:	*To predict cancer specific mortality in patients with renal cancer treated with partial or total nephrectomy regardless of TNM stage*
***Participants** including selection criteria and setting:*	*Patients with renal cancer treated with partial or total nephrectomy regardless of TNM stage*
***Predictors** (used in prediction modelling), including types of predictors (e.g. history, clinical examination, biochemical markers, imaging tests), time of measurement, specific measurement issues (e.g., any requirements/ prohibitions for specialized equipment):*	*Predictors used in clinical practice measured when a nephrectomy for renal cancer is indicated*
Outcome to be predicted:	*Cancer specific mortality*

Step 2: Classify the type of prediction model evaluation

Use the following table to classify the evaluation as model development, model validation or model update, or combination. Different signalling questions apply for different types of prediction model evaluation. If the evaluation does not fit one of these classifications then PROBAST should not be used.

Classify the evaluation based on its aim			
**Type of prediction study**	**PROBAST boxes to complete**	**Tick as appropriate**	**Definition for type of prediction model study**
Development only	Development	✓	Prediction model development without external validation. These studies may include internal validation methods, such as bootstrapping and cross-validation techniques.
Development and validation	Development and validation	✗	Prediction model development combined with external validation in other participants in the same article.
Validation only	Validation	✗	External validation of existing (previously developed) model in other participants.

This table should be completed once for each publication being assessed and for each relevant outcome in your review.

**Publication reference**	Wei Chen, Hajime Tanaka, Masaki Kobayashi, Shohei Fukuda, Akinori Nakayama, Margaret F. Meagher, Rachel Greenwald, Benjamin Schmeusser, Edouard Nicase, Yuma Waseda. Development and validation of nomograms and integrated software incorporating preoperative C-reactive protein level for prognostic prediction of nonmetastatic clear cell renal cell carcinoma: Results from the International Marker Consortium for Renal Cancer (INMARC) Registry, 2025: 44-64.<br>World Journal of Urology
**Models of interest**	Nomogram
**Outcome of interest**	*Cancer specific mortality*

Step 3: Assess risk of bias and applicability

PROBAST is structured as four key domains. Each domain is judged for risk of bias (low, high or unclear) and includes signalling questions to help make judgements. Signalling questions are rated as yes (Y), probably yes (PY), probably no (PN), no (N) or no information (NI). All signalling questions are phrased so that "yes" indicates absence of bias. Any signalling question rated as "no" or "probably no" flags the potential for bias; you will need to use your judgement to determine whether the domain should be rated as "high", "low" or "unclear" risk of bias. The guidance document contains further instructions and examples on rating signalling questions and risk of bias for each domain.

The first three domains are also rated for concerns regarding applicability (low/ high/ unclear) to your review question defined above.

Complete all domains separately for each evaluation of a distinct model. Shaded boxes indicate where signalling questions do not apply and should not be answered.

DOMAIN 1: Participants			
**A. Risk of Bias**			
Describe the sources of data and criteria for participant selection:<br><br>"Data were retrospectively collected from the International Marker Consortium for Renal Cancer (INMARC) registry from 2000 to 2019, based on an international collaboration of four institutions: Institute of Science Tokyo, University of California San Diego, Emory University, and Dokkyo Medical University Saitama Medical Center. The INMARC database encompasses patient characteristics, perioperative data,and follow-up information for individuals who underwent partial and radical nephrectomy for renal mass"<br>"The inclusion criteria were as follows: (1) availability of preoperative standard CRP value measured within one month before surgery, (2) pathological confirmation of ccRCC diagnosis,and (3) absence of adjuvant or neoadjuvant therapy"			
		Dev	Val
1.1 Were appropriate data sources used, e.g. cohort, RCT or nested case-control study data?		Y	Y
1.2 Were all inclusions and exclusions of participants appropriate?		Y	Y
**Risk of bias introduced by selection of participants**	**RISK:**<br>*(low/ high/ unclear)*	Low	Low
Rationale of bias rating:<br>Cohort study include patients with clear cell Renal Cell Carcinoma who underwent surgery.			
**B. Applicability**			
Describe included participants, setting and dates:<br>"A total of 2284 patients were enrolled in the study, with1599 (70%) and 685 (30%) randomly assigned "<br>"The inclusion criteria were as follows: (1) availability of preoperative standard CRP value measured within one month before surgery, (2) pathological confirmation of ccRCC diagnosis,and (3) absence of adjuvant or neoadjuvant therapy"			
**Concern that the included participants and setting do not match the review question**	**CONCERN:**<br>*(low/ high/ unclear)*	Low	Low
Rationale of applicability rating:<br>Cohort study include patients with clear cell Renal Cell Carcinoma who underwent surgery.			

DOMAIN 2: Predictors			
**A. Risk of Bias**			
List and describe predictors included in the final model, e.g. definition and timing of assessment:<br><br>The predictors included in the model were: preoperative C-reactive protein, necrosis and T stage.<br><br>All the predictors were measured before or after the treatment			
		Dev	Val
2.1 Were predictors defined and assessed in a similar way for all participants?		PY	PY
2.2 Were predictor assessments made without knowledge of outcome data?		PY	PY
2.3 Are all predictors available at the time the model is intended to be used?		PY	PY
**Risk of bias introduced by predictors or their assessment**	**RISK:**<br>*(low/ high/ unclear)*	Low	Low
Rationale of bias rating:<br>*The blinding of measurement is unknown but all the predictors are objective.*			
**B. Applicability**			
Concern that the definition, assessment or timing of predictors in the model do not match the review question	**CONCERN:**<br>*(low/ high/ unclear)*	Low	Low
Rationale of applicability rating:<br>*No major issues identified.*			

DOMAIN 3: Outcome			
**A. Risk of Bias**			
Describe the outcome, how it was defined and determined, and the time interval between predictor assessment and outcome determination:<br>The outcome was cancer specific survival at 1, 3 and 5 years			
		Dev	Val
3.1 Was the outcome determined appropriately?		PY	PY
3.2 Was a pre-specified or standard outcome definition used?		Y	Y
3.3 Were predictors excluded from the outcome definition?		PY	PY
3.4 Was the outcome defined and determined in a similar way for all participants?		PY	PY
3.5 Was the outcome determined without knowledge of predictor information?		Y	Y
3.6 Was the time interval between predictor assessment and outcome determination appropriate?		Y	Y
**Risk of bias introduced by the outcome or its determination**	**RISK:**<br>*(low/ high/ unclear)*	Low	Low
Rationale of bias rating:<br>*No major issues identified*			
**B. Applicability**			
At what time point was the outcome determined:<br>1,3 and 5 years<br><br>*If a composite outcome was used, describe the relative frequency/distribution of each contributing outcome:*<br>*N/A*			
**Concern that the outcome, its definition, timing or determination do not match the review question**	**CONCERN:**<br>*(low/ high/ unclear)*	Low	Low
Rationale of applicability rating:<br>*The outcome of the primary study matches the outcome of interest of the review*			

DOMAIN 4: Analysis		
**Risk of Bias**		
Describe numbers of participants, number of candidate predictors, outcome events and events per candidate predictor:<br>"A total of 2284 patients were enrolled in the study, with 1599 (70%) and 685 (30%) randomly assigned to the training and validation cohorts, respectively"<br><br>The total number of predictors is 17 (table 2)<br>The number of events is unknown<br>EPV= unknown		
Describe how the model was developed (for example in regards to modelling technique (e.g. survival or logistic modelling), predictor selection, and risk group definition):<br><br>"The patients were randomly divided into training and validation cohorts at a 7:3 ratio. Nomogram models for OS, CSS,and RFS were developed using univariable and multivariable Cox regression analysis in the training cohort. Continuous variables were categorized based on established thresholds for age (40 and 65 years), BMI (18 and 24 kg/m2), tumor diameter (7 and 10 cm), and preoperative CRP (cutoff values of 5 and 50 mg/L)."		
Describe whether and how the model was validated, either internally (e.g. bootstrapping, cross validation, random split sample) or externally (e.g. temporal validation, geographical validation, different setting, different type of participants):<br><br>"An artificial intelligence (AI) model was developed for comparison, providing predictions for all-cause mortality, cancer-specific mortality, and recurrence at 1, 3, and 5 years. The AI models were compared with nomograms and CRP alone using AUC values"		
Describe the performance measures of the model, e.g. (re)calibration, discrimination, (re)classification, net benefit, and whether they were adjusted for optimism:<br><br>"In the validation cohort, nomogram performance was assessed through the C-index, calibration,and area under the receiver operating characteristic curve (AUC)."		
Describe any participants who were excluded from the analysis:<br>"To ensure at least one year of follow-up, we excluded patients who were diagnosed after December 31, 2015"<br>"The following exclusion criteria were set: (1) cases with distant or lymphnode metastasis, (2) recurrent cases at initial diagnosis, and (3) unobtainable or insufficient followup time (< 1 month)."		
Describe missing data on predictors and outcomes as well as methods used for missing data:<br>"Unknown"		
	Dev	Val
4.1 Were there a reasonable number of participants with the outcome?	PY	PN
4.2 Were continuous and categorical predictors handled appropriately?	N	N
4.3 Were all enrolled participants included in the analysis?	Y	Y
4.4 Were participants with missing data handled appropriately?	N	N
4.5 Was selection of predictors based on univariable analysis avoided?	N	
4.6 Were complexities in the data (e.g. censoring, competing risks, sampling of controls) accounted for appropriately?	Y	Y

4.7 Were relevant model performance measures evaluated appropriately?		Y	Y
4.8 Were model overfitting and optimism in model performance accounted for?		Y	
4.9 Do predictors and their assigned weights in the final model correspond to the results from multivariable analysis?		PY	
**Risk of bias introduced by the analysis**	**RISK:** *(low/ high/ unclear)*	High	High
Rationale of bias rating: *They number of patients with the outcome is unknown. They did categorizations. They don't report how handled missing data. They selected the predictors based on univariable. They didn't use competing risks.*			

Step 4: Overall assessment

Use the following tables to reach overall judgements about risk of bias and concerns regarding applicability of the prediction model evaluation (development and/or validation) across all assessed domains.
Complete for each evaluation of a distinct model.

**Reaching an overall judgement about risk of bias of the prediction model evaluation**	
**Low risk of bias**	If all domains were rated low risk of bias.<br>If a prediction model was developed without any external validation, and it was rated as low risk of bias for all domains, consider downgrading to **high risk of bias**. Such a model can only be considered as low risk of bias, if the development was based on a very large data set and included some form of internal validation.
**High risk of bias**	If at least one domain is judged to be at **high risk of bias**.
**Unclear risk of bias**	If an unclear risk of bias was noted in at least one domain and it was low risk for all other domains.

**Reaching an overall judgement about applicability of the prediction model evaluation**	
**Low concerns regarding applicability**	If low concerns regarding applicability for all domains, the prediction model evaluation is judged to have **low concerns regarding applicability**.
**High concerns regarding applicability**	If high concerns regarding applicability for at least one domain, the prediction model evaluation is judged to have **high concerns regarding applicability**.
**Unclear concerns regarding applicability**	If unclear concerns (but no "high concern") regarding applicability for at least one domain, the prediction model evaluation is judged to have **unclear concerns regarding applicability** overall.

**Overall judgement about risk of bias and applicability of the prediction model evaluation**		
**Overall judgement of risk of bias**	**RISK:**<br>*(low/ high/ unclear)*	Low
Summary of sources of potential bias:<br>No major issues		
**Overall judgement of applicability**	**CONCERN:**<br>*(low/ high/ unclear)*	High
Summary of applicability concerns:<br>Analysis domain shows major issues		

PROBAST

(Prediction model study Risk Of Bias Assessment Tool)

Published in Annals of Internal Medicine (freely available):

1. PROBAST: A Tool to Assess the Risk of Bias and Applicability of Prediction Model Studies
2. PROBAST: A Tool to Assess Risk of Bias and Applicability of Prediction Model Studies: Explanation and Elaboration

What does PROBAST assess?

PROBAST assesses both the *risk of bias* and *concerns regarding applicability* of a study that evaluates (develops, validates or updates) a multivariable diagnostic or prognostic prediction model. It is designed to assess primary studies included in a systematic review.

Bias occurs if systematic flaws or limitations in the design, conduct or analysis of a primary study distort the results. For the purpose of prediction modelling studies, we have defined *risk of bias* to occur when shortcomings in the study design, conduct or analysis lead to systematically distorted estimates of a model's predictive performance or to an inadequate model to address the research question. Model predictive performance is typically evaluated using calibration, discrimination and sometimes classification measures, and these are likely inaccurately estimated in studies with high risk of bias. *Applicability* refers to the extent to which the prediction model from the primary study matches your systematic review question, for example in terms of the participants, predictors or outcome of interest.

A primary study may include the development and/or validation or update of more than one prediction model. A PROBAST assessment should be completed for each distinct model that is developed, validated or updated (extended) for making individualised predictions. Where a publication assesses multiple prediction models, only complete a PROBAST assessment for those models that meet the inclusion criteria for your systematic review. Please note that subsequent use of the term "model" includes derivatives of models, such as simplified risk scores, nomograms, or recalibrations of models.

PROBAST is not designed for all multivariable diagnostic or prognostic studies. For example, studies using multivariable models to identify predictors associated with an outcome but not attempting to develop a model for making individualised predictions are not covered by PROBAST.

PROBAST includes four steps.

Step	Task	When to complete
**1**	Specify your systematic review question(s)	Once per systematic review
**2**	Classify the type of prediction model evaluation	Once for each model of interest in each publication being assessed, for each relevant outcome
**3**	Assess risk of bias and applicability	Once for each development and validation of each distinct prediction model in a publication
**4**	Overall judgment	Once for each development and validation of each distinct prediction model in a publication

If this is your first time using PROBAST, we strongly recommend reading the detailed explanation and elaboration (E&E, see link above) paper and to check the examples on www.probast.org

Step 1: Specify your systematic review question

State your systematic review question to facilitate the assessment of the applicability of the evaluated models to your question. *The following table should be completed once per systematic review.*

Criteria	Specify your systematic review question
Intended use of model:	*To predict cancer specific mortality in patients with renal cancer treated with partial or total nephrectomy regardless of TNM stage*
***Participants** including selection criteria and setting:*	*Patients with renal cancer treated with partial or total nephrectomy regardless of TNM stage*
***Predictors** (used in prediction modelling), including types of predictors (e.g. history, clinical examination, biochemical markers, imaging tests), time of measurement, specific measurement issues (e.g., any requirements/ prohibitions for specialized equipment):*	*Predictors used in clinical practice measured when a nephrectomy for renal cancer is indicated*
Outcome to be predicted:	*Cancer specific mortality*

Step 2: Classify the type of prediction model evaluation

Use the following table to classify the evaluation as model development, model validation or model update, or combination. Different signalling questions apply for different types of prediction model evaluation. If the evaluation does not fit one of these classifications then PROBAST should not be used.

**Classify the evaluation based on its aim**			
**Type of prediction study**	**PROBAST boxes to complete**	**Tick as appropriate**	**Definition for type of prediction model study**
Development only	Development	✓	Prediction model development without external validation. These studies may include internal validation methods, such as bootstrapping and cross-validation techniques.
Development and validation	Development and validation	✗	Prediction model development combined with external validation in other participants in the same article.
Validation only	Validation	✗	External validation of existing (previously developed) model in other participants.

This table should be completed once for each publication being assessed and for each relevant outcome in your review.

**Publication reference**	Jinliang Ni, Xiaoxiang Yao, Wei Song, Heng Zhang, Houliang Zhang, Yidi Wang, Yifan Zhang, Guangchun Wang, Keyi Wang, Weipu Mao, Bo Peng. Prognostic value of preoperative combined neutrophil, monocyte, and lymphocyte scores in patients with rena cell carcinoma undergoing laparoscopic nephrectomy: A retrospective study. Cancer Medicine. 2024;13:e7214.
**Models of interest**	Nomogram
**Outcome of interest**	*Cancer specific mortality*

Step 3: Assess risk of bias and applicability

PROBAST is structured as four key domains. Each domain is judged for risk of bias (low, high or unclear) and includes signalling questions to help make judgements. Signalling questions are rated as yes (Y), probably yes (PY), probably no (PN), no (N) or no information (NI). All signalling questions are phrased so that “yes” indicates absence of bias. Any signalling question rated as “no” or “probably no” flags the potential for bias; you will need to use your judgement to determine whether the domain should be rated as “high”, “low” or “unclear” risk of bias. The guidance document contains further instructions and examples on rating signalling questions and risk of bias for each domain.

The first three domains are also rated for concerns regarding applicability (low/ high/ unclear) to your review question defined above.

Complete all domains separately for each evaluation of a distinct model. Shaded boxes indicate where signalling questions do not apply and should not be answered.

<table>
<tr><td colspan="4">DOMAIN 1: Participants</td></tr>
<tr><td colspan="4">A. Risk of Bias</td></tr>
<tr><td colspan="4">Describe the sources of data and criteria for participant selection:

“Altogether, at Zhongda Hospital of Southeast University, Shanghai Shidong Hospital, and the Shanghai Tenth People's Hospital of Tongji University from January 2014 to December 2019, 590 patients with RCC who underwent partial or radical nephrectomy were collected in the present multi-institutional”
“The criteria for inclusion of patients were determined as follows: (1) patients diagnosed with RCC by histopathology;(2) patients treated with laparoscopic nephrectomy; (3) patients with complete medical records and no missing follow-up data”</td></tr>
<tr><td colspan="2"></td><td>Dev</td><td>Val</td></tr>
<tr><td colspan="2">1.1 Were appropriate data sources used, e.g. cohort, RCT or nested case-control study data?</td><td>Y</td><td>N/A</td></tr>
<tr><td colspan="2">1.2 Were all inclusions and exclusions of participants appropriate?</td><td>N</td><td>N/A</td></tr>
<tr><td>Risk of bias introduced by selection of participants</td><td>RISK:
(low/ high/ unclear)</td><td>High</td><td>N/A</td></tr>
<tr><td colspan="4">Rationale of bias rating:
Cohort study only patients treated with laparoscopic surgery.</td></tr>
<tr><td colspan="4">B. Applicability</td></tr>
<tr><td colspan="4">Describe included participants, setting and dates:

“Altogether, at Zhongda Hospital of Southeast University, Shanghai Shidong Hospital, and the Shanghai Tenth People's Hospital of Tongji University from January 2014 to December 2019, 590 patients with RCC who underwent partial or radical nephrectomy were collected in the present multi-institutional”
“The criteria for inclusion of patients were determined as follows: (1) patients diagnosed with RCC by histopathology;(2) patients treated with laparoscopic nephrectomy; (3) patients with complete medical records and no missing follow-up data”</td></tr>
<tr><td>Concern that the included participants and setting do not match the review question</td><td>CONCERN:
(low/ high/ unclear)</td><td>High</td><td>N/A</td></tr>
<tr><td colspan="4">Rationale of applicability rating:
Cohort study only patients treated with laparoscopic surgery.</td></tr>
</table>

DOMAIN 2: Predictors			
**A. Risk of Bias**			
List and describe predictors included in the final model, e.g. definition and timing of assessment:<br><br>The predictors included in the model were: M stage, AJCC stage and PBIS score.<br><br>All the predictors were measured at diagnosis or after the treatment			
		Dev	Val
2.1 Were predictors defined and assessed in a similar way for all participants?		PY	N/A
2.2 Were predictor assessments made without knowledge of outcome data?		PY	N/A
2.3 Are all predictors available at the time the model is intended to be used?		PY	N/A
**Risk of bias introduced by predictors or their assessment**	**RISK:**<br>*(low/ high/ unclear)*	Low	N/A
Rationale of bias rating:<br>*The blinding of measurement is unknown but all the predictors are objective.*			
**B. Applicability**			
Concern that the definition, assessment or timing of predictors in the model do not match the review question	**CONCERN:**<br>*(low/ high/ unclear)*	Low	N/A
Rationale of applicability rating:<br>*No major issues identified.*			

DOMAIN 3: Outcome			
**A. Risk of Bias**			
Describe the outcome, how it was defined and determined, and the time interval between predictor assessment and outcome determination:<br>The outcome was cancer specific survival at 3 and 5 years			
		Dev	Val
3.1 Was the outcome determined appropriately?		PY	N/A
3.2 Was a pre-specified or standard outcome definition used?		Y	N/A
3.3 Were predictors excluded from the outcome definition?		Y	N/A
3.4 Was the outcome defined and determined in a similar way for all participants?		Y	N/A
3.5 Was the outcome determined without knowledge of predictor information?		PY	N/A
3.6 Was the time interval between predictor assessment and outcome determination appropriate?		Y	N/A
**Risk of bias introduced by the outcome or its determination**	**RISK:**<br>*(low/ high/ unclear)*	Low	N/A
Rationale of bias rating:<br>*No major issues identified*			
**B. Applicability**			
At what time point was the outcome determined:<br>3 and 5 years<br><br>*If a composite outcome was used, describe the relative frequency/distribution of each contributing outcome:*<br>*N/A*			
**Concern that the outcome, its definition, timing or determination do not match the review question**	**CONCERN:**<br>*(low/ high/ unclear)*	Low	N/A
Rationale of applicability rating:<br>*The outcome of the primary study matches the outcome of interest of the review*			

DOMAIN 4: Analysis			
**Risk of Bias**			
Describe numbers of participants, number of candidate predictors, outcome events and events per candidate predictor:<br><br>The total number of predictors is 21.<br>The number of events is 36<br>EPV = 36/21 = 1.71			
Describe how the model was developed (for example in regards to modelling technique (e.g. survival or logistic modelling), predictor selection, and risk group definition):<br><br>*"Univariate and multivariate Cox proportional hazards regression models were performed to assess independent risk factors for OS and CSS. The hazard ratios (HRs) calculated from the Cox regression models were taken as relative risks with95% confidence interval (CI)."*			
Describe whether and how the model was validated, either internally (e.g. bootstrapping, cross validation, random split sample) or externally (e.g. temporal validation, geographical validation, different setting, different type of participants):<br><br>"Internal and external verification of the nomograms were evaluated with bootstrap resampling and 10-fold cross-validation to plot the calibration curves"			
Describe the performance measures of the model, e.g. (re)calibration, discrimination, (re)classification, net benefit, and whether they were adjusted for optimism:<br><br>*"Decision curve analysis (DCA), Harrell's concordance index (C index) and receiver operating characteristic (ROC) curves were conducted to assess potential clinical discernment and efficacy of the nomogram."*			
Describe any participants who were excluded from the analysis:<br>"The exclusion criteria are established as follows: (1) patients with a combination of other malignancies that seriously affect survival; (2) patients received other anti-cancer treatments prior to laparoscopic nephrectomy; (3) patients with pre-operative blood or other autoimmune diseases"			
Describe missing data on predictors and outcomes as well as methods used for missing data:<br>*Patients with incomplete medical records or missing data were excluded.*			
		Dev	Val
4.1 Were there a reasonable number of participants with the outcome?		N	N/A
4.2 Were continuous and categorical predictors handled appropriately?		N	N/A
4.3 Were all enrolled participants included in the analysis?		Y	N/A
4.4 Were participants with missing data handled appropriately?		N	N/A
4.5 Was selection of predictors based on univariable analysis avoided?		N	
4.6 Were complexities in the data (e.g. censoring, competing risks, sampling of controls) accounted for appropriately?		N	N/A
4.7 Were relevant model performance measures evaluated appropriately?		Y	N/A
4.8 Were model overfitting and optimism in model performance accounted for?		Y	
4.9 Do predictors and their assigned weights in the final model correspond to the results from multivariable analysis?		Y	
**Risk of bias introduced by the analysis**	**RISK:**<br>*(low/ high/ unclear)*	High	N/A

Rationale of bias rating:
They didn´t have enough patients with the outcome. They did categorizations. They excluded patients with missing data. They selected the predictors based on univariate analysis. They didn’t use competing risks.

Step 4: Overall assessment

Use the following tables to reach overall judgements about risk of bias and concerns regarding applicability of the prediction model evaluation (development and/or validation) across all assessed domains.
Complete for each evaluation of a distinct model.

**Reaching an overall judgement about risk of bias of the prediction model evaluation**	
**Low risk of bias**	If all domains were rated low risk of bias.<br>If a prediction model was developed without any external validation, and it was rated as low risk of bias for all domains, consider downgrading to **high risk of bias**. Such a model can only be considered as low risk of bias, if the development was based on a very large data set and included some form of internal validation.
**High risk of bias**	If at least one domain is judged to be at **high risk of bias**.
**Unclear risk of bias**	If an unclear risk of bias was noted in at least one domain and it was low risk for all other domains.

**Reaching an overall judgement about applicability of the prediction model evaluation**	
**Low concerns regarding applicability**	If low concerns regarding applicability for all domains, the prediction model evaluation is judged to have **low concerns regarding applicability**.
**High concerns regarding applicability**	If high concerns regarding applicability for at least one domain, the prediction model evaluation is judged to have **high concerns regarding applicability**.
**Unclear concerns regarding applicability**	If unclear concerns (but no "high concern") regarding applicability for at least one domain, the prediction model evaluation is judged to have **unclear concerns regarding applicability** overall.

**Overall judgement about risk of bias and applicability of the prediction model evaluation**		
**Overall judgement of risk of bias**	**RISK:**<br>*(low/ high/ unclear)*	High
Summary of sources of potential bias:<br>Analysis and participants domains show several major issues		
**Overall judgement of applicability**	**CONCERN:**<br>*(low/ high/ unclear)*	High
Summary of applicability concerns:<br><br>Participant domain shows several major issues		

PROBAST

(Prediction model study Risk Of Bias Assessment Tool)

Published in Annals of Internal Medicine (freely available):

1. PROBAST: A Tool to Assess the Risk of Bias and Applicability of Prediction Model Studies
2. PROBAST: A Tool to Assess Risk of Bias and Applicability of Prediction Model Studies: Explanation and Elaboration

What does PROBAST assess?

PROBAST assesses both the *risk of bias* and *concerns regarding applicability* of a study that evaluates (develops, validates or updates) a multivariable diagnostic or prognostic prediction model. It is designed to assess primary studies included in a systematic review.

Bias occurs if systematic flaws or limitations in the design, conduct or analysis of a primary study distort the results. For the purpose of prediction modelling studies, we have defined *risk of bias* to occur when shortcomings in the study design, conduct or analysis lead to systematically distorted estimates of a model's predictive performance or to an inadequate model to address the research question. Model predictive performance is typically evaluated using calibration, discrimination and sometimes classification measures, and these are likely inaccurately estimated in studies with high risk of bias. *Applicability* refers to the extent to which the prediction model from the primary study matches your systematic review question, for example in terms of the participants, predictors or outcome of interest.

A primary study may include the development and/or validation or update of more than one prediction model. A PROBAST assessment should be completed for each distinct model that is developed, validated or updated (extended) for making individualised predictions. Where a publication assesses multiple prediction models, only complete a PROBAST assessment for those models that meet the inclusion criteria for your systematic review. Please note that subsequent use of the term "model" includes derivatives of models, such as simplified risk scores, nomograms, or recalibrations of models.

PROBAST is not designed for all multivariable diagnostic or prognostic studies. For example, studies using multivariable models to identify predictors associated with an outcome but not attempting to develop a model for making individualised predictions are not covered by PROBAST.

PROBAST includes four steps.

Step	Task	When to complete
**1**	Specify your systematic review question(s)	Once per systematic review
**2**	Classify the type of prediction model evaluation	Once for each model of interest in each publication being assessed, for each relevant outcome
**3**	Assess risk of bias and applicability	Once for each development and validation of each distinct prediction model in a publication
**4**	Overall judgment	Once for each development and validation of each distinct prediction model in a publication

If this is your first time using PROBAST, we strongly recommend reading the detailed explanation and elaboration (E&E, see link above) paper and to check the examples on www.probast.org

Step 1: Specify your systematic review question

State your systematic review question to facilitate the assessment of the applicability of the evaluated models to your question. *The following table should be completed once per systematic review.*

Criteria	Specify your systematic review question
Intended use of model:	*To predict cancer specific mortality in patients with renal cancer treated with partial or total nephrectomy regardless of TNM stage*
***Participants** including selection criteria and setting:*	*Patients with renal cancer treated with partial or total nephrectomy regardless of TNM stage*
***Predictors** (used in prediction modelling), including types of predictors (e.g. history, clinical examination, biochemical markers, imaging tests), time of measurement, specific measurement issues (e.g., any requirements/ prohibitions for specialized equipment):*	*Predictors used in clinical practice measured when a nephrectomy for renal cancer is indicated*
Outcome to be predicted:	*Cancer specific mortality*

Step 2: Classify the type of prediction model evaluation

Use the following table to classify the evaluation as model development, model validation or model update, or combination. Different signalling questions apply for different types of prediction model evaluation. If the evaluation does not fit one of these classifications then PROBAST should not be used.

Classify the evaluation based on its aim			
**Type of prediction study**	**PROBAST boxes to complete**	**Tick as appropriate**	**Definition for type of prediction model study**
Development only	Development	✗	Prediction model development without external validation. These studies may include internal validation methods, such as bootstrapping and cross-validation techniques.
Development and validation	Development and validation	✓	Prediction model development combined with external validation in other participants in the same article.
Validation only	Validation	✗	External validation of existing (previously developed) model in other participants.

This table should be completed once for each publication being assessed and for each relevant outcome in your review.

**Publication reference**	Qingxiang Guo, Sai Li, Jiawei Zhu, Zewei Wang, Zhen Li, Junqi Wang, Rumin Wen, Hailong Li. Development and validation of prognostic nomograms for adult with papillary renal cell carcinoma: A retrospective study. Clinics 79. 2024: 100374
**Models of interest**	Nomogram and Risk Score
**Outcome of interest**	*Cancer specific mortality*

Step 3: Assess risk of bias and applicability

PROBAST is structured as four key domains. Each domain is judged for risk of bias (low, high or unclear) and includes signalling questions to help make judgements. Signalling questions are rated as yes (Y), probably yes (PY), probably no (PN), no (N) or no information (NI). All signalling questions are phrased so that "yes" indicates absence of bias. Any signalling question rated as "no" or "probably no" flags the potential for bias; you will need to use your judgement to determine whether the domain should be rated as "high", "low" or "unclear" risk of bias. The guidance document contains further instructions and examples on rating signalling questions and risk of bias for each domain.

The first three domains are also rated for concerns regarding applicability (low/ high/ unclear) to your review question defined above.

Complete all domains separately for each evaluation of a distinct model. Shaded boxes indicate where signalling questions do not apply and should not be answered.

<table>
<tr><td colspan="4">DOMAIN 1: Participants</td></tr>
<tr><td colspan="4">A. Risk of Bias</td></tr>
<tr><td colspan="4">Describe the sources of data and criteria for participant selection:

“The authors used the Surveillance, Epidemiology, and End Results (SEER)* statsoftwaretoidentify1074suitableadultswhowerediag-nosedwithpRCCbetween2004and2015fromtheSEERdatabase, which contains clinicopathological and distinct prognostic data. The inclusion criteria were as follows: (i)Papillary renal cell carcinoma as the only or first primary tumor that was confirmed by histology, (ii)Patientw ith surgically removed malignancies, and(iii) Active follow-up to ensure reliable patient status.”</td></tr>
<tr><td colspan="2"></td><td>Dev</td><td>Val</td></tr>
<tr><td colspan="2">1.1 Were appropriate data sources used, e.g. cohort, RCT or nested case-control study data?</td><td>Y</td><td>Y</td></tr>
<tr><td colspan="2">1.2 Were all inclusions and exclusions of participants appropriate?</td><td>N</td><td>N</td></tr>
<tr><td>Risk of bias introduced by selection of participants</td><td>RISK:
(low/ high/ unclear)</td><td>High</td><td>High</td></tr>
<tr><td colspan="4">Rationale of bias rating:
Cohort study included patients without nephrectomy
The study only included patients with papillary RCC.</td></tr>
<tr><td colspan="4">B. Applicability</td></tr>
<tr><td colspan="4">Describe included participants, setting and dates:
“A total of 4,859 patients with pRCC diagnosed between 2010 and 2014 were included in this study (selection flow chart was in Figure 1. After randomly grouping, 3,403 patients were included in the training cohort and the remaining 1,456 patients were in the validation cohort”

“The authors used the Surveillance, Epidemiology, and End Results (SEER)* statsoftwaretoidentify1074suitableadultswhowerediag-nosedwithpRCCbetween2004and2015fromtheSEERdatabase, which contains clinicopathological and distinct prognostic data. The inclusion criteria were as follows: (i)Papillary renal cell carcinoma as the only or first primary tumor that was confirmed by histology, (ii)Patientw ith surgically removed malignancies, and(iii) Active follow-up to ensure reliable patient status.”</td></tr>
<tr><td>Concern that the included participants and setting do not match the review question</td><td>CONCERN:
(low/ high/ unclear)</td><td>High</td><td>High</td></tr>
<tr><td colspan="4">The study only included patients with papillary RCC. They used treatment (chemotherapy and radiotherapy) as a predictor</td></tr>
</table>

DOMAIN 2: Predictors			
**A. Risk of Bias**			
List and describe predictors included in the final model, e.g. definition and timing of assessment:<br><br>The predictors included in the model were: age, TNM, AJCC stage and radiotherapy.<br><br>All the predictors were measured after treatment			
		Dev	Val
2.1 Were predictors defined and assessed in a similar way for all participants?		PY	PY
2.2 Were predictor assessments made without knowledge of outcome data?		PY	PY
2.3 Are all predictors available at the time the model is intended to be used?		PY	PY
**Risk of bias introduced by predictors or their assessment**	**RISK:**<br>*(low/ high/ unclear)*	Low	Low
Rationale of bias rating:<br>*The blinding of measurement is unknown but all the predictors are objective.*			
**B. Applicability**			
Concern that the definition, assessment or timing of predictors in the model do not match the review question	**CONCERN:**<br>*(low/ high/ unclear)*	Low	Low
Rationale of applicability rating:<br>*No major issues identified.*			

DOMAIN 3: Outcome			
**A. Risk of Bias**			
Describe the outcome, how it was defined and determined, and the time interval between predictor assessment and outcome determination:<br>The outcome was cancer specific survival at 3, 5 and 8 years			
		Dev	Val
3.1 Was the outcome determined appropriately?		PY	PY
3.2 Was a pre-specified or standard outcome definition used?		Y	Y
3.3 Were predictors excluded from the outcome definition?		PY	PY
3.4 Was the outcome defined and determined in a similar way for all participants?		PY	PY
3.5 Was the outcome determined without knowledge of predictor information?		PY	PY
3.6 Was the time interval between predictor assessment and outcome determination appropriate?		Y	Y
**Risk of bias introduced by the outcome or its determination**	**RISK:**<br>*(low/ high/ unclear)*	Low	Low
Rationale of bias rating:<br>*No major issues identified*			
**B. Applicability**			
At what time point was the outcome determined:<br>3,5 and 8 -year CSS<br><br>*If a composite outcome was used, describe the relative frequency/distribution of each contributing outcome:*<br>*N/A*			
**Concern that the outcome, its definition, timing or determination do not match the review question**	**CONCERN:**<br>*(low/ high/ unclear)*	Low	Low
Rationale of applicability rating:<br>*The outcome of the primary study matches the outcome of interest of the review*			

DOMAIN 4: Analysis		
**Risk of Bias**		
Describe numbers of participants, number of candidate predictors, outcome events and events per candidate predictor:<br>*N= 752 (E= unknown) to develop the model, 322 (E= unknown) for internal validation and 107 (E= unknown) for external validation. (E= unknown)*<br><br>*A total of 31 predictors were included (Table 3)*<br>*The number of events is unknown*<br><br>*EPV= unknown*		
Describe how the model was developed (for example in regards to modelling technique (e.g. survival or logistic modelling), predictor selection, and risk group definition):<br><br>*"In the training cohort, prognostic factors were screened out using univariate Cox regression, and independent risk factors were examined using multivariate Cox regression."*		
Describe whether and how the model was validated, either internally (e.g. bootstrapping, cross validation, random split sample) or externally (e.g. temporal validation, geographical validation, different setting, different type of participants):<br><br>*"Internal and external validations were used to test the performance of the nomogram. Externally validated patient data were gathered from the Affiliated Hospital of Xuzhou Medical University."*		
Describe the performance measures of the model, e.g. (re)calibration, discrimination, (re)classification, net benefit, and whether they were adjusted for optimism:<br><br>*"The nomogram was then verified using a series of verification methods, such as the C-index, the Receiver Operating Characteristic (ROC) curve, and a calibration curve."*		
Describe any participants who were excluded from the analysis:<br>*"The exclusion criteria were as follows: (i) Patients who were older than 85 years and younger than 18 years; (ii) Patients with insufficient follow-up data, such as missing information on ethnicity, surgery type, lymph node status, tumor stage, pathological grade of the tumor, and adjuvant therapy; and (iii) Following initial diagnosis, the patient died within 1 month.*		
Describe missing data on predictors and outcomes as well as methods used for missing data:<br>*Patients with insufficient follow-up data, such as missing information on ethnicity, surgery type, lymph node status, tumor stage, pathological grade of the tumor, and adjuvant therapy were excluded.*		
	Dev	Val
4.1 Were there a reasonable number of participants with the outcome?	NI	PN
4.2 Were continuous and categorical predictors handled appropriately?	N	N
4.3 Were all enrolled participants included in the analysis?	Y	Y
4.4 Were participants with missing data handled appropriately?	N	N
4.5 Was selection of predictors based on univariable analysis avoided?	N	
4.6 Were complexities in the data (e.g. censoring, competing risks, sampling of controls) accounted for appropriately?	N	N
4.7 Were relevant model performance measures evaluated appropriately?	Y	Y

4.8 Were model overfitting and optimism in model performance accounted for?		N	
4.9 Do predictors and their assigned weights in the final model correspond to the results from multivariable analysis?		PY	
**Risk of bias introduced by the analysis**	**RISK:** *(low/ high/ unclear)*	High	High
Rationale of bias rating: *They didn´t say the number of events. They did categorizations. They excluded patients with missing data. They selected the predictors based on univariable. They didn't use competing risks. They didn´t do an overfitting and optimism in model performance.*			

Step 4: Overall assessment

Use the following tables to reach overall judgements about risk of bias and concerns regarding applicability of the prediction model evaluation (development and/or validation) across all assessed domains.
Complete for each evaluation of a distinct model.

Reaching an overall judgement about risk of bias of the prediction model evaluation	
**Low risk of bias**	If all domains were rated low risk of bias.<br>If a prediction model was developed without any external validation, and it was rated as low risk of bias for all domains, consider downgrading to **high risk of bias**. Such a model can only be considered as low risk of bias, if the development was based on a very large data set and included some form of internal validation.
**High risk of bias**	If at least one domain is judged to be at **high risk of bias**.
**Unclear risk of bias**	If an unclear risk of bias was noted in at least one domain and it was low risk for all other domains.

Reaching an overall judgement about applicability of the prediction model evaluation	
**Low concerns regarding applicability**	If low concerns regarding applicability for all domains, the prediction model evaluation is judged to have **low concerns regarding applicability**.
**High concerns regarding applicability**	If high concerns regarding applicability for at least one domain, the prediction model evaluation is judged to have **high concerns regarding applicability**.
**Unclear concerns regarding applicability**	If unclear concerns (but no "high concern") regarding applicability for at least one domain, the prediction model evaluation is judged to have **unclear concerns regarding applicability** overall.

Overall judgement about risk of bias and applicability of the prediction model evaluation		
**Overall judgement of risk of bias**	**RISK:**<br>*(low/ high/ unclear)*	High
Summary of sources of potential bias:<br>Analysis and participants domains show several major issues		
**Overall judgement of applicability**	**CONCERN:**<br>*(low/ high/ unclear)*	High
Summary of applicability concerns:<br><br>Participants domains shows several major issues		

PROBAST

(Prediction model study Risk Of Bias Assessment Tool)

Published in Annals of Internal Medicine (freely available):

1. PROBAST: A Tool to Assess the Risk of Bias and Applicability of Prediction Model Studies
2. PROBAST: A Tool to Assess Risk of Bias and Applicability of Prediction Model Studies: Explanation and Elaboration

What does PROBAST assess?

PROBAST assesses both the *risk of bias* and *concerns regarding applicability* of a study that evaluates (develops, validates or updates) a multivariable diagnostic or prognostic prediction model. It is designed to assess primary studies included in a systematic review.

Bias occurs if systematic flaws or limitations in the design, conduct or analysis of a primary study distort the results. For the purpose of prediction modelling studies, we have defined *risk of bias* to occur when shortcomings in the study design, conduct or analysis lead to systematically distorted estimates of a model's predictive performance or to an inadequate model to address the research question. Model predictive performance is typically evaluated using calibration, discrimination and sometimes classification measures, and these are likely inaccurately estimated in studies with high risk of bias. *Applicability* refers to the extent to which the prediction model from the primary study matches your systematic review question, for example in terms of the participants, predictors or outcome of interest.

A primary study may include the development and/or validation or update of more than one prediction model. A PROBAST assessment should be completed for each distinct model that is developed, validated or updated (extended) for making individualised predictions. Where a publication assesses multiple prediction models, only complete a PROBAST assessment for those models that meet the inclusion criteria for your systematic review. Please note that subsequent use of the term "model" includes derivatives of models, such as simplified risk scores, nomograms, or recalibrations of models.

PROBAST is not designed for all multivariable diagnostic or prognostic studies. For example, studies using multivariable models to identify predictors associated with an outcome but not attempting to develop a model for making individualised predictions are not covered by PROBAST.

PROBAST includes four steps.

**Step**	**Task**	**When to complete**
**1**	Specify your systematic review question(s)	Once per systematic review
**2**	Classify the type of prediction model evaluation	Once for each model of interest in each publication being assessed, for each relevant outcome
**3**	Assess risk of bias and applicability	Once for each development and validation of each distinct prediction model in a publication
**4**	Overall judgment	Once for each development and validation of each distinct prediction model in a publication

If this is your first time using PROBAST, we strongly recommend reading the detailed explanation and elaboration (E&E, see link above) paper and to check the examples on www.probast.org

Step 1: Specify your systematic review question

State your systematic review question to facilitate the assessment of the applicability of the evaluated models to your question. *The following table should be completed once per systematic review.*

Criteria	Specify your systematic review question
Intended use of model:	*To predict cancer specific mortality in patients with renal cancer treated with partial or total nephrectomy regardless of TNM stage*
***Participants** including selection criteria and setting:*	*Patients with renal cancer treated with partial or total nephrectomy regardless of TNM stage*
***Predictors** (used in prediction modelling), including types of predictors (e.g. history, clinical examination, biochemical markers, imaging tests), time of measurement, specific measurement issues (e.g., any requirements/ prohibitions for specialized equipment):*	*Predictors used in clinical practice measured when a nephrectomy for renal cancer is indicated*
Outcome to be predicted:	*Cancer specific mortality*

Step 2: Classify the type of prediction model evaluation

Use the following table to classify the evaluation as model development, model validation or model update, or combination. Different signalling questions apply for different types of prediction model evaluation. If the evaluation does not fit one of these classifications then PROBAST should not be used.

**Classify the evaluation based on its aim**			
**Type of prediction study**	**PROBAST boxes to complete**	**Tick as appropriate**	**Definition for type of prediction model study**
Development only	Development	✗	Prediction model development without external validation. These studies may include internal validation methods, such as bootstrapping and cross-validation techniques.
Development and validation	Development and validation	✓	Prediction model development combined with external validation in other participants in the same article.
Validation only	Validation	✗	External validation of existing (previously developed) model in other participants.

This table should be completed once for each publication being assessed and for each relevant outcome in your review.

**Publication reference**	Yan H, Wei X, Wu A, Sha Y, Li X, Qi F. Nomograms for predicting overall and cancer-specific survival in patients with papillary renal cell carcinoma: a population-based study using SEER database. Transl Androl Urol. 2020 Jun;9(3):1146-1158. doi: 10.21037/tau-19-807.
**Models of interest**	Nomogram
**Outcome of interest**	*Cancer specific mortality*

Step 3: Assess risk of bias and applicability

PROBAST is structured as four key domains. Each domain is judged for risk of bias (low, high or unclear) and includes signalling questions to help make judgements. Signalling questions are rated as yes (Y), probably yes (PY), probably no (PN), no (N) or no information (NI). All signalling questions are phrased so that "yes" indicates absence of bias. Any signalling question rated as "no" or "probably no" flags the potential for bias; you will need to use your judgement to determine whether the domain should be rated as "high", "low" or "unclear" risk of bias. The guidance document contains further instructions and examples on rating signalling questions and risk of bias for each domain.

The first three domains are also rated for concerns regarding applicability (low/ high/ unclear) to your review question defined above.

Complete all domains separately for each evaluation of a distinct model. Shaded boxes indicate where signalling questions do not apply and should not be answered.

DOMAIN 1: Participants			
**A. Risk of Bias**			
Describe the sources of data and criteria for participant selection:<br><br>*"A total of 4,859 patients with pRCC diagnosed between 2010 and 2014 were included in this study (selection flow chart was in Figure 1. After randomly grouping, 3,403 patients were included in the training cohort and the remaining 1,456 patients were in the validation cohort"*<br><br>*"The inclusion criteria of this study were as follows: (I) diagnosed as pRCC (International Classification of Diseases for Oncology: 8260/3) with positive histology, (II) year at diagnosis was from 2010 to 2014 to ensure a relatively long follow-up period, (III) complete data were available with active follow-up. Additionally, the exclusion criteria were as follows: (I) missing/unknown data in following variables: age, sex, race, American Joint Committee on Cancer (AJCC) 7th edition TNM stage, tumor laterality (bilateral tumors also been excluded), surgery, radiation, chemotherapy, follow-up time, insurance status, marital status, survival outcomes and so on, (II) pRCC was not the first primary malignancy, (III) type of reporting source was autopsy only or death certificate only"*			
		Dev	Val
1.1 Were appropriate data sources used, e.g. cohort, RCT or nested case-control study data?		Y	Y
1.2 Were all inclusions and exclusions of participants appropriate?		N	N
**Risk of bias introduced by selection of participants**	**RISK:**<br>*(low/ high/ unclear)*	High	High
Rationale of bias rating:<br>*Cohort study included patients without nephrectomy*<br>*The study only included patients with papillary RCC*			
**B. Applicability**			
Describe included participants, setting and dates:<br>*"A total of 4,859 patients with pRCC diagnosed between 2010 and 2014 were included in this study (selection flow chart was in Figure 1. After randomly grouping, 3,403 patients were included in the training cohort and the remaining 1,456 patients were in the validation cohort"*<br><br>*"The inclusion criteria of this study were as follows: (I) diagnosed as pRCC (International Classification of Diseases for Oncology: 8260/3) with positive histology, (II) year at diagnosis was from 2010 to 2014 to ensure a relatively long follow-up period, (III) complete data were available with active follow-up. Additionally, the exclusion criteria were as follows: (I) missing/unknown data in following variables: age, sex, race, American Joint Committee on Cancer (AJCC) 7th edition TNM stage, tumor laterality (bilateral tumors also been excluded), surgery, radiation, chemotherapy, follow-up time, insurance status, marital status, survival outcomes and so on, (II) pRCC was not the first primary malignancy, (III) type of reporting source was autopsy only or death certificate only"*			
**Concern that the included participants and setting do not match the review question**	**CONCERN:**<br>*(low/ high/ unclear)*	High	High
Rationale of applicability rating:<br>*Included patients without nephrectomy*<br>*The study only included patients with papillary RCC*			

DOMAIN 2: Predictors			
**A. Risk of Bias**			
List and describe predictors included in the final model, e.g. definition and timing of assessment:<br><br>The predictors included in the model were: age, TNM, surgery, lymph node removal and chemotherapy<br><br>All the predictors were measured after treatment			
		Dev	Val
2.1 Were predictors defined and assessed in a similar way for all participants?		PY	PY
2.2 Were predictor assessments made without knowledge of outcome data?		PY	PY
2.3 Are all predictors available at the time the model is intended to be used?		PY	PY
**Risk of bias introduced by predictors or their assessment**	**RISK:**<br>*(low/ high/ unclear)*	Low	Low
Rationale of bias rating:<br>*The blinding of measurement is unknown but all the predictors are objective.*			
**B. Applicability**			
Concern that the definition, assessment or timing of predictors in the model do not match the review question	**CONCERN:**<br>*(low/ high/ unclear)*	Low	Low
Rationale of applicability rating:<br>*No major issues identified.*			

DOMAIN 3: Outcome

A. Risk of Bias

Describe the outcome, how it was defined and determined, and the time interval between predictor assessment and outcome determination:

The outcome was cancer specific survival at 3 and 5 years

"Survival time was calculated from the date of diagnosis to the date of death from pRCC (defined as CSS) or any disease cause (defined as OS)"

		Dev	Val
3.1 Was the outcome determined appropriately?		PY	PY
3.2 Was a pre-specified or standard outcome definition used?		Y	Y
3.3 Were predictors excluded from the outcome definition?		PY	PY
3.4 Was the outcome defined and determined in a similar way for all participants?		PY	PY
3.5 Was the outcome determined without knowledge of predictor information?		PY	PY
3.6 Was the time interval between predictor assessment and outcome determination appropriate?		Y	Y
**Risk of bias introduced by the outcome or its determination**	**RISK:** *(low/ high/ unclear)*	Low	Low

Rationale of bias rating:

No major issues identified

B. Applicability

At what time point was the outcome determined:

3 and 5 years

If a composite outcome was used, describe the relative frequency/distribution of each contributing outcome:

N/A

**Concern that the outcome, its definition, timing or determination do not match the review question**	**CONCERN:** *(low/ high/ unclear)*	Low	Low

Rationale of applicability rating:

The outcome of the primary study matches the outcome of interest of the review

DOMAIN 4: Analysis
**Risk of Bias**
Describe numbers of participants, number of candidate predictors, outcome events and events per candidate predictor:<br>*"A total of 4,859 patients with pRCC diagnosed between 2010 and 2014 were included in this study (selection flow chart was in Figure 1. After randomly grouping, 3,403 patients were included in the training cohort and the remaining 1,456 patients were in the validation cohort"*<br><br>*A total of 19 predictors were included (Table 3)*<br>*The number of events is unknown*<br><br>*EPV= unknown*
Describe how the model was developed (for example in regards to modelling technique (e.g. survival or logistic modelling), predictor selection, and risk group definition):<br><br>*"Uni and multivariate Cox regression analyses were conducted to explore the prognostic factors which affect OS and CSS significantly. Additionally, hazard ratios (HRs) and corresponding 95% confidence intervals (CIs) of selected factors were calculated. According to the results of multivariate Cox regression analysis, predictive nomograms for 3- and 5-year CSS and OS were developed. In the training group, survival curves for different variables were produced by Kaplan-Meier (KM) analyses and were compared utilizing the log-rank test"*
Describe whether and how the model was validated, either internally (e.g. bootstrapping, cross validation, random split sample) or externally (e.g. temporal validation, geographical validation, different setting, different type of participants):<br><br>*"In order to develop the prognostic nomograms and undergo further external validation, all of the enrolled patients were divided into training group and validation group randomly at a ratio of 7:3 by using random-number generation method. Finally, chi-square test was utilized to make comparisons in basic characteristics between two groups"*
Describe the performance measures of the model, e.g. (re)calibration, discrimination, (re)classification, net benefit, and whether they were adjusted for optimism:<br><br>*"To assess the predictive ability and accuracy of the nomograms, discrimination and calibration of the nomograms were measured in two groups. The area under receiver operating characteristic (ROC) curve (defined as AUC) (13,14) and Harrell's concordance index (C-index) (15) were applied to assess the discrimination. The AUC and C-index range from 0.5 to 1.0, with 0.5 suggesting the total chance and 1.0 suggesting a perfect discrimination ability (16). Calibration curves were performed to identify the consistency between the observed survival and the predicted survival"*
Describe any participants who were excluded from the analysis:<br>*"Exclude patients with unknown/missing data:*<br>*1. Tumor laterality (bilateral tumors also been excluded): 49*<br>*2. 7th AJCC TNM stage: 4,834*<br>*3. Use of surgery: 1*<br>*4. Use of lymph node removal: 6*<br>*5. Cause of death: 17*

6. Race: 34, marital status: 292, insurance status: 40”			
Describe missing data on predictors and outcomes as well as methods used for missing data:<br>*“Additionally, the exclusion criteria were as follows: (I) missing/unknown data in following variables: age, sex, race, American Joint Committee on Cancer (AJCC) 7th edition TNM stage, tumor laterality (bilateral tumors also been excluded), surgery, radiation, chemotherapy, follow-up time, insurance status, marital status, survival outcomes and so on”*			
		Dev	Val
4.1 Were there a reasonable number of participants with the outcome?		NI	PY
4.2 Were continuous and categorical predictors handled appropriately?		N	N
4.3 Were all enrolled participants included in the analysis?		N	N
4.4 Were participants with missing data handled appropriately?		N	N
4.5 Was selection of predictors based on univariable analysis avoided?		N	
4.6 Were complexities in the data (e.g. censoring, competing risks, sampling of controls) accounted for appropriately?		N	N
4.7 Were relevant model performance measures evaluated appropriately?		Y	Y
4.8 Were model overfitting and optimism in model performance accounted for?		N	
4.9 Do predictors and their assigned weights in the final model correspond to the results from multivariable analysis?		PY	
**Risk of bias introduced by the analysis**	**RISK:**<br>*(low/ high/ unclear)*	High	High
Rationale of bias rating:<br>*They didn´t say the number of events. They did categorizations. They excluded patients with missing data. They selected the predictors based on univariable. They didn’t use competing risks. They didn´t do an overfitting and optimism in model performance.*			

Step 4: Overall assessment

Use the following tables to reach overall judgements about risk of bias and concerns regarding applicability of the prediction model evaluation (development and/or validation) across all assessed domains.
Complete for each evaluation of a distinct model.

**Reaching an overall judgement about risk of bias of the prediction model evaluation**	
**Low risk of bias**	If all domains were rated low risk of bias.<br>If a prediction model was developed without any external validation, and it was rated as low risk of bias for all domains, consider downgrading to **high risk of bias**. Such a model can only be considered as low risk of bias, if the development was based on a very large data set and included some form of internal validation.
**High risk of bias**	If at least one domain is judged to be at **high risk of bias**.
**Unclear risk of bias**	If an unclear risk of bias was noted in at least one domain and it was low risk for all other domains.

**Reaching an overall judgement about applicability of the prediction model evaluation**	
**Low concerns regarding applicability**	If low concerns regarding applicability for all domains, the prediction model evaluation is judged to have **low concerns regarding applicability**.
**High concerns regarding applicability**	If high concerns regarding applicability for at least one domain, the prediction model evaluation is judged to have **high concerns regarding applicability**.
**Unclear concerns regarding applicability**	If unclear concerns (but no "high concern") regarding applicability for at least one domain, the prediction model evaluation is judged to have **unclear concerns regarding applicability** overall.

**Overall judgement about risk of bias and applicability of the prediction model evaluation**		
**Overall judgement of risk of bias**	**RISK:**<br>*(low/ high/ unclear)*	High
Summary of sources of potential bias:<br>Analysis and participants domains show several major issues		
**Overall judgement of applicability**	**CONCERN:**<br>*(low/ high/ unclear)*	High
Summary of applicability concerns:<br><br>Participants domains shows several major issues		

PROBAST

(Prediction model study Risk Of Bias Assessment Tool)

Published in Annals of Internal Medicine (freely available):

1. PROBAST: A Tool to Assess the Risk of Bias and Applicability of Prediction Model Studies
2. PROBAST: A Tool to Assess Risk of Bias and Applicability of Prediction Model Studies: Explanation and Elaboration

What does PROBAST assess?

PROBAST assesses both the *risk of bias* and *concerns regarding applicability* of a study that evaluates (develops, validates or updates) a multivariable diagnostic or prognostic prediction model. It is designed to assess primary studies included in a systematic review.

Bias occurs if systematic flaws or limitations in the design, conduct or analysis of a primary study distort the results. For the purpose of prediction modelling studies, we have defined *risk of bias* to occur when shortcomings in the study design, conduct or analysis lead to systematically distorted estimates of a model's predictive performance or to an inadequate model to address the research question. Model predictive performance is typically evaluated using calibration, discrimination and sometimes classification measures, and these are likely inaccurately estimated in studies with high risk of bias. *Applicability* refers to the extent to which the prediction model from the primary study matches your systematic review question, for example in terms of the participants, predictors or outcome of interest.

A primary study may include the development and/or validation or update of more than one prediction model. A PROBAST assessment should be completed for each distinct model that is developed, validated or updated (extended) for making individualised predictions. Where a publication assesses multiple prediction models, only complete a PROBAST assessment for those models that meet the inclusion criteria for your systematic review. Please note that subsequent use of the term "model" includes derivatives of models, such as simplified risk scores, nomograms, or recalibrations of models.

PROBAST is not designed for all multivariable diagnostic or prognostic studies. For example, studies using multivariable models to identify predictors associated with an outcome but not attempting to develop a model for making individualised predictions are not covered by PROBAST.

PROBAST includes four steps.

Step	Task	When to complete
**1**	Specify your systematic review question(s)	Once per systematic review
**2**	Classify the type of prediction model evaluation	Once for each model of interest in each publication being assessed, for each relevant outcome
**3**	Assess risk of bias and applicability	Once for each development and validation of each distinct prediction model in a publication
**4**	Overall judgment	Once for each development and validation of each distinct prediction model in a publication

If this is your first time using PROBAST, we strongly recommend reading the detailed explanation and elaboration (E&E, see link above) paper and to check the examples on www.probast.org

Step 1: Specify your systematic review question

State your systematic review question to facilitate the assessment of the applicability of the evaluated models to your question. *The following table should be completed once per systematic review.*

Criteria	Specify your systematic review question
Intended use of model:	*To predict cancer specific mortality in patients with renal cancer treated with partial or total nephrectomy regardless of TNM stage*
***Participants** including selection criteria and setting:*	*Patients with renal cancer treated with partial or total nephrectomy regardless of TNM stage*
***Predictors** (used in prediction modelling), including types of predictors (e.g. history, clinical examination, biochemical markers, imaging tests), time of measurement, specific measurement issues (e.g., any requirements/ prohibitions for specialized equipment):*	*Predictors used in clinical practice measured when a nephrectomy for renal cancer is indicated*
Outcome to be predicted:	*Cancer specific mortality*

Step 2: Classify the type of prediction model evaluation

Use the following table to classify the evaluation as model development, model validation or model update, or combination. Different signalling questions apply for different types of prediction model evaluation. If the evaluation does not fit one of these classifications then PROBAST should not be used.

**Classify the evaluation based on its aim**			
**Type of prediction study**	**PROBAST boxes to complete**	**Tick as appropriate**	**Definition for type of prediction model study**
Development only	Development	✗	Prediction model development without external validation. These studies may include internal validation methods, such as bootstrapping and cross-validation techniques.
Development and validation	Development and validation	✓	Prediction model development combined with external validation in other participants in the same article.
Validation only	Validation	✗	External validation of existing (previously developed) model in other participants.

This table should be completed once for each publication being assessed and for each relevant outcome in your review.

**Publication reference**	Chen C, Geng X, Liang R, Zhang D, Sun M. Nomograms-based prediction of overall and cancer-specific survivals for patients with chromophobe renal cell carcinoma. Exp Biol Med (Maywood). 2021 Mar;246(6):729-739. doi: 10.1177/1535370220977107.
**Models of interest**	Nomogram
**Outcome of interest**	*Cancer specific mortality*

Step 3: Assess risk of bias and applicability

PROBAST is structured as four key domains. Each domain is judged for risk of bias (low, high or unclear) and includes signalling questions to help make judgements. Signalling questions are rated as yes (Y), probably yes (PY), probably no (PN), no (N) or no information (NI). All signalling questions are phrased so that "yes" indicates absence of bias. Any signalling question rated as "no" or "probably no" flags the potential for bias; you will need to use your judgement to determine whether the domain should be rated as "high", "low" or "unclear" risk of bias. The guidance document contains further instructions and examples on rating signalling questions and risk of bias for each domain.

The first three domains are also rated for concerns regarding applicability (low/ high/ unclear) to your review question defined above.

Complete all domains separately for each evaluation of a distinct model. Shaded boxes indicate where signalling questions do not apply and should not be answered.

DOMAIN 1: Participants			
**A. Risk of Bias**			
Describe the sources of data and criteria for participant selection:<br><br>*"In our study, the data of 6933 chRCC patients recorded from 2004 to 2015 in SEER were included in this research. The inclusion criteria were specifically described as follows: those patients with only kidney malignancy; patients at least 18 years old; patients with known survival time; patients with known AJCC, T, or N stage. Patients who failed to meet one of these criteria were excluded. A final 4835 chRCC patients were included for OS and CSS analyses, resulting in 60% (2901 chRCC patients) of the subjects in the training group and 40% (1934 chRCC patients) in the validation group"*			
		Dev	Val
1.1 Were appropriate data sources used, e.g. cohort, RCT or nested case-control study data?		Y	Y
1.2 Were all inclusions and exclusions of participants appropriate?		N	N
**Risk of bias introduced by selection of participants**	**RISK:** *(low/ high/ unclear)*	High	High
Rationale of bias rating:<br>*Cohort study included patients without nephrectomy*<br>*Cohort study not included patients with history of another cancer*<br>*Cohort study only included chromophobe renal cancer*			
**B. Applicability**			
Describe included participants, setting and dates:<br>*""In our study, the data of 6933 chRCC patients recorded from 2004 to 2015 in SEER were included in this research. The inclusion criteria were specifically described as follows: those patients with only kidney malignancy; patients at least 18 years old; patients with known survival time; patients with known AJCC, T, or N stage. Patients who failed to meet one of these criteria were excluded. A final 4835 chRCC patients were included for OS and CSS analyses, resulting in 60% (2901 chRCC patients) of the subjects in the training group and 40% (1934 chRCC patients) in the validation group"*			
**Concern that the included participants and setting do not match the review question**	**CONCERN:** *(low/ high/ unclear)*	High	High
Rationale of applicability rating:<br>*Included patients without nephrectomy*<br>*Cohort study not included patients with history of another cancer*<br>*Cohort study only included chromophobe renal cancer*			

DOMAIN 2: Predictors			
**A. Risk of Bias**			
List and describe predictors included in the final model, e.g. definition and timing of assessment:<br><br>The predictors included in the model were: age, grade, N stage, SEER stage, surgery, radiotherapy and chemotherapy<br><br>All the predictors were measured after treatment			
		Dev	Val
2.1 Were predictors defined and assessed in a similar way for all participants?		PY	PY
2.2 Were predictor assessments made without knowledge of outcome data?		PY	PY
2.3 Are all predictors available at the time the model is intended to be used?		PY	PY
**Risk of bias introduced by predictors or their assessment**	**RISK:**<br>*(low/ high/ unclear)*	Low	Low
Rationale of bias rating:<br>*The blinding of measurement is unknown but all the predictors are objective.*			
**B. Applicability**			
Concern that the definition, assessment or timing of predictors in the model do not match the review question	**CONCERN:**<br>*(low/ high/ unclear)*	Low	Low
Rationale of applicability rating:<br>*No major issues identified.*			

DOMAIN 3: Outcome			
**A. Risk of Bias**			
Describe the outcome, how it was defined and determined, and the time interval between predictor assessment and outcome determination:<br>The outcome was cancer specific survival at 3 and 5 years			
		Dev	Val
3.1 Was the outcome determined appropriately?		PY	PY
3.2 Was a pre-specified or standard outcome definition used?		Y	Y
3.3 Were predictors excluded from the outcome definition?		PY	PY
3.4 Was the outcome defined and determined in a similar way for all participants?		PY	PY
3.5 Was the outcome determined without knowledge of predictor information?		PY	PY
3.6 Was the time interval between predictor assessment and outcome determination appropriate?		Y	Y
**Risk of bias introduced by the outcome or its determination**	**RISK:**<br>*(low/ high/ unclear)*	Low	Low
Rationale of bias rating:<br>*No major issues identified*			
**B. Applicability**			
At what time point was the outcome determined:<br>3 and 5 years<br><br>*If a composite outcome was used, describe the relative frequency/distribution of each contributing outcome:*<br>*N/A*			
**Concern that the outcome, its definition, timing or determination do not match the review question**	**CONCERN:**<br>*(low/ high/ unclear)*	Low	Low
Rationale of applicability rating:<br>*The outcome of the primary study matches the outcome of interest of the review*			

DOMAIN 4: Analysis
**Risk of Bias**
Describe numbers of participants, number of candidate predictors, outcome events and events per candidate predictor:<br>*"A final 4835 chRCC patients were included for OS and CSS analyses, resulting in 60% (2901 chRCC patients) of the subjects in the training group and 40% (1934 chRCC patients) in the validation group*<br><br>*A total of 26 predictors were included (Table 3)*<br>*The number of events is unknown*<br><br>*EPV= unknown*
Describe how the model was developed (for example in regards to modelling technique (e.g. survival or logistic modelling), predictor selection, and risk group definition):<br><br>*"To identify the significant characteristics and OS- and CSS- related independent prognostic factors, the information of gender, race, marital status, age, grade, stage (T/N/M), AJCC and SEER stages, histories of chemotherapy, radiotherapy, and surgery of the subjects in the training group were extracted for multivariate and univariate Cox regression analyses. To further evaluate the effects of each factor on survival risks, we applied the hazard ratios (HR) and 95% confidence interval (CI). The significant variables of P < 0.05 were determined for developing the nomograms. HR > 1, HR < 1, and HR ¼ 1 indicated increased degree of risk, decreased degree of risk, and no effect, respectively, as compared with the reference group.*
Describe whether and how the model was validated, either internally (e.g. bootstrapping, cross validation, random split sample) or externally (e.g. temporal validation, geographical validation, different setting, different type of participants):<br><br>*"We calibrated the nomograms for three-year and five-year CSS and OS with data from the validation cohort"*
Describe the performance measures of the model, e.g. (re)calibration, discrimination, (re)classification, net benefit, and whether they were adjusted for optimism:<br>*"The receiver operating characteristic (ROC) curves of the two nomograms were drawn by the MedCalc software (version 15.2.0) and the areas under the curves, which refer to AUC, were calculated. Calibration curves and concordance (C-index) were also employed to evaluate the performance of the nomograms in the prognostic prediction of patients' survival time"*<br>*"In addition, based on the clinical outcome, the accuracy of the nomogram models in prognostic prediction was examined by the decision curve analysis (DCA) for net benefit assessment"*
Describe any participants who were excluded from the analysis:<br>*"The inclusion criteria were specifically described as follows: those patients with only kidney malignancy; patients at least 18 years old; patients with known survival time; patients with known AJCC, T, or N stage. Patients who failed to meet one of these criteria were excluded"*
Describe missing data on predictors and outcomes as well as methods used for missing data:<br>*"The inclusion criteria were specifically described as follows: those patients with only kidney malignancy; patients at least 18 years old; patients with known survival time; patients with known AJCC, T, or N stage.*

Patients who failed to meet one of these criteria were excluded"		
	Dev	Val
4.1 Were there a reasonable number of participants with the outcome?	NI	PY
4.2 Were continuous and categorical predictors handled appropriately?	N	N
4.3 Were all enrolled participants included in the analysis?	N	N
4.4 Were participants with missing data handled appropriately?	N	N
4.5 Was selection of predictors based on univariable analysis avoided?	N	
4.6 Were complexities in the data (e.g. censoring, competing risks, sampling of controls) accounted for appropriately?	N	N
4.7 Were relevant model performance measures evaluated appropriately?	Y	Y
4.8 Were model overfitting and optimism in model performance accounted for?	N	
4.9 Do predictors and their assigned weights in the final model correspond to the results from multivariable analysis?	PY	
**Risk of bias introduced by the analysis** — **RISK:** *(low/ high/ unclear)*	High	High
Rationale of bias rating: *They didn´t say the number of events. They did categorizations. They excluded patients with missing data. They selected the predictors based on univariable. They didn't use competing risks. They didn´t do bootstrapping.*		

Step 4: Overall assessment

Use the following tables to reach overall judgements about risk of bias and concerns regarding applicability of the prediction model evaluation (development and/or validation) across all assessed domains.
Complete for each evaluation of a distinct model.

**Reaching an overall judgement about risk of bias of the prediction model evaluation**	
**Low risk of bias**	If all domains were rated low risk of bias.<br>If a prediction model was developed without any external validation, and it was rated as low risk of bias for all domains, consider downgrading to **high risk of bias**. Such a model can only be considered as low risk of bias, if the development was based on a very large data set and included some form of internal validation.
**High risk of bias**	If at least one domain is judged to be at **high risk of bias**.
**Unclear risk of bias**	If an unclear risk of bias was noted in at least one domain and it was low risk for all other domains.

**Reaching an overall judgement about applicability of the prediction model evaluation**	
**Low concerns regarding applicability**	If low concerns regarding applicability for all domains, the prediction model evaluation is judged to have **low concerns regarding applicability**.
**High concerns regarding applicability**	If high concerns regarding applicability for at least one domain, the prediction model evaluation is judged to have **high concerns regarding applicability**.
**Unclear concerns regarding applicability**	If unclear concerns (but no "high concern") regarding applicability for at least one domain, the prediction model evaluation is judged to have **unclear concerns regarding applicability** overall.

**Overall judgement about risk of bias and applicability of the prediction model evaluation**		
**Overall judgement of risk of bias**	**RISK:**<br>*(low/ high/ unclear)*	High
Summary of sources of potential bias:<br>Analysis and participants domains show several major issues		
**Overall judgement of applicability**	**CONCERN:**<br>*(low/ high/ unclear)*	High
Summary of applicability concerns:<br><br>Participants domains shows several major issues		

PROBAST

(Prediction model study Risk Of Bias Assessment Tool)

Published in Annals of Internal Medicine (freely available):

1. PROBAST: A Tool to Assess the Risk of Bias and Applicability of Prediction Model Studies
2. PROBAST: A Tool to Assess Risk of Bias and Applicability of Prediction Model Studies: Explanation and Elaboration

What does PROBAST assess?

PROBAST assesses both the *risk of bias* and *concerns regarding applicability* of a study that evaluates (develops, validates or updates) a multivariable diagnostic or prognostic prediction model. It is designed to assess primary studies included in a systematic review.

Bias occurs if systematic flaws or limitations in the design, conduct or analysis of a primary study distort the results. For the purpose of prediction modelling studies, we have defined *risk of bias* to occur when shortcomings in the study design, conduct or analysis lead to systematically distorted estimates of a model's predictive performance or to an inadequate model to address the research question. Model predictive performance is typically evaluated using calibration, discrimination and sometimes classification measures, and these are likely inaccurately estimated in studies with high risk of bias. *Applicability* refers to the extent to which the prediction model from the primary study matches your systematic review question, for example in terms of the participants, predictors or outcome of interest.

A primary study may include the development and/or validation or update of more than one prediction model. A PROBAST assessment should be completed for each distinct model that is developed, validated or updated (extended) for making individualised predictions. Where a publication assesses multiple prediction models, only complete a PROBAST assessment for those models that meet the inclusion criteria for your systematic review. Please note that subsequent use of the term "model" includes derivatives of models, such as simplified risk scores, nomograms, or recalibrations of models.

PROBAST is not designed for all multivariable diagnostic or prognostic studies. For example, studies using multivariable models to identify predictors associated with an outcome but not attempting to develop a model for making individualised predictions are not covered by PROBAST.

PROBAST includes four steps.

Step	Task	When to complete
**1**	Specify your systematic review question(s)	Once per systematic review
**2**	Classify the type of prediction model evaluation	Once for each model of interest in each publication being assessed, for each relevant outcome
**3**	Assess risk of bias and applicability	Once for each development and validation of each distinct prediction model in a publication
**4**	Overall judgment	Once for each development and validation of each distinct prediction model in a publication

If this is your first time using PROBAST, we strongly recommend reading the detailed explanation and elaboration (E&E, see link above) paper and to check the examples on www.probast.org

Step 1: Specify your systematic review question

State your systematic review question to facilitate the assessment of the applicability of the evaluated models to your question. *The following table should be completed once per systematic review.*

Criteria	Specify your systematic review question
Intended use of model:	*To predict cancer specific mortality in patients with renal cancer treated with partial or total nephrectomy regardless of TNM stage*
***Participants** including selection criteria and setting:*	*Patients with renal cancer treated with partial or total nephrectomy regardless of TNM stage*
***Predictors** (used in prediction modelling), including types of predictors (e.g. history, clinical examination, biochemical markers, imaging tests), time of measurement, specific measurement issues (e.g., any requirements/ prohibitions for specialized equipment):*	*Predictors used in clinical practice measured when a nephrectomy for renal cancer is indicated*
Outcome to be predicted:	*Cancer specific mortality*

Step 2: Classify the type of prediction model evaluation

Use the following table to classify the evaluation as model development, model validation or model update, or combination. Different signalling questions apply for different types of prediction model evaluation. If the evaluation does not fit one of these classifications then PROBAST should not be used.

**Classify the evaluation based on its aim**			
**Type of prediction study**	**PROBAST boxes to complete**	**Tick as appropriate**	**Definition for type of prediction model study**
Development only	Development	✓	Prediction model development without external validation. These studies may include internal validation methods, such as bootstrapping and cross-validation techniques.
Development and validation	Development and validation	✗	Prediction model development combined with external validation in other participants in the same article.
Validation only	Validation	✗	External validation of existing (previously developed) model in other participants.

This table should be completed once for each publication being assessed and for each relevant outcome in your review.

**Publication reference**	Zhu J, Liu Z, Zhang Z, Fan Y, Chen Y, He Z, Zhou L, Jin J, Shen C, Yu W. Development and internal validation of nomograms for the prediction of postoperative survival of patients with grade 4 renal cell carcinoma (RCC). Transl Androl Urol. 2020 Dec;9(6):2629-2639. doi: 10.21037/tau-19-687.
**Models of interest**	Nomogram
**Outcome of interest**	*Cancer specific mortality*

Step 3: Assess risk of bias and applicability

PROBAST is structured as four key domains. Each domain is judged for risk of bias (low, high or unclear) and includes signalling questions to help make judgements. Signalling questions are rated as yes (Y), probably yes (PY), probably no (PN), no (N) or no information (NI). All signalling questions are phrased so that "yes" indicates absence of bias. Any signalling question rated as "no" or "probably no" flags the potential for bias; you will need to use your judgement to determine whether the domain should be rated as "high", "low" or "unclear" risk of bias. The guidance document contains further instructions and examples on rating signalling questions and risk of bias for each domain.

The first three domains are also rated for concerns regarding applicability (low/ high/ unclear) to your review question defined above.

Complete all domains separately for each evaluation of a distinct model. Shaded boxes indicate where signalling questions do not apply and should not be answered.

DOMAIN 1: Participants			
**A. Risk of Bias**			
Describe the sources of data and criteria for participant selection:<br><br>*"Retrospectively data were collected from patients diagnosed with RCC between January 2013 and October 2018"*<br>*"All tumor specimens with sarcomatoid or rhabdoid differentiation were classified as grade 4, according to the International Society of Urological Pathology (ISUP)"*			
		Dev	Val
1.1 Were appropriate data sources used, e.g. cohort, RCT or nested case-control study data?		Y	N/A
1.2 Were all inclusions and exclusions of participants appropriate?		N	N/A
**Risk of bias introduced by selection of participants**	**RISK:**<br>*(low/ high/ unclear)*	High	N/A
Rationale of bias rating:<br>*The study only included patients with grade 4 RCC*			
**B. Applicability**			
Describe included participants, setting and dates:<br><br>*"Retrospectively data were collected from patients diagnosed with RCC between January 2013 and October 2018"*<br>*"All tumor specimens with sarcomatoid or rhabdoid differentiation were classified as grade 4, according to the International Society of Urological Pathology (ISUP)"*			
**Concern that the included participants and setting do not match the review question**	**CONCERN:**<br>*(low/ high/ unclear)*	High	N/A
Rationale of applicability rating:<br>*The study only included patients with grade 4 RCC*			

DOMAIN 2: Predictors			
**A. Risk of Bias**			
List and describe predictors included in the final model, e.g. definition and timing of assessment:<br><br>The predictors included in the model were: AST, diameter, lymph node metastasis, IMDC risk group<br><br>All the predictors were measured after treatment			
		Dev	Val
2.1 Were predictors defined and assessed in a similar way for all participants?		PY	N/A
2.2 Were predictor assessments made without knowledge of outcome data?		PY	N/A
2.3 Are all predictors available at the time the model is intended to be used?		PY	N/A
**Risk of bias introduced by predictors or their assessment**	**RISK:**<br>*(low/ high/ unclear)*	Low	N/A
Rationale of bias rating:<br>*The blinding of measurement is unknown but all the predictors are objective.*			
**B. Applicability**			
Concern that the definition, assessment or timing of predictors in the model do not match the review question	**CONCERN:**<br>*(low/ high/ unclear)*	Low	N/A
Rationale of applicability rating:<br>*No major issues identified.*			

DOMAIN 3: Outcome			
**A. Risk of Bias**			
Describe the outcome, how it was defined and determined, and the time interval between predictor assessment and outcome determination:<br>The outcome was cancer specific survival at 1, 3 and 5 years			
		Dev	Val
3.1 Was the outcome determined appropriately?		PY	N/A
3.2 Was a pre-specified or standard outcome definition used?		Y	N/A
3.3 Were predictors excluded from the outcome definition?		PY	N/A
3.4 Was the outcome defined and determined in a similar way for all participants?		PY	N/A
3.5 Was the outcome determined without knowledge of predictor information?		PY	N/A
3.6 Was the time interval between predictor assessment and outcome determination appropriate?		Y	N/A
**Risk of bias introduced by the outcome or its determination**	**RISK:**<br>*(low/ high/ unclear)*	Low	N/A
Rationale of bias rating:<br>*No major issues identified*			
**B. Applicability**			
At what time point was the outcome determined:<br>1, 3 and 5 years<br><br>*If a composite outcome was used, describe the relative frequency/distribution of each contributing outcome:*<br>*N/A*			
**Concern that the outcome, its definition, timing or determination do not match the review question**	**CONCERN:**<br>*(low/ high/ unclear)*	Low	N/A
Rationale of applicability rating:<br>*The outcome of the primary study matches the outcome of interest of the review*			

DOMAIN 4: Analysis
**Risk of Bias**
Describe numbers of participants, number of candidate predictors, outcome events and events per candidate predictor:<br>*"One hundred and fifty-four patients underwent partial and radical nephrectomy (RN) at our center were included. Nineteen (12.3%) patients were failed to follow-up, while 135 patients were included in a multivariate analysis of overall survival (OS)"*<br><br>*The total number of predictors is unknown due than the definition of IMDC and MSCKCC predictors is not clear (Table 3)*<br>*The number of events is unknown*<br><br>*EPV= unknown*
Describe how the model was developed (for example in regards to modelling technique (e.g. survival or logistic modelling), predictor selection, and risk group definition):<br><br>*"Univariate and multivariate analyses of OS, PFS, CSS were performed to identify prognostic factors using Cox regression analysis, while hazard ratio (HR) was used to measure the effect of these factors"*
Describe whether and how the model was validated, either internally (e.g. bootstrapping, cross validation, random split sample) or externally (e.g. temporal validation, geographical validation, different setting, different type of participants):<br><br>*"Both Bootstrap validation and calibration plot were used to perform the internal validation of 200 samples"*
Describe the performance measures of the model, e.g. (re)calibration, discrimination, (re)classification, net benefit, and whether they were adjusted for optimism:<br><br>*"The survival curves have been estimated by the Kaplan-Meier method, while nomograms were studied to predict the postoperative (1-, 3-, and 5-year) survival using the multivariate analysis"*<br>*"The predictive ability was assessed using the C-index presented by Harrell et al. (14)"*<br>*"Both Bootstrap validation and calibration plot were used to perform the internal validation of 200 samples"*
Describe any participants who were excluded from the analysis:<br>*"Nineteen (12.3%) patients were failed to follow-up"*
Describe missing data on predictors and outcomes as well as methods used for missing data:<br>*"Nineteen (12.3%) patients were failed to follow-up"*

	Dev	Val
4.1 Were there a reasonable number of participants with the outcome?	NI	N/A
4.2 Were continuous and categorical predictors handled appropriately?	N	N/A
4.3 Were all enrolled participants included in the analysis?	N	N/A
4.4 Were participants with missing data handled appropriately?	N	N/A
4.5 Was selection of predictors based on univariable analysis avoided?	N	

4.6 Were complexities in the data (e.g. censoring, competing risks, sampling of controls) accounted for appropriately?	N	N/A
4.7 Were relevant model performance measures evaluated appropriately?	Y	N/A
4.8 Were model overfitting and optimism in model performance accounted for?	Y	
4.9 Do predictors and their assigned weights in the final model correspond to the results from multivariable analysis?	PY	
**Risk of bias introduced by the analysis** — **RISK:** *(low/ high/ unclear)*	High	N/A

Rationale of bias rating:
They didn´t say the number of events. They did categorizations. They excluded patients with missing data. They selected the predictors based on univariable. They didn't use competing risks.

Step 4: Overall assessment

Use the following tables to reach overall judgements about risk of bias and concerns regarding applicability of the prediction model evaluation (development and/or validation) across all assessed domains.
Complete for each evaluation of a distinct model.

**Reaching an overall judgement about risk of bias of the prediction model evaluation**	
**Low risk of bias**	If all domains were rated low risk of bias.<br>If a prediction model was developed without any external validation, and it was rated as low risk of bias for all domains, consider downgrading to **high risk of bias**. Such a model can only be considered as low risk of bias, if the development was based on a very large data set and included some form of internal validation.
**High risk of bias**	If at least one domain is judged to be at **high risk of bias**.
**Unclear risk of bias**	If an unclear risk of bias was noted in at least one domain and it was low risk for all other domains.

**Reaching an overall judgement about applicability of the prediction model evaluation**	
**Low concerns regarding applicability**	If low concerns regarding applicability for all domains, the prediction model evaluation is judged to have **low concerns regarding applicability**.
**High concerns regarding applicability**	If high concerns regarding applicability for at least one domain, the prediction model evaluation is judged to have **high concerns regarding applicability**.
**Unclear concerns regarding applicability**	If unclear concerns (but no "high concern") regarding applicability for at least one domain, the prediction model evaluation is judged to have **unclear concerns regarding applicability** overall.

**Overall judgement about risk of bias and applicability of the prediction model evaluation**		
**Overall judgement of risk of bias**	**RISK:**<br>*(low/ high/ unclear)*	High
Summary of sources of potential bias:<br>Analysis and participants domains show several major issues		
**Overall judgement of applicability**	**CONCERN:**<br>*(low/ high/ unclear)*	High
Summary of applicability concerns:<br><br>Participants domain shows several major issues		

PROBAST

(Prediction model study Risk Of Bias Assessment Tool)

Published in Annals of Internal Medicine (freely available):

1. PROBAST: A Tool to Assess the Risk of Bias and Applicability of Prediction Model Studies
2. PROBAST: A Tool to Assess Risk of Bias and Applicability of Prediction Model Studies: Explanation and Elaboration

What does PROBAST assess?

PROBAST assesses both the *risk of bias* and *concerns regarding applicability* of a study that evaluates (develops, validates or updates) a multivariable diagnostic or prognostic prediction model. It is designed to assess primary studies included in a systematic review.

Bias occurs if systematic flaws or limitations in the design, conduct or analysis of a primary study distort the results. For the purpose of prediction modelling studies, we have defined *risk of bias* to occur when shortcomings in the study design, conduct or analysis lead to systematically distorted estimates of a model's predictive performance or to an inadequate model to address the research question. Model predictive performance is typically evaluated using calibration, discrimination and sometimes classification measures, and these are likely inaccurately estimated in studies with high risk of bias. *Applicability* refers to the extent to which the prediction model from the primary study matches your systematic review question, for example in terms of the participants, predictors or outcome of interest.

A primary study may include the development and/or validation or update of more than one prediction model. A PROBAST assessment should be completed for each distinct model that is developed, validated or updated (extended) for making individualised predictions. Where a publication assesses multiple prediction models, only complete a PROBAST assessment for those models that meet the inclusion criteria for your systematic review. Please note that subsequent use of the term "model" includes derivatives of models, such as simplified risk scores, nomograms, or recalibrations of models.

PROBAST is not designed for all multivariable diagnostic or prognostic studies. For example, studies using multivariable models to identify predictors associated with an outcome but not attempting to develop a model for making individualised predictions are not covered by PROBAST.

PROBAST includes four steps.

**Step**	**Task**	**When to complete**
**1**	Specify your systematic review question(s)	Once per systematic review
**2**	Classify the type of prediction model evaluation	Once for each model of interest in each publication being assessed, for each relevant outcome
**3**	Assess risk of bias and applicability	Once for each development and validation of each distinct prediction model in a publication
**4**	Overall judgment	Once for each development and validation of each distinct prediction model in a publication

If this is your first time using PROBAST, we strongly recommend reading the detailed explanation and elaboration (E&E, see link above) paper and to check the examples on www.probast.org

Step 1: Specify your systematic review question

State your systematic review question to facilitate the assessment of the applicability of the evaluated models to your question. *The following table should be completed once per systematic review.*

Criteria	Specify your systematic review question
Intended use of model:	*To predict cancer specific mortality in patients with renal cancer treated with partial or total nephrectomy regardless of TNM stage*
***Participants** including selection criteria and setting:*	*Patients with renal cancer treated with partial or total nephrectomy regardless of TNM stage*
***Predictors** (used in prediction modelling), including types of predictors (e.g. history, clinical examination, biochemical markers, imaging tests), time of measurement, specific measurement issues (e.g., any requirements/ prohibitions for specialized equipment):*	*Predictors used in clinical practice measured when a nephrectomy for renal cancer is indicated*
Outcome to be predicted:	*Cancer specific mortality*

Step 2: Classify the type of prediction model evaluation

Use the following table to classify the evaluation as model development, model validation or model update, or combination. Different signalling questions apply for different types of prediction model evaluation. If the evaluation does not fit one of these classifications then PROBAST should not be used.

**Classify the evaluation based on its aim**			
**Type of prediction study**	**PROBAST boxes to complete**	**Tick as appropriate**	**Definition for type of prediction model study**
Development only	Development	✓	Prediction model development without external validation. These studies may include internal validation methods, such as bootstrapping and cross-validation techniques.
Development and validation	Development and validation	✗	Prediction model development combined with external validation in other participants in the same article.
Validation only	Validation	✗	External validation of existing (previously developed) model in other participants.

This table should be completed once for each publication being assessed and for each relevant outcome in your review.

**Publication reference**	Zhang G, Wu Y, Zhang J, Fang Z, Liu Z, XU Z, Fan Y. Nomograms for predicting long-term overall survival and disease-specific survival of patients with clear cell renal cell carcinoma. Onco Targets Ther. 2018 Sep 6;11:5535-5544. doi: 10.2147/OTT.S171881.
**Models of interest**	Nomogram
**Outcome of interest**	*Cancer specific mortality*

Step 3: Assess risk of bias and applicability

PROBAST is structured as four key domains. Each domain is judged for risk of bias (low, high or unclear) and includes signalling questions to help make judgements. Signalling questions are rated as yes (Y), probably yes (PY), probably no (PN), no (N) or no information (NI). All signalling questions are phrased so that "yes" indicates absence of bias. Any signalling question rated as "no" or "probably no" flags the potential for bias; you will need to use your judgement to determine whether the domain should be rated as "high", "low" or "unclear" risk of bias. The guidance document contains further instructions and examples on rating signalling questions and risk of bias for each domain.

The first three domains are also rated for concerns regarding applicability (low/ high/ unclear) to your review question defined above.

Complete all domains separately for each evaluation of a distinct model. Shaded boxes indicate where signalling questions do not apply and should not be answered.

DOMAIN 1: Participants			
**A. Risk of Bias**			
Describe the sources of data and criteria for participant selection:<br><br>*"From 20 cancer registries that cover ~28% of the population of the United States, the SEER program collects and publishes data including cancer incidence and mortality. Only patients diagnosed between 2004 and 2014 with ccRCC were considered. Patients diagnosed before 2004 were excluded since TNM stage information was not recorded in the SEER database until 2004. Additionally, to ensure adequate follow-up time, patients diagnosed after 2014 were not included. From the SEER database, we identified a cohort of 35,151 patients according to the following inclusion criteria: age at diagnosis between 18 and 80 years, known Fuhrman grade, positive histology, active follow-up, known race, known marital status at diagnosis, and surgical treatment with either par- tial or radical nephrectomy (RN). Patients whose race was recorded as American Indian/Alaskan Native or Asian/ Pacific Islander in SEER were assigned to an "others" race category for analysis. Moreover, patients with T0/TX/NX/ MX stage were excluded"*			
		Dev	Val
1.1 Were appropriate data sources used, e.g. cohort, RCT or nested case-control study data?		Y	N/A
1.2 Were all inclusions and exclusions of participants appropriate?		N	N/A
**Risk of bias introduced by selection of participants**	**RISK:**<br>*(low/ high/ unclear)*	High	N/A
Rationale of bias rating:<br>*Cohort study only included clear cell renal cancer*<br>*Cohort study only included patients between 18- 80 years*			
**B. Applicability**			
Describe included participants, setting and dates:<br><br>*"From 20 cancer registries that cover ~28% of the population of the United States, the SEER program collects and publishes data including cancer incidence and mortality. Only patients diagnosed between 2004 and 2014 with ccRCC were considered. Patients diagnosed before 2004 were excluded since TNM stage information was not recorded in the SEER database until 2004. Additionally, to ensure adequate follow-up time, patients diagnosed after 2014 were not included. From the SEER database, we identified a cohort of 35,151 patients according to the following inclusion criteria: age at diagnosis between 18 and 80 years, known Fuhrman grade, positive histology, active follow-up, known race, known marital status at diagnosis, and surgical treatment with either par- tial or radical nephrectomy (RN). Patients whose race was recorded as American Indian/Alaskan Native or Asian/ Pacific Islander in SEER were assigned to an "others" race category for analysis. Moreover, patients with T0/TX/NX/ MX stage were excluded"*			
**Concern that the included participants and setting do not match the review question**	**CONCERN:**<br>*(low/ high/ unclear)*	High	N/A
Rationale of applicability rating:<br>*Cohort study only included clear cell renal cancer*<br>*Cohort study only included patients between 18- 80 years*			

DOMAIN 2: Predictors			
**A. Risk of Bias**			
List and describe predictors included in the final model, e.g. definition and timing of assessment:<br><br>The predictors included in the model were: race, sex, grade, laterality, TNM, nephrectomy and marital status<br><br>All the predictors were measured after treatment			
		Dev	Val
2.1 Were predictors defined and assessed in a similar way for all participants?		PY	N/A
2.2 Were predictor assessments made without knowledge of outcome data?		PY	N/A
2.3 Are all predictors available at the time the model is intended to be used?		PY	N/A
**Risk of bias introduced by predictors or their assessment**	**RISK:**<br>*(low/ high/ unclear)*	Low	N/A
Rationale of bias rating:<br>*The blinding of measurement is unknown but all the predictors are objective.*			
**B. Applicability**			
Concern that the definition, assessment or timing of predictors in the model do not match the review question	**CONCERN:**<br>*(low/ high/ unclear)*	Low	N/A
Rationale of applicability rating:<br>*No major issues identified.*			

DOMAIN 3: Outcome			
**A. Risk of Bias**			
Describe the outcome, how it was defined and determined, and the time interval between predictor assessment and outcome determination:<br>The outcome was cancer specific survival at 3, 5 and 10 years			
		Dev	Val
3.1 Was the outcome determined appropriately?		PY	N/A
3.2 Was a pre-specified or standard outcome definition used?		Y	N/A
3.3 Were predictors excluded from the outcome definition?		PY	N/A
3.4 Was the outcome defined and determined in a similar way for all participants?		PY	N/A
3.5 Was the outcome determined without knowledge of predictor information?		PY	N/A
3.6 Was the time interval between predictor assessment and outcome determination appropriate?		Y	N/A
**Risk of bias introduced by the outcome or its determination**	**RISK:**<br>*(low/ high/ unclear)*	Low	N/A
Rationale of bias rating:<br>*No major issues identified*			
**B. Applicability**			
At what time point was the outcome determined:<br>3, 5 and 10 years<br><br>*If a composite outcome was used, describe the relative frequency/distribution of each contributing outcome:*<br>*N/A*			
**Concern that the outcome, its definition, timing or determination do not match the review question**	**CONCERN:**<br>*(low/ high/ unclear)*	Low	N/A
Rationale of applicability rating:<br>*The outcome of the primary study matches the outcome of interest of the review*			

DOMAIN 4: Analysis		
**Risk of Bias**		
Describe numbers of participants, number of candidate predictors, outcome events and events per candidate predictor:<br>*"According to the inclusion criteria, a total of 35,151 patients were analyzed in this study"*<br><br>*The total number of predictors is 18 (Table 2)*<br>*The number of events were unknown*<br>*EPV =unknown*		
Describe how the model was developed (for example in regards to modelling technique (e.g. survival or logistic modelling), predictor selection, and risk group definition):<br><br>*"The univariable and multivariable Cox regression analyses were used for the analysis of variables that influence OS and DSS, and we use HR to quantify the effect of each variable on OS and DSS. The following variables are selected to be analyzed in Cox regression analysis: race, sex, Fuhrman grade, pathological stage (T/N/M, AJCC, sixth edition), surgical treatment, age at diagnosis, and marital status; the results are shown in Table 2"*		
Describe whether and how the model was validated, either internally (e.g. bootstrapping, cross validation, random split sample) or externally (e.g. temporal validation, geographical validation, different setting, different type of participants):<br><br>*"To decrease the overfit bias, the nomograms were subjected to 1,000 bootstrap resamples for internal validation in the validation cohort"*<br><br>*"The predictive performance of the nomograms was evaluated using an internal bootstrap resampling method"*		
Describe the performance measures of the model, e.g. (re)calibration, discrimination, (re)classification, net benefit, and whether they were adjusted for optimism:<br><br>*"The C-index demonstrated the accuracy of nomograms to predict the 3-, 5-, and 10-year OS and DSS rates of ccRCC patients"*<br><br>*"Marginal estimates and model-average prediction probabilities were used to create calibration curves"*		
Describe any participants who were excluded from the analysis:<br>*"Moreover, patients with T0/TX/NX/ MX stage were excluded"*		
Describe missing data on predictors and outcomes as well as methods used for missing data:<br>*"Moreover, patients with T0/TX/NX/ MX stage were excluded"*		
	Dev	Val
4.1 Were there a reasonable number of participants with the outcome?	NI	N/A
4.2 Were continuous and categorical predictors handled appropriately?	N	N/A
4.3 Were all enrolled participants included in the analysis?	N	N/A
4.4 Were participants with missing data handled appropriately?	N	N/A

4.5 Was selection of predictors based on univariable analysis avoided?		N	
4.6 Were complexities in the data (e.g. censoring, competing risks, sampling of controls) accounted for appropriately?		N	N/A
4.7 Were relevant model performance measures evaluated appropriately?		Y	N/A
4.8 Were model overfitting and optimism in model performance accounted for?		Y	
4.9 Do predictors and their assigned weights in the final model correspond to the results from multivariable analysis?		PY	
**Risk of bias introduced by the analysis**	**RISK:** *(low/ high/ unclear)*	High	N/A
Rationale of bias rating: They number of events is unknown. They did categorizations. They didn´t say anything about missing data. They selected the predictors based on univariable. They didn’t use competing risks.			

Step 4: Overall assessment

Use the following tables to reach overall judgements about risk of bias and concerns regarding applicability of the prediction model evaluation (development and/or validation) across all assessed domains.
Complete for each evaluation of a distinct model.

**Reaching an overall judgement about risk of bias of the prediction model evaluation**	
**Low risk of bias**	If all domains were rated low risk of bias.<br>If a prediction model was developed without any external validation, and it was rated as low risk of bias for all domains, consider downgrading to **high risk of bias**. Such a model can only be considered as low risk of bias, if the development was based on a very large data set and included some form of internal validation.
**High risk of bias**	If at least one domain is judged to be at **high risk of bias**.
**Unclear risk of bias**	If an unclear risk of bias was noted in at least one domain and it was low risk for all other domains.

**Reaching an overall judgement about applicability of the prediction model evaluation**	
**Low concerns regarding applicability**	If low concerns regarding applicability for all domains, the prediction model evaluation is judged to have **low concerns regarding applicability**.
**High concerns regarding applicability**	If high concerns regarding applicability for at least one domain, the prediction model evaluation is judged to have **high concerns regarding applicability**.
**Unclear concerns regarding applicability**	If unclear concerns (but no "high concern") regarding applicability for at least one domain, the prediction model evaluation is judged to have **unclear concerns regarding applicability** overall.

**Overall judgement about risk of bias and applicability of the prediction model evaluation**		
**Overall judgement of risk of bias**	**RISK:**<br>*(low/ high/ unclear)*	High
Summary of sources of potential bias:<br>Analysis and participants domains show several major issues		
**Overall judgement of applicability**	**CONCERN:**<br>*(low/ high/ unclear)*	High
Summary of applicability concerns:<br>Participant domain shows several major issues		

PROBAST

(Prediction model study Risk Of Bias Assessment Tool)

Published in Annals of Internal Medicine (freely available):

1. PROBAST: A Tool to Assess the Risk of Bias and Applicability of Prediction Model Studies
2. PROBAST: A Tool to Assess Risk of Bias and Applicability of Prediction Model Studies: Explanation and Elaboration

What does PROBAST assess?

PROBAST assesses both the *risk of bias* and *concerns regarding applicability* of a study that evaluates (develops, validates or updates) a multivariable diagnostic or prognostic prediction model. It is designed to assess primary studies included in a systematic review.

Bias occurs if systematic flaws or limitations in the design, conduct or analysis of a primary study distort the results. For the purpose of prediction modelling studies, we have defined *risk of bias* to occur when shortcomings in the study design, conduct or analysis lead to systematically distorted estimates of a model's predictive performance or to an inadequate model to address the research question. Model predictive performance is typically evaluated using calibration, discrimination and sometimes classification measures, and these are likely inaccurately estimated in studies with high risk of bias. *Applicability* refers to the extent to which the prediction model from the primary study matches your systematic review question, for example in terms of the participants, predictors or outcome of interest.

A primary study may include the development and/or validation or update of more than one prediction model. A PROBAST assessment should be completed for each distinct model that is developed, validated or updated (extended) for making individualised predictions. Where a publication assesses multiple prediction models, only complete a PROBAST assessment for those models that meet the inclusion criteria for your systematic review. Please note that subsequent use of the term "model" includes derivatives of models, such as simplified risk scores, nomograms, or recalibrations of models.

PROBAST is not designed for all multivariable diagnostic or prognostic studies. For example, studies using multivariable models to identify predictors associated with an outcome but not attempting to develop a model for making individualised predictions are not covered by PROBAST.

PROBAST includes four steps.

Step	Task	When to complete
**1**	Specify your systematic review question(s)	Once per systematic review
**2**	Classify the type of prediction model evaluation	Once for each model of interest in each publication being assessed, for each relevant outcome
**3**	Assess risk of bias and applicability	Once for each development and validation of each distinct prediction model in a publication
**4**	Overall judgment	Once for each development and validation of each distinct prediction model in a publication

If this is your first time using PROBAST, we strongly recommend reading the detailed explanation and elaboration (E&E, see link above) paper and to check the examples on www.probast.org

Step 1: Specify your systematic review question

State your systematic review question to facilitate the assessment of the applicability of the evaluated models to your question. *The following table should be completed once per systematic review.*

Criteria	Specify your systematic review question
Intended use of model:	*To predict cancer specific mortality in patients with renal cancer treated with partial or total nephrectomy regardless of TNM stage*
***Participants** including selection criteria and setting:*	*Patients with renal cancer treated with partial or total nephrectomy regardless of TNM stage*
***Predictors** (used in prediction modelling), including types of predictors (e.g. history, clinical examination, biochemical markers, imaging tests), time of measurement, specific measurement issues (e.g., any requirements/ prohibitions for specialized equipment):*	*Predictors used in clinical practice measured when a nephrectomy for renal cancer is indicated*
Outcome to be predicted:	*Cancer specific mortality*

Step 2: Classify the type of prediction model evaluation

Use the following table to classify the evaluation as model development, model validation or model update, or combination. Different signalling questions apply for different types of prediction model evaluation. If the evaluation does not fit one of these classifications then PROBAST should not be used.

**Classify the evaluation based on its aim**			
**Type of prediction study**	**PROBAST boxes to complete**	**Tick as appropriate**	**Definition for type of prediction model study**
Development only	Development	✗	Prediction model development without external validation. These studies may include internal validation methods, such as bootstrapping and cross-validation techniques.
Development and validation	Development and validation	✓	Prediction model development combined with external validation in other participants in the same article.
Validation only	Validation	✗	External validation of existing (previously developed) model in other participants.

This table should be completed once for each publication being assessed and for each relevant outcome in your review.

**Publication reference**	Zhou W, Huang C, Yuan N. Prognostic nomograms based on log odds of positive lymph nodes for patients with renal cell carcinoma: A retrospective cohort study. Int J Surg. 2018 Dec;60:28-40. doi: 10.1016/j.ijsu.2018.10.038.
**Models of interest**	Nomogram
**Outcome of interest**	*Cancer specific mortality*

Step 3: Assess risk of bias and applicability

PROBAST is structured as four key domains. Each domain is judged for risk of bias (low, high or unclear) and includes signalling questions to help make judgements. Signalling questions are rated as yes (Y), probably yes (PY), probably no (PN), no (N) or no information (NI). All signalling questions are phrased so that “yes” indicates absence of bias. Any signalling question rated as “no” or “probably no” flags the potential for bias; you will need to use your judgement to determine whether the domain should be rated as “high”, “low” or “unclear” risk of bias. The guidance document contains further instructions and examples on rating signalling questions and risk of bias for each domain.

The first three domains are also rated for concerns regarding applicability (low/ high/ unclear) to your review question defined above.

Complete all domains separately for each evaluation of a distinct model. Shaded boxes indicate where signalling questions do not apply and should not be answered.

<table>
<tr><td colspan="4">DOMAIN 1: Participants</td></tr>
<tr><td colspan="4">A. Risk of Bias</td></tr>
<tr><td colspan="4">Describe the sources of data and criteria for participant selection:
“Patents with a diagnosis of RCC between 2005 to 2015 were identified from the SEER database. A total of 166017 individuals were retrieved. The inclusion criteria were as follows: 1) the International Classification of Diseases for Oncology, third edition(ICD-O-3) categories including RCC (8310/3, 8312/3, 8317/3 and 8318/3); 2) patients after cancer-directed surgery of primary tumors; 3) no history of malignancy; 4) age at diagnosis≥18;5) adequate information of grade, SEER stage, T/N/M stage and LN count; 6) radiation with complete data; 7) complete follow-up data and cause of death”</td></tr>
<tr><td colspan="2"></td><td>Dev</td><td>Val</td></tr>
<tr><td colspan="2">1.1 Were appropriate data sources used, e.g. cohort, RCT or nested case-control study data?</td><td>Y</td><td>Y</td></tr>
<tr><td colspan="2">1.2 Were all inclusions and exclusions of participants appropriate?</td><td>N</td><td>N</td></tr>
<tr><td>Risk of bias introduced by selection of participants</td><td>RISK:
(low/ high/ unclear)</td><td>High</td><td>High</td></tr>
<tr><td colspan="4">Rationale of bias rating:
Cohort study not included patients with history of another cancer</td></tr>
<tr><td colspan="4">B. Applicability</td></tr>
<tr><td colspan="4">Describe included participants, setting and dates:
“Patents with a diagnosis of RCC between 2005 to 2015 were identified from the SEER database. A total of 166017 individuals were retrieved. The inclusion criteria were as follows: 1) the International Classification of Diseases for Oncology, third edition(ICD-O-3) categories including RCC (8310/3, 8312/3, 8317/3 and 8318/3); 2) patients after cancer-directed surgery of primary tumors; 3) no history of malignancy; 4) age at diagnosis≥18;5) adequate information of grade, SEER stage, T/N/M stage and LN count; 6) radiation with complete data; 7) complete follow-up data and cause of death”</td></tr>
<tr><td>Concern that the included participants and setting do not match the review question</td><td>CONCERN:
(low/ high/ unclear)</td><td>High</td><td>High</td></tr>
<tr><td colspan="4">Rationale of applicability rating:
Cohort study not included patients with history of another cancer</td></tr>
</table>

DOMAIN 2: Predictors			
**A. Risk of Bias**			
List and describe predictors included in the final model, e.g. definition and timing of assessment:<br><br>The predictors included in the model were: insurance, grade, T stage, SEER stage and LODDS (Figure 3)<br><br>All the predictors were measured after treatment			
		Dev	Val
2.1 Were predictors defined and assessed in a similar way for all participants?		PY	PY
2.2 Were predictor assessments made without knowledge of outcome data?		PY	PY
2.3 Are all predictors available at the time the model is intended to be used?		PY	PY
**Risk of bias introduced by predictors or their assessment**	**RISK:**<br>*(low/ high/ unclear)*	Low	Low
Rationale of bias rating:<br>*The blinding of measurement is unknown but all the predictors are objective.*			
**B. Applicability**			
Concern that the definition, assessment or timing of predictors in the model do not match the review question	**CONCERN:**<br>*(low/ high/ unclear)*	Low	Low
Rationale of applicability rating:<br>*No major issues identified.*			

<table>
<tr><td colspan="4">DOMAIN 3: Outcome</td></tr>
<tr><td colspan="4">A. Risk of Bias</td></tr>
<tr><td colspan="4">Describe the outcome, how it was defined and determined, and the time interval between predictor assessment and outcome determination:
The outcome was cancer specific survival at 1, 3 and 5 years</td></tr>
<tr><td colspan="2"></td><td>Dev</td><td>Val</td></tr>
<tr><td colspan="2">3.1 Was the outcome determined appropriately?</td><td>PY</td><td>PY</td></tr>
<tr><td colspan="2">3.2 Was a pre-specified or standard outcome definition used?</td><td>Y</td><td>Y</td></tr>
<tr><td colspan="2">3.3 Were predictors excluded from the outcome definition?</td><td>PY</td><td>PY</td></tr>
<tr><td colspan="2">3.4 Was the outcome defined and determined in a similar way for all participants?</td><td>PY</td><td>PY</td></tr>
<tr><td colspan="2">3.5 Was the outcome determined without knowledge of predictor information?</td><td>PY</td><td>PY</td></tr>
<tr><td colspan="2">3.6 Was the time interval between predictor assessment and outcome determination appropriate?</td><td>Y</td><td>Y</td></tr>
<tr><td>Risk of bias introduced by the outcome or its determination</td><td>RISK:
(low/ high/ unclear)</td><td>Low</td><td>Low</td></tr>
<tr><td colspan="4">Rationale of bias rating:
No major issues identified</td></tr>
<tr><td colspan="4">B. Applicability</td></tr>
<tr><td colspan="4">At what time point was the outcome determined:
1, 3 and 5 years

If a composite outcome was used, describe the relative frequency/distribution of each contributing outcome:
N/A</td></tr>
<tr><td>Concern that the outcome, its definition, timing or determination do not match the review question</td><td>CONCERN:
(low/ high/ unclear)</td><td>Low</td><td>Low</td></tr>
<tr><td colspan="4">Rationale of applicability rating:
The outcome of the primary study matches the outcome of interest of the review</td></tr>
</table>

DOMAIN 4: Analysis
**Risk of Bias**
Describe numbers of participants, number of candidate predictors, outcome events and events per candidate predictor:<br>*Development cohort=1199*<br>*Validation cohort= 1199*<br>*(Figure 1)*<br><br>*A total of 12 predictors were included (Table 3)*<br>*The number of events is unknown*<br>*EPV= unknown*
Describe how the model was developed (for example in regards to modelling technique (e.g. survival or logistic modelling), predictor selection, and risk group definition):<br><br>*"The univariate and multivariate COX regression analysis were performed to identify risk factors of CSS and OS in development cohort. As shown in Table 3, insurance, grade, SEER stage, T stage, radiation and LODDS were significantly associated with CSS. A nomogram for predicting 1-, 3- and 5-year CSS was built based on the six key risk factors (Figure 3A). According to OS, age, marriage, histologic grade, T stage, SEER stage, radiation and LODDS were considered as independent risk factors (Table 4). A OS-nomogram also constructed using 7 above-mentioned parameters in the development cohort (Figure 3B). Each variable had a corresponding score in the nomograms. The estimated probability of 1-, 3- and 5-year CSS and OS could be calculated, based on locating and summing the scores on the total score scale"*
Describe whether and how the model was validated, either internally (e.g. bootstrapping, cross validation, random split sample) or externally (e.g. temporal validation, geographical validation, different setting, different type of participants):<br><br>*"The performance of nomogram was 1000-bootstrapped validated internally and externally using the C-index and calibration curves"*<br>*"The match cohort was randomly divided into two equally groups including development and validation cohort, to construct and validate the nomogram, respectively"*
Describe the performance measures of the model, e.g. (re)calibration, discrimination, (re)classification, net benefit, and whether they were adjusted for optimism:<br>*"Discrimination and calibration were recorded as appropriate performance evaluation tools. More specifically, concordance index (C-index) was carried out to determine the discrimination of the nomogram, and calibration plots falling on a 1-slope diagonal line would indicate an excellent model"*
Describe any participants who were excluded from the analysis:<br>*Figure 1*<br>*- Age <18: n=2037*<br>*- Unmatched histological type: n=38283*<br>*- Unknown histological grade: n=27123*<br>*- Inadequate information of surgery/ chemotherapy/radiotherapy: n=13289*<br>*- Incomplete follow-up data: n=5973*<br>*- Unknown tumor size: n= 7564*

- Unknown lymph node count: n= 47851
- Unknown metastatic status: n= 12255
- Multiple primary cancer: n= 4895
- Malignant history: n= 3312
- Mismatched cases: n= 7055

Describe missing data on predictors and outcomes as well as methods used for missing data:
Figure 1
- Age <18: n=2037
- Unmatched histological type: n=38283
- Unknown histological grade: n=27123
- Inadequate information of surgery/ chemotherapy/radiotherapy: n=13289
- Incomplete follow-up data: n=5973
- Unknown tumor size: n= 7564
- Unknown lymph node count: n= 47851
- Unknown metastatic status: n= 12255
- Multiple primary cancer: n= 4895
- Malignant history: n= 3312
- Mismatched cases: n= 7055

		Dev	Val
4.1 Were there a reasonable number of participants with the outcome?		NI	PY
4.2 Were continuous and categorical predictors handled appropriately?		N	N
4.3 Were all enrolled participants included in the analysis?		N	N
4.4 Were participants with missing data handled appropriately?		N	N
4.5 Was selection of predictors based on univariable analysis avoided?		N	
4.6 Were complexities in the data (e.g. censoring, competing risks, sampling of controls) accounted for appropriately?		N	N
4.7 Were relevant model performance measures evaluated appropriately?		Y	Y
4.8 Were model overfitting and optimism in model performance accounted for?		Y	
4.9 Do predictors and their assigned weights in the final model correspond to the results from multivariable analysis?		PY	
**Risk of bias introduced by the analysis**	**RISK:** *(low/ high/ unclear)*	High	High

Rationale of bias rating:
They didn´t say the number of events. They did categorizations. They excluded patients with missing data. They selected the predictors based on univariable. They didn't use competing risks.

Step 4: Overall assessment

Use the following tables to reach overall judgements about risk of bias and concerns regarding applicability of the prediction model evaluation (development and/or validation) across all assessed domains.
Complete for each evaluation of a distinct model.

**Reaching an overall judgement about risk of bias of the prediction model evaluation**	
**Low risk of bias**	If all domains were rated low risk of bias.<br>If a prediction model was developed without any external validation, and it was rated as low risk of bias for all domains, consider downgrading to **high risk of bias**. Such a model can only be considered as low risk of bias, if the development was based on a very large data set and included some form of internal validation.
**High risk of bias**	If at least one domain is judged to be at **high risk of bias**.
**Unclear risk of bias**	If an unclear risk of bias was noted in at least one domain and it was low risk for all other domains.

**Reaching an overall judgement about applicability of the prediction model evaluation**	
**Low concerns regarding applicability**	If low concerns regarding applicability for all domains, the prediction model evaluation is judged to have **low concerns regarding applicability**.
**High concerns regarding applicability**	If high concerns regarding applicability for at least one domain, the prediction model evaluation is judged to have **high concerns regarding applicability**.
**Unclear concerns regarding applicability**	If unclear concerns (but no "high concern") regarding applicability for at least one domain, the prediction model evaluation is judged to have **unclear concerns regarding applicability** overall.

**Overall judgement about risk of bias and applicability of the prediction model evaluation**		
**Overall judgement of risk of bias**	**RISK:**<br>*(low/ high/ unclear)*	High
Summary of sources of potential bias:<br>Analysis and participants domains show several major issues		
**Overall judgement of applicability**	**CONCERN:**<br>*(low/ high/ unclear)*	High
Summary of applicability concerns:<br><br>Participants domain shows several major issues		

PROBAST

(Prediction model study Risk Of Bias Assessment Tool)

Published in Annals of Internal Medicine (freely available):

1. PROBAST: A Tool to Assess the Risk of Bias and Applicability of Prediction Model Studies
2. PROBAST: A Tool to Assess Risk of Bias and Applicability of Prediction Model Studies: Explanation and Elaboration

What does PROBAST assess?
PROBAST assesses both the *risk of bias* and *concerns regarding applicability* of a study that evaluates (develops, validates or updates) a multivariable diagnostic or prognostic prediction model. It is designed to assess primary studies included in a systematic review.

Bias occurs if systematic flaws or limitations in the design, conduct or analysis of a primary study distort the results. For the purpose of prediction modelling studies, we have defined *risk of bias* to occur when shortcomings in the study design, conduct or analysis lead to systematically distorted estimates of a model's predictive performance or to an inadequate model to address the research question. Model predictive performance is typically evaluated using calibration, discrimination and sometimes classification measures, and these are likely inaccurately estimated in studies with high risk of bias. *Applicability* refers to the extent to which the prediction model from the primary study matches your systematic review question, for example in terms of the participants, predictors or outcome of interest.

A primary study may include the development and/or validation or update of more than one prediction model. A PROBAST assessment should be completed for each distinct model that is developed, validated or updated (extended) for making individualised predictions. Where a publication assesses multiple prediction models, only complete a PROBAST assessment for those models that meet the inclusion criteria for your systematic review. Please note that subsequent use of the term "model" includes derivatives of models, such as simplified risk scores, nomograms, or recalibrations of models.

PROBAST is not designed for all multivariable diagnostic or prognostic studies. For example, studies using multivariable models to identify predictors associated with an outcome but not attempting to develop a model for making individualised predictions are not covered by PROBAST.

PROBAST includes four steps.

Step	Task	When to complete
**1**	Specify your systematic review question(s)	Once per systematic review
**2**	Classify the type of prediction model evaluation	Once for each model of interest in each publication being assessed, for each relevant outcome
**3**	Assess risk of bias and applicability	Once for each development and validation of each distinct prediction model in a publication
**4**	Overall judgment	Once for each development and validation of each distinct prediction model in a publication

If this is your first time using PROBAST, we strongly recommend reading the detailed explanation and elaboration (E&E, see link above) paper and to check the examples on www.probast.org

Step 1: Specify your systematic review question

State your systematic review question to facilitate the assessment of the applicability of the evaluated models to your question. *The following table should be completed once per systematic review.*

Criteria	Specify your systematic review question
Intended use of model:	*To predict cancer specific mortality in patients with renal cancer treated with partial or total nephrectomy regardless of TNM stage*
***Participants** including selection criteria and setting:*	*Patients with renal cancer treated with partial or total nephrectomy regardless of TNM stage*
***Predictors** (used in prediction modelling), including types of predictors (e.g. history, clinical examination, biochemical markers, imaging tests), time of measurement, specific measurement issues (e.g., any requirements/ prohibitions for specialized equipment):*	*Predictors used in clinical practice measured when a nephrectomy for renal cancer is indicated*
Outcome to be predicted:	*Cancer specific mortality*

Step 2: Classify the type of prediction model evaluation

Use the following table to classify the evaluation as model development, model validation or model update, or combination. Different signalling questions apply for different types of prediction model evaluation. If the evaluation does not fit one of these classifications then PROBAST should not be used.

**Classify the evaluation based on its aim**			
**Type of prediction study**	**PROBAST boxes to complete**	**Tick as appropriate**	**Definition for type of prediction model study**
Development only	Development	✗	Prediction model development without external validation. These studies may include internal validation methods, such as bootstrapping and cross-validation techniques.
Development and validation	Development and validation	✓	Prediction model development combined with external validation in other participants in the same article.
Validation only	Validation	✗	External validation of existing (previously developed) model in other participants.

This table should be completed once for each publication being assessed and for each relevant outcome in your review.

**Publication reference**	Iimura Y, Saito K, Fujii Y, Kumagai J, Kawakami S, Komai Y, Yonese J, Fukui I, Kihara K. Development and External Validation of a New Outcome Prediction Model for Patients With Clear Cell Renal Cell Carcinoma Treated With Nephrectomy Based on Preoperative Serum C-Reactive Protein and TNM Classification: The TNM-C Score. J Urol. 2009 Mar;181(3):1004-12; discussion 1012. doi: 10.1016/j.juro.2008.10.156.
**Models of interest**	Risk score
**Outcome of interest**	*Cancer specific mortality*

Step 3: Assess risk of bias and applicability

PROBAST is structured as four key domains. Each domain is judged for risk of bias (low, high or unclear) and includes signalling questions to help make judgements. Signalling questions are rated as yes (Y), probably yes (PY), probably no (PN), no (N) or no information (NI). All signalling questions are phrased so that “yes” indicates absence of bias. Any signalling question rated as “no” or “probably no” flags the potential for bias; you will need to use your judgement to determine whether the domain should be rated as “high”, “low” or “unclear” risk of bias. The guidance document contains further instructions and examples on rating signalling questions and risk of bias for each domain.

The first three domains are also rated for concerns regarding applicability (low/ high/ unclear) to your review question defined above.

Complete all domains separately for each evaluation of a distinct model. Shaded boxes indicate where signalling questions do not apply and should not be answered.

DOMAIN 1: Participants			
**A. Risk of Bias**			
Describe the sources of data and criteria for participant selection:<br>*"From 1993 to 2006, 346 consecutive Japanese patients underwent radical or partial nephrectomy due to a diagnosis of RCC at Tokyo Medical and Dental University Hospital. Of the 346 patients 97 were excluded from study due to hemodialysis (39), a pathological condition other than clear cell carcinoma (36), no preoperative CRP evaluation (19), inflammatory disease (2) or bilateral synchro nous tumors (1). The remaining 249 patients with clear cell carcinoma served as the model development cohort"*			
		Dev	Val
1.1 Were appropriate data sources used, e.g. cohort, RCT or nested case-control study data?		Y	Y
1.2 Were all inclusions and exclusions of participants appropriate?		N	N
**Risk of bias introduced by selection of participants**	**RISK:**<br>*(low/ high/ unclear)*	High	High
Rationale of bias rating:<br>*Cohort study only included patients with clear cell papillary renal cancer.*<br>*They excluded patients in hemodialysis*			
**B. Applicability**			
Describe included participants, setting and dates:<br>*"From 1993 to 2006, 346 consecutive Japanese patients underwent radical or partial nephrectomy due to a diagnosis of RCC at Tokyo Medical and Dental University Hospital. Of the 346 patients 97 were excluded from study due to hemodialysis (39), a pathological condition other than clear cell carcinoma (36), no preoperative CRP evaluation (19), inflammatory disease (2) or bilateral synchro nous tumors (1). The remaining 249 patients with clear cell carcinoma served as the model development cohort"*			
**Concern that the included participants and setting do not match the review question**	**CONCERN:**<br>*(low/ high/ unclear)*	High	High
Rationale of applicability rating:<br>*Cohort study only included patients with clear cell papillary renal cancer.*<br>*They excluded patients in hemodialysis*			

DOMAIN 2: Predictors			
**A. Risk of Bias**			
List and describe predictors included in the final model, e.g. definition and timing of assessment:<br><br>The predictors included in the model were: TNM stage and CRP (c-reactive protein)<br><br>CRP was measure preoperatively and TNM after the treatment<br>"All resected tumors were pathologically examined for pathological T (pT) stage, lymph node involvement (N), tumor size, Fuhrman nuclear grade and tumor necrosis by 1 experienced pathologist (JK) who was blinded to patient outcome"			
		Dev	Val
2.1 Were predictors defined and assessed in a similar way for all participants?		PY	PY
2.2 Were predictor assessments made without knowledge of outcome data?		PY	PY
2.3 Are all predictors available at the time the model is intended to be used?		PY	PY
**Risk of bias introduced by predictors or their assessment**	**RISK:**<br>*(low/ high/ unclear)*	Low	Low
Rationale of bias rating:<br>*The* pathologically predictors were measured by a *blinded pathologist. To the rest of predictors the blinding is unknown but all of them are objectives*			
**B. Applicability**			
Concern that the definition, assessment or timing of predictors in the model do not match the review question	**CONCERN:**<br>*(low/ high/ unclear)*	Low	Low
Rationale of applicability rating:<br>*No major issues identified.*			

**DOMAIN 3: Outcome**			
**A. Risk of Bias**			
Describe the outcome, how it was defined and determined, and the time interval between predictor assessment and outcome determination:<br>The outcome was cancer specific survival 5 years			
		Dev	Val
3.1 Was the outcome determined appropriately?		PY	PY
3.2 Was a pre-specified or standard outcome definition used?		Y	Y
3.3 Were predictors excluded from the outcome definition?		PY	PY
3.4 Was the outcome defined and determined in a similar way for all participants?		PY	PY
3.5 Was the outcome determined without knowledge of predictor information?		PY	PY
3.6 Was the time interval between predictor assessment and outcome determination appropriate?		Y	Y
**Risk of bias introduced by the outcome or its determination**	**RISK:**<br>*(low/ high/ unclear)*	Low	Low
Rationale of bias rating:<br>*No major issues identified*			
**B. Applicability**			
At what time point was the outcome determined:<br>5 years<br><br>*If a composite outcome was used, describe the relative frequency/distribution of each contributing outcome:*<br>*N/A*			
**Concern that the outcome, its definition, timing or determination do not match the review question**	**CONCERN:**<br>*(low/ high/ unclear)*	Low	Low
Rationale of applicability rating:<br>*The outcome of the primary study matches the outcome of interest of the review*			

DOMAIN 4: Analysis		
**Risk of Bias**		
Describe numbers of participants, number of candidate predictors, outcome events and events per candidate predictor:<br>*N= 249 (E= 46) to develop the mode*<br>*N= 290 (E= unknown) to validate the model*<br>*EPV= 46/12=3.83*<br><br>*A total of 12 predictors were included (Table 2)*		
Describe how the model was developed (for example in regards to modelling technique (e.g. survival or logistic modelling), predictor selection, and risk group definition):<br><br>*"The association of clinicopathological factors with cancer specific survival was evaluated using Cox proportional hazard models. A new prediction model for cancer specific survival was developed using the regression coefficients from the final multivariate Cox proportional hazard model"*		
Describe whether and how the model was validated, either internally (e.g. bootstrapping, cross validation, random split sample) or externally (e.g. temporal validation, geographical validation, different setting, different type of participants):<br><br>*"The final model was externally validated in an independent cohort of 290 patients with clear cell RCC undergoing nephrectomy at Cancer Institute Hospital, representing an external validation cohort"*		
Describe the performance measures of the model, e.g. (re)calibration, discrimination, (re)classification, net benefit, and whether they were adjusted for optimism:<br>*"The predictive ability of prediction models was evaluated using the c index"*		
Describe any participants who were excluded from the analysis:<br>*"Of the 346 patients 97 were excluded from study due to hemodialysis (39), a pathological condition other than clear cell carcinoma (36), no preoperative CRP evaluation (19), inflammatory disease (2) or bilateral synchronous tumors"*		
Describe missing data on predictors and outcomes as well as methods used for missing data:<br>*"Of the 346 patients 97 were excluded from study due to hemodialysis (39), a pathological condition other than clear cell carcinoma (36), no preoperative CRP evaluation (19), inflammatory disease (2) or bilateral synchronous tumors"*		
	Dev	Val
4.1 Were there a reasonable number of participants with the outcome?	N	PN
4.2 Were continuous and categorical predictors handled appropriately?	N	N
4.3 Were all enrolled participants included in the analysis?	N	N
4.4 Were participants with missing data handled appropriately?	N	N
4.5 Was selection of predictors based on univariable analysis avoided?	N	
4.6 Were complexities in the data (e.g. censoring, competing risks, sampling of controls) accounted for appropriately?	N	N
4.7 Were relevant model performance measures evaluated appropriately?	N	N
4.8 Were model overfitting and optimism in model performance accounted for?	N	
4.9 Do predictors and their assigned weights in the final model correspond to the	PY	

results from multivariable analysis?			
**Risk of bias introduced by the analysis**	**RISK:** *(low/ high/ unclear)*	High	High

Rationale of bias rating:
They didn´t have a reasonable number of patients with the outcome. On the validation cohort they didn´t say the number of events. They did categorisation. They excluded patients with missing data. They selected the predictors based on univariable. They didn't use competing risks. They didn´t do calibration. They didn´t do bootstrapping

Step 4: Overall assessment

Use the following tables to reach overall judgements about risk of bias and concerns regarding applicability of the prediction model evaluation (development and/or validation) across all assessed domains.
Complete for each evaluation of a distinct model.

**Reaching an overall judgement about risk of bias of the prediction model evaluation**	
**Low risk of bias**	If all domains were rated low risk of bias.<br>If a prediction model was developed without any external validation, and it was rated as low risk of bias for all domains, consider downgrading to **high risk of bias**. Such a model can only be considered as low risk of bias, if the development was based on a very large data set and included some form of internal validation.
**High risk of bias**	If at least one domain is judged to be at **high risk of bias**.
**Unclear risk of bias**	If an unclear risk of bias was noted in at least one domain and it was low risk for all other domains.

**Reaching an overall judgement about applicability of the prediction model evaluation**	
**Low concerns regarding applicability**	If low concerns regarding applicability for all domains, the prediction model evaluation is judged to have **low concerns regarding applicability**.
**High concerns regarding applicability**	If high concerns regarding applicability for at least one domain, the prediction model evaluation is judged to have **high concerns regarding applicability**.
**Unclear concerns regarding applicability**	If unclear concerns (but no "high concern") regarding applicability for at least one domain, the prediction model evaluation is judged to have **unclear concerns regarding applicability** overall.

**Overall judgement about risk of bias and applicability of the prediction model evaluation**		
**Overall judgement of risk of bias**	**RISK:**<br>*(low/ high/ unclear)*	High
Summary of sources of potential bias:<br>Analysis and participants domains show several major issues		
**Overall judgement of applicability**	**CONCERN:**<br>*(low/ high/ unclear)*	High
Summary of applicability concerns:<br><br>Participants domain shows several major issues		

PROBAST

(Prediction model study Risk Of Bias Assessment Tool)

Published in Annals of Internal Medicine (freely available):

1. PROBAST: A Tool to Assess the Risk of Bias and Applicability of Prediction Model Studies
2. PROBAST: A Tool to Assess Risk of Bias and Applicability of Prediction Model Studies: Explanation and Elaboration

What does PROBAST assess?

PROBAST assesses both the *risk of bias* and *concerns regarding applicability* of a study that evaluates (develops, validates or updates) a multivariable diagnostic or prognostic prediction model. It is designed to assess primary studies included in a systematic review.

Bias occurs if systematic flaws or limitations in the design, conduct or analysis of a primary study distort the results. For the purpose of prediction modelling studies, we have defined *risk of bias* to occur when shortcomings in the study design, conduct or analysis lead to systematically distorted estimates of a model's predictive performance or to an inadequate model to address the research question. Model predictive performance is typically evaluated using calibration, discrimination and sometimes classification measures, and these are likely inaccurately estimated in studies with high risk of bias. *Applicability* refers to the extent to which the prediction model from the primary study matches your systematic review question, for example in terms of the participants, predictors or outcome of interest.

A primary study may include the development and/or validation or update of more than one prediction model. A PROBAST assessment should be completed for each distinct model that is developed, validated or updated (extended) for making individualised predictions. Where a publication assesses multiple prediction models, only complete a PROBAST assessment for those models that meet the inclusion criteria for your systematic review. Please note that subsequent use of the term "model" includes derivatives of models, such as simplified risk scores, nomograms, or recalibrations of models.

PROBAST is not designed for all multivariable diagnostic or prognostic studies. For example, studies using multivariable models to identify predictors associated with an outcome but not attempting to develop a model for making individualised predictions are not covered by PROBAST.

PROBAST includes four steps.

Step	Task	When to complete
**1**	Specify your systematic review question(s)	Once per systematic review
**2**	Classify the type of prediction model evaluation	Once for each model of interest in each publication being assessed, for each relevant outcome
**3**	Assess risk of bias and applicability	Once for each development and validation of each distinct prediction model in a publication
**4**	Overall judgment	Once for each development and validation of each distinct prediction model in a publication

If this is your first time using PROBAST, we strongly recommend reading the detailed explanation and elaboration (E&E, see link above) paper and to check the examples on www.probast.org

Step 1: Specify your systematic review question

State your systematic review question to facilitate the assessment of the applicability of the evaluated models to your question. *The following table should be completed once per systematic review.*

Criteria	Specify your systematic review question
Intended use of model:	*To predict cancer specific mortality in patients with renal cancer treated with partial or total nephrectomy regardless of TNM stage*
***Participants** including selection criteria and setting:*	*Patients with renal cancer treated with partial or total nephrectomy regardless of TNM stage*
***Predictors** (used in prediction modelling), including types of predictors (e.g. history, clinical examination, biochemical markers, imaging tests), time of measurement, specific measurement issues (e.g., any requirements/ prohibitions for specialized equipment):*	*Predictors used in clinical practice measured when a nephrectomy for renal cancer is indicated*
Outcome to be predicted:	*Cancer specific mortality*

Step 2: Classify the type of prediction model evaluation

Use the following table to classify the evaluation as model development, model validation or model update, or combination. Different signalling questions apply for different types of prediction model evaluation. If the evaluation does not fit one of these classifications then PROBAST should not be used.

**Classify the evaluation based on its aim**			
**Type of prediction study**	**PROBAST boxes to complete**	**Tick as appropriate**	**Definition for type of prediction model study**
Development only	Development	✗	Prediction model development without external validation. These studies may include internal validation methods, such as bootstrapping and cross-validation techniques.
Development and validation	Development and validation	✓	Prediction model development combined with external validation in other participants in the same article.
Validation only	Validation	✗	External validation of existing (previously developed) model in other participants.

This table should be completed once for each publication being assessed and for each relevant outcome in your review.

**Publication reference**	Karakiewicz PI, Suardi N, Capitanio U, Jeldres C, Ficarra V, Cindolo L, de la Taille A, Tostain J, Muders PFA, Bensalah K, Artiban W, Salomon L, Zigeuner R, Valeri A, Descotes JL, Rambeaud JJ, Me'jean A, Montorsi F, Bertini R, Patard JJ. A Preoperative Prognostic Model for Patients Treated with Nephrectomy for Renal Cell Carcinoma. Eur Urol. 2009 Feb;55(2):287-95. doi: 10.1016/j.eururo.2008.07.037.
**Models of interest**	Nomogram
**Outcome of interest**	*Cancer specific mortality*

Step 3: Assess risk of bias and applicability

PROBAST is structured as four key domains. Each domain is judged for risk of bias (low, high or unclear) and includes signalling questions to help make judgements. Signalling questions are rated as yes (Y), probably yes (PY), probably no (PN), no (N) or no information (NI). All signalling questions are phrased so that "yes" indicates absence of bias. Any signalling question rated as "no" or "probably no" flags the potential for bias; you will need to use your judgement to determine whether the domain should be rated as "high", "low" or "unclear" risk of bias. The guidance document contains further instructions and examples on rating signalling questions and risk of bias for each domain.

The first three domains are also rated for concerns regarding applicability (low/ high/ unclear) to your review question defined above.

Complete all domains separately for each evaluation of a distinct model. Shaded boxes indicate where signalling questions do not apply and should not be answered.

DOMAIN 1: Participants			
**A. Risk of Bias**			
Describe the sources of data and criteria for participant selection:<br>*"Five participating institutions contributed data from a total of 2485 patients at various stages of RCC between 1984 and 2006; this constituted the nomogram development cohort. Data from an additional 1978 patients from seven institutions were included in the external validation cohort. All patients were treated with either open radical or partial nephrectomy"*<br>*"In RCC-specific survival analyses, perioperative deaths (within 30 d of surgery) were censored"*			
		Dev	Val
1.1 Were appropriate data sources used, e.g. cohort, RCT or nested case-control study data?		Y	Y
1.2 Were all inclusions and exclusions of participants appropriate?		N	N
**Risk of bias introduced by selection of participants**	**RISK:**<br>*(low/ high/ unclear)*	High	High
Rationale of bias rating:<br>*They excluded events within 30 days of surgery*			
**B. Applicability**			
Describe included participants, setting and dates:<br>*"Five participating institutions contributed data from a total of 2485 patients at various stages of RCC between 1984 and 2006; this constituted the nomogram development cohort. Data from an additional 1978 patients from seven institutions were included in the external validation cohort. All patients were treated with either open radical or partial nephrectomy"*<br>*"In RCC-specific survival analyses, perioperative deaths (within 30 d of surgery) were censored"*			
**Concern that the included participants and setting do not match the review question**	**CONCERN:**<br>*(low/ high/ unclear)*	High	High
Rationale of applicability rating:<br>*They excluded events within 30 days of surgery*			

DOMAIN 2: Predictors			
**A. Risk of Bias**			
List and describe predictors included in the final model, e.g. definition and timing of assessment:<br><br>The predictors included in the model were: age, gender, symptoms, tumor size, T stage and metastasis (Figure 2)<br><br>The predictors were measured at diagnosis			
		Dev	Val
2.1 Were predictors defined and assessed in a similar way for all participants?		PY	PY
2.2 Were predictor assessments made without knowledge of outcome data?		PY	PY
2.3 Are all predictors available at the time the model is intended to be used?		PY	PY
**Risk of bias introduced by predictors or their assessment**	**RISK:**<br>*(low/ high/ unclear)*	Low	Low
Rationale of bias rating:<br>*No major issues identified.*			
**B. Applicability**			
Concern that the definition, assessment or timing of predictors in the model do not match the review question	**CONCERN:**<br>*(low/ high/ unclear)*	Low	Low
Rationale of applicability rating:<br>*No major issues identified.*			

**DOMAIN 3: Outcome**			
**A. Risk of Bias**			
Describe the outcome, how it was defined and determined, and the time interval between predictor assessment and outcome determination:<br>The outcome was cancer specific survival 1,2, 5 and 10 years			
		Dev	Val
3.1 Was the outcome determined appropriately?		PY	PY
3.2 Was a pre-specified or standard outcome definition used?		Y	Y
3.3 Were predictors excluded from the outcome definition?		PY	PY
3.4 Was the outcome defined and determined in a similar way for all participants?		PY	PY
3.5 Was the outcome determined without knowledge of predictor information?		PY	PY
3.6 Was the time interval between predictor assessment and outcome determination appropriate?		Y	Y
**Risk of bias introduced by the outcome or its determination**	**RISK:**<br>*(low/ high/ unclear)*	Low	Low
Rationale of bias rating:<br>*No major issues identified*			
**B. Applicability**			
At what time point was the outcome determined:<br>1, 2, 5 and 10 years<br><br>*If a composite outcome was used, describe the relative frequency/distribution of each contributing outcome:*<br>*N/A*			
**Concern that the outcome, its definition, timing or determination do not match the review question**	**CONCERN:**<br>*(low/ high/ unclear)*	Low	Low
Rationale of applicability rating:<br>*The outcome of the primary study matches the outcome of interest of the review*			

DOMAIN 4: Analysis		
**Risk of Bias**		
Describe numbers of participants, number of candidate predictors, outcome events and events per candidate predictor:<br>*N=* 2474 *(E=* 535*) to develop the mode*<br>*N=* 1972 *(E=* 272*) to validate the model*<br>*EPV= 535/9=59.4*<br>*A total of 9 predictors were included (Table 2)*		
Describe how the model was developed (for example in regards to modelling technique (e.g. survival or logistic modelling), predictor selection, and risk group definition):<br><br>*"Univariate and multivariate Cox regression models addressed time to RCC-specific mortality. Main predictors consisted of the 2002 tumor (T) and metastasis (M) stages, which form the basis for the American Joint Committee on Cancer (AJCC) stages. For patients diagnosed before 2002, clinical data were converted to the 2002 tumor-node-metastasis (TNM) staging system. Additional variables included age, gender, tumor size, and symptom classification. Proportional hazard assumptions were verified systematically for all proposed models using the Grambsch-Therneau residual-based test"*		
Describe whether and how the model was validated, either internally (e.g. bootstrapping, cross validation, random split sample) or externally (e.g. temporal validation, geographical validation, different setting, different type of participants):<br>*"Internal validation relied on 200 bootstrap resamples. Finally, we used the external validation cohort to compare the nomogram-predicted probability of RCC-specific mortality relative to the observed RCC-specific mortality rates at 1 yr, 2 yr, 5 yr, and 10 yr"*		
Describe the performance measures of the model, e.g. (re)calibration, discrimination, (re)classification, net benefit, and whether they were adjusted for optimism:<br>*"In Cox regression models, the AUC is substituted with Harrell's concordance index, which was used in this analysis"*<br>*"The calibration plots of the internally validated nomogram (Fig. 3a) are shown for 1-yr, 2-yr, 5-yr, and 10-yr predictions"*		
Describe any participants who were excluded from the analysis:<br>*"Within the nomogram development cohort (2485 patients), 11 patients were excluded because of missing data regarding tumor size (n = 8), cause of death (n = 2), and pretreatment assessment of the symptom classification (n = 1). Within the external validation cohort (1978 patients), 6 patients were excluded due to missing data regarding gender (n = 1) and age (n = 5)"*		
Describe missing data on predictors and outcomes as well as methods used for missing data:<br>*"Within the nomogram development cohort (2485 patients), 11 patients were excluded because of missing data regarding tumor size (n = 8), cause of death (n = 2), and pretreatment assessment of the symptom classification (n = 1). Within the external validation cohort (1978 patients), 6 patients were excluded due to missing data regarding gender (n = 1) and age (n = 5)"*		
	Dev	Val
4.1 Were there a reasonable number of participants with the outcome?	Y	Y
4.2 Were continuous and categorical predictors handled appropriately?	Y	Y
4.3 Were all enrolled participants included in the analysis?	N	N

4.4 Were participants with missing data handled appropriately?	N	N
4.5 Was selection of predictors based on univariable analysis avoided?	N	
4.6 Were complexities in the data (e.g. censoring, competing risks, sampling of controls) accounted for appropriately?	Y	Y
4.7 Were relevant model performance measures evaluated appropriately?	Y	Y
4.8 Were model overfitting and optimism in model performance accounted for?	Y	
4.9 Do predictors and their assigned weights in the final model correspond to the results from multivariable analysis?	PY	
**Risk of bias introduced by the analysis** — **RISK:** *(low/ high/ unclear)*	High	High
Rationale of bias rating:<br>*They excluded patients with missing data. They selected the predictors based on univariable.*		

Step 4: Overall assessment

Use the following tables to reach overall judgements about risk of bias and concerns regarding applicability of the prediction model evaluation (development and/or validation) across all assessed domains.
Complete for each evaluation of a distinct model.

Reaching an overall judgement about risk of bias of the prediction model evaluation	
**Low risk of bias**	If all domains were rated low risk of bias.<br>If a prediction model was developed without any external validation, and it was rated as low risk of bias for all domains, consider downgrading to **high risk of bias**. Such a model can only be considered as low risk of bias, if the development was based on a very large data set and included some form of internal validation.
**High risk of bias**	If at least one domain is judged to be at **high risk of bias**.
**Unclear risk of bias**	If an unclear risk of bias was noted in at least one domain and it was low risk for all other domains.

Reaching an overall judgement about applicability of the prediction model evaluation	
**Low concerns regarding applicability**	If low concerns regarding applicability for all domains, the prediction model evaluation is judged to have **low concerns regarding applicability**.
**High concerns regarding applicability**	If high concerns regarding applicability for at least one domain, the prediction model evaluation is judged to have **high concerns regarding applicability**.
**Unclear concerns regarding applicability**	If unclear concerns (but no "high concern") regarding applicability for at least one domain, the prediction model evaluation is judged to have **unclear concerns regarding applicability** overall.

Overall judgement about risk of bias and applicability of the prediction model evaluation		
**Overall judgement of risk of bias**	**RISK:**<br>*(low/ high/ unclear)*	High
Summary of sources of potential bias:<br>Analysis and participants domains show several major issues		
**Overall judgement of applicability**	**CONCERN:**<br>*(low/ high/ unclear)*	High
Summary of applicability concerns:<br><br>Participants domain shows several major issues		

PROBAST

(Prediction model study Risk Of Bias Assessment Tool)

Published in Annals of Internal Medicine (freely available):

1. PROBAST: A Tool to Assess the Risk of Bias and Applicability of Prediction Model Studies
2. PROBAST: A Tool to Assess Risk of Bias and Applicability of Prediction Model Studies: Explanation and Elaboration

What does PROBAST assess?

PROBAST assesses both the *risk of bias* and *concerns regarding applicability* of a study that evaluates (develops, validates or updates) a multivariable diagnostic or prognostic prediction model. It is designed to assess primary studies included in a systematic review.

Bias occurs if systematic flaws or limitations in the design, conduct or analysis of a primary study distort the results. For the purpose of prediction modelling studies, we have defined *risk of bias* to occur when shortcomings in the study design, conduct or analysis lead to systematically distorted estimates of a model's predictive performance or to an inadequate model to address the research question. Model predictive performance is typically evaluated using calibration, discrimination and sometimes classification measures, and these are likely inaccurately estimated in studies with high risk of bias. *Applicability* refers to the extent to which the prediction model from the primary study matches your systematic review question, for example in terms of the participants, predictors or outcome of interest.

A primary study may include the development and/or validation or update of more than one prediction model. A PROBAST assessment should be completed for each distinct model that is developed, validated or updated (extended) for making individualised predictions. Where a publication assesses multiple prediction models, only complete a PROBAST assessment for those models that meet the inclusion criteria for your systematic review. Please note that subsequent use of the term "model" includes derivatives of models, such as simplified risk scores, nomograms, or recalibrations of models.

PROBAST is not designed for all multivariable diagnostic or prognostic studies. For example, studies using multivariable models to identify predictors associated with an outcome but not attempting to develop a model for making individualised predictions are not covered by PROBAST.

PROBAST includes four steps.

Step	Task	When to complete
**1**	Specify your systematic review question(s)	Once per systematic review
**2**	Classify the type of prediction model evaluation	Once for each model of interest in each publication being assessed, for each relevant outcome
**3**	Assess risk of bias and applicability	Once for each development and validation of each distinct prediction model in a publication
**4**	Overall judgment	Once for each development and validation of each distinct prediction model in a publication

If this is your first time using PROBAST, we strongly recommend reading the detailed explanation and elaboration (E&E, see link above) paper and to check the examples on www.probast.org

Step 1: Specify your systematic review question

State your systematic review question to facilitate the assessment of the applicability of the evaluated models to your question. *The following table should be completed once per systematic review.*

Criteria	Specify your systematic review question
Intended use of model:	*To predict cancer specific mortality in patients with renal cancer treated with partial or total nephrectomy regardless of TNM stage*
***Participants** including selection criteria and setting:*	*Patients with renal cancer treated with partial or total nephrectomy regardless of TNM stage*
***Predictors** (used in prediction modelling), including types of predictors (e.g. history, clinical examination, biochemical markers, imaging tests), time of measurement, specific measurement issues (e.g., any requirements/ prohibitions for specialized equipment):*	*Predictors used in clinical practice measured when a nephrectomy for renal cancer is indicated*
Outcome to be predicted:	*Cancer specific mortality*

Step 2: Classify the type of prediction model evaluation

Use the following table to classify the evaluation as model development, model validation or model update, or combination. Different signalling questions apply for different types of prediction model evaluation. If the evaluation does not fit one of these classifications then PROBAST should not be used.

**Classify the evaluation based on its aim**			
**Type of prediction study**	**PROBAST boxes to complete**	**Tick as appropriate**	**Definition for type of prediction model study**
Development only	Development	✓	Prediction model development without external validation. These studies may include internal validation methods, such as bootstrapping and cross-validation techniques.
Development and validation	Development and validation	✗	Prediction model development combined with external validation in other participants in the same article.
Validation only	Validation	✗	External validation of existing (previously developed) model in other participants.

This table should be completed once for each publication being assessed and for each relevant outcome in your review.

**Publication reference**	Kanao K, Mizuno R, Kikuchi E, Miyajima A, Nakagawa K, Ohigash T, Nakashima J, Oya M. Preoperative Prognostic Nomogram (Probability Table) for Renal Cell Carcinoma Based on TNM Classification. J Urol. 2009 Feb;181(2):480-5. doi: 10.1016/j.juro.2008.10.017.
**Models of interest**	Nomogram
**Outcome of interest**	*Cancer specific mortality*

Step 3: Assess risk of bias and applicability

PROBAST is structured as four key domains. Each domain is judged for risk of bias (low, high or unclear) and includes signalling questions to help make judgements. Signalling questions are rated as yes (Y), probably yes (PY), probably no (PN), no (N) or no information (NI). All signalling questions are phrased so that “yes” indicates absence of bias. Any signalling question rated as “no” or “probably no” flags the potential for bias; you will need to use your judgement to determine whether the domain should be rated as “high”, “low” or “unclear” risk of bias. The guidance document contains further instructions and examples on rating signalling questions and risk of bias for each domain.

The first three domains are also rated for concerns regarding applicability (low/ high/ unclear) to your review question defined above.

Complete all domains separately for each evaluation of a distinct model. Shaded boxes indicate where signalling questions do not apply and should not be answered.

DOMAIN 1: Participants			
**A. Risk of Bias**			
Describe the sources of data and criteria for participant selection:<br>*"From December 1985 to December 2003, 545 patients with RCC underwent radical nephrectomy or nephron sparing surgery at our institution"*			
		Dev	Val
1.1 Were appropriate data sources used, e.g. cohort, RCT or nested case-control study data?		Y	N/A
1.2 Were all inclusions and exclusions of participants appropriate?		Y	N/A
**Risk of bias introduced by selection of participants**	**RISK:**<br>*(low/ high/ unclear)*	Low	N/A
Rationale of bias rating:<br>*Cohort study with clear inclusion and exclusion criterial*			
**B. Applicability**			
Describe included participants, setting and dates:<br>*"From December 1985 to December 2003, 545 patients with RCC underwent radical nephrectomy or nephron sparing surgery at our institution"*			
**Concern that the included participants and setting do not match the review question**	**CONCERN:**<br>*(low/ high/ unclear)*	Low	N/A
Rationale of applicability rating:<br>*Cohort study with clear inclusion and exclusion criterial*			

DOMAIN 2: Predictors			
**A. Risk of Bias**			
List and describe predictors included in the final model, e.g. definition and timing of assessment:<br><br>The predictors included in the model were: TNM score<br><br>The predictors were measured at diagnosis			
		Dev	Val
2.1 Were predictors defined and assessed in a similar way for all participants?		PY	N/A
2.2 Were predictor assessments made without knowledge of outcome data?		PY	N/A
2.3 Are all predictors available at the time the model is intended to be used?		PY	N/A
**Risk of bias introduced by predictors or their assessment**	**RISK:**<br>*(low/ high/ unclear)*	Low	N/A
Rationale of bias rating:<br>*The blinding of measurement is unknown but all the predictors are objective.*			
**B. Applicability**			
Concern that the definition, assessment or timing of predictors in the model do not match the review question	**CONCERN:**<br>*(low/ high/ unclear)*	Low	N/A
Rationale of applicability rating:<br>*No major issues identified.*			

DOMAIN 3: Outcome			
**A. Risk of Bias**			
Describe the outcome, how it was defined and determined, and the time interval between predictor assessment and outcome determination:<br>The outcome was cancer specific survival at 1, 3 and 5 years			
		Dev	Val
3.1 Was the outcome determined appropriately?		PY	N/A
3.2 Was a pre-specified or standard outcome definition used?		Y	N/A
3.3 Were predictors excluded from the outcome definition?		PY	N/A
3.4 Was the outcome defined and determined in a similar way for all participants?		PY	N/A
3.5 Was the outcome determined without knowledge of predictor information?		PY	N/A
3.6 Was the time interval between predictor assessment and outcome determination appropriate?		Y	N/A
**Risk of bias introduced by the outcome or its determination**	**RISK:**<br>*(low/ high/ unclear)*	Low	N/A
Rationale of bias rating:<br>*No major issues identified*			
**B. Applicability**			
At what time point was the outcome determined:<br>1,3 and 5 years<br><br>*If a composite outcome was used, describe the relative frequency/distribution of each contributing outcome:*<br>*N/A*			
**Concern that the outcome, its definition, timing or determination do not match the review question**	**CONCERN:**<br>*(low/ high/ unclear)*	Low	N/A
Rationale of applicability rating:<br>*The outcome of the primary study matches the outcome of interest of the review*			

DOMAIN 4: Analysis		
**Risk of Bias**		
Describe numbers of participants, number of candidate predictors, outcome events and events per candidate predictor:<br>*N= 545 (E= 60)*<br>*The total number of predictors is 7 (Table 2)*<br>*The number of events were 60*<br>*EPV= 60/7=8.57*		
Describe how the model was developed (for example in regards to modelling technique (e.g. survival or logistic modelling), predictor selection, and risk group definition):<br><br>*"As calculated by the Kaplan-Meier method, cause specific survival was significantly related to the T, N and M classifications (each p <0.001, fig. 1). T factor was significantly divided (T1a and T1b p= 0.007, T1b and T3a p <0.008, T2 and T3b/c p< 0.015, and T3b/c and T4 p < 0.002) except for T2 and T3a (p= 0.784). T, N and M factors were also significant prognostic factors on multivariate analysis using a Cox proportional hazards regression model"*		
Describe whether and how the model was validated, either internally (e.g. bootstrapping, cross validation, random split sample) or externally (e.g. temporal validation, geographical validation, different setting, different type of participants):<br><br>*"A nomogram predicting 1, 3 and 5-year cause specific survival was developed by repeating analysis on 200 bootstrap samples to decrease overfit bias and determine 95% CIs"*		
Describe the performance measures of the model, e.g. (re)calibration, discrimination, (re)classification, net benefit, and whether they were adjusted for optimism:<br><br>*"To validate the nomogram a concordance index was estimated to calculate an unbiased measure of the ability of the nomogram to discriminate among patients"*<br><br>*"Furthermore, calibration was examined by plotting the predictions made by the nomogram against actual 1, 3 and 5-year survival rates, which were measured by the Kaplan-Meier method using 200 bootstrap samples"*		
Describe any participants who were excluded from the analysis:<br>*Not indicated*		
Describe missing data on predictors and outcomes as well as methods used for missing data:<br>*Not indicated*		
	Dev	Val
4.1 Were there a reasonable number of participants with the outcome?	N	N/A
4.2 Were continuous and categorical predictors handled appropriately?	NI	N/A
4.3 Were all enrolled participants included in the analysis?	NI	N/A
4.4 Were participants with missing data handled appropriately?	NI	N/A
4.5 Was selection of predictors based on univariable analysis avoided? Poner abajo	N	
4.6 Were complexities in the data (e.g. censoring, competing risks, sampling of controls) accounted for appropriately?	N	N/A
4.7 Were relevant model performance measures evaluated appropriately?	Y	N/A
4.8 Were model overfitting and optimism in model performance accounted for?	Y	

4.9 Do predictors and their assigned weights in the final model correspond to the results from multivariable analysis?		PY	
**Risk of bias introduced by the analysis**	**RISK:** *(low/ high/ unclear)*	High	N/A
Rationale of bias rating: *They didn´t have a reasonable number of patients with the outcome. They didn´t included continues predictors.* They didn´t say anything about missing data. They didn´t do competing risk.			

Step 4: Overall assessment

Use the following tables to reach overall judgements about risk of bias and concerns regarding applicability of the prediction model evaluation (development and/or validation) across all assessed domains.
Complete for each evaluation of a distinct model.

**Reaching an overall judgement about risk of bias of the prediction model evaluation**	
**Low risk of bias**	If all domains were rated low risk of bias.<br>If a prediction model was developed without any external validation, and it was rated as low risk of bias for all domains, consider downgrading to **high risk of bias**. Such a model can only be considered as low risk of bias, if the development was based on a very large data set and included some form of internal validation.
**High risk of bias**	If at least one domain is judged to be at **high risk of bias**.
**Unclear risk of bias**	If an unclear risk of bias was noted in at least one domain and it was low risk for all other domains.

**Reaching an overall judgement about applicability of the prediction model evaluation**	
**Low concerns regarding applicability**	If low concerns regarding applicability for all domains, the prediction model evaluation is judged to have **low concerns regarding applicability**.
**High concerns regarding applicability**	If high concerns regarding applicability for at least one domain, the prediction model evaluation is judged to have **high concerns regarding applicability**.
**Unclear concerns regarding applicability**	If unclear concerns (but no "high concern") regarding applicability for at least one domain, the prediction model evaluation is judged to have **unclear concerns regarding applicability** overall.

**Overall judgement about risk of bias and applicability of the prediction model evaluation**		
**Overall judgement of risk of bias**	**RISK:**<br>*(low/ high/ unclear)*	High
Summary of sources of potential bias:<br>Analysis domain shows several major issues		
**Overall judgement of applicability**	**CONCERN:**<br>*(low/ high/ unclear)*	Low
Summary of applicability concerns:<br>No major issues		

PROBAST

(Prediction model study Risk Of Bias Assessment Tool)

Published in Annals of Internal Medicine (freely available):

1. PROBAST: A Tool to Assess the Risk of Bias and Applicability of Prediction Model Studies
2. PROBAST: A Tool to Assess Risk of Bias and Applicability of Prediction Model Studies: Explanation and Elaboration

What does PROBAST assess?

PROBAST assesses both the *risk of bias* and *concerns regarding applicability* of a study that evaluates (develops, validates or updates) a multivariable diagnostic or prognostic prediction model. It is designed to assess primary studies included in a systematic review.

Bias occurs if systematic flaws or limitations in the design, conduct or analysis of a primary study distort the results. For the purpose of prediction modelling studies, we have defined *risk of bias* to occur when shortcomings in the study design, conduct or analysis lead to systematically distorted estimates of a model's predictive performance or to an inadequate model to address the research question. Model predictive performance is typically evaluated using calibration, discrimination and sometimes classification measures, and these are likely inaccurately estimated in studies with high risk of bias. *Applicability* refers to the extent to which the prediction model from the primary study matches your systematic review question, for example in terms of the participants, predictors or outcome of interest.

A primary study may include the development and/or validation or update of more than one prediction model. A PROBAST assessment should be completed for each distinct model that is developed, validated or updated (extended) for making individualised predictions. Where a publication assesses multiple prediction models, only complete a PROBAST assessment for those models that meet the inclusion criteria for your systematic review. Please note that subsequent use of the term "model" includes derivatives of models, such as simplified risk scores, nomograms, or recalibrations of models.

PROBAST is not designed for all multivariable diagnostic or prognostic studies. For example, studies using multivariable models to identify predictors associated with an outcome but not attempting to develop a model for making individualised predictions are not covered by PROBAST.

PROBAST includes four steps.

**Step**	**Task**	**When to complete**
**1**	Specify your systematic review question(s)	Once per systematic review
**2**	Classify the type of prediction model evaluation	Once for each model of interest in each publication being assessed, for each relevant outcome
**3**	Assess risk of bias and applicability	Once for each development and validation of each distinct prediction model in a publication
**4**	Overall judgment	Once for each development and validation of each distinct prediction model in a publication

If this is your first time using PROBAST, we strongly recommend reading the detailed explanation and elaboration (E&E, see link above) paper and to check the examples on www.probast.org

Step 1: Specify your systematic review question

State your systematic review question to facilitate the assessment of the applicability of the evaluated models to your question. *The following table should be completed once per systematic review.*

Criteria	Specify your systematic review question
Intended use of model:	*To predict cancer specific mortality in patients with renal cancer treated with partial or total nephrectomy regardless of TNM stage*
***Participants** including selection criteria and setting:*	*Patients with renal cancer treated with partial or total nephrectomy regardless of TNM stage*
***Predictors** (used in prediction modelling), including types of predictors (e.g. history, clinical examination, biochemical markers, imaging tests), time of measurement, specific measurement issues (e.g., any requirements/ prohibitions for specialized equipment):*	*Predictors used in clinical practice measured when a nephrectomy for renal cancer is indicated*
Outcome to be predicted:	*Cancer specific mortality*

Step 2: Classify the type of prediction model evaluation

Use the following table to classify the evaluation as model development, model validation or model update, or combination. Different signalling questions apply for different types of prediction model evaluation. If the evaluation does not fit one of these classifications then PROBAST should not be used.

**Classify the evaluation based on its aim**			
**Type of prediction study**	**PROBAST boxes to complete**	**Tick as appropriate**	**Definition for type of prediction model study**
Development only	Development	✗	Prediction model development without external validation. These studies may include internal validation methods, such as bootstrapping and cross-validation techniques.
Development and validation	Development and validation	✓	Prediction model development combined with external validation in other participants in the same article.
Validation only	Validation	✗	External validation of existing (previously developed) model in other participants.

This table should be completed once for each publication being assessed and for each relevant outcome in your review.

**Publication reference**	KaKarakiewicz PI, Briganti A, Chun GKH, Trin QD, Perrotte P, Ficarra V, Cindolo L, De La Taille A, tostain J, Mulders PFA, Salomon L, Zigeuner R, Prayer-Galetti T, Chautard D, Valeri A, Lechevallier E, Descotes JL, Lang H, Mejean A, Patard JJ. Multi-Institutional Validation of a New Renal Cancer–Specific Survival Nomogram. J Clin Oncol. 2007 Apr 10;25(11):1316-22. doi: 10.1200/JCO.2006.06.1218.
**Models of interest**	Nomogram
**Outcome of interest**	*Cancer specific mortality*

Step 3: Assess risk of bias and applicability

PROBAST is structured as four key domains. Each domain is judged for risk of bias (low, high or unclear) and includes signalling questions to help make judgements. Signalling questions are rated as yes (Y), probably yes (PY), probably no (PN), no (N) or no information (NI). All signalling questions are phrased so that “yes” indicates absence of bias. Any signalling question rated as “no” or “probably no” flags the potential for bias; you will need to use your judgement to determine whether the domain should be rated as “high”, “low” or “unclear” risk of bias. The guidance document contains further instructions and examples on rating signalling questions and risk of bias for each domain.

The first three domains are also rated for concerns regarding applicability (low/ high/ unclear) to your review question defined above.

Complete all domains separately for each evaluation of a distinct model. Shaded boxes indicate where signalling questions do not apply and should not be answered.

DOMAIN 1: Participants			
**A. Risk of Bias**			
Describe the sources of data and criteria for participant selection:<br>*"Five participating institutions contributed 2,576 patients treated with either radical or partial nephrectomy for RC. This cohort constituted the nomogram development cohort, whereas 1,430 additional patients from six institutions were included in the external validation cohort"*<br>*"In cancer-specific survival analyses, perioperative deaths (within 30 days of surgery) were censored"*			
		Dev	Val
1.1 Were appropriate data sources used, e.g. cohort, RCT or nested case-control study data?		Y	Y
1.2 Were all inclusions and exclusions of participants appropriate?		N	N
**Risk of bias introduced by selection of participants**	**RISK:**<br>*(low/ high/ unclear)*	High	High
Rationale of bias rating:<br>*They excluded events within 30 days of surgery*			
**B. Applicability**			
Describe included participants, setting and dates:<br>*"Five participating institutions contributed 2,576 patients treated with either radical or partial nephrectomy for RC. This cohort constituted the nomogram development cohort, whereas 1,430 additional patients from six institutions were included in the external validation cohort"*<br>*"In cancer-specific survival analyses, perioperative deaths (within 30 days of surgery) were censored"*			
**Concern that the included participants and setting do not match the review question**	**CONCERN:**<br>*(low/ high/ unclear)*	High	High
Rationale of applicability rating:<br>*They excluded events within 30 days of surgery*			

<table>
<tr><th colspan="4">DOMAIN 2: Predictors</th></tr>
<tr><th colspan="4">A. Risk of Bias</th></tr>
<tr><td colspan="4">List and describe predictors included in the final model, e.g. definition and timing of assessment:

The predictors included in the model were: TNM stage, tumor size and Fuhrman grade (Figure 1)

The predictors were measured at treatment</td></tr>
<tr><td colspan="2"></td><td>Dev</td><td>Val</td></tr>
<tr><td colspan="2">2.1 Were predictors defined and assessed in a similar way for all participants?</td><td>PY</td><td>PY</td></tr>
<tr><td colspan="2">2.2 Were predictor assessments made without knowledge of outcome data?</td><td>PY</td><td>PY</td></tr>
<tr><td colspan="2">2.3 Are all predictors available at the time the model is intended to be used?</td><td>PY</td><td>PY</td></tr>
<tr><td>Risk of bias introduced by predictors or their assessment</td><td>RISK:
(low/ high/ unclear)</td><td>Low</td><td>Low</td></tr>
<tr><td colspan="4">Rationale of bias rating:
No major issues identified.</td></tr>
<tr><th colspan="4">B. Applicability</th></tr>
<tr><td>Concern that the definition, assessment or timing of predictors in the model do not match the review question</td><td>CONCERN:
(low/ high/ unclear)</td><td>Low</td><td>Low</td></tr>
<tr><td colspan="4">Rationale of applicability rating:
No major issues identified.</td></tr>
</table>

DOMAIN 3: Outcome			
**A. Risk of Bias**			
Describe the outcome, how it was defined and determined, and the time interval between predictor assessment and outcome determination:<br>The outcome was cancer specific survival 1,2, 5 and 10 years			
		Dev	Val
3.1 Was the outcome determined appropriately?		PY	PY
3.2 Was a pre-specified or standard outcome definition used?		Y	Y
3.3 Were predictors excluded from the outcome definition?		PY	PY
3.4 Was the outcome defined and determined in a similar way for all participants?		PY	PY
3.5 Was the outcome determined without knowledge of predictor information?		PY	PY
3.6 Was the time interval between predictor assessment and outcome determination appropriate?		Y	Y
**Risk of bias introduced by the outcome or its determination**	**RISK:**<br>*(low/ high/ unclear)*	Low	Low
Rationale of bias rating:<br>*No major issues identified*			
**B. Applicability**			
At what time point was the outcome determined:<br>1, 2, 5 and 10 years<br><br>*If a composite outcome was used, describe the relative frequency/distribution of each contributing outcome:*<br>*N/A*			
**Concern that the outcome, its definition, timing or determination do not match the review question**	**CONCERN:**<br>*(low/ high/ unclear)*	Low	Low
Rationale of applicability rating:<br>*The outcome of the primary study matches the outcome of interest of the review*			

**DOMAIN 4: Analysis**		
**Risk of Bias**		
Describe numbers of participants, number of candidate predictors, outcome events and events per candidate predictor:<br>*N=* 2530 *(E=* 598*) to develop the mode*<br>*N=* 1377 *(E=* 168*) to validate the model*<br>*EPV= 598/16=37.37*<br>*A total of 16 predictors were included (Table 3)*		
Describe how the model was developed (for example in regards to modelling technique (e.g. survival or logistic modelling), predictor selection, and risk group definition):<br><br>*"Univariable and multivariable Cox regression models addressed time to cancer-specific mortality. Main predictors consisted of the 2002 TNM stages, which form the basis for the AJCC stages. Additional variables included age, sex, tumor size, symptom classification, Fuhrman grade, and the histologic subtypes. Reduced model selection was performed using a backward step- down selection process, which used as stopping rule the Akaike's information criterion. Proportional hazards assumptions were verified systematically for all proposed models, using the Grambsch-Therneau residual-based test.*<br>*Given that a proportion of patients with RC die as a result of other causes, competing risk regression was used to test the significance of the described variables in predicting RC-specific mortality, after accounting for other-cause mortality"*		
Describe whether and how the model was validated, either internally (e.g. bootstrapping, cross validation, random split sample) or externally (e.g. temporal validation, geographical validation, different setting, different type of participants):<br>*"Internal validation relied on 200 bootstrap resamples"*<br>*"Finally, we used the external validation cohort to compare the final, reduced, nomogram-predicted RC-specific mortality versus the observed RC- specific mortality at 1, 2, 5, and 10 years"*		
Describe the performance measures of the model, e.g. (re)calibration, discrimination, (re)classification, net benefit, and whether they were adjusted for optimism:<br>*"In Cox regression models, the area under the curve is substituted with Harrell's concordance index, which was used in this analysis"*<br>*"Calibration plots were generated to explore the performance characteristics of the nomo- gram at 1, 2, 5, and 10 years after nephrectomy"*		
Describe any participants who were excluded from the analysis:<br>*"Within the nomogram development cohort of 2,576, 46 patients were excluded because of missing data on tumor size (n=8), Fuhrman grade (n=5), histologic subtype (n=30), cause of death (n=2), or symptom classification (n=1). Within the external validation cohort of 1,430 patients, 53 patients were excluded due to missing sex (n=1) and histologic subtype (n=52).*		
Describe missing data on predictors and outcomes as well as methods used for missing data:<br>*"Within the nomogram development cohort of 2,576, 46 patients were excluded because of missing data on tumor size (n=8), Fuhrman grade (n=5), histologic subtype (n=30), cause of death (n=2), or symptom classification (n=1). Within the external validation cohort of 1,430 patients, 53 patients were excluded due to missing sex (n=1) and histologic subtype (n=52).*		
	Dev	Val

4.1 Were there a reasonable number of participants with the outcome?		Y	Y
4.2 Were continuous and categorical predictors handled appropriately?		Y	Y
4.3 Were all enrolled participants included in the analysis?		N	N
4.4 Were participants with missing data handled appropriately?		N	N
4.5 Was selection of predictors based on univariable analysis avoided?		N	
4.6 Were complexities in the data (e.g. censoring, competing risks, sampling of controls) accounted for appropriately?		Y	Y
4.7 Were relevant model performance measures evaluated appropriately?		Y	Y
4.8 Were model overfitting and optimism in model performance accounted for?		Y	
4.9 Do predictors and their assigned weights in the final model correspond to the results from multivariable analysis?		PY	
**Risk of bias introduced by the analysis**	**RISK:** *(low/ high/ unclear)*	High	High
Rationale of bias rating: *They excluded patients with missing data. They selected the predictors based on univariable.*			

Step 4: Overall assessment

Use the following tables to reach overall judgements about risk of bias and concerns regarding applicability of the prediction model evaluation (development and/or validation) across all assessed domains.
Complete for each evaluation of a distinct model.

**Reaching an overall judgement about risk of bias of the prediction model evaluation**	
**Low risk of bias**	If all domains were rated low risk of bias.<br>If a prediction model was developed without any external validation, and it was rated as low risk of bias for all domains, consider downgrading to **high risk of bias**. Such a model can only be considered as low risk of bias, if the development was based on a very large data set and included some form of internal validation.
**High risk of bias**	If at least one domain is judged to be at **high risk of bias**.
**Unclear risk of bias**	If an unclear risk of bias was noted in at least one domain and it was low risk for all other domains.

**Reaching an overall judgement about applicability of the prediction model evaluation**	
**Low concerns regarding applicability**	If low concerns regarding applicability for all domains, the prediction model evaluation is judged to have **low concerns regarding applicability**.
**High concerns regarding applicability**	If high concerns regarding applicability for at least one domain, the prediction model evaluation is judged to have **high concerns regarding applicability**.
**Unclear concerns regarding applicability**	If unclear concerns (but no "high concern") regarding applicability for at least one domain, the prediction model evaluation is judged to have **unclear concerns regarding applicability** overall.

**Overall judgement about risk of bias and applicability of the prediction model evaluation**		
**Overall judgement of risk of bias**	**RISK:**<br>*(low/ high/ unclear)*	High
Summary of sources of potential bias:<br>Analysis and participants domains show several major issues		
**Overall judgement of applicability**	**CONCERN:**<br>*(low/ high/ unclear)*	High
Summary of applicability concerns:<br><br>Participants domain shows several major issues		

PROBAST

(Prediction model study Risk Of Bias Assessment Tool)

Published in Annals of Internal Medicine (freely available):

1. PROBAST: A Tool to Assess the Risk of Bias and Applicability of Prediction Model Studies
2. PROBAST: A Tool to Assess Risk of Bias and Applicability of Prediction Model Studies: Explanation and Elaboration

What does PROBAST assess?

PROBAST assesses both the *risk of bias* and *concerns regarding applicability* of a study that evaluates (develops, validates or updates) a multivariable diagnostic or prognostic prediction model. It is designed to assess primary studies included in a systematic review.

Bias occurs if systematic flaws or limitations in the design, conduct or analysis of a primary study distort the results. For the purpose of prediction modelling studies, we have defined *risk of bias* to occur when shortcomings in the study design, conduct or analysis lead to systematically distorted estimates of a model's predictive performance or to an inadequate model to address the research question. Model predictive performance is typically evaluated using calibration, discrimination and sometimes classification measures, and these are likely inaccurately estimated in studies with high risk of bias. *Applicability* refers to the extent to which the prediction model from the primary study matches your systematic review question, for example in terms of the participants, predictors or outcome of interest.

A primary study may include the development and/or validation or update of more than one prediction model. A PROBAST assessment should be completed for each distinct model that is developed, validated or updated (extended) for making individualised predictions. Where a publication assesses multiple prediction models, only complete a PROBAST assessment for those models that meet the inclusion criteria for your systematic review. Please note that subsequent use of the term "model" includes derivatives of models, such as simplified risk scores, nomograms, or recalibrations of models.

PROBAST is not designed for all multivariable diagnostic or prognostic studies. For example, studies using multivariable models to identify predictors associated with an outcome but not attempting to develop a model for making individualised predictions are not covered by PROBAST.

PROBAST includes four steps.

Step	Task	When to complete
**1**	Specify your systematic review question(s)	Once per systematic review
**2**	Classify the type of prediction model evaluation	Once for each model of interest in each publication being assessed, for each relevant outcome
**3**	Assess risk of bias and applicability	Once for each development and validation of each distinct prediction model in a publication
**4**	Overall judgment	Once for each development and validation of each distinct prediction model in a publication

If this is your first time using PROBAST, we strongly recommend reading the detailed explanation and elaboration (E&E, see link above) paper and to check the examples on www.probast.org

Step 1: Specify your systematic review question

State your systematic review question to facilitate the assessment of the applicability of the evaluated models to your question. *The following table should be completed once per systematic review.*

Criteria	Specify your systematic review question
Intended use of model:	*To predict cancer specific mortality in patients with renal cancer treated with partial or total nephrectomy regardless of TNM stage*
***Participants** including selection criteria and setting:*	*Patients with renal cancer treated with partial or total nephrectomy regardless of TNM stage*
***Predictors** (used in prediction modelling), including types of predictors (e.g. history, clinical examination, biochemical markers, imaging tests), time of measurement, specific measurement issues (e.g., any requirements/ prohibitions for specialized equipment):*	*Predictors used in clinical practice measured when a nephrectomy for renal cancer is indicated*
Outcome to be predicted:	*Cancer specific mortality*

Step 2: Classify the type of prediction model evaluation

Use the following table to classify the evaluation as model development, model validation or model update, or combination. Different signalling questions apply for different types of prediction model evaluation. If the evaluation does not fit one of these classifications then PROBAST should not be used.

**Classify the evaluation based on its aim**			
**Type of prediction study**	**PROBAST boxes to complete**	**Tick as appropriate**	**Definition for type of prediction model study**
Development only	Development	✓	Prediction model development without external validation. These studies may include internal validation methods, such as bootstrapping and cross-validation techniques.
Development and validation	Development and validation	✗	Prediction model development combined with external validation in other participants in the same article.
Validation only	Validation	✗	External validation of existing (previously developed) model in other participants.

This table should be completed once for each publication being assessed and for each relevant outcome in your review.

**Publication reference**	Leibovich B, Lohse C, Cheville J, Zaid H, Boorjian SA, Frank I, Thompson RH, Parker WP. Predicting Oncologic Outcomes in Renal Cell Carcinoma After Surgery. Eur Urol. 2018 May;73(5):772-780. doi: 10.1016/j.eururo.2018.01.005
**Models of interest**	Risk score
**Outcome of interest**	*Cancer specific mortality*

Step 3: Assess risk of bias and applicability

PROBAST is structured as four key domains. Each domain is judged for risk of bias (low, high or unclear) and includes signalling questions to help make judgements. Signalling questions are rated as yes (Y), probably yes (PY), probably no (PN), no (N) or no information (NI). All signalling questions are phrased so that "yes" indicates absence of bias. Any signalling question rated as "no" or "probably no" flags the potential for bias; you will need to use your judgement to determine whether the domain should be rated as "high", "low" or "unclear" risk of bias. The guidance document contains further instructions and examples on rating signalling questions and risk of bias for each domain.

The first three domains are also rated for concerns regarding applicability (low/ high/ unclear) to your review question defined above.

Complete all domains separately for each evaluation of a distinct model. Shaded boxes indicate where signalling questions do not apply and should not be answered.

DOMAIN 1: Participants			
**A. Risk of Bias**			
Describe the sources of data and criteria for participant selection:<br><br>*"The Mayo Clinic Nephrectomy Registry was queried to identify binephric patients treated with radical or partial nephrectomy between 1980 and 2010 for sporadic, unilateral, nonmetastatic RCC"*			
		Dev	Val
1.1 Were appropriate data sources used, e.g. cohort, RCT or nested case-control study data?		Y	N/A
1.2 Were all inclusions and exclusions of participants appropriate?		N	N/A
**Risk of bias introduced by selection of participants**	**RISK:**<br>*(low/ high/ unclear)*	High	N/A
Rationale of bias rating:<br>*Cohort study not included patients with metastasis*<br>*Cohort study not included one kidney patients*			
**B. Applicability**			
Describe included participants, setting and dates:<br><br>*"The Mayo Clinic Nephrectomy Registry was queried to identify binephric patients treated with radical or partial nephrectomy between 1980 and 2010 for sporadic, unilateral, nonmetastatic RCC"*			
**Concern that the included participants and setting do not match the review question**	**CONCERN:**<br>*(low/ high/ unclear)*	High	N/A
Rationale of applicability rating:<br>*Cohort study not included patients with metastasis*<br>*Cohort study not included one kidney patients*			

DOMAIN 2: Predictors			
**A. Risk of Bias**			
List and describe predictors included in the final model, e.g. definition and timing of assessment:<br><br>The predictors included in the model were:<br>- Clear cell: age, ECOG, constitutional symptoms, adrenalectomy, surgical margins, grade, coagulative necrosis, sarcomatoid differentiation, tumor size, perinephric or renal sinus fat invasion, tumor thrombus and nodal involment<br>- Papillary: grade, perinephric or renal sinus fat invasion and tumor thrombus<br>- Chromophobe: not indicated<br>All the predictors were measured after the treatment			
		Dev	Val
2.1 Were predictors defined and assessed in a similar way for all participants?		PY	N/A
2.2 Were predictor assessments made without knowledge of outcome data?		PY	N/A
2.3 Are all predictors available at the time the model is intended to be used?		PY	N/A
**Risk of bias introduced by predictors or their assessment**	**RISK:**<br>*(low/ high/ unclear)*	Low	N/A
Rationale of bias rating:<br>*The blinding of measurement is unknown but all the predictors are objective.*			
**B. Applicability**			
Concern that the definition, assessment or timing of predictors in the model do not match the review question	**CONCERN:**<br>*(low/ high/ unclear)*	Low	N/A
Rationale of applicability rating:<br>*No major issues identified.*			

**DOMAIN 3: Outcome**			
**A. Risk of Bias**			
Describe the outcome, how it was defined and determined, and the time interval between predictor assessment and outcome determination:<br>The outcome was cancer specific survival at 5, 10 and 15 years			
		Dev	Val
3.1 Was the outcome determined appropriately?		PY	N/A
3.2 Was a pre-specified or standard outcome definition used?		Y	N/A
3.3 Were predictors excluded from the outcome definition?		PY	N/A
3.4 Was the outcome defined and determined in a similar way for all participants?		PY	N/A
3.5 Was the outcome determined without knowledge of predictor information?		PY	N/A
3.6 Was the time interval between predictor assessment and outcome determination appropriate?		Y	N/A
**Risk of bias introduced by the outcome or its determination**	**RISK:**<br>*(low/ high/ unclear)*	Low	N/A
Rationale of bias rating:<br>*No major issues identified*			
**B. Applicability**			
At what time point was the outcome determined:<br>5, 10 and 15 years<br><br>*If a composite outcome was used, describe the relative frequency/distribution of each contributing outcome:*<br>*N/A*			
**Concern that the outcome, its definition, timing or determination do not match the review question**	**CONCERN:**<br>*(low/ high/ unclear)*	Low	N/A
Rationale of applicability rating:<br>*The outcome of the primary study matches the outcome of interest of the review*			

DOMAIN 4: Analysis		
**Risk of Bias**		
Describe numbers of participants, number of candidate predictors, outcome events and events per candidate predictor:<br>*"A total of 3633 patients were identified, of whom 2726 (75%) were diagnosed with ccRCC, while 607 (17%) had papRCC, and 222 (6%) had chromophobe (chrRCC)"*<br><br>*The total number of predictors is 19 (Table 2)*<br><br>*N Clear cell= 2726 (E= 635)*<br>*EPV= 635/19= 33.42*<br>*N papillary= 607 (E= 45)*<br>*EPV= 45/19= 2.36*<br>*N chromophobe= 222 (E= 22)*<br>*EPV= 22/19= 1.15*		
Describe how the model was developed (for example in regards to modelling technique (e.g. survival or logistic modelling), predictor selection, and risk group definition):<br><br>*"Multivariable Cox proportional hazards regression models were developed using a 500-sample bootstrap resampling approach with forward and backward (ccRCC only) selection with the p value threshold for a feature to enter or leave a model set to 0.1. Features that were retained in 70% or more of the 500 samples were included in the final models"*<br>*"The predicted PFS and CSS rates at 5 yr, 10 yr, and 15 yr were obtained using the previously defined Cox proportional regression models and Fine and Gray proportional subdistribution hazard models accounting for the competing risk of death without progression or death from non-RCC causes"*		
Describe whether and how the model was validated, either internally (e.g. bootstrapping, cross validation, random split sample) or externally (e.g. temporal validation, geographical validation, different setting, different type of participants):<br><br>*"Multivariable Cox proportional hazards regression models were developed using a 500-sample bootstrap resampling approach with forward and backward (ccRCC only) selection with the p value threshold for a feature to enter or leave a model set to 0.1"*		
Describe the performance measures of the model, e.g. (re)calibration, discrimination, (re)classification, net benefit, and whether they were adjusted for optimism:<br><br>*"Predictive ability was summarized using bootstrap-corrected c-indexes, and p values for comparisons between two c-indexes were obtained using a jackknife approach"*		
Describe any participants who were excluded from the analysis:<br>*"Of the remaining patients (excluded from analysis), clear cell papillary RCC was present in 32 (1%), RCC not otherwise specified in 31 (1%), collecting duct in seven (< 1%), and other rare subtypes in eight (< 1%)"*		
Describe missing data on predictors and outcomes as well as methods used for missing data:<br>Not indicated		
	Dev	Val
4.1 Were there a reasonable number of participants with the outcome?	Y/N/	N/A

		N*	
4.2 Were continuous and categorical predictors handled appropriately?		N	N/A
4.3 Were all enrolled participants included in the analysis?		N	N/A
4.4 Were participants with missing data handled appropriately?		NI	N/A
4.5 Was selection of predictors based on univariable analysis avoided?		N	
4.6 Were complexities in the data (e.g. censoring, competing risks, sampling of controls) accounted for appropriately?		Y	N/A
4.7 Were relevant model performance measures evaluated appropriately?		N	N/A
4.8 Were model overfitting and optimism in model performance accounted for?		Y	
4.9 Do predictors and their assigned weights in the final model correspond to the results from multivariable analysis?		PY	
**Risk of bias introduced by the analysis**	**RISK:** *(low/ high/ unclear)*	High	N/A

Rationale of bias rating:

*They hadn´t enough patients with the outcome in papillary and chromophobe models and they had enough patients with the outcome in the clear cell model.

They did categorisation. They didn´t say anything about missing data. They didn´t do calibration. They selected the predictors based on univariable.

Step 4: Overall assessment

Use the following tables to reach overall judgements about risk of bias and concerns regarding applicability of the prediction model evaluation (development and/or validation) across all assessed domains.
Complete for each evaluation of a distinct model.

**Reaching an overall judgement about risk of bias of the prediction model evaluation**	
**Low risk of bias**	If all domains were rated low risk of bias.<br>If a prediction model was developed without any external validation, and it was rated as low risk of bias for all domains, consider downgrading to **high risk of bias**. Such a model can only be considered as low risk of bias, if the development was based on a very large data set and included some form of internal validation.
**High risk of bias**	If at least one domain is judged to be at **high risk of bias**.
**Unclear risk of bias**	If an unclear risk of bias was noted in at least one domain and it was low risk for all other domains.

**Reaching an overall judgement about applicability of the prediction model evaluation**	
**Low concerns regarding applicability**	If low concerns regarding applicability for all domains, the prediction model evaluation is judged to have **low concerns regarding applicability**.
**High concerns regarding applicability**	If high concerns regarding applicability for at least one domain, the prediction model evaluation is judged to have **high concerns regarding applicability**.
**Unclear concerns regarding applicability**	If unclear concerns (but no "high concern") regarding applicability for at least one domain, the prediction model evaluation is judged to have **unclear concerns regarding applicability** overall.

**Overall judgement about risk of bias and applicability of the prediction model evaluation**		
**Overall judgement of risk of bias**	**RISK:**<br>*(low/ high/ unclear)*	High
Summary of sources of potential bias:<br>Analysis and participants domains show several major issues		
**Overall judgement of applicability**	**CONCERN:**<br>*(low/ high/ unclear)*	High
Summary of applicability concerns:<br>Participants domain shows several major issues		

PROBAST

(Prediction model study Risk Of Bias Assessment Tool)

Published in Annals of Internal Medicine (freely available):

1. PROBAST: A Tool to Assess the Risk of Bias and Applicability of Prediction Model Studies
2. PROBAST: A Tool to Assess Risk of Bias and Applicability of Prediction Model Studies: Explanation and Elaboration

What does PROBAST assess?

PROBAST assesses both the *risk of bias* and *concerns regarding applicability* of a study that evaluates (develops, validates or updates) a multivariable diagnostic or prognostic prediction model. It is designed to assess primary studies included in a systematic review.

Bias occurs if systematic flaws or limitations in the design, conduct or analysis of a primary study distort the results. For the purpose of prediction modelling studies, we have defined *risk of bias* to occur when shortcomings in the study design, conduct or analysis lead to systematically distorted estimates of a model's predictive performance or to an inadequate model to address the research question. Model predictive performance is typically evaluated using calibration, discrimination and sometimes classification measures, and these are likely inaccurately estimated in studies with high risk of bias. *Applicability* refers to the extent to which the prediction model from the primary study matches your systematic review question, for example in terms of the participants, predictors or outcome of interest.

A primary study may include the development and/or validation or update of more than one prediction model. A PROBAST assessment should be completed for each distinct model that is developed, validated or updated (extended) for making individualised predictions. Where a publication assesses multiple prediction models, only complete a PROBAST assessment for those models that meet the inclusion criteria for your systematic review. Please note that subsequent use of the term "model" includes derivatives of models, such as simplified risk scores, nomograms, or recalibrations of models.

PROBAST is not designed for all multivariable diagnostic or prognostic studies. For example, studies using multivariable models to identify predictors associated with an outcome but not attempting to develop a model for making individualised predictions are not covered by PROBAST.

PROBAST includes four steps.

Step	Task	When to complete
**1**	Specify your systematic review question(s)	Once per systematic review
**2**	Classify the type of prediction model evaluation	Once for each model of interest in each publication being assessed, for each relevant outcome
**3**	Assess risk of bias and applicability	Once for each development and validation of each distinct prediction model in a publication
**4**	Overall judgment	Once for each development and validation of each distinct prediction model in a publication

If this is your first time using PROBAST, we strongly recommend reading the detailed explanation and elaboration (E&E, see link above) paper and to check the examples on www.probast.org

Step 1: Specify your systematic review question

State your systematic review question to facilitate the assessment of the applicability of the evaluated models to your question. *The following table should be completed once per systematic review.*

Criteria	Specify your systematic review question
Intended use of model:	*To predict cancer specific mortality in patients with renal cancer treated with partial or total nephrectomy regardless of TNM stage*
***Participants** including selection criteria and setting:*	*Patients with renal cancer treated with partial or total nephrectomy regardless of TNM stage*
***Predictors** (used in prediction modelling), including types of predictors (e.g. history, clinical examination, biochemical markers, imaging tests), time of measurement, specific measurement issues (e.g., any requirements/ prohibitions for specialized equipment):*	*Predictors used in clinical practice measured when a nephrectomy for renal cancer is indicated*
Outcome to be predicted:	*Cancer specific mortality*

Step 2: Classify the type of prediction model evaluation

Use the following table to classify the evaluation as model development, model validation or model update, or combination. Different signalling questions apply for different types of prediction model evaluation. If the evaluation does not fit one of these classifications then PROBAST should not be used.

Classify the evaluation based on its aim			
**Type of prediction study**	**PROBAST boxes to complete**	**Tick as appropriate**	**Definition for type of prediction model study**
Development only	Development	✓	Prediction model development without external validation. These studies may include internal validation methods, such as bootstrapping and cross-validation techniques.
Development and validation	Development and validation	✗	Prediction model development combined with external validation in other participants in the same article.
Validation only	Validation	✗	External validation of existing (previously developed) model in other participants.

This table should be completed once for each publication being assessed and for each relevant outcome in your review.

**Publication reference**	May M, Ficarra V, Shariat SF, Zigeuner R, Chromecki T, Cindolo L, Burger M, Gunia S, Geciche B, Wenzl V, Aziz A, Chun F, Becker A, Pahernik S, Simeone C, Longo N, Zucchi A, Antonelli A, Mirone V, Stief C, Novara G, Brookman-May S. Impact of Clinical and Histopathological Parameters on Disease Specific Survival in Patients with Collecting Duct Renal Cell Carcinoma: Development of a Disease Specific Risk Model. J Urol. 2013 Aug;190(2):458-63. doi: 10.1016/j.juro.2013.02.035
**Models of interest**	Risk score
**Outcome of interest**	*Cancer specific mortality*

Step 3: Assess risk of bias and applicability

PROBAST is structured as four key domains. Each domain is judged for risk of bias (low, high or unclear) and includes signalling questions to help make judgements. Signalling questions are rated as yes (Y), probably yes (PY), probably no (PN), no (N) or no information (NI). All signalling questions are phrased so that "yes" indicates absence of bias. Any signalling question rated as "no" or "probably no" flags the potential for bias; you will need to use your judgement to determine whether the domain should be rated as "high", "low" or "unclear" risk of bias. The guidance document contains further instructions and examples on rating signalling questions and risk of bias for each domain.

The first three domains are also rated for concerns regarding applicability (low/ high/ unclear) to your review question defined above.

Complete all domains separately for each evaluation of a distinct model. Shaded boxes indicate where signalling questions do not apply and should not be answered.

<table>
<tr><td colspan="4">DOMAIN 1: Participants</td></tr>
<tr><td colspan="4">A. Risk of Bias</td></tr>
<tr><td colspan="4">Describe the sources of data and criteria for participant selection:
“We analyzed clinical and pathological data on 95 patients (0.68%) with CDRCC from a cohort of 14,047 with RCC at a total of 16 centers of the CORONA and SATURN projects, and the Universities of Hamburg and Regensburg. Patients underwent radical or partial nephrectomy between 1992 and 2010”
“Perioperative deaths (within 30 days of surgery) were censored”</td></tr>
<tr><td colspan="2"></td><td>Dev</td><td>Val</td></tr>
<tr><td colspan="2">1.1 Were appropriate data sources used, e.g. cohort, RCT or nested case-control study data?</td><td>Y</td><td>N/A</td></tr>
<tr><td colspan="2">1.2 Were all inclusions and exclusions of participants appropriate?</td><td>N</td><td>N/A</td></tr>
<tr><td>Risk of bias introduced by selection of participants</td><td>RISK:
(low/ high/ unclear)</td><td>High</td><td>N/A</td></tr>
<tr><td colspan="4">Rationale of bias rating:
Cohort study only included collecting duct renal cancer and they excluded patients who died within 30 days of surgery</td></tr>
<tr><td colspan="4">B. Applicability</td></tr>
<tr><td colspan="4">Describe included participants, setting and dates:
“We analyzed clinical and pathological data on 95 patients (0.68%) with CDRCC from a cohort of 14,047 with RCC at a total of 16 centers of the CORONA and SATURN projects, and the Universities of Hamburg and Regensburg. Patients underwent radical or partial nephrectomy between 1992 and 2010”
“Perioperative deaths (within 30 days of surgery) were censored”</td></tr>
<tr><td>Concern that the included participants and setting do not match the review question</td><td>CONCERN:
(low/ high/ unclear)</td><td>High</td><td>N/A</td></tr>
<tr><td colspan="4">Rationale of applicability rating:
Cohort study only included collecting duct renal cancer and they excluded patients who died within 30 days of surgery</td></tr>
</table>

**DOMAIN 2: Predictors**			
**A. Risk of Bias**			
List and describe predictors included in the final model, e.g. definition and timing of assessment:<br><br>The predictors included in the model were: ASA score, tumor size, M stage, Fuhrman grade and LVI.<br><br>The predictors were measured at diagnosis and on the surgical specimen			
		Dev	Val
2.1 Were predictors defined and assessed in a similar way for all participants?		PY	N/A
2.2 Were predictor assessments made without knowledge of outcome data?		PY	N/A
2.3 Are all predictors available at the time the model is intended to be used?		PY	N/A
**Risk of bias introduced by predictors or their assessment**	**RISK:**<br>*(low/ high/ unclear)*	Low	N/A
Rationale of bias rating:<br>*The blinding of measurement is unknown but all the predictors are objective.*			
**B. Applicability**			
Concern that the definition, assessment or timing of predictors in the model do not match the review question	**CONCERN:**<br>*(low/ high/ unclear)*	Low	N/A
Rationale of applicability rating:<br>*No major issues identified.*			

**DOMAIN 3: Outcome**			
**A. Risk of Bias**			
Describe the outcome, how it was defined and determined, and the time interval between predictor assessment and outcome determination:<br>The outcome was cancer specific survival at 5 years			
		Dev	Val
3.1 Was the outcome determined appropriately?		PY	N/A
3.2 Was a pre-specified or standard outcome definition used?		Y	N/A
3.3 Were predictors excluded from the outcome definition?		PY	N/A
3.4 Was the outcome defined and determined in a similar way for all participants?		PY	N/A
3.5 Was the outcome determined without knowledge of predictor information?		PY	N/A
3.6 Was the time interval between predictor assessment and outcome determination appropriate?		Y	N/A
**Risk of bias introduced by the outcome or its determination**	**RISK:**<br>*(low/ high/ unclear)*	Low	N/A
Rationale of bias rating:<br>*No major issues identified*			
**B. Applicability**			
At what time point was the outcome determined:<br>5 years<br><br>*If a composite outcome was used, describe the relative frequency/distribution of each contributing outcome:*<br>*N/A*			
**Concern that the outcome, its definition, timing or determination do not match the review question**	**CONCERN:**<br>*(low/ high/ unclear)*	Low	N/A
Rationale of applicability rating:<br>*The outcome of the primary study matches the outcome of interest of the review*			

DOMAIN 4: Analysis			
**Risk of Bias**			
Describe numbers of participants, number of candidate predictors, outcome events and events per candidate predictor:<br>*"We analyzed clinical and pathological data on 95 patients (0.68%) with CDRCC"*<br>*The total number of predictors is 16 (Table 1)*<br>*The number of events 53*<br>*EPV= 53/16= 3.3*			
Describe how the model was developed (for example in regards to modelling technique (e.g. survival or logistic modelling), predictor selection, and risk group definition):<br><br>*"Univariable and multivariable Cox proportional hazards regression models with stepwise backward elimination were used to assess the influence of clinical and pathological parameters on DSM. In all models proportional hazards assumptions were verified using the Grambsch-Ther-neau residual based test"*			
Describe whether and how the model was validated, either internally (e.g. bootstrapping, cross validation, random split sample) or externally (e.g. temporal validation, geographical validation, different setting, different type of participants):<br><br>*"It was also validated in the development cohort of 95 patients by bootstrap analysis with 200 repeat samples"*			
Describe the performance measures of the model, e.g. (re)calibration, discrimination, (re)classification, net benefit, and whether they were adjusted for optimism:<br><br>*"Cox regression model discrimination was assessed using the Harrell c-index with a value of 1.0 and 0.5 indicating perfect and poor discrimination, respectively. A point value was calculated based on the beta-regression coefficient of all variables with a significant influence on DSM in the Cox model"*			
Describe any participants who were excluded from the analysis:<br>*"Perioperative deaths (within 30 days of surgery) were censored"*			
Describe missing data on predictors and outcomes as well as methods used for missing data:<br>Not indicated			
		Dev	Val
4.1 Were there a reasonable number of participants with the outcome?		N	N/A
4.2 Were continuous and categorical predictors handled appropriately?		N	N/A
4.3 Were all enrolled participants included in the analysis?		N	N/A
4.4 Were participants with missing data handled appropriately?		NI	N/A
4.5 Was selection of predictors based on univariable analysis avoided?		Y	
4.6 Were complexities in the data (e.g. censoring, competing risks, sampling of controls) accounted for appropriately?		N	N/A
4.7 Were relevant model performance measures evaluated appropriately?		N	N/A
4.8 Were model overfitting and optimism in model performance accounted for?		Y	
4.9 Do predictors and their assigned weights in the final model correspond to the results from multivariable analysis?		PY	
**Risk of bias introduced by the analysis**	**RISK:**<br>*(low/ high/ unclear)*	High	N/A

Rationale of bias rating:<br>They didn´t have a reasonable number of patients with the event. They did categorisation. They didn´t say anything about missing data. They didn´t make competing risk. They didn´t make calibration.

Step 4: Overall assessment

Use the following tables to reach overall judgements about risk of bias and concerns regarding applicability of the prediction model evaluation (development and/or validation) across all assessed domains.
Complete for each evaluation of a distinct model.

Reaching an overall judgement about risk of bias of the prediction model evaluation	
**Low risk of bias**	If all domains were rated low risk of bias.<br>If a <u>prediction model was developed without any external validation</u>, and it was rated as <u>low risk of bias for all domains</u>, consider downgrading to **high risk of bias**. Such a model can only be considered as low risk of bias, if the development was based on a very large data set <u>and</u> included some form of internal validation.
**High risk of bias**	If at least one domain is judged to be at **high risk of bias**.
**Unclear risk of bias**	If an unclear risk of bias was noted in at least one domain and it was low risk for all other domains.

Reaching an overall judgement about applicability of the prediction model evaluation	
**Low concerns regarding applicability**	If low concerns regarding applicability for all domains, the prediction model evaluation is judged to have **low concerns regarding applicability**.
**High concerns regarding applicability**	If high concerns regarding applicability for at least one domain, the prediction model evaluation is judged to have **high concerns regarding applicability**.
**Unclear concerns regarding applicability**	If unclear concerns (but no "high concern") regarding applicability for at least one domain, the prediction model evaluation is judged to have **unclear concerns regarding applicability** overall.

Overall judgement about risk of bias and applicability of the prediction model evaluation		
**Overall judgement of risk of bias**	**RISK:**<br>*(low/ high/ unclear)*	High
Summary of sources of potential bias:<br>Analysis and participants domains show several major issues		
**Overall judgement of applicability**	**CONCERN:**<br>*(low/ high/ unclear)*	High
Summary of applicability concerns:<br>Participants domain shows several major issues		

PROBAST

(Prediction model study Risk Of Bias Assessment Tool)

Published in Annals of Internal Medicine (freely available):

1. PROBAST: A Tool to Assess the Risk of Bias and Applicability of Prediction Model Studies
2. PROBAST: A Tool to Assess Risk of Bias and Applicability of Prediction Model Studies: Explanation and Elaboration

What does PROBAST assess?

PROBAST assesses both the *risk of bias* and *concerns regarding applicability* of a study that evaluates (develops, validates or updates) a multivariable diagnostic or prognostic prediction model. It is designed to assess primary studies included in a systematic review.

Bias occurs if systematic flaws or limitations in the design, conduct or analysis of a primary study distort the results. For the purpose of prediction modelling studies, we have defined *risk of bias* to occur when shortcomings in the study design, conduct or analysis lead to systematically distorted estimates of a model's predictive performance or to an inadequate model to address the research question. Model predictive performance is typically evaluated using calibration, discrimination and sometimes classification measures, and these are likely inaccurately estimated in studies with high risk of bias. *Applicability* refers to the extent to which the prediction model from the primary study matches your systematic review question, for example in terms of the participants, predictors or outcome of interest.

A primary study may include the development and/or validation or update of more than one prediction model. A PROBAST assessment should be completed for each distinct model that is developed, validated or updated (extended) for making individualised predictions. Where a publication assesses multiple prediction models, only complete a PROBAST assessment for those models that meet the inclusion criteria for your systematic review. Please note that subsequent use of the term "model" includes derivatives of models, such as simplified risk scores, nomograms, or recalibrations of models.

PROBAST is not designed for all multivariable diagnostic or prognostic studies. For example, studies using multivariable models to identify predictors associated with an outcome but not attempting to develop a model for making individualised predictions are not covered by PROBAST.

PROBAST includes four steps.

Step	Task	When to complete
**1**	Specify your systematic review question(s)	Once per systematic review
**2**	Classify the type of prediction model evaluation	Once for each model of interest in each publication being assessed, for each relevant outcome
**3**	Assess risk of bias and applicability	Once for each development and validation of each distinct prediction model in a publication
**4**	Overall judgment	Once for each development and validation of each distinct prediction model in a publication

If this is your first time using PROBAST, we strongly recommend reading the detailed explanation and elaboration (E&E, see link above) paper and to check the examples on www.probast.org

Step 1: Specify your systematic review question

State your systematic review question to facilitate the assessment of the applicability of the evaluated models to your question. *The following table should be completed once per systematic review.*

Criteria	Specify your systematic review question
Intended use of model:	*To predict cancer specific mortality in patients with renal cancer treated with partial or total nephrectomy regardless of TNM stage*
***Participants** including selection criteria and setting:*	*Patients with renal cancer treated with partial or total nephrectomy regardless of TNM stage*
***Predictors** (used in prediction modelling), including types of predictors (e.g. history, clinical examination, biochemical markers, imaging tests), time of measurement, specific measurement issues (e.g., any requirements/ prohibitions for specialized equipment):*	*Predictors used in clinical practice measured when a nephrectomy for renal cancer is indicated*
Outcome to be predicted:	*Cancer specific mortality*

Step 2: Classify the type of prediction model evaluation

Use the following table to classify the evaluation as model development, model validation or model update, or combination. Different signalling questions apply for different types of prediction model evaluation. If the evaluation does not fit one of these classifications then PROBAST should not be used.

**Classify the evaluation based on its aim**			
**Type of prediction study**	**PROBAST boxes to complete**	**Tick as appropriate**	**Definition for type of prediction model study**
Development only	Development	✗	Prediction model development without external validation. These studies may include internal validation methods, such as bootstrapping and cross-validation techniques.
Development and validation	Development and validation	✓	Prediction model development combined with external validation in other participants in the same article.
Validation only	Validation	✗	External validation of existing (previously developed) model in other participants.

This table should be completed once for each publication being assessed and for each relevant outcome in your review.

**Publication reference**	Klatte T, Remzi M, Zigeuner RE, Mannweiler S, Said JW, Kabbinavar FF, Haitel A, Waldert M, Martino M, Marberger M, Belldegrun AS, Pantuck AJ. Development and External Validation of a Nomogram Predicting Disease Specific Survival After Nephrectomy for Papillary Renal Cell Carcinoma. J Urol. 2010 Jul;184(1):53-8. doi: 10.1016/j.juro.2010.03.026
**Models of interest**	Nomogram
**Outcome of interest**	*Cancer specific mortality*

Step 3: Assess risk of bias and applicability

PROBAST is structured as four key domains. Each domain is judged for risk of bias (low, high or unclear) and includes signalling questions to help make judgements. Signalling questions are rated as yes (Y), probably yes (PY), probably no (PN), no (N) or no information (NI). All signalling questions are phrased so that “yes” indicates absence of bias. Any signalling question rated as “no” or “probably no” flags the potential for bias; you will need to use your judgement to determine whether the domain should be rated as “high”, “low” or “unclear” risk of bias. The guidance document contains further instructions and examples on rating signalling questions and risk of bias for each domain.

The first three domains are also rated for concerns regarding applicability (low/ high/ unclear) to your review question defined above.

Complete all domains separately for each evaluation of a distinct model. Shaded boxes indicate where signalling questions do not apply and should not be answered.

<table>
<tr><th colspan="4">DOMAIN 1: Participants</th></tr>
<tr><th colspan="4">A. Risk of Bias</th></tr>
<tr><td colspan="4">Describe the sources of data and criteria for participant selection:
“Patients who underwent radical or partial nephrectomy between 1984 and 2008 for unilateral sporadic PRCC were included in analysis. Patients with renal tumors of other subtypes, those who presented with bilateral, familial or hereditary RCC, 10 who did not undergo nephrectomy as part of cancer treatment, 13 with positive surgical margins on pathology assessment and 130 with no slides available for review were excluded. Of 5,408 patients with a renal tumor 435 met inclusion criteria and form the principal study population”</td></tr>
<tr><td colspan="2"></td><td>Dev</td><td>Val</td></tr>
<tr><td colspan="2">1.1 Were appropriate data sources used, e.g. cohort, RCT or nested case-control study data?</td><td>Y</td><td>Y</td></tr>
<tr><td colspan="2">1.2 Were all inclusions and exclusions of participants appropriate?</td><td>N</td><td>N</td></tr>
<tr><td>Risk of bias introduced by selection of participants</td><td>RISK:
(low/ high/ unclear)</td><td>High</td><td>High</td></tr>
<tr><td colspan="4">Rationale of bias rating:
Cohort study only included patients with papillary renal cancer
They excluded patients with positive surgical margins</td></tr>
<tr><th colspan="4">B. Applicability</th></tr>
<tr><td colspan="4">Describe included participants, setting and dates:
“Patients who underwent radical or partial nephrectomy between 1984 and 2008 for unilateral sporadic PRCC were included in analysis. Patients with renal tumors of other subtypes, those who presented with bilateral, familial or hereditary RCC, 10 who did not undergo nephrectomy as part of cancer treatment, 13 with positive surgical margins on pathology assessment and 130 with no slides available for review were excluded. Of 5,408 patients with a renal tumor 435 met inclusion criteria and form the principal study population”</td></tr>
<tr><td>Concern that the included participants and setting do not match the review question</td><td>CONCERN:
(low/ high/ unclear)</td><td>High</td><td>High</td></tr>
<tr><td colspan="4">Rationale of applicability rating:
Cohort study only included patients with papillary renal cancer
They excluded patients with positive surgical margins</td></tr>
</table>

DOMAIN 2: Predictors			
**A. Risk of Bias**			
List and describe predictors included in the final model, e.g. definition and timing of assessment:<br><br>The predictors included in the model were: symptoms, T and M stage, vascular invasion and necrosis (Figure 2)<br><br>All the predictors were measured after treatment			
		Dev	Val
2.1 Were predictors defined and assessed in a similar way for all participants?		PY	PY
2.2 Were predictor assessments made without knowledge of outcome data?		PY	PY
2.3 Are all predictors available at the time the model is intended to be used?		PY	PY
**Risk of bias introduced by predictors or their assessment**	**RISK:**<br>*(low/ high/ unclear)*	Low	Low
Rationale of bias rating:<br>*The blinding of measurement is unknown but all the predictors are objective.*			
**B. Applicability**			
Concern that the definition, assessment or timing of predictors in the model do not match the review question	**CONCERN:**<br>*(low/ high/ unclear)*	Low	Low
Rationale of applicability rating:<br>*No major issues identified.*			

DOMAIN 3: Outcome			
**A. Risk of Bias**			
Describe the outcome, how it was defined and determined, and the time interval between predictor assessment and outcome determination:<br>The outcome was cancer specific survival 5 years			
		Dev	Val
3.1 Was the outcome determined appropriately?		PY	PY
3.2 Was a pre-specified or standard outcome definition used?		Y	Y
3.3 Were predictors excluded from the outcome definition?		PY	PY
3.4 Was the outcome defined and determined in a similar way for all participants?		PY	PY
3.5 Was the outcome determined without knowledge of predictor information?		PY	PY
3.6 Was the time interval between predictor assessment and outcome determination appropriate?		Y	Y
**Risk of bias introduced by the outcome or its determination**	**RISK:**<br>*(low/ high/ unclear)*	Low	Low
Rationale of bias rating:<br>*No major issues identified*			
**B. Applicability**			
At what time point was the outcome determined:<br>5 years<br><br>*If a composite outcome was used, describe the relative frequency/distribution of each contributing outcome:*<br>*N/A*			
**Concern that the outcome, its definition, timing or determination do not match the review question**	**CONCERN:**<br>*(low/ high/ unclear)*	Low	Low
Rationale of applicability rating:<br>*The outcome of the primary study matches the outcome of interest of the review*			

**DOMAIN 4: Analysis**
**Risk of Bias**
Describe numbers of participants, number of candidate predictors, outcome events and events per candidate predictor:<br>*N= 258 (E= unknown) to develop the mode*<br>*N= 177 (E= unknown) to validate the model*<br>*The total number of events is 77. They didn´t say how many in the development or validation cohort occurred.*<br>*EPV= unknown*<br><br>*A total of 12 predictors were included (Table 2)*
Describe how the model was developed (for example in regards to modelling technique (e.g. survival or logistic modelling), predictor selection, and risk group definition):<br><br>*"Univariate and multivariate Cox proportional hazards regression models addressed the effect of covariates on DSS. For the final nomogram predicting 5-year DSS a backward selection process (inclusion vs exclusion criteria p ≤0.05 vs >0.10) was done"*
Describe whether and how the model was validated, either internally (e.g. bootstrapping, cross validation, random split sample) or externally (e.g. temporal validation, geographical validation, different setting, different type of participants):<br><br>*"The final model containing only significant co- variates was used to create the nomogram, which was internally validated on the development cohort by boot- strapping (200 repeat samples)"*<br>*"External validation was done in the consecutive cohort of 177 patients treated at Medical University of Graz. DSS probability was calculated with the nomogram formula derived from the development cohort"*
Describe the performance measures of the model, e.g. (re)calibration, discrimination, (re)classification, net benefit, and whether they were adjusted for optimism:<br>*"Calibration plots of the internally validated nomogram were used to assess its performance at 5 years. Predictive accuracy was assessed by the C-index"*
Describe any participants who were excluded from the analysis:<br>*"Patients with renal tumors of other subtypes, those who presented with bilateral, familial or hereditary RCC, 10 who did not undergo nephrectomy as part of cancer treatment, 13 with positive surgical margins on pathology assessment and 130 with no slides available for review were excluded"*
Describe missing data on predictors and outcomes as well as methods used for missing data:<br>*"Patients with renal tumors of other subtypes, those who presented with bilateral, familial or hereditary RCC, 10 who did not undergo nephrectomy as part of cancer treatment, 13 with positive surgical margins on pathology assessment and 130 with no slides available for review were excluded"*

	Dev	Val
4.1 Were there a reasonable number of participants with the outcome?	PN	PN
4.2 Were continuous and categorical predictors handled appropriately?	Y	Y
4.3 Were all enrolled participants included in the analysis?	N	N
4.4 Were participants with missing data handled appropriately?	N	N
4.5 Was selection of predictors based on univariable analysis avoided?	Y	

4.6 Were complexities in the data (e.g. censoring, competing risks, sampling of controls) accounted for appropriately?			N	N
4.7 Were relevant model performance measures evaluated appropriately?			Y	Y
4.8 Were model overfitting and optimism in model performance accounted for?			Y	
4.9 Do predictors and their assigned weights in the final model correspond to the results from multivariable analysis?			PY	
**Risk of bias introduced by the analysis**	**RISK:** *(low/ high/ unclear)*		High	High
Rationale of bias rating: *They didn´t say the number of events in each cohort. The total number of events were 77, because of this we think that there is not a reasonable number of patients in the validation cohort. They excluded patients with missing data. They didn't use competing risks.*				

Step 4: Overall assessment

Use the following tables to reach overall judgements about risk of bias and concerns regarding applicability of the prediction model evaluation (development and/or validation) across all assessed domains.
Complete for each evaluation of a distinct model.

Reaching an overall judgement about risk of bias of the prediction model evaluation	
**Low risk of bias**	If all domains were rated low risk of bias.<br>If a prediction model was developed without any external validation, and it was rated as low risk of bias for all domains, consider downgrading to **high risk of bias**. Such a model can only be considered as low risk of bias, if the development was based on a very large data set and included some form of internal validation.
**High risk of bias**	If at least one domain is judged to be at **high risk of bias**.
**Unclear risk of bias**	If an unclear risk of bias was noted in at least one domain and it was low risk for all other domains.

Reaching an overall judgement about applicability of the prediction model evaluation	
**Low concerns regarding applicability**	If low concerns regarding applicability for all domains, the prediction model evaluation is judged to have **low concerns regarding applicability**.
**High concerns regarding applicability**	If high concerns regarding applicability for at least one domain, the prediction model evaluation is judged to have **high concerns regarding applicability**.
**Unclear concerns regarding applicability**	If unclear concerns (but no "high concern") regarding applicability for at least one domain, the prediction model evaluation is judged to have **unclear concerns regarding applicability** overall.

Overall judgement about risk of bias and applicability of the prediction model evaluation		
**Overall judgement of risk of bias**	**RISK:**<br>*(low/ high/ unclear)*	High
Summary of sources of potential bias:<br>Analysis and participants domains show several major issues		
**Overall judgement of applicability**	**CONCERN:**<br>*(low/ high/ unclear)*	High
Summary of applicability concerns:<br><br>Participants domain shows several major issues		

PROBAST

(Prediction model study Risk Of Bias Assessment Tool)

Published in Annals of Internal Medicine (freely available):

1. PROBAST: A Tool to Assess the Risk of Bias and Applicability of Prediction Model Studies
2. PROBAST: A Tool to Assess Risk of Bias and Applicability of Prediction Model Studies: Explanation and Elaboration

What does PROBAST assess?

PROBAST assesses both the *risk of bias* and *concerns regarding applicability* of a study that evaluates (develops, validates or updates) a multivariable diagnostic or prognostic prediction model. It is designed to assess primary studies included in a systematic review.

Bias occurs if systematic flaws or limitations in the design, conduct or analysis of a primary study distort the results. For the purpose of prediction modelling studies, we have defined *risk of bias* to occur when shortcomings in the study design, conduct or analysis lead to systematically distorted estimates of a model's predictive performance or to an inadequate model to address the research question. Model predictive performance is typically evaluated using calibration, discrimination and sometimes classification measures, and these are likely inaccurately estimated in studies with high risk of bias. *Applicability* refers to the extent to which the prediction model from the primary study matches your systematic review question, for example in terms of the participants, predictors or outcome of interest.

A primary study may include the development and/or validation or update of more than one prediction model. A PROBAST assessment should be completed for each distinct model that is developed, validated or updated (extended) for making individualised predictions. Where a publication assesses multiple prediction models, only complete a PROBAST assessment for those models that meet the inclusion criteria for your systematic review. Please note that subsequent use of the term "model" includes derivatives of models, such as simplified risk scores, nomograms, or recalibrations of models.

PROBAST is not designed for all multivariable diagnostic or prognostic studies. For example, studies using multivariable models to identify predictors associated with an outcome but not attempting to develop a model for making individualised predictions are not covered by PROBAST.

PROBAST includes four steps.

Step	Task	When to complete
**1**	Specify your systematic review question(s)	Once per systematic review
**2**	Classify the type of prediction model evaluation	Once for each model of interest in each publication being assessed, for each relevant outcome
**3**	Assess risk of bias and applicability	Once for each development and validation of each distinct prediction model in a publication
**4**	Overall judgment	Once for each development and validation of each distinct prediction model in a publication

If this is your first time using PROBAST, we strongly recommend reading the detailed explanation and elaboration (E&E, see link above) paper and to check the examples on www.probast.org

Step 1: Specify your systematic review question

State your systematic review question to facilitate the assessment of the applicability of the evaluated models to your question. *The following table should be completed once per systematic review.*

Criteria	Specify your systematic review question
Intended use of model:	*To predict cancer specific mortality in patients with renal cancer treated with partial or total nephrectomy regardless of TNM stage*
***Participants** including selection criteria and setting:*	*Patients with renal cancer treated with partial or total nephrectomy regardless of TNM stage*
***Predictors** (used in prediction modelling), including types of predictors (e.g. history, clinical examination, biochemical markers, imaging tests), time of measurement, specific measurement issues (e.g., any requirements/ prohibitions for specialized equipment):*	*Predictors used in clinical practice measured when a nephrectomy for renal cancer is indicated*
Outcome to be predicted:	*Cancer specific mortality*

Step 2: Classify the type of prediction model evaluation

Use the following table to classify the evaluation as model development, model validation or model update, or combination. Different signalling questions apply for different types of prediction model evaluation. If the evaluation does not fit one of these classifications then PROBAST should not be used.

Classify the evaluation based on its aim			
**Type of prediction study**	**PROBAST boxes to complete**	**Tick as appropriate**	**Definition for type of prediction model study**
Development only	Development	✓	Prediction model development without external validation. These studies may include internal validation methods, such as bootstrapping and cross-validation techniques.
Development and validation	Development and validation	✗	Prediction model development combined with external validation in other participants in the same article.
Validation only	Validation	✗	External validation of existing (previously developed) model in other participants.

This table should be completed once for each publication being assessed and for each relevant outcome in your review.

**Publication reference**	Velis JM, Ancizu FJ, Hevia M, Merino I, Garcia A, Domenech P, Algarra R, Tienza A, Pascual JI, Robles JE. Modelos de riesgo en pacientes con carcinoma de células renales localizado. Act Urol Esp. 2017;41(9):564-570.
**Models of interest**	Risk score
**Outcome of interest**	*Cancer specific mortality*

Step 3: Assess risk of bias and applicability

PROBAST is structured as four key domains. Each domain is judged for risk of bias (low, high or unclear) and includes signalling questions to help make judgements. Signalling questions are rated as yes (Y), probably yes (PY), probably no (PN), no (N) or no information (NI). All signalling questions are phrased so that "yes" indicates absence of bias. Any signalling question rated as "no" or "probably no" flags the potential for bias; you will need to use your judgement to determine whether the domain should be rated as "high", "low" or "unclear" risk of bias. The guidance document contains further instructions and examples on rating signalling questions and risk of bias for each domain.

The first three domains are also rated for concerns regarding applicability (low/ high/ unclear) to your review question defined above.

Complete all domains separately for each evaluation of a distinct model. Shaded boxes indicate where signalling questions do not apply and should not be answered.

DOMAIN 1: Participants			
**A. Risk of Bias**			
Describe the sources of data and criteria for participant selection:<br><br>Patients diagnosed with renal cell carcinoma treated with radical or partial nephrectomy, without nodal or distant metastasis			
		Dev	Val
1.1 Were appropriate data sources used, e.g. cohort, RCT or nested case-control study data?		Y	N/A
1.2 Were all inclusions and exclusions of participants appropriate?		N	N/A
**Risk of bias introduced by selection of participants**	**RISK:**<br>*(low/ high/ unclear)*	High	N/A
Rationale of bias rating:<br>*The study excluded patients with metastasis*			
**B. Applicability**			
Describe included participants, setting and dates:<br><br>Patients diagnosed with renal cell carcinoma treated with radical or partial nephrectomy, without nodal or distant metastasis			
**Concern that the included participants and setting do not match the review question**	**CONCERN:**<br>*(low/ high/ unclear)*	High	N/A
Rationale of applicability rating:<br>*The study excluded patients with metastasis*			

DOMAIN 2: Predictors			
**A. Risk of Bias**			
List and describe predictors included in the final model, e.g. definition and timing of assessment:<br><br>The predictors included in the model were: tumor grade, perirenal fat invasion and tumor necrosis<br><br>All the predictors were measured after treatment			
		Dev	Val
2.1 Were predictors defined and assessed in a similar way for all participants?		PY	N/A
2.2 Were predictor assessments made without knowledge of outcome data?		PY	N/A
2.3 Are all predictors available at the time the model is intended to be used?		PY	N/A
**Risk of bias introduced by predictors or their assessment**	**RISK:**<br>*(low/ high/ unclear)*	Low	N/A
Rationale of bias rating:<br>*The blinding of measurement is unknown but all the predictors are objective.*			
**B. Applicability**			
Concern that the definition, assessment or timing of predictors in the model do not match the review question	**CONCERN:**<br>*(low/ high/ unclear)*	Low	N/A
Rationale of applicability rating:<br>*No major issues identified.*			

DOMAIN 3: Outcome

A. Risk of Bias

Describe the outcome, how it was defined and determined, and the time interval between predictor assessment and outcome determination:

The outcome was cancer specific survival at 5 and 10- years

		Dev	Val
3.1 Was the outcome determined appropriately?		PY	N/A
3.2 Was a pre-specified or standard outcome definition used?		Y	N/A
3.3 Were predictors excluded from the outcome definition?		PY	N/A
3.4 Was the outcome defined and determined in a similar way for all participants?		PY	N/A
3.5 Was the outcome determined without knowledge of predictor information?		PY	N/A
3.6 Was the time interval between predictor assessment and outcome determination appropriate?		Y	N/A
**Risk of bias introduced by the outcome or its determination**	**RISK:** *(low/ high/ unclear)*	Low	N/A

Rationale of bias rating:

No major issues identified

B. Applicability

At what time point was the outcome determined:

5 and 10 years

If a composite outcome was used, describe the relative frequency/distribution of each contributing outcome:

N/A

**Concern that the outcome, its definition, timing or determination do not match the review question**	**CONCERN:** *(low/ high/ unclear)*	Low	N/A

Rationale of applicability rating:

The outcome of the primary study matches the outcome of interest of the review

DOMAIN 4: Analysis			
**Risk of Bias**			
Describe numbers of participants, number of candidate predictors, outcome events and events per candidate predictor:<br><br>596 patients with renal cell carcinoma were included in the study between 1990 and 2012 in one center.<br><br>The total number of predictors is 12 (Table 6)<br>The number of events was 57<br><br>EPV= 57/12= 4.25			
Describe how the model was developed (for example in regards to modelling technique (e.g. survival or logistic modelling), predictor selection, and risk group definition):<br>They did a cox regression (univariate and multivariate)			
Describe whether and how the model was validated, either internally (e.g. bootstrapping, cross validation, random split sample) or externally (e.g. temporal validation, geographical validation, different setting, different type of participants):<br><br>*They didn´t do any validation*			
Describe the performance measures of the model, e.g. (re)calibration, discrimination, (re)classification, net benefit, and whether they were adjusted for optimism:<br><br>*They didn´t do calibration or discrimination*			
Describe any participants who were excluded from the analysis:<br>*They excluded patients with nodal or distant metastasis*			
Describe missing data on predictors and outcomes as well as methods used for missing data:<br>*Not indicated*			
		Dev	Val
4.1 Were there a reasonable number of participants with the outcome?		N	N/A
4.2 Were continuous and categorical predictors handled appropriately?		N	N/A
4.3 Were all enrolled participants included in the analysis?		NI	N/A
4.4 Were participants with missing data handled appropriately?		NI	N/A
4.5 Was selection of predictors based on univariable analysis avoided?		N	
4.6 Were complexities in the data (e.g. censoring, competing risks, sampling of controls) accounted for appropriately?		N	N/A
4.7 Were relevant model performance measures evaluated appropriately?		N	N/A
4.8 Were model overfitting and optimism in model performance accounted for?		N	
4.9 Do predictors and their assigned weights in the final model correspond to the results from multivariable analysis?		PY	
**Risk of bias introduced by the analysis**	**RISK:**<br>*(low/ high/ unclear)*	High	N/A
Rationale of bias rating:<br>They didn´t have enough patients with the outcome. They did categorizations. They didn´t say anything about missing data. They selected the predictors based on univariable. They didn´t do calibration,			

discrimination or validation. They didn't use competing risks.

Step 4: Overall assessment

Use the following tables to reach overall judgements about risk of bias and concerns regarding applicability of the prediction model evaluation (development and/or validation) across all assessed domains.
Complete for each evaluation of a distinct model.

**Reaching an overall judgement about risk of bias of the prediction model evaluation**	
**Low risk of bias**	If all domains were rated low risk of bias.<br>If a prediction model was developed without any external validation, and it was rated as low risk of bias for all domains, consider downgrading to **high risk of bias**. Such a model can only be considered as low risk of bias, if the development was based on a very large data set and included some form of internal validation.
**High risk of bias**	If at least one domain is judged to be at **high risk of bias**.
**Unclear risk of bias**	If an unclear risk of bias was noted in at least one domain and it was low risk for all other domains.

**Reaching an overall judgement about applicability of the prediction model evaluation**	
**Low concerns regarding applicability**	If low concerns regarding applicability for all domains, the prediction model evaluation is judged to have **low concerns regarding applicability**.
**High concerns regarding applicability**	If high concerns regarding applicability for at least one domain, the prediction model evaluation is judged to have **high concerns regarding applicability**.
**Unclear concerns regarding applicability**	If unclear concerns (but no "high concern") regarding applicability for at least one domain, the prediction model evaluation is judged to have **unclear concerns regarding applicability** overall.

**Overall judgement about risk of bias and applicability of the prediction model evaluation**		
**Overall judgement of risk of bias**	**RISK:**<br>*(low/ high/ unclear)*	High
Summary of sources of potential bias:<br>Analysis and participants domains show several major issues		
**Overall judgement of applicability**	**CONCERN:**<br>*(low/ high/ unclear)*	High
Summary of applicability concerns:<br><br>Participants domain shows several major issues		

PROBAST

(Prediction model study Risk Of Bias Assessment Tool)

Published in Annals of Internal Medicine (freely available):

1. PROBAST: A Tool to Assess the Risk of Bias and Applicability of Prediction Model Studies
2. PROBAST: A Tool to Assess Risk of Bias and Applicability of Prediction Model Studies: Explanation and Elaboration

What does PROBAST assess?

PROBAST assesses both the *risk of bias* and *concerns regarding applicability* of a study that evaluates (develops, validates or updates) a multivariable diagnostic or prognostic prediction model. It is designed to assess primary studies included in a systematic review.

Bias occurs if systematic flaws or limitations in the design, conduct or analysis of a primary study distort the results. For the purpose of prediction modelling studies, we have defined *risk of bias* to occur when shortcomings in the study design, conduct or analysis lead to systematically distorted estimates of a model's predictive performance or to an inadequate model to address the research question. Model predictive performance is typically evaluated using calibration, discrimination and sometimes classification measures, and these are likely inaccurately estimated in studies with high risk of bias. *Applicability* refers to the extent to which the prediction model from the primary study matches your systematic review question, for example in terms of the participants, predictors or outcome of interest.

A primary study may include the development and/or validation or update of more than one prediction model. A PROBAST assessment should be completed for each distinct model that is developed, validated or updated (extended) for making individualised predictions. Where a publication assesses multiple prediction models, only complete a PROBAST assessment for those models that meet the inclusion criteria for your systematic review. Please note that subsequent use of the term "model" includes derivatives of models, such as simplified risk scores, nomograms, or recalibrations of models.

PROBAST is not designed for all multivariable diagnostic or prognostic studies. For example, studies using multivariable models to identify predictors associated with an outcome but not attempting to develop a model for making individualised predictions are not covered by PROBAST.

PROBAST includes four steps.

Step	Task	When to complete
**1**	Specify your systematic review question(s)	Once per systematic review
**2**	Classify the type of prediction model evaluation	Once for each model of interest in each publication being assessed, for each relevant outcome
**3**	Assess risk of bias and applicability	Once for each development and validation of each distinct prediction model in a publication
**4**	Overall judgment	Once for each development and validation of each distinct prediction model in a publication

If this is your first time using PROBAST, we strongly recommend reading the detailed explanation and elaboration (E&E, see link above) paper and to check the examples on www.probast.org

Step 1: Specify your systematic review question

State your systematic review question to facilitate the assessment of the applicability of the evaluated models to your question. *The following table should be completed once per systematic review.*

Criteria	Specify your systematic review question
Intended use of model:	*To predict cancer specific mortality in patients with renal cancer treated with partial or total nephrectomy regardless of TNM stage*
***Participants** including selection criteria and setting:*	*Patients with renal cancer treated with partial or total nephrectomy regardless of TNM stage*
***Predictors** (used in prediction modelling), including types of predictors (e.g. history, clinical examination, biochemical markers, imaging tests), time of measurement, specific measurement issues (e.g., any requirements/ prohibitions for specialized equipment):*	*Predictors used in clinical practice measured when a nephrectomy for renal cancer is indicated*
Outcome to be predicted:	*Cancer specific mortality*

Step 2: Classify the type of prediction model evaluation

Use the following table to classify the evaluation as model development, model validation or model update, or combination. Different signalling questions apply for different types of prediction model evaluation. If the evaluation does not fit one of these classifications then PROBAST should not be used.

**Classify the evaluation based on its aim**			
**Type of prediction study**	**PROBAST boxes to complete**	**Tick as appropriate**	**Definition for type of prediction model study**
Development only	Development	✓	Prediction model development without external validation. These studies may include internal validation methods, such as bootstrapping and cross-validation techniques.
Development and validation	Development and validation	✗	Prediction model development combined with external validation in other participants in the same article.
Validation only	Validation	✗	External validation of existing (previously developed) model in other participants.

This table should be completed once for each publication being assessed and for each relevant outcome in your review.

**Publication reference**	Peng D, He ZS, Li XS, Tang Q, Zhang L, Yang KW, Yu XT, Zhang CJ, Zhou LQ. A Novel Predictor of Survival with Renal Cell Carcinoma After Nephrectomy. J Endourol. 2017;31(4):397-404
**Models of interest**	Risk score
**Outcome of interest**	*Cancer specific mortality*

Step 3: Assess risk of bias and applicability

PROBAST is structured as four key domains. Each domain is judged for risk of bias (low, high or unclear) and includes signalling questions to help make judgements. Signalling questions are rated as yes (Y), probably yes (PY), probably no (PN), no (N) or no information (NI). All signalling questions are phrased so that “yes” indicates absence of bias. Any signalling question rated as “no” or “probably no” flags the potential for bias; you will need to use your judgement to determine whether the domain should be rated as “high”, “low” or “unclear” risk of bias. The guidance document contains further instructions and examples on rating signalling questions and risk of bias for each domain.

The first three domains are also rated for concerns regarding applicability (low/ high/ unclear) to your review question defined above.

Complete all domains separately for each evaluation of a distinct model. Shaded boxes indicate where signalling questions do not apply and should not be answered.

<table>
<tr><th colspan="4">DOMAIN 1: Participants</th></tr>
<tr><th colspan="4">A. Risk of Bias</th></tr>
<tr><td colspan="4">Describe the sources of data and criteria for participant selection:

“The medical records of 1360 RCC patients who underwent nephrectomy in Peking University First Hospital were retrospectively collected”</td></tr>
<tr><td colspan="2"></td><td>Dev</td><td>Val</td></tr>
<tr><td colspan="2">1.1 Were appropriate data sources used, e.g. cohort, RCT or nested case-control study data?</td><td>Y</td><td>N/A</td></tr>
<tr><td colspan="2">1.2 Were all inclusions and exclusions of participants appropriate?</td><td>Y</td><td>N/A</td></tr>
<tr><td>Risk of bias introduced by selection of participants</td><td>RISK:
(low/ high/ unclear)</td><td>Low</td><td>N/A</td></tr>
<tr><td colspan="4">Rationale of bias rating:
Cohort study with clear inclusion and exclusion criterial</td></tr>
<tr><th colspan="4">B. Applicability</th></tr>
<tr><td colspan="4">Describe included participants, setting and dates:
“The medical records of 1360 RCC patients who underwent nephrectomy in Peking University First Hospital were retrospectively collected”</td></tr>
<tr><td>Concern that the included participants and setting do not match the review question</td><td>CONCERN:
(low/ high/ unclear)</td><td>Low</td><td>N/A</td></tr>
<tr><td colspan="4">Rationale of applicability rating:
Cohort study with clear inclusion and exclusion criterial</td></tr>
</table>

DOMAIN 2: Predictors			
**A. Risk of Bias**			
List and describe predictors included in the final model, e.g. definition and timing of assessment:<br><br>The predictors included in the model were: fibrinogen, cholesterol and fibrinogen-cholesterol score<br><br>All the predictors were measured at diagnosis			
		Dev	Val
2.1 Were predictors defined and assessed in a similar way for all participants?		PY	N/A
2.2 Were predictor assessments made without knowledge of outcome data?		PY	N/A
2.3 Are all predictors available at the time the model is intended to be used?		PY	N/A
**Risk of bias introduced by predictors or their assessment**	**RISK:**<br>*(low/ high/ unclear)*	Low	N/A
Rationale of bias rating:<br>*The blinding of measurement is unknown but all the predictors are objective.*			
**B. Applicability**			
Concern that the definition, assessment or timing of predictors in the model do not match the review question	**CONCERN:**<br>*(low/ high/ unclear)*	Low	N/A
Rationale of applicability rating:<br>*No major issues identified.*			

DOMAIN 3: Outcome			
**A. Risk of Bias**			
Describe the outcome, how it was defined and determined, and the time interval between predictor assessment and outcome determination:<br>The outcome was cancer specific survival at 5 years			
		Dev	Val
3.1 Was the outcome determined appropriately?		PY	N/A
3.2 Was a pre-specified or standard outcome definition used?		Y	N/A
3.3 Were predictors excluded from the outcome definition?		PY	N/A
3.4 Was the outcome defined and determined in a similar way for all participants?		PY	N/A
3.5 Was the outcome determined without knowledge of predictor information?		PY	N/A
3.6 Was the time interval between predictor assessment and outcome determination appropriate?		Y	N/A
**Risk of bias introduced by the outcome or its determination**	**RISK:**<br>*(low/ high/ unclear)*	Low	N/A
Rationale of bias rating:<br>*No major issues identified*			
**B. Applicability**			
At what time point was the outcome determined:<br>5 years<br><br>*If a composite outcome was used, describe the relative frequency/distribution of each contributing outcome:*<br>*N/A*			
**Concern that the outcome, its definition, timing or determination do not match the review question**	**CONCERN:**<br>*(low/ high/ unclear)*	Low	N/A
Rationale of applicability rating:<br>*The outcome of the primary study matches the outcome of interest of the review*			

**DOMAIN 4: Analysis**		
**Risk of Bias**		
Describe numbers of participants, number of candidate predictors, outcome events and events per candidate predictor:<br>"The clinicopathological characteristics of 1360 RCC patients are in Table 1. The median age was was 55 years (IQR 46-65) and 408 (30%) were female; 1228 (90.29%) had clear-cell RCC. The median fibrinogen level for all patients was 320 mg/dL (interquartile range [IQR] 274-384.25) and median serum cholesterol level was 168.98 mg/dL (IQR 147.33-192.96). The median follow-up was 67 months (IQR 36-74); 221 patients (16.3%) showed disease progression and 139 (10.2%) died due to RCC during follow-up"<br><br>The total number of predictors is 17 (table 4)<br>The number of events is 139<br><br>EPV= 139/17=8.17		
Describe how the model was developed (for example in regards to modelling technique (e.g. survival or logistic modelling), predictor selection, and risk group definition):<br><br>"Variables with significant difference (P < 0.05) on univariate analysis were included in a Cox proportional-hazards model for multivariate survival analyses, estimating hazard ratios (HRs) and their 95% CIs. For all tests, P < 0.05 was considered statistically significant"		
Describe whether and how the model was validated, either internally (e.g. bootstrapping, cross validation, random split sample) or externally (e.g. temporal validation, geographical validation, different setting, different type of participants):<br><br>Not indicated		
Describe the performance measures of the model, e.g. (re)calibration, discrimination, (re)classification, net benefit, and whether they were adjusted for optimism:<br><br>Not indicated		
Describe any participants who were excluded from the analysis:<br>Not indicated		
Describe missing data on predictors and outcomes as well as methods used for missing data:<br>Not indicated		
	Dev	Val
4.1 Were there a reasonable number of participants with the outcome?	N	N/A
4.2 Were continuous and categorical predictors handled appropriately?	N	N/A
4.3 Were all enrolled participants included in the analysis?	NI	N/A
4.4 Were participants with missing data handled appropriately?	NI	N/A
4.5 Was selection of predictors based on univariable analysis avoided?	N	
4.6 Were complexities in the data (e.g. censoring, competing risks, sampling of controls) accounted for appropriately?	N	N/A
4.7 Were relevant model performance measures evaluated appropriately?	N	N/A
4.8 Were model overfitting and optimism in model performance accounted for?	N	

<table>
<tr><td colspan="2">4.9 Do predictors and their assigned weights in the final model correspond to the results from multivariable analysis?</td><td>PY</td><td></td></tr>
<tr><td>Risk of bias introduced by the analysis</td><td>RISK:
(low/ high/ unclear)</td><td>High</td><td>N/A</td></tr>
<tr><td colspan="4">Rationale of bias rating:
They didn´t have enough patients with the outcome. They did categorizations. They didn´t say anything about missing data. They selected the predictors based on univariable. They didn´t do validation, calibration or discrimination. They didn’t use competing risks.</td></tr>
</table>

Step 4: Overall assessment

Use the following tables to reach overall judgements about risk of bias and concerns regarding applicability of the prediction model evaluation (development and/or validation) across all assessed domains.
Complete for each evaluation of a distinct model.

Reaching an overall judgement about risk of bias of the prediction model evaluation	
**Low risk of bias**	If all domains were rated low risk of bias.<br>If a prediction model was developed without any external validation, and it was rated as low risk of bias for all domains, consider downgrading to **high risk of bias**. Such a model can only be considered as low risk of bias, if the development was based on a very large data set and included some form of internal validation.
**High risk of bias**	If at least one domain is judged to be at **high risk of bias**.
**Unclear risk of bias**	If an unclear risk of bias was noted in at least one domain and it was low risk for all other domains.

Reaching an overall judgement about applicability of the prediction model evaluation	
**Low concerns regarding applicability**	If low concerns regarding applicability for all domains, the prediction model evaluation is judged to have **low concerns regarding applicability**.
**High concerns regarding applicability**	If high concerns regarding applicability for at least one domain, the prediction model evaluation is judged to have **high concerns regarding applicability**.
**Unclear concerns regarding applicability**	If unclear concerns (but no "high concern") regarding applicability for at least one domain, the prediction model evaluation is judged to have **unclear concerns regarding applicability** overall.

Overall judgement about risk of bias and applicability of the prediction model evaluation		
**Overall judgement of risk of bias**	**RISK:**<br>*(low/ high/ unclear)*	High
Summary of sources of potential bias:<br>Analysis domain shows several major issues		
**Overall judgement of applicability**	**CONCERN:**<br>*(low/ high/ unclear)*	Low
Summary of applicability concerns:<br><br>No several major issues		

PROBAST

(Prediction model study Risk Of Bias Assessment Tool)

Published in Annals of Internal Medicine (freely available):

1. PROBAST: A Tool to Assess the Risk of Bias and Applicability of Prediction Model Studies
2. PROBAST: A Tool to Assess Risk of Bias and Applicability of Prediction Model Studies: Explanation and Elaboration

What does PROBAST assess?

PROBAST assesses both the *risk of bias* and *concerns regarding applicability* of a study that evaluates (develops, validates or updates) a multivariable diagnostic or prognostic prediction model. It is designed to assess primary studies included in a systematic review.

Bias occurs if systematic flaws or limitations in the design, conduct or analysis of a primary study distort the results. For the purpose of prediction modelling studies, we have defined *risk of bias* to occur when shortcomings in the study design, conduct or analysis lead to systematically distorted estimates of a model's predictive performance or to an inadequate model to address the research question. Model predictive performance is typically evaluated using calibration, discrimination and sometimes classification measures, and these are likely inaccurately estimated in studies with high risk of bias. *Applicability* refers to the extent to which the prediction model from the primary study matches your systematic review question, for example in terms of the participants, predictors or outcome of interest.

A primary study may include the development and/or validation or update of more than one prediction model. A PROBAST assessment should be completed for each distinct model that is developed, validated or updated (extended) for making individualised predictions. Where a publication assesses multiple prediction models, only complete a PROBAST assessment for those models that meet the inclusion criteria for your systematic review. Please note that subsequent use of the term "model" includes derivatives of models, such as simplified risk scores, nomograms, or recalibrations of models.

PROBAST is not designed for all multivariable diagnostic or prognostic studies. For example, studies using multivariable models to identify predictors associated with an outcome but not attempting to develop a model for making individualised predictions are not covered by PROBAST.

PROBAST includes four steps.

Step	Task	When to complete
**1**	Specify your systematic review question(s)	Once per systematic review
**2**	Classify the type of prediction model evaluation	Once for each model of interest in each publication being assessed, for each relevant outcome
**3**	Assess risk of bias and applicability	Once for each development and validation of each distinct prediction model in a publication
**4**	Overall judgment	Once for each development and validation of each distinct prediction model in a publication

If this is your first time using PROBAST, we strongly recommend reading the detailed explanation and elaboration (E&E, see link above) paper and to check the examples on www.probast.org

Step 1: Specify your systematic review question

State your systematic review question to facilitate the assessment of the applicability of the evaluated models to your question. *The following table should be completed once per systematic review.*

Criteria	Specify your systematic review question
Intended use of model:	*To predict cancer specific mortality in patients with renal cancer treated with partial or total nephrectomy regardless of TNM stage*
***Participants** including selection criteria and setting:*	*Patients with renal cancer treated with partial or total nephrectomy regardless of TNM stage*
***Predictors** (used in prediction modelling), including types of predictors (e.g. history, clinical examination, biochemical markers, imaging tests), time of measurement, specific measurement issues (e.g., any requirements/ prohibitions for specialized equipment):*	*Predictors used in clinical practice measured when a nephrectomy for renal cancer is indicated*
Outcome to be predicted:	*Cancer specific mortality*

Step 2: Classify the type of prediction model evaluation

Use the following table to classify the evaluation as model development, model validation or model update, or combination. Different signalling questions apply for different types of prediction model evaluation. If the evaluation does not fit one of these classifications then PROBAST should not be used.

Classify the evaluation based on its aim			
**Type of prediction study**	**PROBAST boxes to complete**	**Tick as appropriate**	**Definition for type of prediction model study**
Development only	Development	✓	Prediction model development without external validation. These studies may include internal validation methods, such as bootstrapping and cross-validation techniques.
Development and validation	Development and validation	✗	Prediction model development combined with external validation in other participants in the same article.
Validation only	Validation	✗	External validation of existing (previously developed) model in other participants.

This table should be completed once for each publication being assessed and for each relevant outcome in your review.

**Publication reference**	Lyon TD, Gershman B, Shah PH, Thompson RH, Boorjian SA, Lohse CM, Costello BA, Cheville JC, Leibovich BC. Risk prediction models for cancer-specific survival following cytoreductive nephrectomy in the contemporary era. Urol Oncol. 2018; 36(11):499.e1-499.e7
**Models of interest**	Risk score
**Outcome of interest**	*Cancer specific mortality*

Step 3: Assess risk of bias and applicability

PROBAST is structured as four key domains. Each domain is judged for risk of bias (low, high or unclear) and includes signalling questions to help make judgements. Signalling questions are rated as yes (Y), probably yes (PY), probably no (PN), no (N) or no information (NI). All signalling questions are phrased so that "yes" indicates absence of bias. Any signalling question rated as "no" or "probably no" flags the potential for bias; you will need to use your judgement to determine whether the domain should be rated as "high", "low" or "unclear" risk of bias. The guidance document contains further instructions and examples on rating signalling questions and risk of bias for each domain.

The first three domains are also rated for concerns regarding applicability (low/ high/ unclear) to your review question defined above.

Complete all domains separately for each evaluation of a distinct model. Shaded boxes indicate where signalling questions do not apply and should not be answered.

DOMAIN 1: Participants			
**A. Risk of Bias**			
Describe the sources of data and criteria for participant selection:<br><br>"Following institutional review board approval, the Mayo Clinic Nephrectomy Registry was queried to identify 314 patients treated with radical or partial (n = 8) nephrectomy for sporadic, unilateral, M1 RCC between 1990 and 2010. One patient who died intraoperatively was excluded, leaving 313 patients for analysis"			
		Dev	Val
1.1 Were appropriate data sources used, e.g. cohort, RCT or nested case-control study data?		Y	N/A
1.2 Were all inclusions and exclusions of participants appropriate?		N	N/A
**Risk of bias introduced by selection of participants**	**RISK:**<br>*(low/ high/ unclear)*	High	N/A
Rationale of bias rating:<br>They excluded patients without metastasis.			
**B. Applicability**			
Describe included participants, setting and dates:<br>"Following institutional review board approval, the Mayo Clinic Nephrectomy Registry was queried to identify 314 patients treated with radical or partial (n = 8) nephrectomy for sporadic, unilateral, M1 RCC between 1990 and 2010. One patient who died intraoperatively was excluded, leaving 313 patients for analysis"			
**Concern that the included participants and setting do not match the review question**	**CONCERN:**<br>*(low/ high/ unclear)*	High	N/A
Rationale of applicability rating:<br>They excluded patients without metastasis.			

<table>
<tr><th colspan="4">DOMAIN 2: Predictors</th></tr>
<tr><th colspan="4">A. Risk of Bias</th></tr>
<tr><td colspan="4">List and describe predictors included in the final model, e.g. definition and timing of assessment:

The predictors included in the model were:
- Preoperative risk score: age, sex, constitutional symptoms, tumor thrombus, lymphadenopathy.
- Postoperative risk score: age, sex, constitutional symptoms, tumor thrombus, lymphadenopathy, coagulative necrosis and sarcomatoid differentiation</td></tr>
<tr><td colspan="2"></td><td>Dev</td><td>Val</td></tr>
<tr><td colspan="2">2.1 Were predictors defined and assessed in a similar way for all participants?</td><td>PY</td><td>N/A</td></tr>
<tr><td colspan="2">2.2 Were predictor assessments made without knowledge of outcome data?</td><td>PY</td><td>N/A</td></tr>
<tr><td colspan="2">2.3 Are all predictors available at the time the model is intended to be used?</td><td>PY</td><td>N/A</td></tr>
<tr><td>Risk of bias introduced by predictors or their assessment</td><td>RISK:
(low/ high/ unclear)</td><td>Low</td><td>N/A</td></tr>
<tr><td colspan="4">Rationale of bias rating:
“Pathologic specimens were reviewed by one urologic pathologist blinded to patient outcome”</td></tr>
<tr><th colspan="4">B. Applicability</th></tr>
<tr><td>Concern that the definition, assessment or timing of predictors in the model do not match the review question</td><td>CONCERN:
(low/ high/ unclear)</td><td>Low</td><td>N/A</td></tr>
<tr><td colspan="4">Rationale of applicability rating:
No major issues identified.</td></tr>
</table>

DOMAIN 3: Outcome			
**A. Risk of Bias**			
Describe the outcome, how it was defined and determined, and the time interval between predictor assessment and outcome determination:<br>The outcome was cancer specific survival at 1, 3 and 5 years<br><br>"If a patient has died, a death certificate is ordered to determine the cause of death" "If the death certificate does not support this, the medical history is reviewed by a urologist to determine the cause of death, a method that has also been employed by others [14]. If a death certificate cannot be obtained, cause of death is verified with the patient's family or local physician"			
		Dev	Val
3.1 Was the outcome determined appropriately?		PY	N/A
3.2 Was a pre-specified or standard outcome definition used?		Y	N/A
3.3 Were predictors excluded from the outcome definition?		PY	N/A
3.4 Was the outcome defined and determined in a similar way for all participants?		PY	N/A
3.5 Was the outcome determined without knowledge of predictor information?		PY	N/A
3.6 Was the time interval between predictor assessment and outcome determination appropriate?		Y	N/A
**Risk of bias introduced by the outcome or its determination**	**RISK:**<br>*(low/ high/ unclear)*	Low	N/A
Rationale of bias rating:<br>*No major issues identified*			
**B. Applicability**			
At what time point was the outcome determined:<br>1, 3 and 5 years<br><br>*If a composite outcome was used, describe the relative frequency/distribution of each contributing outcome:*<br>*N/A*			
**Concern that the outcome, its definition, timing or determination do not match the review question**	**CONCERN:**<br>*(low/ high/ unclear)*	Low	N/A
Rationale of applicability rating:<br>*The outcome of the primary study matches the outcome of interest of the review*			

DOMAIN 4: Analysis

Risk of Bias

Describe numbers of participants, number of candidate predictors, outcome events and events per candidate predictor:

"Following institutional review board approval, the Mayo Clinic Nephrectomy Registry was queried to identify 314 patients treated with radical or partial (n = 8) nephrectomy for sporadic, unilateral, M1 RCC between 1990 and 2010. One patient who died intraoperatively was excluded, leaving 313 patients for analysis"

The total number of predictors is 46 (supplementary table 1)
The number of events was 279
EPV= 279/46=5.06

Describe how the model was developed (for example in regards to modelling technique (e.g. survival or logistic modelling), predictor selection, and risk group definition):

"Descriptive statistics are presented as counts and percen- tages for categorical features, and median and interquartile range (IQR) for continuous features. Associations with cancer-specific mortality (CSM) were evaluated using Cox proportional hazards regression models. Five patients who died from unknown causes were excluded from these analyses. Multivariable models were built using stepwise selec- tion with the P value for a feature to enter the model set to 0.05. One model was limited to features known preoperatively, while a second model included all features"

Describe whether and how the model was validated, either internally (e.g. bootstrapping, cross validation, random split sample) or externally (e.g. temporal validation, geographical validation, different setting, different type of participants):

"Features included in the resulting multivariable model were subjected to bootstrap resampling with replacement 500 times. A multivariable model was developed for each of the 500 samples using stepwise selection, and only those features that entered 70% or more of the models were retained"

Describe the performance measures of the model, e.g. (re)calibration, discrimination, (re)classification, net benefit, and whether they were adjusted for optimism:

"Model performance was assessed with bootstrap-corrected c-indexes and calibration plots. Clinical utility of the preoperative model was assessed using decision curve analysis, plotting predicted probabilities of CSM at 1 year by the net benefit, derived from the true positive and false positive rates for a given threshold"

Describe any participants who were excluded from the analysis:

"One patient who died intraoperatively was excluded, leaving 313 patients for analysis"

Describe missing data on predictors and outcomes as well as methods used for missing data:

Not indicated

	Dev	Val
4.1 Were there a reasonable number of participants with the outcome?	N	N/A
4.2 Were continuous and categorical predictors handled appropriately?	N	N/A
4.3 Were all enrolled participants included in the analysis?	Y	N/A
4.4 Were participants with missing data handled appropriately?	NI	N/A
4.5 Was selection of predictors based on univariable analysis avoided?	N	
4.6 Were complexities in the data (e.g. censoring, competing risks, sampling of controls) accounted for appropriately?	N	N/A
4.7 Were relevant model performance measures evaluated appropriately?	Y	N/A
4.8 Were model overfitting and optimism in model performance accounted for?	Y	
4.9 Do predictors and their assigned weights in the final model correspond to the results from multivariable analysis?	PY	
**Risk of bias introduced by the analysis** — **RISK:** *(low/ high/ unclear)*	High	N/A

Rationale of bias rating:
They didn´t have enough patients with the outcome. They did categorizations. They didn´t say anything about with missing data. They selected the predictors based on univariable. They didn´t do competing risk.

Step 4: Overall assessment

Use the following tables to reach overall judgements about risk of bias and concerns regarding applicability of the prediction model evaluation (development and/or validation) across all assessed domains.
Complete for each evaluation of a distinct model.

**Reaching an overall judgement about risk of bias of the prediction model evaluation**	
**Low risk of bias**	If all domains were rated low risk of bias.<br>If a prediction model was developed without any external validation, and it was rated as low risk of bias for all domains, consider downgrading to **high risk of bias**. Such a model can only be considered as low risk of bias, if the development was based on a very large data set and included some form of internal validation.
**High risk of bias**	If at least one domain is judged to be at **high risk of bias**.
**Unclear risk of bias**	If an unclear risk of bias was noted in at least one domain and it was low risk for all other domains.

**Reaching an overall judgement about applicability of the prediction model evaluation**	
**Low concerns regarding applicability**	If low concerns regarding applicability for all domains, the prediction model evaluation is judged to have **low concerns regarding applicability**.
**High concerns regarding applicability**	If high concerns regarding applicability for at least one domain, the prediction model evaluation is judged to have **high concerns regarding applicability**.
**Unclear concerns regarding applicability**	If unclear concerns (but no "high concern") regarding applicability for at least one domain, the prediction model evaluation is judged to have **unclear concerns regarding applicability** overall.

**Overall judgement about risk of bias and applicability of the prediction model evaluation**		
**Overall judgement of risk of bias**	**RISK:**<br>*(low/ high/ unclear)*	High
Summary of sources of potential bias:<br>Analysis and participants domains show several major issues		
**Overall judgement of applicability**	**CONCERN:**<br>*(low/ high/ unclear)*	High
Summary of applicability concerns:<br><br>Participants domain show so several major issues		

PROBAST

(Prediction model study Risk Of Bias Assessment Tool)

Published in Annals of Internal Medicine (freely available):

1. PROBAST: A Tool to Assess the Risk of Bias and Applicability of Prediction Model Studies
2. PROBAST: A Tool to Assess Risk of Bias and Applicability of Prediction Model Studies: Explanation and Elaboration

What does PROBAST assess?

PROBAST assesses both the *risk of bias* and *concerns regarding applicability* of a study that evaluates (develops, validates or updates) a multivariable diagnostic or prognostic prediction model. It is designed to assess primary studies included in a systematic review.

Bias occurs if systematic flaws or limitations in the design, conduct or analysis of a primary study distort the results. For the purpose of prediction modelling studies, we have defined *risk of bias* to occur when shortcomings in the study design, conduct or analysis lead to systematically distorted estimates of a model's predictive performance or to an inadequate model to address the research question. Model predictive performance is typically evaluated using calibration, discrimination and sometimes classification measures, and these are likely inaccurately estimated in studies with high risk of bias. *Applicability* refers to the extent to which the prediction model from the primary study matches your systematic review question, for example in terms of the participants, predictors or outcome of interest.

A primary study may include the development and/or validation or update of more than one prediction model. A PROBAST assessment should be completed for each distinct model that is developed, validated or updated (extended) for making individualised predictions. Where a publication assesses multiple prediction models, only complete a PROBAST assessment for those models that meet the inclusion criteria for your systematic review. Please note that subsequent use of the term "model" includes derivatives of models, such as simplified risk scores, nomograms, or recalibrations of models.

PROBAST is not designed for all multivariable diagnostic or prognostic studies. For example, studies using multivariable models to identify predictors associated with an outcome but not attempting to develop a model for making individualised predictions are not covered by PROBAST.

PROBAST includes four steps.

Step	Task	When to complete
1	Specify your systematic review question(s)	Once per systematic review
2	Classify the type of prediction model evaluation	Once for each model of interest in each publication being assessed, for each relevant outcome
3	Assess risk of bias and applicability	Once for each development and validation of each distinct prediction model in a publication
4	Overall judgment	Once for each development and validation of each distinct prediction model in a publication

If this is your first time using PROBAST, we strongly recommend reading the detailed explanation and elaboration (E&E, see link above) paper and to check the examples on www.probast.org

Step 1: Specify your systematic review question

State your systematic review question to facilitate the assessment of the applicability of the evaluated models to your question. *The following table should be completed once per systematic review.*

Criteria	Specify your systematic review question
Intended use of model:	*To predict cancer specific mortality in patients with renal cancer treated with partial or total nephrectomy regardless of TNM stage*
***Participants** including selection criteria and setting:*	*Patients with renal cancer treated with partial or total nephrectomy regardless of TNM stage*
***Predictors** (used in prediction modelling), including types of predictors (e.g. history, clinical examination, biochemical markers, imaging tests), time of measurement, specific measurement issues (e.g., any requirements/ prohibitions for specialized equipment):*	*Predictors used in clinical practice measured when a nephrectomy for renal cancer is indicated*
Outcome to be predicted:	*Cancer specific mortality*

Step 2: Classify the type of prediction model evaluation

Use the following table to classify the evaluation as model development, model validation or model update, or combination. Different signalling questions apply for different types of prediction model evaluation. If the evaluation does not fit one of these classifications then PROBAST should not be used.

**Classify the evaluation based on its aim**			
**Type of prediction study**	**PROBAST boxes to complete**	**Tick as appropriate**	**Definition for type of prediction model study**
Development only	Development	✓	Prediction model development without external validation. These studies may include internal validation methods, such as bootstrapping and cross-validation techniques.
Development and validation	Development and validation	✗	Prediction model development combined with external validation in other participants in the same article.
Validation only	Validation	✗	External validation of existing (previously developed) model in other participants.

This table should be completed once for each publication being assessed and for each relevant outcome in your review.

**Publication reference**	Hsiao W, Herrel LA, Yu C, Kattan M, Canter DJ, Carthon BC, Ogan K, Master VA. Nomograms incorporating serum C-reactive protein effectively predict mortality before and after surgical treatment of renal cell carcinoma. Int J Urol. 2015 Mar;22(3):264-70. doi: 10.1111/iju.12672.
**Models of interest**	Nomogram
**Outcome of interest**	*Cancer specific mortality*

Step 3: Assess risk of bias and applicability

PROBAST is structured as four key domains. Each domain is judged for risk of bias (low, high or unclear) and includes signalling questions to help make judgements. Signalling questions are rated as yes (Y), probably yes (PY), probably no (PN), no (N) or no information (NI). All signalling questions are phrased so that “yes” indicates absence of bias. Any signalling question rated as “no” or “probably no” flags the potential for bias; you will need to use your judgement to determine whether the domain should be rated as “high”, “low” or “unclear” risk of bias. The guidance document contains further instructions and examples on rating signalling questions and risk of bias for each domain.

The first three domains are also rated for concerns regarding applicability (low/ high/ unclear) to your review question defined above.

Complete all domains separately for each evaluation of a distinct model. Shaded boxes indicate where signalling questions do not apply and should not be answered.

<table>
<tr><td colspan="4">DOMAIN 1: Participants</td></tr>
<tr><td colspan="4">A. Risk of Bias</td></tr>
<tr><td colspan="4">Describe the sources of data and criteria for participant selection:

“We retrospectively reviewed the records of 664 patients who underwent radical or partial nephrectomy for pathologically confirmed renal cancer at Emory University Hospital, Atlanta, Georgia, USA, from 2005 to 2012. Patients underwent either radical nephrectomy or partial nephrectomy for presumed renal cancer and for enhancing renal tumors identified on cross-sectional imaging. Patients undergoing cytoreductive nephrectomy were also included. Inclusion criteria included age >18 years, pathologically con- firmed renal cancer, having undergone radical or partial nephrectomy, and having preoperative and postoperative CRP levels available for review. Exclusion criteria included incomplete CRP data, benign final pathology or upper tract urothelial cell carcinoma. A total of 516 patients (77.7%) met the inclusion criteria and were considered in this analysis”</td></tr>
<tr><td colspan="2"></td><td>Dev</td><td>Val</td></tr>
<tr><td colspan="2">1.1 Were appropriate data sources used, e.g. cohort, RCT or nested case-control study data?</td><td>Y</td><td>N/A</td></tr>
<tr><td colspan="2">1.2 Were all inclusions and exclusions of participants appropriate?</td><td>Y</td><td>N/A</td></tr>
<tr><td>Risk of bias introduced by selection of participants</td><td>RISK:
(low/ high/ unclear)</td><td>Low</td><td>N/A</td></tr>
<tr><td colspan="4">Rationale of bias rating:
Cohort study with clear inclusion criterial</td></tr>
<tr><td colspan="4">B. Applicability</td></tr>
<tr><td colspan="4">Describe included participants, setting and dates:

“We retrospectively reviewed the records of 664 patients who underwent radical or partial nephrectomy for pathologically confirmed renal cancer at Emory University Hospital, Atlanta, Georgia, USA, from 2005 to 2012. Patients underwent either radical nephrectomy or partial nephrectomy for presumed renal cancer and for enhancing renal tumors identified on cross-sectional imaging. Patients undergoing cytoreductive nephrectomy were also included. Inclusion criteria included age >18 years, pathologically con- firmed renal cancer, having undergone radical or partial nephrectomy, and having preoperative and postoperative CRP levels available for review. Exclusion criteria included incomplete CRP data, benign final pathology or upper tract urothelial cell carcinoma. A total of 516 patients (77.7%) met the inclusion criteria and were considered in this analysis”</td></tr>
<tr><td>Concern that the included participants and setting do not match the review question</td><td>CONCERN:
(low/ high/ unclear)</td><td>Low</td><td>N/A</td></tr>
<tr><td colspan="4">Rationale of applicability rating:
Cohort study with clear inclusion criterial</td></tr>
</table>

**DOMAIN 2: Predictors**			
**A. Risk of Bias**			
List and describe predictors included in the final model, e.g. definition and timing of assessment:<br><br>The predictors included in the model were:<br>- In the preoperative model: M stage, preoperative CRP, age at surgery<br>- In the postoperative model: M stage, preoperative and postoperative CRP, age at surgery<br><br>All the predictors were measured at diagnosis in the preoperative model.<br>On the postoperative model the predictors were measured at diagnosis and after the treatment.			
		Dev	Val
2.1 Were predictors defined and assessed in a similar way for all participants?		PY	N/A
2.2 Were predictor assessments made without knowledge of outcome data?		PY	N/A
2.3 Are all predictors available at the time the model is intended to be used?		PY	N/A
**Risk of bias introduced by predictors or their assessment**	**RISK:**<br>*(low/ high/ unclear)*	Low	N/A
Rationale of bias rating:<br>*The blinding of measurement is unknown but all the predictors are objective.*			
**B. Applicability**			
Concern that the definition, assessment or timing of predictors in the model do not match the review question	**CONCERN:**<br>*(low/ high/ unclear)*	Low	N/A
Rationale of applicability rating:<br>*No major issues identified.*			

<table>
<tr><th colspan="4">DOMAIN 3: Outcome</th></tr>
<tr><th colspan="4">A. Risk of Bias</th></tr>
<tr><td colspan="4">Describe the outcome, how it was defined and determined, and the time interval between predictor assessment and outcome determination:
The outcome was cancer specific survival at 3 years</td></tr>
<tr><td colspan="2"></td><td>Dev</td><td>Val</td></tr>
<tr><td colspan="2">3.1 Was the outcome determined appropriately?</td><td>PY</td><td>N/A</td></tr>
<tr><td colspan="2">3.2 Was a pre-specified or standard outcome definition used?</td><td>Y</td><td>N/A</td></tr>
<tr><td colspan="2">3.3 Were predictors excluded from the outcome definition?</td><td>PY</td><td>N/A</td></tr>
<tr><td colspan="2">3.4 Was the outcome defined and determined in a similar way for all participants?</td><td>PY</td><td>N/A</td></tr>
<tr><td colspan="2">3.5 Was the outcome determined without knowledge of predictor information?</td><td>PY</td><td>N/A</td></tr>
<tr><td colspan="2">3.6 Was the time interval between predictor assessment and outcome determination appropriate?</td><td>Y</td><td>N/A</td></tr>
<tr><td>Risk of bias introduced by the outcome or its determination</td><td>RISK:
(low/ high/ unclear)</td><td>Low</td><td>N/A</td></tr>
<tr><td colspan="4">Rationale of bias rating:
No major issues identified</td></tr>
<tr><th colspan="4">B. Applicability</th></tr>
<tr><td colspan="4">At what time point was the outcome determined:
3 years

If a composite outcome was used, describe the relative frequency/distribution of each contributing outcome:
N/A</td></tr>
<tr><td>Concern that the outcome, its definition, timing or determination do not match the review question</td><td>CONCERN:
(low/ high/ unclear)</td><td>Low</td><td>N/A</td></tr>
<tr><td colspan="4">Rationale of applicability rating:
The outcome of the primary study matches the outcome of interest of the review</td></tr>
</table>

**DOMAIN 4: Analysis**		
**Risk of Bias**		
Describe numbers of participants, number of candidate predictors, outcome events and events per candidate predictor:<br>*"A total of 516 patients (77.7%) met the inclusion criteria and were considered in this analysis"*<br>*The total number of predictors is 21 (Table 3)*<br>*The number of events were 53*<br><br>*We can´t calculate EPV because we don´t known how many splines functions they used to each continuous candidate predictors. Despite of that probably the EPV is not over 20 due the number of events.*		
Describe how the model was developed (for example in regards to modelling technique (e.g. survival or logistic modelling), predictor selection, and risk group definition):<br><br>*"First, univariable Cox proportional hazards regression and competing risks regression were carried out for each predictor to examine the ability of predicting OM and RCC-SM, respectively. After that, all statistically significant predictors (P<0.05) in the aforementioned univariable analysis were analyzed in multivariable Cox proportional hazards regression for OM or competing risks regression for RCC-SM. For each outcome, OM or RCC-SM, the corresponding multivariable regression analysis was carried out separately preoperatively and postoperatively. To reduce the complexity of the final models, we used the stepdown method to eliminate redundant predictors, and created more parsimonious models with fewer predictors, but preserved predictive accuracy, on which the final nomograms were built"*		
Describe whether and how the model was validated, either internally (e.g. bootstrapping, cross validation, random split sample) or externally (e.g. temporal validation, geographical validation, different setting, different type of participants):<br><br>*"Nomograms were internally validated using bootstrapping with 1000 resamples for OM and 10-fold cross-validation for RCC-SM, respectively, to estimate the predictive performance when the models are applied to new patients"*		
Describe the performance measures of the model, e.g. (re)calibration, discrimination, (re)classification, net benefit, and whether they were adjusted for optimism:<br><br>*"In the first step of the validation, the concordance index was calculated to quan- tify the ability of the nomograms to discriminate patients with versus without outcomes"*<br><br>*"Additionally, calibration was visually checked by plotting the nomogram-predicted probabilities against the observed proportions"*		
Describe any participants who were excluded from the analysis:<br>*"Exclusion criteria included incomplete CRP data, benign final pathology or upper tract urothelial cell carcinoma"*<br>*They excluded 148 patients*		
Describe missing data on predictors and outcomes as well as methods used for missing data:<br>"Missing values in the predictors were multiply imputed"		
	Dev	Val
4.1 Were there a reasonable number of participants with the outcome?	N	N/A

4.2 Were continuous and categorical predictors handled appropriately?		Y	N/A
4.3 Were all enrolled participants included in the analysis?		N	N/A
4.4 Were participants with missing data handled appropriately?		Y	N/A
4.5 Was selection of predictors based on univariable analysis avoided?		N	
4.6 Were complexities in the data (e.g. censoring, competing risks, sampling of controls) accounted for appropriately?		Y	N/A
4.7 Were relevant model performance measures evaluated appropriately?		Y	N/A
4.8 Were model overfitting and optimism in model performance accounted for?		Y	
4.9 Do predictors and their assigned weights in the final model correspond to the results from multivariable analysis?		PY	
**Risk of bias introduced by the analysis**	**RISK:** *(low/ high/ unclear)*	High	N/A
Rationale of bias rating: They hadn´t enough patients with the outcome. They selected the predictors based on univariable.			

Step 4: Overall assessment

Use the following tables to reach overall judgements about risk of bias and concerns regarding applicability of the prediction model evaluation (development and/or validation) across all assessed domains.
Complete for each evaluation of a distinct model.

**Reaching an overall judgement about risk of bias of the prediction model evaluation**	
**Low risk of bias**	If all domains were rated low risk of bias.<br>If a prediction model was developed without any external validation, and it was rated as low risk of bias for all domains, consider downgrading to **high risk of bias**. Such a model can only be considered as low risk of bias, if the development was based on a very large data set and included some form of internal validation.
**High risk of bias**	If at least one domain is judged to be at **high risk of bias**.
**Unclear risk of bias**	If an unclear risk of bias was noted in at least one domain and it was low risk for all other domains.

**Reaching an overall judgement about applicability of the prediction model evaluation**	
**Low concerns regarding applicability**	If low concerns regarding applicability for all domains, the prediction model evaluation is judged to have **low concerns regarding applicability**.
**High concerns regarding applicability**	If high concerns regarding applicability for at least one domain, the prediction model evaluation is judged to have **high concerns regarding applicability**.
**Unclear concerns regarding applicability**	If unclear concerns (but no "high concern") regarding applicability for at least one domain, the prediction model evaluation is judged to have **unclear concerns regarding applicability** overall.

**Overall judgement about risk of bias and applicability of the prediction model evaluation**		
**Overall judgement of risk of bias**	**RISK:**<br>*(low/ high/ unclear)*	High
Summary of sources of potential bias:<br>Analysis domain shows several major issues		
**Overall judgement of applicability**	**CONCERN:**<br>*(low/ high/ unclear)*	Low
Summary of applicability concerns:<br>No several major issues		

PROBAST

(Prediction model study Risk Of Bias Assessment Tool)

Published in Annals of Internal Medicine (freely available):

1. PROBAST: A Tool to Assess the Risk of Bias and Applicability of Prediction Model Studies
2. PROBAST: A Tool to Assess Risk of Bias and Applicability of Prediction Model Studies: Explanation and Elaboration

What does PROBAST assess?

PROBAST assesses both the *risk of bias* and *concerns regarding applicability* of a study that evaluates (develops, validates or updates) a multivariable diagnostic or prognostic prediction model. It is designed to assess primary studies included in a systematic review.

Bias occurs if systematic flaws or limitations in the design, conduct or analysis of a primary study distort the results. For the purpose of prediction modelling studies, we have defined *risk of bias* to occur when shortcomings in the study design, conduct or analysis lead to systematically distorted estimates of a model's predictive performance or to an inadequate model to address the research question. Model predictive performance is typically evaluated using calibration, discrimination and sometimes classification measures, and these are likely inaccurately estimated in studies with high risk of bias. *Applicability* refers to the extent to which the prediction model from the primary study matches your systematic review question, for example in terms of the participants, predictors or outcome of interest.

A primary study may include the development and/or validation or update of more than one prediction model. A PROBAST assessment should be completed for each distinct model that is developed, validated or updated (extended) for making individualised predictions. Where a publication assesses multiple prediction models, only complete a PROBAST assessment for those models that meet the inclusion criteria for your systematic review. Please note that subsequent use of the term "model" includes derivatives of models, such as simplified risk scores, nomograms, or recalibrations of models.

PROBAST is not designed for all multivariable diagnostic or prognostic studies. For example, studies using multivariable models to identify predictors associated with an outcome but not attempting to develop a model for making individualised predictions are not covered by PROBAST.

PROBAST includes four steps.

**Step**	**Task**	**When to complete**
**1**	Specify your systematic review question(s)	Once per systematic review
**2**	Classify the type of prediction model evaluation	Once for each model of interest in each publication being assessed, for each relevant outcome
**3**	Assess risk of bias and applicability	Once for each development and validation of each distinct prediction model in a publication
**4**	Overall judgment	Once for each development and validation of each distinct prediction model in a publication

If this is your first time using PROBAST, we strongly recommend reading the detailed explanation and elaboration (E&E, see link above) paper and to check the examples on www.probast.org

Step 1: Specify your systematic review question

State your systematic review question to facilitate the assessment of the applicability of the evaluated models to your question. *The following table should be completed once per systematic review.*

Criteria	Specify your systematic review question
Intended use of model:	*To predict cancer specific mortality in patients with renal cancer treated with partial or total nephrectomy regardless of TNM stage*
***Participants** including selection criteria and setting:*	*Patients with renal cancer treated with partial or total nephrectomy regardless of TNM stage*
***Predictors** (used in prediction modelling), including types of predictors (e.g. history, clinical examination, biochemical markers, imaging tests), time of measurement, specific measurement issues (e.g., any requirements/ prohibitions for specialized equipment):*	*Predictors used in clinical practice measured when a nephrectomy for renal cancer is indicated*
Outcome to be predicted:	*Cancer specific mortality*

Step 2: Classify the type of prediction model evaluation

Use the following table to classify the evaluation as model development, model validation or model update, or combination. Different signalling questions apply for different types of prediction model evaluation. If the evaluation does not fit one of these classifications then PROBAST should not be used.

**Classify the evaluation based on its aim**			
**Type of prediction study**	**PROBAST boxes to complete**	**Tick as appropriate**	**Definition for type of prediction model study**
Development only	Development	✓	Prediction model development without external validation. These studies may include internal validation methods, such as bootstrapping and cross-validation techniques.
Development and validation	Development and validation	✗	Prediction model development combined with external validation in other participants in the same article.
Validation only	Validation	✗	External validation of existing (previously developed) model in other participants.

This table should be completed once for each publication being assessed and for each relevant outcome in your review.

**Publication reference**	Cho KG, Choi YD, Kim SJ, Kim CI, Chung BH, Seong DH, Lee DH, Cho JS, Cho IR, Hong SJ. A Comprehensive Prognostic Stratification for Patients with Metastatic Renal Clear Cell Carcinom. Yonsei Med J. 2008. 30; 49(3): 451–458
**Models of interest**	Risk score
**Outcome of interest**	*Cancer specific mortality*

Step 3: Assess risk of bias and applicability

PROBAST is structured as four key domains. Each domain is judged for risk of bias (low, high or unclear) and includes signalling questions to help make judgements. Signalling questions are rated as yes (Y), probably yes (PY), probably no (PN), no (N) or no information (NI). All signalling questions are phrased so that "yes" indicates absence of bias. Any signalling question rated as "no" or "probably no" flags the potential for bias; you will need to use your judgement to determine whether the domain should be rated as "high", "low" or "unclear" risk of bias. The guidance document contains further instructions and examples on rating signalling questions and risk of bias for each domain.

The first three domains are also rated for concerns regarding applicability (low/ high/ unclear) to your review question defined above.

Complete all domains separately for each evaluation of a distinct model. Shaded boxes indicate where signalling questions do not apply and should not be answered.

DOMAIN 1: Participants			
**A. Risk of Bias**			

Describe the sources of data and criteria for participant selection:

"The medical records of 368 patients with histologically proven metastatic RCC from 8 university hospitals were retrospectively reviewed. The cohort was limited to patients who underwent radical nephrectomy and treatment between 1995 and 2004 with at least 1 cycle of immunotherapy [interferon-α, interleukin-2 (IL-2), or a combination thereof with or without 5-flourouracil]. Patients who received other biologic response modifiers and chemotherapeutic regimens were excluded. Exclusion criteria also included non-clear cell histology, von Hippel-Lindau disease, other malignant disease, and follow-up duration of less than 3 months. A total of 197 patients were eligible for this study"

		Dev	Val
1.1 Were appropriate data sources used, e.g. cohort, RCT or nested case-control study data?		Y	N/A
1.2 Were all inclusions and exclusions of participants appropriate?		N	N/A
**Risk of bias introduced by selection of participants**	**RISK:** *(low/ high/ unclear)*	High	N/A

Rationale of bias rating:

They excluded patients who received other biologic response modifiers and chemotherapeutic regimens, non-clear cell histology, von Hippel-Lindau disease, other malignant disease, and follow-up duration of less than 3 months

B. Applicability

Describe included participants, setting and dates:

"The medical records of 368 patients with histologically proven metastatic RCC from 8 university hospitals were retrospectively reviewed. The cohort was limited to patients who underwent radical nephrectomy and treatment between 1995 and 2004 with at least 1 cycle of immunotherapy [interferon-α, interleukin-2 (IL-2), or a combination thereof with or without 5-flourouracil]. Patients who received other biologic response modifiers and chemotherapeutic regimens were excluded. Exclusion criteria also included non-clear cell histology, von Hippel-Lindau disease, other malignant disease, and follow-up duration of less than 3 months. A total of 197 patients were eligible for this study"

**Concern that the included participants and setting do not match the review question**	**CONCERN:** *(low/ high/ unclear)*	High	N/A

Rationale of applicability rating:

They excluded patients who received other biologic response modifiers and chemotherapeutic regimens, non-clear cell histology, von Hippel-Lindau disease, other malignant disease, and follow-up duration of less than 3 months

DOMAIN 2: Predictors			
**A. Risk of Bias**			
List and describe predictors included in the final model, e.g. definition and timing of assessment:<br><br>The predictors included in the model were: sarcomatoid differentiation, liver metastasis, ECOG, N-stage and number of metastatic sites<br><br>All the predictors were measured at diagnosis or after the treatment			
		Dev	Val
2.1 Were predictors defined and assessed in a similar way for all participants?		PY	N/A
2.2 Were predictor assessments made without knowledge of outcome data?		PY	N/A
2.3 Are all predictors available at the time the model is intended to be used?		PY	N/A
**Risk of bias introduced by predictors or their assessment**	**RISK:**<br>*(low/ high/ unclear)*	Low	N/A
Rationale of bias rating:<br>*The blinding of measurement is unknown but all the predictors are objective.*			
**B. Applicability**			
Concern that the definition, assessment or timing of predictors in the model do not match the review question	**CONCERN:**<br>*(low/ high/ unclear)*	Low	N/A
Rationale of applicability rating:<br>*No major issues identified.*			

DOMAIN 3: Outcome			
**A. Risk of Bias**			
Describe the outcome, how it was defined and determined, and the time interval between predictor assessment and outcome determination:<br>The outcome was cancer specific survival at 1,3 and 5 years			
		Dev	Val
3.1 Was the outcome determined appropriately?		PY	N/A
3.2 Was a pre-specified or standard outcome definition used?		Y	N/A
3.3 Were predictors excluded from the outcome definition?		PY	N/A
3.4 Was the outcome defined and determined in a similar way for all participants?		PY	N/A
3.5 Was the outcome determined without knowledge of predictor information?		PY	N/A
3.6 Was the time interval between predictor assessment and outcome determination appropriate?		Y	N/A
**Risk of bias introduced by the outcome or its determination**	**RISK:**<br>*(low/ high/ unclear)*	Low	N/A
Rationale of bias rating:<br>*No major issues identified*			
**B. Applicability**			
At what time point was the outcome determined:<br>1,3 and 5 years<br><br>*If a composite outcome was used, describe the relative frequency/distribution of each contributing outcome:*<br>*N/A*			
**Concern that the outcome, its definition, timing or determination do not match the review question**	**CONCERN:**<br>*(low/ high/ unclear)*	Low	N/A
Rationale of applicability rating:<br>*The outcome of the primary study matches the outcome of interest of the review*			

**DOMAIN 4: Analysis**		
**Risk of Bias**		
Describe numbers of participants, number of candidate predictors, outcome events and events per candidate predictor:<br>"A total of 197 patients were eligible for this study"<br><br>The total number of predictors is 24 (table 2)<br>The number of events is 127<br><br>EPV= 127/24=5.29		
Describe how the model was developed (for example in regards to modelling technique (e.g. survival or logistic modelling), predictor selection, and risk group definition):<br><br>"Kaplan-Meier curves were generated and compared by using log-rank test for univariate survival analyses. To assess the independent impact of clinicopathological factors on disease- specific survival, Cox proportional hazards regression was used for multivariate survival analyses. Based on rounded regression coefficients [log hazard ratios (HR) in the final Cox model] of variables, the weights of prognostic features were determined. A prognostic score was defined as the sum of the weights of the independent prognostic factors"		
Describe whether and how the model was validated, either internally (e.g. bootstrapping, cross validation, random split sample) or externally (e.g. temporal validation, geographical validation, different setting, different type of participants):<br><br>Not indicated		
Describe the performance measures of the model, e.g. (re)calibration, discrimination, (re)classification, net benefit, and whether they were adjusted for optimism:<br><br>Not indicated		
Describe any participants who were excluded from the analysis:<br>*"Patients who received other biologic response modifiers and chemotherapeutic regimens were excluded. Exclusion criteria also included non-clear cell histology, von Hippel-Lindau disease, other malignant disease, and follow-up duration of less than 3 mos"*		
Describe missing data on predictors and outcomes as well as methods used for missing data:<br>Not indicated		
	Dev	Val
4.1 Were there a reasonable number of participants with the outcome?	N	N/A
4.2 Were continuous and categorical predictors handled appropriately?	N	N/A
4.3 Were all enrolled participants included in the analysis?	Y	N/A
4.4 Were participants with missing data handled appropriately?	NI	N/A
4.5 Was selection of predictors based on univariable analysis avoided?	N	
4.6 Were complexities in the data (e.g. censoring, competing risks, sampling of controls) accounted for appropriately?	N	N/A
4.7 Were relevant model performance measures evaluated appropriately?	N	N/A

4.8 Were model overfitting and optimism in model performance accounted for?		N	
4.9 Do predictors and their assigned weights in the final model correspond to the results from multivariable analysis?		PY	
**Risk of bias introduced by the analysis**	**RISK:** *(low/ high/ unclear)*	High	N/A
Rationale of bias rating: *They didn´t have enough patients with the outcome. They did categorizations. They didn´t say anything about missing data. They selected the predictors based on univariable. They didn´t do validation, calibration or discrimination. They didn't use competing risks.*			

Step 4: Overall assessment

Use the following tables to reach overall judgements about risk of bias and concerns regarding applicability of the prediction model evaluation (development and/or validation) across all assessed domains.
Complete for each evaluation of a distinct model.

**Reaching an overall judgement about risk of bias of the prediction model evaluation**	
**Low risk of bias**	If all domains were rated low risk of bias.<br>If a prediction model was developed without any external validation, and it was rated as low risk of bias for all domains, consider downgrading to **high risk of bias**. Such a model can only be considered as low risk of bias, if the development was based on a very large data set and included some form of internal validation.
**High risk of bias**	If at least one domain is judged to be at **high risk of bias**.
**Unclear risk of bias**	If an unclear risk of bias was noted in at least one domain and it was low risk for all other domains.

**Reaching an overall judgement about applicability of the prediction model evaluation**	
**Low concerns regarding applicability**	If low concerns regarding applicability for all domains, the prediction model evaluation is judged to have **low concerns regarding applicability**.
**High concerns regarding applicability**	If high concerns regarding applicability for at least one domain, the prediction model evaluation is judged to have **high concerns regarding applicability**.
**Unclear concerns regarding applicability**	If unclear concerns (but no "high concern") regarding applicability for at least one domain, the prediction model evaluation is judged to have **unclear concerns regarding applicability** overall.

**Overall judgement about risk of bias and applicability of the prediction model evaluation**		
**Overall judgement of risk of bias**	**RISK:** *(low/ high/ unclear)*	High
Summary of sources of potential bias:<br>Analysis and participants domains show several major issues		
**Overall judgement of applicability**	**CONCERN:** *(low/ high/ unclear)*	High
Summary of applicability concerns:<br><br>Participants domain show several major issues		

PROBAST

(Prediction model study Risk Of Bias Assessment Tool)

Published in Annals of Internal Medicine (freely available):

1. PROBAST: A Tool to Assess the Risk of Bias and Applicability of Prediction Model Studies
2. PROBAST: A Tool to Assess Risk of Bias and Applicability of Prediction Model Studies: Explanation and Elaboration

What does PROBAST assess?

PROBAST assesses both the *risk of bias* and *concerns regarding applicability* of a study that evaluates (develops, validates or updates) a multivariable diagnostic or prognostic prediction model. It is designed to assess primary studies included in a systematic review.

Bias occurs if systematic flaws or limitations in the design, conduct or analysis of a primary study distort the results. For the purpose of prediction modelling studies, we have defined *risk of bias* to occur when shortcomings in the study design, conduct or analysis lead to systematically distorted estimates of a model's predictive performance or to an inadequate model to address the research question. Model predictive performance is typically evaluated using calibration, discrimination and sometimes classification measures, and these are likely inaccurately estimated in studies with high risk of bias. *Applicability* refers to the extent to which the prediction model from the primary study matches your systematic review question, for example in terms of the participants, predictors or outcome of interest.

A primary study may include the development and/or validation or update of more than one prediction model. A PROBAST assessment should be completed for each distinct model that is developed, validated or updated (extended) for making individualised predictions. Where a publication assesses multiple prediction models, only complete a PROBAST assessment for those models that meet the inclusion criteria for your systematic review. Please note that subsequent use of the term "model" includes derivatives of models, such as simplified risk scores, nomograms, or recalibrations of models.

PROBAST is not designed for all multivariable diagnostic or prognostic studies. For example, studies using multivariable models to identify predictors associated with an outcome but not attempting to develop a model for making individualised predictions are not covered by PROBAST.

PROBAST includes four steps.

Ste p	Task	When to complete
**1**	Specify your systematic review question(s)	Once per systematic review
**2**	Classify the type of prediction model evaluation	Once for each model of interest in each publication being assessed, for each relevant outcome
**3**	Assess risk of bias and applicability	Once for each development and validation of each distinct prediction model in a publication
**4**	Overall judgment	Once for each development and validation of each distinct prediction model in a publication

If this is your first time using PROBAST, we strongly recommend reading the detailed explanation and elaboration (E&E, see link above) paper and to check the examples on www.probast.org

Step 1: Specify your systematic review question

State your systematic review question to facilitate the assessment of the applicability of the evaluated models to your question. *The following table should be completed once per systematic review.*

Criteria	Specify your systematic review question
Intended use of model:	*To predict cancer specific mortality in patients with renal cancer treated with partial or total nephrectomy regardless of TNM stage*
***Participants** including selection criteria and setting:*	*Patients with renal cancer treated with partial or total nephrectomy regardless of TNM stage*
***Predictors** (used in prediction modelling), including types of predictors (e.g. history, clinical examination, biochemical markers, imaging tests), time of measurement, specific measurement issues (e.g., any requirements/ prohibitions for specialized equipment):*	*Predictors used in clinical practice measured when a nephrectomy for renal cancer is indicated*
Outcome to be predicted:	*Cancer specific mortality*

Step 2: Classify the type of prediction model evaluation

Use the following table to classify the evaluation as model development, model validation or model update, or combination. Different signalling questions apply for different types of prediction model evaluation. If the evaluation does not fit one of these classifications then PROBAST should not be used.

**Classify the evaluation based on its aim**			
**Type of prediction study**	**PROBAST boxes to complete**	**Tick as appropriate**	**Definition for type of prediction model study**
Development only	Development	✓	Prediction model development without external validation. These studies may include internal validation methods, such as bootstrapping and cross-validation techniques.
Development and validation	Development and validation	✗	Prediction model development combined with external validation in other participants in the same article.
Validation only	Validation	✗	External validation of existing (previously developed) model in other participants.

This table should be completed once for each publication being assessed and for each relevant outcome in your review.

**Publication reference**	Su X, Hou NN, Yang LJ, Li PX, Yang XJ, Hou GD, Gao XL, Ma SJ, Guo F, Zhang R, Zhang WH, Qin WJ, Wang FL. The first competing risk survival nomogram in patients with papillary renal cell carcinoma. Sci rep. 2021 4;11(1):11835
**Models of interest**	Nomogram
**Outcome of interest**	*Cancer specific mortality*

Step 3: Assess risk of bias and applicability

PROBAST is structured as four key domains. Each domain is judged for risk of bias (low, high or unclear) and includes signalling questions to help make judgements. Signalling questions are rated as yes (Y), probably yes (PY), probably no (PN), no (N) or no information (NI). All signalling questions are phrased so that "yes" indicates absence of bias. Any signalling question rated as "no" or "probably no" flags the potential for bias; you will need to use your judgement to determine whether the domain should be rated as "high", "low" or "unclear" risk of bias. The guidance document contains further instructions and examples on rating signalling questions and risk of bias for each domain.

The first three domains are also rated for concerns regarding applicability (low/ high/ unclear) to your review question defined above.

Complete all domains separately for each evaluation of a distinct model. Shaded boxes indicate where signalling questions do not apply and should not be answered.

<table>
<tr><th colspan="4">DOMAIN 1: Participants</th></tr>
<tr><th colspan="4">A. Risk of Bias</th></tr>
<tr><td colspan="4">Describe the sources of data and criteria for participant selection:

“Data of patients with pRCC (ICD-O-3 site code C64.9 and histology code 8260/3) between 2010 and 2016 were extracted from the SEER database (2004–2016 dataset), which contains the population-based cancer incidence information from 18 registries and covers nearly 28% of the United States population22. To ensure at least one year of follow-up, we excluded patients who were diagnosed after December 31, 2015. A total of 9690 patients were identified through the SEER*Stat software (username: 10646-Nov 2018).
The inclusion criteria of eligible patients were as follows: (1) primary pRCC; (2) diagnosis with positive histology; (3) underwent partial or radical nephrectomy. (4) Unilateral tumor. (5) Aged over 18 years. The exclusion criteria were patients who received chemotherapy, and with unknown or missing information on surgery records, tumor side, TNM stages, cause of death, race and survival time. In addition, we also excluded patients with brain metastases (N = 7) and multiple metastases (N = 7) as the cases were very few. Eventually, a total of 5993 patients were eligible in the present study”</td></tr>
<tr><td colspan="2"></td><td>Dev</td><td>Val</td></tr>
<tr><td colspan="2">1.1 Were appropriate data sources used, e.g. cohort, RCT or nested case-control study data?</td><td>Y</td><td>N/A</td></tr>
<tr><td colspan="2">1.2 Were all inclusions and exclusions of participants appropriate?</td><td>N</td><td>N/A</td></tr>
<tr><td>Risk of bias introduced by selection of participants</td><td>RISK:
(low/ high/ unclear)</td><td>High</td><td>N/A</td></tr>
<tr><td colspan="4">Rationale of bias rating:
Cohort study only include patients with papillary renal tumor</td></tr>
<tr><th colspan="4">B. Applicability</th></tr>
<tr><td colspan="4">Describe included participants, setting and dates:
“Data of patients with pRCC (ICD-O-3 site code C64.9 and histology code 8260/3) between 2010 and 2016 were extracted from the SEER database (2004–2016 dataset), which contains the population-based cancer incidence information from 18 registries and covers nearly 28% of the United States population22. To ensure at least one year of follow-up, we excluded patients who were diagnosed after December 31, 2015. A total of 9690 patients were identified through the SEER*Stat software (username: 10646-Nov 2018).
The inclusion criteria of eligible patients were as follows: (1) primary pRCC; (2) diagnosis with positive histology; (3) underwent partial or radical nephrectomy. (4) Unilateral tumor. (5) Aged over 18 years. The exclusion criteria were patients who received chemotherapy, and with unknown or missing information on surgery records, tumor side, TNM stages, cause of death, race and survival time. In addition, we also excluded patients with brain metastases (N = 7) and multiple metastases (N = 7) as the cases were very few. Eventually, a total of 5993 patients were eligible in the present study”</td></tr>
<tr><td>Concern that the included participants and setting do not match the review question</td><td>CONCERN:
(low/ high/ unclear)</td><td>High</td><td>N/A</td></tr>
<tr><td colspan="4">Rationale of applicability rating:
Cohort study only include patients with papillary renal tumor</td></tr>
</table>

DOMAIN 2: Predictors			
**A. Risk of Bias**			
List and describe predictors included in the final model, e.g. definition and timing of assessment:<br><br>The predictors included in the model were: age, grade, T and N stage and bone, liver and lung metastases<br><br>All the predictors were measured at diagnosis or after the treatment			
		Dev	Val
2.1 Were predictors defined and assessed in a similar way for all participants?		PY	N/A
2.2 Were predictor assessments made without knowledge of outcome data?		PY	N/A
2.3 Are all predictors available at the time the model is intended to be used?		PY	N/A
**Risk of bias introduced by predictors or their assessment**	**RISK:**<br>*(low/ high/ unclear)*	Low	N/A
Rationale of bias rating:<br>*The blinding of measurement is unknown but all the predictors are objective.*			
**B. Applicability**			
Concern that the definition, assessment or timing of predictors in the model do not match the review question	**CONCERN:**<br>*(low/ high/ unclear)*	Low	N/A
Rationale of applicability rating:<br>*No major issues identified.*			

DOMAIN 3: Outcome			
**A. Risk of Bias**			
Describe the outcome, how it was defined and determined, and the time interval between predictor assessment and outcome determination:<br>The outcome was cancer specific survival at 2, 3 and 5 years			
		Dev	Val
3.1 Was the outcome determined appropriately?		PY	N/A
3.2 Was a pre-specified or standard outcome definition used?		Y	N/A
3.3 Were predictors excluded from the outcome definition?		PY	N/A
3.4 Was the outcome defined and determined in a similar way for all participants?		PY	N/A
3.5 Was the outcome determined without knowledge of predictor information?		PY	N/A
3.6 Was the time interval between predictor assessment and outcome determination appropriate?		Y	N/A
**Risk of bias introduced by the outcome or its determination**	**RISK:**<br>*(low/ high/ unclear)*	Low	N/A
Rationale of bias rating:<br>*No major issues identified*			
**B. Applicability**			
At what time point was the outcome determined:<br>2,3 and 5 years<br><br>*If a composite outcome was used, describe the relative frequency/distribution of each contributing outcome:*<br>*N/A*			
**Concern that the outcome, its definition, timing or determination do not match the review question**	**CONCERN:**<br>*(low/ high/ unclear)*	Low	N/A
Rationale of applicability rating:<br>*The outcome of the primary study matches the outcome of interest of the review*			

DOMAIN 4: Analysis
**Risk of Bias**
Describe numbers of participants, number of candidate predictors, outcome events and events per candidate predictor:<br>"Data of patients with pRCC (ICD-O-3 site code C64.9 and histology code 8260/3) between 2010 and 2016 were extracted from the SEER database (2004–2016 dataset), which contains the population-based cancer incidence information from 18 registries and covers nearly 28% of the United States population22. To ensure at least one year of follow-up, we excluded patients who were diagnosed after December 31, 2015. A total of 9690 patients were identified through the SEER*Stat software (username: 10646-Nov 2018).<br>The inclusion criteria of eligible patients were as follows: (1) primary pRCC; (2) diagnosis with positive histology; (3) underwent partial or radical nephrectomy. (4) Unilateral tumor. (5) Aged over 18 years. The exclusion criteria were patients who received chemotherapy, and with unknown or missing information on surgery records, tumor side, TNM stages, cause of death, race and survival time. In addition, we also excluded patients with brain metastases (N = 7) and multiple metastases (N = 7) as the cases were very few. Eventually, a total of 5993 patients were eligible in the present study"<br><br>The total number of predictors is 17 (table 2)<br>The number of events is 298<br>EPV= 298/17=17.53
Describe how the model was developed (for example in regards to modelling technique (e.g. survival or logistic modelling), predictor selection, and risk group definition):<br><br>"At first, we performed the cumulative incidence function (CIF) to describe the probability of each event among the categorical variables over time and plotted the corresponding CIF curves at the same time. The differences within the subgroups were assessed by Gray's test. Second, significant variables in univariable analysis (*P* < 0.05) were selected to fit the optimal proportional subdistribution hazard model using a backward elimination method and the 2-, 3-, and 5-year prognostic nomogram for CSM was further generated based on the significant model coefficients"
Describe whether and how the model was validated, either internally (e.g. bootstrapping, cross validation, random split sample) or externally (e.g. temporal validation, geographical validation, different setting, different type of participants):<br><br>"Finally, the predictive per- formance of our nomogram was internally validated via bootstrapping with 1000 resamples"
Describe the performance measures of the model, e.g. (re)calibration, discrimination, (re)classification, net benefit, and whether they were adjusted for optimism:<br><br>"The discrimination was measured by the time-dependent area under the receiver operating characteristic (ROC) curve (AUCt)26. Subsequently, the 2-, 3-, and 5-year calibration curves were plotted to visually compare the nomogram-predicted probabilities with the observed CSM rates"
Describe any participants who were excluded from the analysis:<br>"To ensure at least one year of follow-up, we excluded patients who were diagnosed after December 31, 2015"<br>"The exclusion criteria were patients who received chemotherapy, and with unknown or missing information on surgery records, tumor side, TNM stages, cause of death, race and survival time. In addition, we also excluded patients with brain metastases (N = 7) and multiple metastases (N = 7) as the

cases were very few”			
Describe missing data on predictors and outcomes as well as methods used for missing data:<br>“The exclusion criteria were patients who received chemotherapy, and with unknown or missing information on surgery records, tumor side, TNM stages, cause of death, race and survival time”			
		Dev	Val
4.1 Were there a reasonable number of participants with the outcome?		N	N/A
4.2 Were continuous and categorical predictors handled appropriately?		N	N/A
4.3 Were all enrolled participants included in the analysis?		N	N/A
4.4 Were participants with missing data handled appropriately?		N	N/A
4.5 Was selection of predictors based on univariable analysis avoided?		N	
4.6 Were complexities in the data (e.g. censoring, competing risks, sampling of controls) accounted for appropriately?		N	N/A
4.7 Were relevant model performance measures evaluated appropriately?		Y	N/A
4.8 Were model overfitting and optimism in model performance accounted for?		Y	
4.9 Do predictors and their assigned weights in the final model correspond to the results from multivariable analysis?		PY	
**Risk of bias introduced by the analysis**	**RISK:**<br>*(low/ high/ unclear)*	High	N/A
Rationale of bias rating:<br>*They didn´t have enough patients with the outcome. They did categorizations. They excluded patients with missing data. They selected the predictors based on univariable. They didn’t use competing risks.*			

Step 4: Overall assessment

Use the following tables to reach overall judgements about risk of bias and concerns regarding applicability of the prediction model evaluation (development and/or validation) across all assessed domains.
Complete for each evaluation of a distinct model.

**Reaching an overall judgement about risk of bias of the prediction model evaluation**	
**Low risk of bias**	If all domains were rated low risk of bias.<br>If a prediction model was developed without any external validation, and it was rated as low risk of bias for all domains, consider downgrading to **high risk of bias**. Such a model can only be considered as low risk of bias, if the development was based on a very large data set and included some form of internal validation.
**High risk of bias**	If at least one domain is judged to be at **high risk of bias**.
**Unclear risk of bias**	If an unclear risk of bias was noted in at least one domain and it was low risk for all other domains.

**Reaching an overall judgement about applicability of the prediction model evaluation**	
**Low concerns regarding applicability**	If low concerns regarding applicability for all domains, the prediction model evaluation is judged to have **low concerns regarding applicability**.
**High concerns regarding applicability**	If high concerns regarding applicability for at least one domain, the prediction model evaluation is judged to have **high concerns regarding applicability**.
**Unclear concerns regarding applicability**	If unclear concerns (but no "high concern") regarding applicability for at least one domain, the prediction model evaluation is judged to have **unclear concerns regarding applicability** overall.

**Overall judgement about risk of bias and applicability of the prediction model evaluation**		
**Overall judgement of risk of bias**	**RISK:**<br>*(low/ high/ unclear)*	High
Summary of sources of potential bias:<br>Analysis and participants domains show several major issues		
**Overall judgement of applicability**	**CONCERN:**<br>*(low/ high/ unclear)*	High
Summary of applicability concerns:<br><br>Participant domain shows several major issues		

PROBAST

(Prediction model study Risk Of Bias Assessment Tool)

Published in Annals of Internal Medicine (freely available):

1. PROBAST: A Tool to Assess the Risk of Bias and Applicability of Prediction Model Studies
2. PROBAST: A Tool to Assess Risk of Bias and Applicability of Prediction Model Studies: Explanation and Elaboration

What does PROBAST assess?

PROBAST assesses both the *risk of bias* and *concerns regarding applicability* of a study that evaluates (develops, validates or updates) a multivariable diagnostic or prognostic prediction model. It is designed to assess primary studies included in a systematic review.

Bias occurs if systematic flaws or limitations in the design, conduct or analysis of a primary study distort the results. For the purpose of prediction modelling studies, we have defined *risk of bias* to occur when shortcomings in the study design, conduct or analysis lead to systematically distorted estimates of a model's predictive performance or to an inadequate model to address the research question. Model predictive performance is typically evaluated using calibration, discrimination and sometimes classification measures, and these are likely inaccurately estimated in studies with high risk of bias. *Applicability* refers to the extent to which the prediction model from the primary study matches your systematic review question, for example in terms of the participants, predictors or outcome of interest.

A primary study may include the development and/or validation or update of more than one prediction model. A PROBAST assessment should be completed for each distinct model that is developed, validated or updated (extended) for making individualised predictions. Where a publication assesses multiple prediction models, only complete a PROBAST assessment for those models that meet the inclusion criteria for your systematic review. Please note that subsequent use of the term "model" includes derivatives of models, such as simplified risk scores, nomograms, or recalibrations of models.

PROBAST is not designed for all multivariable diagnostic or prognostic studies. For example, studies using multivariable models to identify predictors associated with an outcome but not attempting to develop a model for making individualised predictions are not covered by PROBAST.

PROBAST includes four steps.

Step	Task	When to complete
**1**	Specify your systematic review question(s)	Once per systematic review
**2**	Classify the type of prediction model evaluation	Once for each model of interest in each publication being assessed, for each relevant outcome
**3**	Assess risk of bias and applicability	Once for each development and validation of each distinct prediction model in a publication
**4**	Overall judgment	Once for each development and validation of each distinct prediction model in a publication

If this is your first time using PROBAST, we strongly recommend reading the detailed explanation and elaboration (E&E, see link above) paper and to check the examples on www.probast.org

Step 1: Specify your systematic review question

State your systematic review question to facilitate the assessment of the applicability of the evaluated models to your question. *The following table should be completed once per systematic review.*

Criteria	Specify your systematic review question
Intended use of model:	*To predict cancer specific mortality in patients with renal cancer treated with partial or total nephrectomy regardless of TNM stage*
***Participants** including selection criteria and setting:*	*Patients with renal cancer treated with partial or total nephrectomy regardless of TNM stage*
***Predictors** (used in prediction modelling), including types of predictors (e.g. history, clinical examination, biochemical markers, imaging tests), time of measurement, specific measurement issues (e.g., any requirements/ prohibitions for specialized equipment):*	*Predictors used in clinical practice measured when a nephrectomy for renal cancer is indicated*
Outcome to be predicted:	*Cancer specific mortality*

Step 2: Classify the type of prediction model evaluation

Use the following table to classify the evaluation as model development, model validation or model update, or combination. Different signalling questions apply for different types of prediction model evaluation. If the evaluation does not fit one of these classifications then PROBAST should not be used.

**Classify the evaluation based on its aim**			
**Type of prediction study**	**PROBAST boxes to complete**	**Tick as appropriate**	**Definition for type of prediction model study**
Development only	Development	✓	Prediction model development without external validation. These studies may include internal validation methods, such as bootstrapping and cross-validation techniques.
Development and validation	Development and validation	✗	Prediction model development combined with external validation in other participants in the same article.
Validation only	Validation	✗	External validation of existing (previously developed) model in other participants.

This table should be completed once for each publication being assessed and for each relevant outcome in your review.

**Publication reference**	Xiao R, Liu C, He W, Ma L. Prognostic Factors and a Nomogram Predicting Overall Survival and Cancer-Specific Survival for Patients with Collecting Duct Renal Cell Carcinoma. Biomed Res Int. 2021 11;2021:6736008.
**Models of interest**	Nomogram
**Outcome of interest**	*Cancer specific mortality*

Step 3: Assess risk of bias and applicability

PROBAST is structured as four key domains. Each domain is judged for risk of bias (low, high or unclear) and includes signalling questions to help make judgements. Signalling questions are rated as yes (Y), probably yes (PY), probably no (PN), no (N) or no information (NI). All signalling questions are phrased so that “yes” indicates absence of bias. Any signalling question rated as “no” or “probably no” flags the potential for bias; you will need to use your judgement to determine whether the domain should be rated as “high”, “low” or “unclear” risk of bias. The guidance document contains further instructions and examples on rating signalling questions and risk of bias for each domain.

The first three domains are also rated for concerns regarding applicability (low/ high/ unclear) to your review question defined above.

Complete all domains separately for each evaluation of a distinct model. Shaded boxes indicate where signalling questions do not apply and should not be answered.

<table>
<tr><td colspan="4">DOMAIN 1: Participants</td></tr>
<tr><td colspan="4">A. Risk of Bias</td></tr>
<tr><td colspan="4">Describe the sources of data and criteria for participant selection:
“Collecting duct renal cell carcinoma (CDRCC) is a rare type of renal cancer characterized by a poor prognosis. The aim of this work was to develop a nomogram predicting the overall survival (OS) and cancer-specific survival (CSS) for patients with CDRCC.”
“Patients diagnosed with CDRCC (histological diagnostic code 8319/03 in the International Classification of Diseases for Oncology, 3rd Edition (ICD-O-3)) from 2004 to 2015 were included in this study”</td></tr>
<tr><td colspan="2"></td><td>Dev</td><td>Val</td></tr>
<tr><td colspan="2">1.1 Were appropriate data sources used, e.g. cohort, RCT or nested case-control study data?</td><td>N</td><td>N/A</td></tr>
<tr><td colspan="2">1.2 Were all inclusions and exclusions of participants appropriate?</td><td>Y</td><td>N/A</td></tr>
<tr><td>Risk of bias introduced by selection of participants</td><td>RISK:
(low/ high/ unclear)</td><td>High</td><td>N/A</td></tr>
<tr><td colspan="4">Rationale of bias rating:
The authors excluded patients with other types of renal cancer.</td></tr>
<tr><td colspan="4">B. Applicability</td></tr>
<tr><td colspan="4">Describe included participants, setting and dates:
“Collecting duct renal cell carcinoma (CDRCC) is a rare type of renal cancer characterized by a poor prognosis. The aim of this work was to develop a nomogram predicting the overall survival (OS) and cancer-specific survival (CSS) for patients with CDRCC.”
“Patients diagnosed with CDRCC (histological diagnostic code 8319/03 in the International Classification of Diseases for Oncology, 3rd Edition (ICD-O-3)) from 2004 to 2015 were included in this study”</td></tr>
<tr><td>Concern that the included participants and setting do not match the review question</td><td>CONCERN:
(low/ high/ unclear)</td><td>High</td><td>N/A</td></tr>
<tr><td colspan="4">Rationale of applicability rating:
The authors excluded patients with other types of renal cancer.</td></tr>
</table>

<table>
<tr><th colspan="4">DOMAIN 2: Predictors</th></tr>
<tr><th colspan="4">A. Risk of Bias</th></tr>
<tr><td colspan="4">List and describe predictors included in the final model, e.g. definition and timing of assessment:

The predictors included in the model were: tumor size, grade, N and M stage, surgical type and chemotherapy.

All the predictors were measured at diagnosis or after the treatment</td></tr>
<tr><td colspan="2"></td><td>Dev</td><td>Val</td></tr>
<tr><td colspan="2">2.1 Were predictors defined and assessed in a similar way for all participants?</td><td>N</td><td>N/A</td></tr>
<tr><td colspan="2">2.2 Were predictor assessments made without knowledge of outcome data?</td><td>PY</td><td>N/A</td></tr>
<tr><td colspan="2">2.3 Are all predictors available at the time the model is intended to be used?</td><td>PY</td><td>N/A</td></tr>
<tr><td>Risk of bias introduced by predictors or their assessment</td><td>RISK:
(low/ high/ unclear)</td><td>High</td><td>N/A</td></tr>
<tr><td colspan="4">Rationale of bias rating:
Although the model not clearly say the moment of the administration of chemotherapy and radiotherapy the investigators supposed that was before the radical cystectomy based on clinical experience.
They made a comparison of outcomes by treatment received, although SEER database managers say that this analysis would NOT be supported by the RT/chemotherapy data, due to the incompleteness of the variable (Chemotherapy data are categorized as either “yes – patient had chemotherapy” or “no/unknown – no evidence of chemotherapy was found in the medical records examined”).</td></tr>
<tr><th colspan="4">B. Applicability</th></tr>
<tr><td>Concern that the definition, assessment or timing of predictors in the model do not match the review question</td><td>CONCERN:
(low/ high/ unclear)</td><td>Low</td><td>N/A</td></tr>
<tr><td colspan="4">Rationale of applicability rating:
No major issues identified.</td></tr>
</table>

<table>
<tr><th colspan="4">DOMAIN 3: Outcome</th></tr>
<tr><th colspan="4">A. Risk of Bias</th></tr>
<tr><td colspan="4">Describe the outcome, how it was defined and determined, and the time interval between predictor assessment and outcome determination:
The outcome was cancer specific survival at 1, 3 and 5 years</td></tr>
<tr><td colspan="2"></td><td>Dev</td><td>Val</td></tr>
<tr><td colspan="2">3.1 Was the outcome determined appropriately?</td><td>PY</td><td>N/A</td></tr>
<tr><td colspan="2">3.2 Was a pre-specified or standard outcome definition used?</td><td>Y</td><td>N/A</td></tr>
<tr><td colspan="2">3.3 Were predictors excluded from the outcome definition?</td><td>PY</td><td>N/A</td></tr>
<tr><td colspan="2">3.4 Was the outcome defined and determined in a similar way for all participants?</td><td>PY</td><td>N/A</td></tr>
<tr><td colspan="2">3.5 Was the outcome determined without knowledge of predictor information?</td><td>PY</td><td>N/A</td></tr>
<tr><td colspan="2">3.6 Was the time interval between predictor assessment and outcome determination appropriate?</td><td>Y</td><td>N/A</td></tr>
<tr><td>Risk of bias introduced by the outcome or its determination</td><td>RISK:
(low/ high/ unclear)</td><td>Low</td><td>N/A</td></tr>
<tr><td colspan="4">Rationale of bias rating:
No major issues identified</td></tr>
<tr><th colspan="4">B. Applicability</th></tr>
<tr><td colspan="4">At what time point was the outcome determined:
1, 3 and 5 years

If a composite outcome was used, describe the relative frequency/distribution of each contributing outcome:
N/A</td></tr>
<tr><td>Concern that the outcome, its definition, timing or determination do not match the review question</td><td>CONCERN:
(low/ high/ unclear)</td><td>Low</td><td>N/A</td></tr>
<tr><td colspan="4">Rationale of applicability rating:
The outcome of the primary study matches the outcome of interest of the review</td></tr>
</table>

DOMAIN 4: Analysis		
**Risk of Bias**		
Describe numbers of participants, number of candidate predictors, outcome events and events per candidate predictor:<br>"Patients diagnosed with CDRCC (histological diagnostic code 8319/03 in the International Classification of Diseases for Oncology, 3rd Edition (ICD-O-3)) from 2004 to 2015 were included in this study. Finally, 324 eligible patients were included for further analysis."<br><br>The total number of predictors is 23 (table 2)<br>The number of events is 208<br>EPV= 209/23=9.04		
Describe how the model was developed (for example in regards to modelling technique (e.g. survival or logistic modelling), predictor selection, and risk group definition):<br><br>"The Kaplan-Meier method was used to estimate the 1-year, 3-year, and 5-year OS and CSS in the study cohort. Univariable and multivariable Cox proportional hazard regressions were performed to identify the independent prognostic factors associated with OS and CSS (forward stepwise selection methods). The selected independent factors were incorporated in the nomograms to predict the probability of 1-year, 3-year, and 5-year OS and CSS"		
Describe whether and how the model was validated, either internally (e.g. bootstrapping, cross validation, random split sample) or externally (e.g. temporal validation, geographical validation, different setting, different type of participants):<br><br>"The calibration was evaluated using a calibration curve, which was assessed between the observed outcome probability and the nomogram-predicted probability, with a bootstrap resample of 1000 times"		
Describe the performance measures of the model, e.g. (re)calibration, discrimination, (re)classification, net benefit, and whether they were adjusted for optimism:<br><br>"The discrimination of the nomogram was measured by the concordance index (Cindex), which ranges from 0.5 (no predictive power) to 1 (perfect prediction) [17]. The Kaplan-Meier curve and logrank test were also performed to evaluate the ability of the risk stratification of the nomogram associated with OS and CSS. The calibration was evaluated using a calibration curve, which was assessed between the observed outcome probability and the nomogram-predicted probability, with a bootstrap resample of 1000 times"		
Describe any participants who were excluded from the analysis:<br><br>"Patients with missing data on baseline characteristics and follow-up were excluded"		
Describe missing data on predictors and outcomes as well as methods used for missing data:<br><br>"Patients with missing data on baseline characteristics and follow-up were excluded"		
	Dev	Val
4.1 Were there a reasonable number of participants with the outcome?	N	N/A
4.2 Were continuous and categorical predictors handled appropriately?	Y	N/A
4.3 Were all enrolled participants included in the analysis?	Y	N/A
4.4 Were participants with missing data handled appropriately?	N	N/A
4.5 Was selection of predictors based on univariable analysis avoided?	N	

4.6 Were complexities in the data (e.g. censoring, competing risks, sampling of controls) accounted for appropriately?		N	N/A
4.7 Were relevant model performance measures evaluated appropriately?		Y	N/A
4.8 Were model overfitting and optimism in model performance accounted for?		Y	
4.9 Do predictors and their assigned weights in the final model correspond to the results from multivariable analysis?		PY	
**Risk of bias introduced by the analysis**	**RISK:** *(low/ high/ unclear)*	High	N/A
Rationale of bias rating: *They didn´t have enough patients with the outcome. They excluded patients with missing data. They selected the predictors based on univariable. They didn't use competing risks.*			

Step 4: Overall assessment

Use the following tables to reach overall judgements about risk of bias and concerns regarding applicability of the prediction model evaluation (development and/or validation) across all assessed domains.
Complete for each evaluation of a distinct model.

**Reaching an overall judgement about risk of bias of the prediction model evaluation**	
**Low risk of bias**	If all domains were rated low risk of bias.<br>If a prediction model was developed without any external validation, and it was rated as low risk of bias for all domains, consider downgrading to **high risk of bias**. Such a model can only be considered as low risk of bias, if the development was based on a very large data set and included some form of internal validation.
**High risk of bias**	If at least one domain is judged to be at **high risk of bias**.
**Unclear risk of bias**	If an unclear risk of bias was noted in at least one domain and it was low risk for all other domains.

**Reaching an overall judgement about applicability of the prediction model evaluation**	
**Low concerns regarding applicability**	If low concerns regarding applicability for all domains, the prediction model evaluation is judged to have **low concerns regarding applicability**.
**High concerns regarding applicability**	If high concerns regarding applicability for at least one domain, the prediction model evaluation is judged to have **high concerns regarding applicability**.
**Unclear concerns regarding applicability**	If unclear concerns (but no "high concern") regarding applicability for at least one domain, the prediction model evaluation is judged to have **unclear concerns regarding applicability** overall.

**Overall judgement about risk of bias and applicability of the prediction model evaluation**		
**Overall judgement of risk of bias**	**RISK:**<br>*(low/ high/ unclear)*	High
Summary of sources of potential bias:<br>Participants, predictors and analysis domains show several major issues		
**Overall judgement of applicability**	**CONCERN:**<br>*(low/ high/ unclear)*	Low
Summary of applicability concerns:<br><br>Participants domain shows several major issues		

PROBAST

(Prediction model study Risk Of Bias Assessment Tool)

Published in Annals of Internal Medicine (freely available):

1. PROBAST: A Tool to Assess the Risk of Bias and Applicability of Prediction Model Studies
2. PROBAST: A Tool to Assess Risk of Bias and Applicability of Prediction Model Studies: Explanation and Elaboration

What does PROBAST assess?

PROBAST assesses both the *risk of bias* and *concerns regarding applicability* of a study that evaluates (develops, validates or updates) a multivariable diagnostic or prognostic prediction model. It is designed to assess primary studies included in a systematic review.

Bias occurs if systematic flaws or limitations in the design, conduct or analysis of a primary study distort the results. For the purpose of prediction modelling studies, we have defined *risk of bias* to occur when shortcomings in the study design, conduct or analysis lead to systematically distorted estimates of a model's predictive performance or to an inadequate model to address the research question. Model predictive performance is typically evaluated using calibration, discrimination and sometimes classification measures, and these are likely inaccurately estimated in studies with high risk of bias. *Applicability* refers to the extent to which the prediction model from the primary study matches your systematic review question, for example in terms of the participants, predictors or outcome of interest.

A primary study may include the development and/or validation or update of more than one prediction model. A PROBAST assessment should be completed for each distinct model that is developed, validated or updated (extended) for making individualised predictions. Where a publication assesses multiple prediction models, only complete a PROBAST assessment for those models that meet the inclusion criteria for your systematic review. Please note that subsequent use of the term "model" includes derivatives of models, such as simplified risk scores, nomograms, or recalibrations of models.

PROBAST is not designed for all multivariable diagnostic or prognostic studies. For example, studies using multivariable models to identify predictors associated with an outcome but not attempting to develop a model for making individualised predictions are not covered by PROBAST.

PROBAST includes four steps.

Step	Task	When to complete
**1**	Specify your systematic review question(s)	Once per systematic review
**2**	Classify the type of prediction model evaluation	Once for each model of interest in each publication being assessed, for each relevant outcome
**3**	Assess risk of bias and applicability	Once for each development and validation of each distinct prediction model in a publication
**4**	Overall judgment	Once for each development and validation of each distinct prediction model in a publication

If this is your first time using PROBAST, we strongly recommend reading the detailed explanation and elaboration (E&E, see link above) paper and to check the examples on www.probast.org

Step 1: Specify your systematic review question

State your systematic review question to facilitate the assessment of the applicability of the evaluated models to your question. *The following table should be completed once per systematic review.*

Criteria	Specify your systematic review question
Intended use of model:	*To predict cancer specific mortality in patients with renal cancer treated with partial or total nephrectomy regardless of TNM stage*
***Participants** including selection criteria and setting:*	*Patients with renal cancer treated with partial or total nephrectomy regardless of TNM stage*
***Predictors** (used in prediction modelling), including types of predictors (e.g. history, clinical examination, biochemical markers, imaging tests), time of measurement, specific measurement issues (e.g., any requirements/ prohibitions for specialized equipment):*	*Predictors used in clinical practice measured when a nephrectomy for renal cancer is indicated*
Outcome to be predicted:	*Cancer specific mortality*

Step 2: Classify the type of prediction model evaluation

Use the following table to classify the evaluation as model development, model validation or model update, or combination. Different signalling questions apply for different types of prediction model evaluation. If the evaluation does not fit one of these classifications then PROBAST should not be used.

**Classify the evaluation based on its aim**			
**Type of prediction study**	**PROBAST boxes to complete**	**Tick as appropriate**	**Definition for type of prediction model study**
Development only	Development	✗	Prediction model development without external validation. These studies may include internal validation methods, such as bootstrapping and cross-validation techniques.
Development and validation	Development and validation	✓	Prediction model development combined with external validation in other participants in the same article.
Validation only	Validation	✗	External validation of existing (previously developed) model in other participants.

This table should be completed once for each publication being assessed and for each relevant outcome in your review.

**Publication reference**	Tian S, Sun S, Mao W, Qian S, Zhang L, Zhang G, Xu B, Chen M. Development and Validation of Prognostic Nomogram for Young Patients with Kidney Cancer. Int J Gen Med. 2021; 1:14:5091-5103
**Models of interest**	Nomogram
**Outcome of interest**	*Cancer specific mortality*

Step 3: Assess risk of bias and applicability

PROBAST is structured as four key domains. Each domain is judged for risk of bias (low, high or unclear) and includes signalling questions to help make judgements. Signalling questions are rated as yes (Y), probably yes (PY), probably no (PN), no (N) or no information (NI). All signalling questions are phrased so that "yes" indicates absence of bias. Any signalling question rated as "no" or "probably no" flags the potential for bias; you will need to use your judgement to determine whether the domain should be rated as "high", "low" or "unclear" risk of bias. The guidance document contains further instructions and examples on rating signalling questions and risk of bias for each domain.

The first three domains are also rated for concerns regarding applicability (low/ high/ unclear) to your review question defined above.

Complete all domains separately for each evaluation of a distinct model. Shaded boxes indicate where signalling questions do not apply and should not be answered.

DOMAIN 1: Participants			
**A. Risk of Bias**			
Describe the sources of data and criteria for participant selection:<br><br>*"Clinical data of EOKC patients from 2004 to 2015 were retrieved from SEER using SEER*Stat software. Because the patient information in the SEER database is anonymized and publicly available, our study was not monitored by an institutional review board. The exclusion criteria included: (I) age >46 years old; (II) incomplete TNM stage information; (III) multiple primary tumor lesions; (IV) incomplete survival data; (V) not having undergone surgical treatment (Figure 1). The training group accounted for two-thirds of the total, and the included EOKCs were randomly assigned to these two sets"*			
		Dev	Val
1.1 Were appropriate data sources used, e.g. cohort, RCT or nested case-control study data?		Y	Y
1.2 Were all inclusions and exclusions of participants appropriate?		Y	Y
**Risk of bias introduced by selection of participants**	**RISK:**<br>*(low/ high/ unclear)*	Low	Low
Rationale of bias rating:<br>*No major issues identified*			
**B. Applicability**			
Describe included participants, setting and dates:<br>*"Clinical data of EOKC patients from 2004 to 2015 were retrieved from SEER using SEER*Stat software. Because the patient information in the SEER database is anonymized and publicly available, our study was not monitored by an institutional review board. The exclusion criteria included: (I) age >46 years old; (II) incomplete TNM stage information; (III) multiple primary tumor lesions; (IV) incomplete survival data; (V) not having undergone surgical treatment (Figure 1). The training group accounted for two-thirds of the total, and the included EOKCs were randomly assigned to these two sets"*			
**Concern that the included participants and setting do not match the review question**	**CONCERN:**<br>*(low/ high/ unclear)*	Low	Low
Rationale of applicability rating:<br>*No major issues identified*			

DOMAIN 2: Predictors			
**A. Risk of Bias**			
List and describe predictors included in the final model, e.g. definition and timing of assessment:<br><br>The predictors included in the model were: age, race, grade, AJCC stage, TNM stage, histology, chemotherapy and radiotherapy<br>All the predictors were measured after treatment			
		Dev	Val
2.1 Were predictors defined and assessed in a similar way for all participants?		N	N
2.2 Were predictor assessments made without knowledge of outcome data?		PY	PY
2.3 Are all predictors available at the time the model is intended to be used?		PY	PY
**Risk of bias introduced by predictors or their assessment**	**RISK:**<br>*(low/ high/ unclear)*	High	High
Rationale of bias rating:<br>Although the model not clearly say the moment of the administration of chemotherapy and radiotherapy the investigators supposed that was before the radical cystectomy based on clinical experience.<br>*They made a comparison of outcomes by treatment received, although SEER database managers say that this analysis would NOT be supported by the RT/chemotherapy data, due to the incompleteness of the variable (Chemotherapy data are categorized as either "yes – patient had chemotherapy" or "no/unknown – no evidence of chemotherapy was found in the medical records examined")*			
**B. Applicability**			
Concern that the definition, assessment or timing of predictors in the model do not match the review question	**CONCERN:**<br>*(low/ high/ unclear)*	Low	Low
Rationale of applicability rating:<br>*No major issues identified.*			

DOMAIN 3: Outcome			
**A. Risk of Bias**			
Describe the outcome, how it was defined and determined, and the time interval between predictor assessment and outcome determination:<br><br>The outcome was cancer specific survival at 3 and 5 years<br>"CSS was defined as the date of diagnosis to the date of death from kidney cancer"			
		Dev	Val
3.1 Was the outcome determined appropriately?		PY	PY
3.2 Was a pre-specified or standard outcome definition used?		Y	Y
3.3 Were predictors excluded from the outcome definition?		PY	PY
3.4 Was the outcome defined and determined in a similar way for all participants?		PY	PY
3.5 Was the outcome determined without knowledge of predictor information?		PY	PY
3.6 Was the time interval between predictor assessment and outcome determination appropriate?		Y	Y
**Risk of bias introduced by the outcome or its determination**	**RISK:**<br>*(low/ high/ unclear)*	Low	Low
Rationale of bias rating:<br>*No major issues identified*			
**B. Applicability**			
At what time point was the outcome determined:<br>3 and 5 years<br><br>*If a composite outcome was used, describe the relative frequency/distribution of each contributing outcome:*<br>*N/A*			
**Concern that the outcome, its definition, timing or determination do not match the review question**	**CONCERN:**<br>*(low/ high/ unclear)*	Low	Low
Rationale of applicability rating:<br>*The outcome of the primary study matches the outcome of interest of the review*			

**DOMAIN 4: Analysis**
**Risk of Bias**
Describe numbers of participants, number of candidate predictors, outcome events and events per candidate predictor:<br>*"A total of 12,526 EOKC were included in this study, 8350 of whom were assigned to the training set"*<br><br>*A total of 25 predictors were included (Table 2)*<br>*The number of events is unknown*<br><br>*EPV= unknown*
Describe how the model was developed (for example in regards to modelling technique (e.g. survival or logistic modelling), predictor selection, and risk group definition):<br><br>*"In the training set, univariate and multivariate Cox regressions were used to analyze the factors associated with OS and CSS, and the results were presented as hazard ratios (HR) and 95% confidence interval (CI). Based on the results of multivariate Cox regression analysis, we constructed patient OS and CSS prognostic nomograms"*
Describe whether and how the model was validated, either internally (e.g. bootstrapping, cross validation, random split sample) or externally (e.g. temporal validation, geographical validation, different setting, different type of participants):<br><br>*Not indicated*
Describe the performance measures of the model, e.g. (re)calibration, discrimination, (re)classification, net benefit, and whether they were adjusted for optimism:<br><br>*"In addition, C-index, ROC curves, DCA curves, and internal validation were used to assess the predictive performance of nomograms"*
Describe any participants who were excluded from the analysis:<br>*"The exclusion criteria included: (I) age >46 years old; (II) incomplete TNM stage information; (III) multiple primary tumor lesions; (IV) incomplete survival data; (V) not having undergone surgical treatment"*
Describe missing data on predictors and outcomes as well as methods used for missing data:<br>*"The exclusion criteria included: (I) age >46 years old; (II) incomplete TNM stage information; (III) multiple primary tumor lesions; (IV) incomplete survival data; (V) not having undergone surgical treatment"*

	Dev	Val
4.1 Were there a reasonable number of participants with the outcome?	NI	PY
4.2 Were continuous and categorical predictors handled appropriately?	N	N
4.3 Were all enrolled participants included in the analysis?	Y	Y
4.4 Were participants with missing data handled appropriately?	N	N
4.5 Was selection of predictors based on univariable analysis avoided?	N	
4.6 Were complexities in the data (e.g. censoring, competing risks, sampling of controls) accounted for appropriately?	N	N
4.7 Were relevant model performance measures evaluated appropriately?	Y	Y
4.8 Were model overfitting and optimism in model performance accounted for?	N	
4.9 Do predictors and their assigned weights in the final model correspond to the	PY	

results from multivariable analysis?			
**Risk of bias introduced by the analysis**	**RISK:** *(low/ high/ unclear)*	High	High
Rationale of bias rating: *They didn´t say the number of events in each cohort, but according the number of total events probably the are enough events per variable on the validation cohort. They did categorizations. They excluded patients with missing data. They selected the predictors based on univariable. They didn't use competing risks. They didn´t do bootstrapping*			

Step 4: Overall assessment

Use the following tables to reach overall judgements about risk of bias and concerns regarding applicability of the prediction model evaluation (development and/or validation) across all assessed domains.
Complete for each evaluation of a distinct model.

**Reaching an overall judgement about risk of bias of the prediction model evaluation**	
**Low risk of bias**	If all domains were rated low risk of bias.<br>If a prediction model was developed without any external validation, and it was rated as low risk of bias for all domains, consider downgrading to **high risk of bias**. Such a model can only be considered as low risk of bias, if the development was based on a very large data set and included some form of internal validation.
**High risk of bias**	If at least one domain is judged to be at **high risk of bias**.
**Unclear risk of bias**	If an unclear risk of bias was noted in at least one domain and it was low risk for all other domains.

**Reaching an overall judgement about applicability of the prediction model evaluation**	
**Low concerns regarding applicability**	If low concerns regarding applicability for all domains, the prediction model evaluation is judged to have **low concerns regarding applicability**.
**High concerns regarding applicability**	If high concerns regarding applicability for at least one domain, the prediction model evaluation is judged to have **high concerns regarding applicability**.
**Unclear concerns regarding applicability**	If unclear concerns (but no “high concern”) regarding applicability for at least one domain, the prediction model evaluation is judged to have **unclear concerns regarding applicability** overall.

**Overall judgement about risk of bias and applicability of the prediction model evaluation**		
**Overall judgement of risk of bias**	**RISK:**<br>*(low/ high/ unclear)*	High
Summary of sources of potential bias:<br>Analysis and predictors domains show several major issues		
**Overall judgement of applicability**	**CONCERN:**<br>*(low/ high/ unclear)*	Low
Summary of applicability concerns:<br><br>No major issues		

PROBAST

(Prediction model study Risk Of Bias Assessment Tool)

Published in Annals of Internal Medicine (freely available):

1. PROBAST: A Tool to Assess the Risk of Bias and Applicability of Prediction Model Studies
2. PROBAST: A Tool to Assess Risk of Bias and Applicability of Prediction Model Studies: Explanation and Elaboration

What does PROBAST assess?

PROBAST assesses both the *risk of bias* and *concerns regarding applicability* of a study that evaluates (develops, validates or updates) a multivariable diagnostic or prognostic prediction model. It is designed to assess primary studies included in a systematic review.

Bias occurs if systematic flaws or limitations in the design, conduct or analysis of a primary study distort the results. For the purpose of prediction modelling studies, we have defined *risk of bias* to occur when shortcomings in the study design, conduct or analysis lead to systematically distorted estimates of a model's predictive performance or to an inadequate model to address the research question. Model predictive performance is typically evaluated using calibration, discrimination and sometimes classification measures, and these are likely inaccurately estimated in studies with high risk of bias. *Applicability* refers to the extent to which the prediction model from the primary study matches your systematic review question, for example in terms of the participants, predictors or outcome of interest.

A primary study may include the development and/or validation or update of more than one prediction model. A PROBAST assessment should be completed for each distinct model that is developed, validated or updated (extended) for making individualised predictions. Where a publication assesses multiple prediction models, only complete a PROBAST assessment for those models that meet the inclusion criteria for your systematic review. Please note that subsequent use of the term "model" includes derivatives of models, such as simplified risk scores, nomograms, or recalibrations of models.

PROBAST is not designed for all multivariable diagnostic or prognostic studies. For example, studies using multivariable models to identify predictors associated with an outcome but not attempting to develop a model for making individualised predictions are not covered by PROBAST.

PROBAST includes four steps.

Step	Task	When to complete
**1**	Specify your systematic review question(s)	Once per systematic review
**2**	Classify the type of prediction model evaluation	Once for each model of interest in each publication being assessed, for each relevant outcome
**3**	Assess risk of bias and applicability	Once for each development and validation of each distinct prediction model in a publication
**4**	Overall judgment	Once for each development and validation of each distinct prediction model in a publication

If this is your first time using PROBAST, we strongly recommend reading the detailed explanation and elaboration (E&E, see link above) paper and to check the examples on www.probast.org

Step 1: Specify your systematic review question

State your systematic review question to facilitate the assessment of the applicability of the evaluated models to your question. *The following table should be completed once per systematic review.*

Criteria	Specify your systematic review question
Intended use of model:	*To predict cancer specific mortality in patients with renal cancer treated with partial or total nephrectomy regardless of TNM stage*
***Participants** including selection criteria and setting:*	*Patients with renal cancer treated with partial or total nephrectomy regardless of TNM stage*
***Predictors** (used in prediction modelling), including types of predictors (e.g. history, clinical examination, biochemical markers, imaging tests), time of measurement, specific measurement issues (e.g., any requirements/ prohibitions for specialized equipment):*	*Predictors used in clinical practice measured when a nephrectomy for renal cancer is indicated*
Outcome to be predicted:	*Cancer specific mortality*

Step 2: Classify the type of prediction model evaluation

Use the following table to classify the evaluation as model development, model validation or model update, or combination. Different signalling questions apply for different types of prediction model evaluation. If the evaluation does not fit one of these classifications then PROBAST should not be used.

**Classify the evaluation based on its aim**			
**Type of prediction study**	**PROBAST boxes to complete**	**Tick as appropriate**	**Definition for type of prediction model study**
Development only	Development	✓	Prediction model development without external validation. These studies may include internal validation methods, such as bootstrapping and cross-validation techniques.
Development and validation	Development and validation	✗	Prediction model development combined with external validation in other participants in the same article.
Validation only	Validation	✗	External validation of existing (previously developed) model in other participants.

This table should be completed once for each publication being assessed and for each relevant outcome in your review.

**Publication reference**	Leibovich BC, Han K, Bui MHT, Pantuck AJ, Dorey FJ, Figlin RA Belldegrun A. Scoring algorithm to predict survival after nephrectomy and immunotherapy in patients with metastatic renal cell carcinoma: a stratification tool for prospective clinical trials. Cancer. 2003. 15;98(12):2566-75
**Models of interest**	Risk score
**Outcome of interest**	*Cancer specific mortality*

Step 3: Assess risk of bias and applicability

PROBAST is structured as four key domains. Each domain is judged for risk of bias (low, high or unclear) and includes signalling questions to help make judgements. Signalling questions are rated as yes (Y), probably yes (PY), probably no (PN), no (N) or no information (NI). All signalling questions are phrased so that “yes” indicates absence of bias. Any signalling question rated as “no” or “probably no” flags the potential for bias; you will need to use your judgement to determine whether the domain should be rated as “high”, “low” or “unclear” risk of bias. The guidance document contains further instructions and examples on rating signalling questions and risk of bias for each domain.

The first three domains are also rated for concerns regarding applicability (low/ high/ unclear) to your review question defined above.

Complete all domains separately for each evaluation of a distinct model. Shaded boxes indicate where signalling questions do not apply and should not be answered.

DOMAIN 1: Participants			
**A. Risk of Bias**			
Describe the sources of data and criteria for participant selection:<br><br>"Patients with non-RCC renal tumors or inheritable forms of RCC, including hereditary papillary, von Hippel–Lindau, and tuberous sclerosis syndromes, were excluded. To eliminate possible confounding factors, the cohort was limited further to patients with metastases at presentation who were treated with radical nephrectomy and immunotherapy between 1989 and 2000. Patients received recombinant IL-2-based immunotherapy regimens within the framework of 11 clinical trials. Patients who received non-IL-2-based biologic response modifiers or chemotherapeutic regimens were excluded. After the exclusions, there were 173 patients with metastatic RCC at presentation who were treated with nephrectomy and IL-2-based immunotherapy available for study"			
		Dev	Val
1.1 Were appropriate data sources used, e.g. cohort, RCT or nested case-control study data?		Y	N/A
1.2 Were all inclusions and exclusions of participants appropriate?		N	N/A
**Risk of bias introduced by selection of participants**	**RISK:**<br>*(low/ high/ unclear)*	High	N/A
Rationale of bias rating:<br>They excluded patients with non-RCC renal tumors or inheritable forms of RCC (including hereditary papillary, von Hippel–Lindau, and tuberous sclerosis syndromes) and patients who received non-IL-2-based biologic response modifiers or chemotherapeutic regimens			
**B. Applicability**			
Describe included participants, setting and dates:<br>"Patients with non-RCC renal tumors or inheritable forms of RCC, including hereditary papillary, von Hippel–Lindau, and tuberous sclerosis syndromes, were excluded. To eliminate possible confounding factors, the cohort was limited further to patients with metastases at presentation who were treated with radical nephrectomy and immunotherapy between 1989 and 2000. Patients received recombinant IL-2-based immunotherapy regimens within the framework of 11 clinical trials. Patients who received non-IL-2-based biologic response modifiers or chemotherapeutic regimens were excluded. After the exclusions, there were 173 patients with metastatic RCC at presentation who were treated with nephrectomy and IL-2-based immunotherapy available for study"			
**Concern that the included participants and setting do not match the review question**	**CONCERN:**<br>*(low/ high/ unclear)*	High	N/A
Rationale of applicability rating:<br>They excluded patients with non-RCC renal tumors or inheritable forms of RCC (including hereditary papillary, von Hippel–Lindau, and tuberous sclerosis syndromes) and patients who received non-IL-2-based biologic response modifiers or chemotherapeutic regimens			

DOMAIN 2: Predictors			
**A. Risk of Bias**			
List and describe predictors included in the final model, e.g. definition and timing of assessment:<br><br>The predictors included in the model were: N stage, constitutional symptoms, metastases location, sarcomatoid histology and thyroid-stimulating hormone<br><br>All the predictors were measured at diagnosis and after the treatment			
		Dev	Val
2.1 Were predictors defined and assessed in a similar way for all participants?		PY	N/A
2.2 Were predictor assessments made without knowledge of outcome data?		PY	N/A
2.3 Are all predictors available at the time the model is intended to be used?		PY	N/A
**Risk of bias introduced by predictors or their assessment**	**RISK:**<br>*(low/ high/ unclear)*	Low	N/A
Rationale of bias rating:<br>*The blinding of measurement is unknown but all the predictors are objective.*			
**B. Applicability**			
Concern that the definition, assessment or timing of predictors in the model do not match the review question	**CONCERN:**<br>*(low/ high/ unclear)*	Low	N/A
Rationale of applicability rating:<br>*No major issues identified.*			

DOMAIN 3: Outcome			
**A. Risk of Bias**			
Describe the outcome, how it was defined and determined, and the time interval between predictor assessment and outcome determination:<br>The outcome was cancer specific survival at 1, 3 and 5 years			
		Dev	Val
3.1 Was the outcome determined appropriately?		PY	N/A
3.2 Was a pre-specified or standard outcome definition used?		Y	N/A
3.3 Were predictors excluded from the outcome definition?		PY	N/A
3.4 Was the outcome defined and determined in a similar way for all participants?		PY	N/A
3.5 Was the outcome determined without knowledge of predictor information?		PY	N/A
3.6 Was the time interval between predictor assessment and outcome determination appropriate?		Y	N/A
**Risk of bias introduced by the outcome or its determination**	**RISK:**<br>*(low/ high/ unclear)*	Low	N/A
Rationale of bias rating:<br>*No major issues identified*			
**B. Applicability**			
At what time point was the outcome determined:<br>1,3 and 5 years<br><br>*If a composite outcome was used, describe the relative frequency/distribution of each contributing outcome:*<br>*N/A*			
**Concern that the outcome, its definition, timing or determination do not match the review question**	**CONCERN:**<br>*(low/ high/ unclear)*	Low	N/A
Rationale of applicability rating:<br>*The outcome of the primary study matches the outcome of interest of the review*			

DOMAIN 4: Analysis

Risk of Bias

Describe numbers of participants, number of candidate predictors, outcome events and events per candidate predictor:

"After the exclusions, there were 173 patients with metastatic RCC at presentation who were treated with nephrectomy and IL-2-based immunotherapy available for study"

The total number of predictors is 19 (table 1)
The number of events is 123

EPV= 123/19=6.47

Describe how the model was developed (for example in regards to modelling technique (e.g. survival or logistic modelling), predictor selection, and risk group definition):

"Disease-specific survival was estimated using the Kaplan–Meier method. There were no non-RCC-related deaths in this cohort; therefore, overall survival and disease-specific survival were the same. The duration of follow-up was calculated from the date of radical nephrectomy to the date of death or last follow-up. Univariate and multivariate Cox proportional hazards models were fit to determine the clinical, surgical, and pathologic features that were associated significantly with death from RCC. A multivariate model was built using a stepwise selection procedure with the *P* value for a feature to enter the model set to 0.15. The resulting Cox model was then reduced to include only variables significant at the 5% level. The hazard ratios (HR) from the resulting model were used to define a simple scoring system"

Describe whether and how the model was validated, either internally (e.g. bootstrapping, cross validation, random split sample) or externally (e.g. temporal validation, geographical validation, different setting, different type of participants):

Not indicated

Describe the performance measures of the model, e.g. (re)calibration, discrimination, (re)classification, net benefit, and whether they were adjusted for optimism:

"Internal validation by calibration of the simplified SANI score risk groups was evaluated by comparing the mean of the predicted 1-year, 2-year, and 3-year survivorship predicted by the SANI score groupings with the Kaplan–Meier estimates for several well known prognostic subgroups (Table 4)"

Describe any participants who were excluded from the analysis:
"Patients with non-RCC renal tumors or inheritable forms of RCC, including hereditary papillary, von Hippel–Lindau, and tuberous sclerosis syndromes, were excluded".
"Patients who received non-IL-2-based biologic response modifiers or chemotherapeutic regimens were excluded. After the exclusions, there were 173 patients with metastatic RCC at presentation who were treated with nephrectomy and IL-2-based immunotherapy available for study"

Describe missing data on predictors and outcomes as well as methods used for missing data:
Not indicated

	Dev	Val
4.1 Were there a reasonable number of participants with the outcome?	N	N/A
4.2 Were continuous and categorical predictors handled appropriately?	N	N/A

4.3 Were all enrolled participants included in the analysis?		Y	N/A
4.4 Were participants with missing data handled appropriately?		NI	N/A
4.5 Was selection of predictors based on univariable analysis avoided?		N	
4.6 Were complexities in the data (e.g. censoring, competing risks, sampling of controls) accounted for appropriately?		N	N/A
4.7 Were relevant model performance measures evaluated appropriately?		N	N/A
4.8 Were model overfitting and optimism in model performance accounted for?		N	
4.9 Do predictors and their assigned weights in the final model correspond to the results from multivariable analysis?		PY	
**Risk of bias introduced by the analysis**	**RISK:** *(low/ high/ unclear)*	High	N/A
Rationale of bias rating: *They didn´t have enough patients with the outcome. They did categorizations. They didn´t say anything about missing data. They selected the predictors based on univariable. They didn´t do validation or discrimination. They didn't use competing risks.*			

Step 4: Overall assessment

Use the following tables to reach overall judgements about risk of bias and concerns regarding applicability of the prediction model evaluation (development and/or validation) across all assessed domains.
Complete for each evaluation of a distinct model.

Reaching an overall judgement about risk of bias of the prediction model evaluation	
**Low risk of bias**	If all domains were rated low risk of bias.<br>If a prediction model was developed without any external validation, and it was rated as low risk of bias for all domains, consider downgrading to **high risk of bias**. Such a model can only be considered as low risk of bias, if the development was based on a very large data set and included some form of internal validation.
**High risk of bias**	If at least one domain is judged to be at **high risk of bias**.
**Unclear risk of bias**	If an unclear risk of bias was noted in at least one domain and it was low risk for all other domains.

Reaching an overall judgement about applicability of the prediction model evaluation	
**Low concerns regarding applicability**	If low concerns regarding applicability for all domains, the prediction model evaluation is judged to have **low concerns regarding applicability**.
**High concerns regarding applicability**	If high concerns regarding applicability for at least one domain, the prediction model evaluation is judged to have **high concerns regarding applicability**.
**Unclear concerns regarding applicability**	If unclear concerns (but no "high concern") regarding applicability for at least one domain, the prediction model evaluation is judged to have **unclear concerns regarding applicability** overall.

Overall judgement about risk of bias and applicability of the prediction model evaluation		
**Overall judgement of risk of bias**	**RISK:** *(low/ high/ unclear)*	High
Summary of sources of potential bias:<br>Analysis and participants domains show several major issues		
**Overall judgement of applicability**	**CONCERN:** *(low/ high/ unclear)*	High
Summary of applicability concerns:<br><br>Participants domain shows several major issues		

PROBAST

(Prediction model study Risk Of Bias Assessment Tool)

Published in Annals of Internal Medicine (freely available):

1. PROBAST: A Tool to Assess the Risk of Bias and Applicability of Prediction Model Studies
2. PROBAST: A Tool to Assess Risk of Bias and Applicability of Prediction Model Studies: Explanation and Elaboration

What does PROBAST assess?

PROBAST assesses both the *risk of bias* and *concerns regarding applicability* of a study that evaluates (develops, validates or updates) a multivariable diagnostic or prognostic prediction model. It is designed to assess primary studies included in a systematic review.

Bias occurs if systematic flaws or limitations in the design, conduct or analysis of a primary study distort the results. For the purpose of prediction modelling studies, we have defined *risk of bias* to occur when shortcomings in the study design, conduct or analysis lead to systematically distorted estimates of a model's predictive performance or to an inadequate model to address the research question. Model predictive performance is typically evaluated using calibration, discrimination and sometimes classification measures, and these are likely inaccurately estimated in studies with high risk of bias. *Applicability* refers to the extent to which the prediction model from the primary study matches your systematic review question, for example in terms of the participants, predictors or outcome of interest.

A primary study may include the development and/or validation or update of more than one prediction model. A PROBAST assessment should be completed for each distinct model that is developed, validated or updated (extended) for making individualised predictions. Where a publication assesses multiple prediction models, only complete a PROBAST assessment for those models that meet the inclusion criteria for your systematic review. Please note that subsequent use of the term "model" includes derivatives of models, such as simplified risk scores, nomograms, or recalibrations of models.

PROBAST is not designed for all multivariable diagnostic or prognostic studies. For example, studies using multivariable models to identify predictors associated with an outcome but not attempting to develop a model for making individualised predictions are not covered by PROBAST.

PROBAST includes four steps.

Step	Task	When to complete
**1**	Specify your systematic review question(s)	Once per systematic review
**2**	Classify the type of prediction model evaluation	Once for each model of interest in each publication being assessed, for each relevant outcome
**3**	Assess risk of bias and applicability	Once for each development and validation of each distinct prediction model in a publication
**4**	Overall judgment	Once for each development and validation of each distinct prediction model in a publication

If this is your first time using PROBAST, we strongly recommend reading the detailed explanation and elaboration (E&E, see link above) paper and to check the examples on www.probast.org

Step 1: Specify your systematic review question

State your systematic review question to facilitate the assessment of the applicability of the evaluated models to your question. *The following table should be completed once per systematic review.*

Criteria	Specify your systematic review question
Intended use of model:	*To predict cancer specific mortality in patients with renal cancer treated with partial or total nephrectomy regardless of TNM stage*
***Participants** including selection criteria and setting:*	*Patients with renal cancer treated with partial or total nephrectomy regardless of TNM stage*
***Predictors** (used in prediction modelling), including types of predictors (e.g. history, clinical examination, biochemical markers, imaging tests), time of measurement, specific measurement issues (e.g., any requirements/ prohibitions for specialized equipment):*	*Predictors used in clinical practice measured when a nephrectomy for renal cancer is indicated*
Outcome to be predicted:	*Cancer specific mortality*

Step 2: Classify the type of prediction model evaluation

Use the following table to classify the evaluation as model development, model validation or model update, or combination. Different signalling questions apply for different types of prediction model evaluation. If the evaluation does not fit one of these classifications then PROBAST should not be used.

**Classify the evaluation based on its aim**			
**Type of prediction study**	**PROBAST boxes to complete**	**Tick as appropriate**	**Definition for type of prediction model study**
Development only	Development	✓	Prediction model development without external validation. These studies may include internal validation methods, such as bootstrapping and cross-validation techniques.
Development and validation	Development and validation	✗	Prediction model development combined with external validation in other participants in the same article.
Validation only	Validation	✗	External validation of existing (previously developed) model in other participants.

This table should be completed once for each publication being assessed and for each relevant outcome in your review.

**Publication reference**	Peng D, He ZS, Li XS, Tang Q, Zhang L, Yang KW, Yu XT, Zhang CJ, Zhou LQ. A Novel Predictor of Survival with Renal Cell Carcinoma After Nephrectomy. J Endourol. 2017;31(4):397-404
**Models of interest**	Risk score
**Outcome of interest**	*Cancer specific mortality*

Step 3: Assess risk of bias and applicability

PROBAST is structured as four key domains. Each domain is judged for risk of bias (low, high or unclear) and includes signalling questions to help make judgements. Signalling questions are rated as yes (Y), probably yes (PY), probably no (PN), no (N) or no information (NI). All signalling questions are phrased so that "yes" indicates absence of bias. Any signalling question rated as "no" or "probably no" flags the potential for bias; you will need to use your judgement to determine whether the domain should be rated as "high", "low" or "unclear" risk of bias. The guidance document contains further instructions and examples on rating signalling questions and risk of bias for each domain.

The first three domains are also rated for concerns regarding applicability (low/ high/ unclear) to your review question defined above.

Complete all domains separately for each evaluation of a distinct model. Shaded boxes indicate where signalling questions do not apply and should not be answered.

<table>
<tr><th colspan="4">DOMAIN 1: Participants</th></tr>
<tr><th colspan="4">A. Risk of Bias</th></tr>
<tr><td colspan="4">Describe the sources of data and criteria for participant selection:

“The medical records of 1360 RCC patients who underwent nephrectomy in Peking University First Hospital were retrospectively collected”</td></tr>
<tr><td colspan="2"></td><td>Dev</td><td>Val</td></tr>
<tr><td colspan="2">1.1 Were appropriate data sources used, e.g. cohort, RCT or nested case-control study data?</td><td>Y</td><td>N/A</td></tr>
<tr><td colspan="2">1.2 Were all inclusions and exclusions of participants appropriate?</td><td>Y</td><td>N/A</td></tr>
<tr><td>Risk of bias introduced by selection of participants</td><td>RISK:
(low/ high/ unclear)</td><td>Low</td><td>N/A</td></tr>
<tr><td colspan="4">Rationale of bias rating:
Cohort study with clear inclusion and exclusion criterial</td></tr>
<tr><th colspan="4">B. Applicability</th></tr>
<tr><td colspan="4">Describe included participants, setting and dates:
“The medical records of 1360 RCC patients who underwent nephrectomy in Peking University First Hospital were retrospectively collected”</td></tr>
<tr><td>Concern that the included participants and setting do not match the review question</td><td>CONCERN:
(low/ high/ unclear)</td><td>Low</td><td>N/A</td></tr>
<tr><td colspan="4">Rationale of applicability rating:
Cohort study with clear inclusion and exclusion criterial</td></tr>
</table>

DOMAIN 2: Predictors			
**A. Risk of Bias**			
List and describe predictors included in the final model, e.g. definition and timing of assessment:<br><br>The predictors included in the model were: TNM stage, grade and HALP<br>All the predictors were measured at diagnosis			
		Dev	Val
2.1 Were predictors defined and assessed in a similar way for all participants?		PY	N/A
2.2 Were predictor assessments made without knowledge of outcome data?		PY	N/A
2.3 Are all predictors available at the time the model is intended to be used?		PY	N/A
**Risk of bias introduced by predictors or their assessment**	**RISK:**<br>*(low/ high/ unclear)*	Low	N/A
Rationale of bias rating:<br>*The blinding of measurement is unknown but all the predictors are objective.*			
**B. Applicability**			
Concern that the definition, assessment or timing of predictors in the model do not match the review question	**CONCERN:**<br>*(low/ high/ unclear)*	Low	N/A
Rationale of applicability rating:<br>*No major issues identified.*			

**DOMAIN 3: Outcome**			
**A. Risk of Bias**			
Describe the outcome, how it was defined and determined, and the time interval between predictor assessment and outcome determination:<br>The outcome was cancer specific survival at 5 years			
		Dev	Val
3.1 Was the outcome determined appropriately?		PY	N/A
3.2 Was a pre-specified or standard outcome definition used?		Y	N/A
3.3 Were predictors excluded from the outcome definition?		PY	N/A
3.4 Was the outcome defined and determined in a similar way for all participants?		PY	N/A
3.5 Was the outcome determined without knowledge of predictor information?		PY	N/A
3.6 Was the time interval between predictor assessment and outcome determination appropriate?		Y	N/A
**Risk of bias introduced by the outcome or its determination**	**RISK:**<br>*(low/ high/ unclear)*	Low	N/A
Rationale of bias rating:<br>*No major issues identified*			
**B. Applicability**			
At what time point was the outcome determined:<br>5 years<br><br>*If a composite outcome was used, describe the relative frequency/distribution of each contributing outcome:*<br>*N/A*			
**Concern that the outcome, its definition, timing or determination do not match the review question**	**CONCERN:**<br>*(low/ high/ unclear)*	Low	N/A
Rationale of applicability rating:<br>*The outcome of the primary study matches the outcome of interest of the review*			

DOMAIN 4: Analysis		
**Risk of Bias**		
Describe numbers of participants, number of candidate predictors, outcome events and events per candidate predictor:<br>"The clinicopathological characteristics of 1360 RCC patients are in Table 1. The median age was was 55 years (IQR 46-65) and 408 (30%) were female; 1228 (90.29%) had clear-cell RCC. The median fibrinogen level for all patients was 320 mg/dL (interquartile range [IQR] 274-384.25) and median serum cholesterol level was 168.98 mg/dL (IQR 147.33-192.96). The median follow-up was 67 months (IQR 36-74); 221 patients (16.3%) showed disease progression and 139 (10.2%) died due to RCC during follow-up"<br><br>The total number of predictors is 17 (table 4)<br>The number of events is 139<br><br>EPV= 139/17=8.17		
Describe how the model was developed (for example in regards to modelling technique (e.g. survival or logistic modelling), predictor selection, and risk group definition):<br><br>"Variables with significant difference (P < 0.05) on univariate analysis were included in a Cox proportional-hazards model for multivariate survival analyses, estimating hazard ratios (HRs) and their 95% CIs. For all tests, P < 0.05 was considered statistically significant"		
Describe whether and how the model was validated, either internally (e.g. bootstrapping, cross validation, random split sample) or externally (e.g. temporal validation, geographical validation, different setting, different type of participants):<br><br>Not indicated		
Describe the performance measures of the model, e.g. (re)calibration, discrimination, (re)classification, net benefit, and whether they were adjusted for optimism:<br><br>Not indicated		
Describe any participants who were excluded from the analysis:<br>Not indicated		
Describe missing data on predictors and outcomes as well as methods used for missing data:<br>Not indicated		
	Dev	Val
4.1 Were there a reasonable number of participants with the outcome?	N	N/A
4.2 Were continuous and categorical predictors handled appropriately?	N	N/A
4.3 Were all enrolled participants included in the analysis?	NI	N/A
4.4 Were participants with missing data handled appropriately?	NI	N/A
4.5 Was selection of predictors based on univariable analysis avoided?	N	
4.6 Were complexities in the data (e.g. censoring, competing risks, sampling of controls) accounted for appropriately?	N	N/A
4.7 Were relevant model performance measures evaluated appropriately?	N	N/A
4.8 Were model overfitting and optimism in model performance accounted for?	N	

4.9 Do predictors and their assigned weights in the final model correspond to the results from multivariable analysis?		PY	
**Risk of bias introduced by the analysis**	**RISK:** *(low/ high/ unclear)*	High	N/A

Rationale of bias rating:
They didn´t have enough patients with the outcome. They did categorizations. They didn´t say anything about missing data. They selected the predictors based on univariable. They didn´t do validation, calibration or discrimination. They didn't use competing risks.

Step 4: Overall assessment

Use the following tables to reach overall judgements about risk of bias and concerns regarding applicability of the prediction model evaluation (development and/or validation) across all assessed domains.
Complete for each evaluation of a distinct model.

**Reaching an overall judgement about risk of bias of the prediction model evaluation**	
**Low risk of bias**	If all domains were rated low risk of bias.<br>If a prediction model was developed without any external validation, and it was rated as low risk of bias for all domains, consider downgrading to **high risk of bias**. Such a model can only be considered as low risk of bias, if the development was based on a very large data set and included some form of internal validation.
**High risk of bias**	If at least one domain is judged to be at **high risk of bias**.
**Unclear risk of bias**	If an unclear risk of bias was noted in at least one domain and it was low risk for all other domains.

**Reaching an overall judgement about applicability of the prediction model evaluation**	
**Low concerns regarding applicability**	If low concerns regarding applicability for all domains, the prediction model evaluation is judged to have **low concerns regarding applicability**.
**High concerns regarding applicability**	If high concerns regarding applicability for at least one domain, the prediction model evaluation is judged to have **high concerns regarding applicability**.
**Unclear concerns regarding applicability**	If unclear concerns (but no "high concern") regarding applicability for at least one domain, the prediction model evaluation is judged to have **unclear concerns regarding applicability** overall.

**Overall judgement about risk of bias and applicability of the prediction model evaluation**		
**Overall judgement of risk of bias**	**RISK:**<br>*(low/ high/ unclear)*	High
Summary of sources of potential bias:<br>Analysis domain shows several major issues		
**Overall judgement of applicability**	**CONCERN:**<br>*(low/ high/ unclear)*	Low
Summary of applicability concerns:<br><br>No several major issues		

PROBAST

(Prediction model study Risk Of Bias Assessment Tool)

Published in Annals of Internal Medicine (freely available):

1. PROBAST: A Tool to Assess the Risk of Bias and Applicability of Prediction Model Studies
2. PROBAST: A Tool to Assess Risk of Bias and Applicability of Prediction Model Studies: Explanation and Elaboration

What does PROBAST assess?

PROBAST assesses both the *risk of bias* and *concerns regarding applicability* of a study that evaluates (develops, validates or updates) a multivariable diagnostic or prognostic prediction model. It is designed to assess primary studies included in a systematic review.

Bias occurs if systematic flaws or limitations in the design, conduct or analysis of a primary study distort the results. For the purpose of prediction modelling studies, we have defined *risk of bias* to occur when shortcomings in the study design, conduct or analysis lead to systematically distorted estimates of a model's predictive performance or to an inadequate model to address the research question. Model predictive performance is typically evaluated using calibration, discrimination and sometimes classification measures, and these are likely inaccurately estimated in studies with high risk of bias. *Applicability* refers to the extent to which the prediction model from the primary study matches your systematic review question, for example in terms of the participants, predictors or outcome of interest.

A primary study may include the development and/or validation or update of more than one prediction model. A PROBAST assessment should be completed for each distinct model that is developed, validated or updated (extended) for making individualised predictions. Where a publication assesses multiple prediction models, only complete a PROBAST assessment for those models that meet the inclusion criteria for your systematic review. Please note that subsequent use of the term "model" includes derivatives of models, such as simplified risk scores, nomograms, or recalibrations of models.

PROBAST is not designed for all multivariable diagnostic or prognostic studies. For example, studies using multivariable models to identify predictors associated with an outcome but not attempting to develop a model for making individualised predictions are not covered by PROBAST.

PROBAST includes four steps.

Step	Task	When to complete
1	Specify your systematic review question(s)	Once per systematic review
2	Classify the type of prediction model evaluation	Once for each model of interest in each publication being assessed, for each relevant outcome
3	Assess risk of bias and applicability	Once for each development and validation of each distinct prediction model in a publication
4	Overall judgment	Once for each development and validation of each distinct prediction model in a publication

If this is your first time using PROBAST, we strongly recommend reading the detailed explanation and elaboration (E&E, see link above) paper and to check the examples on www.probast.org

Step 1: Specify your systematic review question

State your systematic review question to facilitate the assessment of the applicability of the evaluated models to your question. *The following table should be completed once per systematic review.*

Criteria	Specify your systematic review question
Intended use of model:	*To predict cancer specific mortality in patients with renal cancer treated with partial or total nephrectomy regardless of TNM stage*
***Participants** including selection criteria and setting:*	*Patients with renal cancer treated with partial or total nephrectomy regardless of TNM stage*
***Predictors** (used in prediction modelling), including types of predictors (e.g. history, clinical examination, biochemical markers, imaging tests), time of measurement, specific measurement issues (e.g., any requirements/ prohibitions for specialized equipment):*	*Predictors used in clinical practice measured when a nephrectomy for renal cancer is indicated*
Outcome to be predicted:	*Cancer specific mortality*

Step 2: Classify the type of prediction model evaluation

Use the following table to classify the evaluation as model development, model validation or model update, or combination. Different signalling questions apply for different types of prediction model evaluation. If the evaluation does not fit one of these classifications then PROBAST should not be used.

**Classify the evaluation based on its aim**			
**Type of prediction study**	**PROBAST boxes to complete**	**Tick as appropriate**	**Definition for type of prediction model study**
Development only	Development	✓	Prediction model development without external validation. These studies may include internal validation methods, such as bootstrapping and cross-validation techniques.
Development and validation	Development and validation	✗	Prediction model development combined with external validation in other participants in the same article.
Validation only	Validation	✗	External validation of existing (previously developed) model in other participants.

This table should be completed once for each publication being assessed and for each relevant outcome in your review.

**Publication reference**	Kutikov A, Egleston B, Canter D, Smaldone MC, Wont YN, Uzzo RG. Competing Risks of Death in Patients with Localized Renal Cell Carcinoma: A Comorbidity Based Model. J Urol. 2012 Dec;188(6):2077-83. doi: 10.1016/j.juro.2012.07.100.
**Models of interest**	Nomogram
**Outcome of interest**	*Cancer specific mortality*

Step 3: Assess risk of bias and applicability

PROBAST is structured as four key domains. Each domain is judged for risk of bias (low, high or unclear) and includes signalling questions to help make judgements. Signalling questions are rated as yes (Y), probably yes (PY), probably no (PN), no (N) or no information (NI). All signalling questions are phrased so that “yes” indicates absence of bias. Any signalling question rated as “no” or “probably no” flags the potential for bias; you will need to use your judgement to determine whether the domain should be rated as “high”, “low” or “unclear” risk of bias. The guidance document contains further instructions and examples on rating signalling questions and risk of bias for each domain.

The first three domains are also rated for concerns regarding applicability (low/ high/ unclear) to your review question defined above.

Complete all domains separately for each evaluation of a distinct model. Shaded boxes indicate where signalling questions do not apply and should not be answered.

DOMAIN 1: Participants			
**A. Risk of Bias**			
Describe the sources of data and criteria for participant selection:<br>*"We included in analysis patients for whom kidney cancer was the first lifetime cancer diagnosis. We also included only patients who had Medicare Part A and B coverage for 1 year before and 1 year after cancer diagnosis. Those who died less than 1 year after diagnosis but had Medicare Part A and B coverage until death were also included. As such, although most beneficiaries enter Medicare at age 65 years, we restricted the study sample to those 66 years old or older at diagnosis to ensure that all patients had at least 1 year of claims data from which to derive the co- morbidity burden. Of these patients 22 were excluded due to missing diagnosis dates, leaving 7,326 with sufficient Medicare coverage for study inclusion. A further 460 pa- tients without common histologies (clear cell, papillary, chromophobe or nonspecified adenocarcinoma), 27 with tumors greater than 20 cm and 174 with missing covariate data were excluded from study. The final sample included 6,665 individuals 66 years old or older with localized, node negative RCC in the linked SEER Medicare data set for 1995 to 2005"*			
		Dev	Val
1.1 Were appropriate data sources used, e.g. cohort, RCT or nested case-control study data?		Y	N/A
1.2 Were all inclusions and exclusions of participants appropriate?		N	N/A
**Risk of bias introduced by selection of participants**	**RISK:**<br>*(low/ high/ unclear)*	High	N/A
Rationale of bias rating:<br>*Cohort study not included patients with previous tumor.*<br>*Cohort study only included patients with a medicare coverage for on years before and after cancer diagnosis*<br>*Cohort study only included patients older than 65 years*			
**B. Applicability**			
Describe included participants, setting and dates:<br>*"We included in analysis patients for whom kidney cancer was the first lifetime cancer diagnosis. We also included only patients who had Medicare Part A and B coverage for 1 year before and 1 year after cancer diagnosis. Those who died less than 1 year after diagnosis but had Medicare Part A and B coverage until death were also included. As such, although most beneficiaries enter Medicare at age 65 years, we restricted the study sample to those 66 years old or older at diagnosis to ensure that all patients had at least 1 year of claims data from which to derive the co- morbidity burden. Of these patients 22 were excluded due to missing diagnosis dates, leaving 7,326 with sufficient Medicare coverage for study inclusion. A further 460 pa- tients without common histologies (clear cell, papillary, chromophobe or nonspecified adenocarcinoma), 27 with tumors greater than 20 cm and 174 with missing covariate data were excluded from study. The final sample included 6,665 individuals 66 years old or older with localized, node negative RCC in the linked SEER Medicare data set for 1995 to 2005"*			
**Concern that the included participants and setting do not match the review question**	**CONCERN:**<br>*(low/ high/ unclear)*	High	N/A
Rationale of applicability rating:<br>*Cohort study not included patients with previous tumor.*<br>*Cohort study only included patients with a medicare coverage for on years before and after cancer diagnosis*<br>*Cohort study only included patients older than 65 years*			

DOMAIN 2: Predictors			
**A. Risk of Bias**			
List and describe predictors included in the final model, e.g. definition and timing of assessment:<br><br>The predictors included in the model were: race, sex, size, age and Charlson score<br><br>The predictors were measured at diagnosis			
		Dev	Val
2.1 Were predictors defined and assessed in a similar way for all participants?		PY	N/A
2.2 Were predictor assessments made without knowledge of outcome data?		PY	N/A
2.3 Are all predictors available at the time the model is intended to be used?		PY	N/A
**Risk of bias introduced by predictors or their assessment**	**RISK:**<br>*(low/ high/ unclear)*	Low	N/A
Rationale of bias rating:<br>*The blinding of measurement is unknown but all the predictors are objective.*			
**B. Applicability**			
Concern that the definition, assessment or timing of predictors in the model do not match the review question	**CONCERN:**<br>*(low/ high/ unclear)*	Low	N/A
Rationale of applicability rating:<br>*No major issues identified.*			

DOMAIN 3: Outcome			
**A. Risk of Bias**			
Describe the outcome, how it was defined and determined, and the time interval between predictor assessment and outcome determination:<br>The outcome was cancer specific survival at 5 years			
		Dev	Val
3.1 Was the outcome determined appropriately?		PY	N/A
3.2 Was a pre-specified or standard outcome definition used?		Y	N/A
3.3 Were predictors excluded from the outcome definition?		PY	N/A
3.4 Was the outcome defined and determined in a similar way for all participants?		PY	N/A
3.5 Was the outcome determined without knowledge of predictor information?		PY	N/A
3.6 Was the time interval between predictor assessment and outcome determination appropriate?		Y	N/A
**Risk of bias introduced by the outcome or its determination**	**RISK:**<br>*(low/ high/ unclear)*	Low	N/A
Rationale of bias rating:<br>*No major issues identified*			
**B. Applicability**			
At what time point was the outcome determined:<br>5 years<br><br>*If a composite outcome was used, describe the relative frequency/distribution of each contributing outcome:*<br>*N/A*			
**Concern that the outcome, its definition, timing or determination do not match the review question**	**CONCERN:**<br>*(low/ high/ unclear)*	Low	N/A
Rationale of applicability rating:<br>*The outcome of the primary study matches the outcome of interest of the review*			

DOMAIN 4: Analysis			
**Risk of Bias**			
Describe numbers of participants, number of candidate predictors, outcome events and events per candidate predictor:<br>*"The final sample included 6,665 individuals 66 years old or older with localized, node negative RCC in the linked SEER Medicare data set for 1995 to 2005"*<br>*The total number of predictors is unknown (Table 1)*<br>*The number of events were 466*<br>*EPV= 466/9 = 51.7*			
Describe how the model was developed (for example in regards to modelling technique (e.g. survival or logistic modelling), predictor selection, and risk group definition):<br><br>*"We used the Fine and Gray competing risks proportional hazards regression to predict 5-year probabilities of competing mortality outcomes from kidney cancer death and other causes of death"*			
Describe whether and how the model was validated, either internally (e.g. bootstrapping, cross validation, random split sample) or externally (e.g. temporal validation, geographical validation, different setting, different type of participants):<br><br>*None*			
Describe the performance measures of the model, e.g. (re)calibration, discrimination, (re)classification, net benefit, and whether they were adjusted for optimism:<br><br>*"We used quintile calibration, as previously described.13,14 Figure 5 shows the results of model calibration"*			
Describe any participants who were excluded from the analysis:<br>*"As such, although most beneficiaries enter Medicare at age 65 years, we restricted the study sample to those 66 years old or older at diagnosis to ensure that all patients had at least 1 year of claims data from which to derive the comorbidity burden. Of these patients 22 were excluded due to missing diagnosis dates, leaving 7,326 with sufficient Medicare coverage for study inclusion. A further 460 patients without common histologies (clear cell, papillary, chromophobe or nonspecified adenocarcinoma), 27 with tumors greater than 20 cm and 174 with missing covariate data were excluded from study"*			
Describe missing data on predictors and outcomes as well as methods used for missing data:<br>*"Of these patients 22 were excluded due to missing diagnosis dates"*			
		Dev	Val
4.1 Were there a reasonable number of participants with the outcome?		Y	N/A
4.2 Were continuous and categorical predictors handled appropriately?		Y	N/A
4.3 Were all enrolled participants included in the analysis?		N	N/A
4.4 Were participants with missing data handled appropriately?		N	N/A
4.5 Was selection of predictors based on univariable analysis avoided?		Y	
4.6 Were complexities in the data (e.g. censoring, competing risks, sampling of controls) accounted for appropriately?		Y	N/A
4.7 Were relevant model performance measures evaluated appropriately?		N	N/A
4.8 Were model overfitting and optimism in model performance accounted for?		N	
4.9 Do predictors and their assigned weights in the final model correspond to the results from multivariable analysis?		PY	
**Risk of bias introduced by the analysis**	**RISK:**	High	N/A

	(low/ high/ unclear)		

Rationale of bias rating:<br>They excluded patients with missing data. They didn´t make discrimination. They didn´t do overfitting and optimism in model.

Step 4: Overall assessment

Use the following tables to reach overall judgements about risk of bias and concerns regarding applicability of the prediction model evaluation (development and/or validation) across all assessed domains.
Complete for each evaluation of a distinct model.

Reaching an overall judgement about risk of bias of the prediction model evaluation	
**Low risk of bias**	If all domains were rated low risk of bias.<br>If a prediction model was developed without any external validation, and it was rated as low risk of bias for all domains, consider downgrading to **high risk of bias**. Such a model can only be considered as low risk of bias, if the development was based on a very large data set and included some form of internal validation.
**High risk of bias**	If at least one domain is judged to be at **high risk of bias**.
**Unclear risk of bias**	If an unclear risk of bias was noted in at least one domain and it was low risk for all other domains.

Reaching an overall judgement about applicability of the prediction model evaluation	
**Low concerns regarding applicability**	If low concerns regarding applicability for all domains, the prediction model evaluation is judged to have **low concerns regarding applicability**.
**High concerns regarding applicability**	If high concerns regarding applicability for at least one domain, the prediction model evaluation is judged to have **high concerns regarding applicability**.
**Unclear concerns regarding applicability**	If unclear concerns (but no "high concern") regarding applicability for at least one domain, the prediction model evaluation is judged to have **unclear concerns regarding applicability** overall.

Overall judgement about risk of bias and applicability of the prediction model evaluation		
**Overall judgement of risk of bias**	**RISK:**<br>*(low/ high/ unclear)*	High
Summary of sources of potential bias:<br>Analysis and participants domains show several major issues		
**Overall judgement of applicability**	**CONCERN:**<br>*(low/ high/ unclear)*	High
Summary of applicability concerns:<br>Participants domain shows several major issues		

PROBAST

(Prediction model study Risk Of Bias Assessment Tool)

Published in Annals of Internal Medicine (freely available):

1. PROBAST: A Tool to Assess the Risk of Bias and Applicability of Prediction Model Studies
2. PROBAST: A Tool to Assess Risk of Bias and Applicability of Prediction Model Studies: Explanation and Elaboration

What does PROBAST assess?

PROBAST assesses both the *risk of bias* and *concerns regarding applicability* of a study that evaluates (develops, validates or updates) a multivariable diagnostic or prognostic prediction model. It is designed to assess primary studies included in a systematic review.

Bias occurs if systematic flaws or limitations in the design, conduct or analysis of a primary study distort the results. For the purpose of prediction modelling studies, we have defined *risk of bias* to occur when shortcomings in the study design, conduct or analysis lead to systematically distorted estimates of a model's predictive performance or to an inadequate model to address the research question. Model predictive performance is typically evaluated using calibration, discrimination and sometimes classification measures, and these are likely inaccurately estimated in studies with high risk of bias. *Applicability* refers to the extent to which the prediction model from the primary study matches your systematic review question, for example in terms of the participants, predictors or outcome of interest.

A primary study may include the development and/or validation or update of more than one prediction model. A PROBAST assessment should be completed for each distinct model that is developed, validated or updated (extended) for making individualised predictions. Where a publication assesses multiple prediction models, only complete a PROBAST assessment for those models that meet the inclusion criteria for your systematic review. Please note that subsequent use of the term "model" includes derivatives of models, such as simplified risk scores, nomograms, or recalibrations of models.

PROBAST is not designed for all multivariable diagnostic or prognostic studies. For example, studies using multivariable models to identify predictors associated with an outcome but not attempting to develop a model for making individualised predictions are not covered by PROBAST.

PROBAST includes four steps.

Step	Task	When to complete
**1**	Specify your systematic review question(s)	Once per systematic review
**2**	Classify the type of prediction model evaluation	Once for each model of interest in each publication being assessed, for each relevant outcome
**3**	Assess risk of bias and applicability	Once for each development and validation of each distinct prediction model in a publication
**4**	Overall judgment	Once for each development and validation of each distinct prediction model in a publication

If this is your first time using PROBAST, we strongly recommend reading the detailed explanation and elaboration (E&E, see link above) paper and to check the examples on www.probast.org

Step 1: Specify your systematic review question

State your systematic review question to facilitate the assessment of the applicability of the evaluated models to your question. *The following table should be completed once per systematic review.*

Criteria	Specify your systematic review question
Intended use of model:	*To predict cancer specific mortality in patients with renal cancer treated with partial or total nephrectomy regardless of TNM stage*
***Participants** including selection criteria and setting:*	*Patients with renal cancer treated with partial or total nephrectomy regardless of TNM stage*
***Predictors** (used in prediction modelling), including types of predictors (e.g. history, clinical examination, biochemical markers, imaging tests), time of measurement, specific measurement issues (e.g., any requirements/ prohibitions for specialized equipment):*	*Predictors used in clinical practice measured when a nephrectomy for renal cancer is indicated*
Outcome to be predicted:	*Cancer specific mortality*

Step 2: Classify the type of prediction model evaluation

Use the following table to classify the evaluation as model development, model validation or model update, or combination. Different signalling questions apply for different types of prediction model evaluation. If the evaluation does not fit one of these classifications then PROBAST should not be used.

**Classify the evaluation based on its aim**			
**Type of prediction study**	**PROBAST boxes to complete**	**Tick as appropriate**	**Definition for type of prediction model study**
Development only	Development	✓	Prediction model development without external validation. These studies may include internal validation methods, such as bootstrapping and cross-validation techniques.
Development and validation	Development and validation	✗	Prediction model development combined with external validation in other participants in the same article.
Validation only	Validation	✗	External validation of existing (previously developed) model in other participants.

This table should be completed once for each publication being assessed and for each relevant outcome in your review.

**Publication reference**	Frank I, Blute ML, Cheville JC, Lohse CM, Weaver AL, Zincke H. An outcome prediction model for patients with clear cell renal cell carcinoma treated with radical nephrectomy based on tumor stage, size, grade and necrosis: the SSIGN score. J Urol. 2002;168(6):2395-400.
**Models of interest**	Risk score
**Outcome of interest**	*Cancer specific mortality*

Step 3: Assess risk of bias and applicability

PROBAST is structured as four key domains. Each domain is judged for risk of bias (low, high or unclear) and includes signalling questions to help make judgements. Signalling questions are rated as yes (Y), probably yes (PY), probably no (PN), no (N) or no information (NI). All signalling questions are phrased so that “yes” indicates absence of bias. Any signalling question rated as “no” or “probably no” flags the potential for bias; you will need to use your judgement to determine whether the domain should be rated as “high”, “low” or “unclear” risk of bias. The guidance document contains further instructions and examples on rating signalling questions and risk of bias for each domain.

The first three domains are also rated for concerns regarding applicability (low/ high/ unclear) to your review question defined above.

Complete all domains separately for each evaluation of a distinct model. Shaded boxes indicate where signalling questions do not apply and should not be answered.

<table>
<tr><td colspan="4">DOMAIN 1: Participants</td></tr>
<tr><td colspan="4">A. Risk of Bias</td></tr>
<tr><td colspan="4">Describe the sources of data and criteria for participant selection:
“A total of 1,896 patients underwent radical nephrectomy for clear cell renal cell carcinoma between 1970 and 1998”

“Patients with bilateral synchronous tumors (29), familial von Hippel-Lindau or tuberous sclerosis syndromes (24), or Wilms tumor and those who were younger than 18 years at surgery (6), denied access to medical records for research (7) or were missing data for tumor stage and size (29) were excluded from study”</td></tr>
<tr><td colspan="2"></td><td>Dev</td><td>Val</td></tr>
<tr><td colspan="2">1.1 Were appropriate data sources used, e.g. cohort, RCT or nested case-control study data?</td><td>Y</td><td>N/A</td></tr>
<tr><td colspan="2">1.2 Were all inclusions and exclusions of participants appropriate?</td><td>Y</td><td>N/A</td></tr>
<tr><td>Risk of bias introduced by selection of participants</td><td>RISK:
(low/ high/ unclear)</td><td>Low</td><td>N/A</td></tr>
<tr><td colspan="4">Rationale of bias rating:
The author described the initially selected patients and those excluded from the study</td></tr>
<tr><td colspan="4">B. Applicability</td></tr>
<tr><td colspan="4">Describe included participants, setting and dates:

“A total of 1,896 patients underwent radical nephrectomy for clear cell renal cell carcinoma between 1970 and 1998”

“Patients with bilateral synchronous tumors (29), familial von Hippel-Lindau or tuberous sclerosis syndromes (24), or Wilms tumor and those who were younger than 18 years at surgery (6), denied access to medical records for research (7) or were missing data for tumor stage and size (29) were excluded from study”</td></tr>
<tr><td>Concern that the included participants and setting do not match the review question</td><td>CONCERN:
(low/ high/ unclear)</td><td>Low</td><td>N/A</td></tr>
<tr><td colspan="4">Rationale of applicability rating:
The author described the initially selected patients and those excluded from the study</td></tr>
</table>

**DOMAIN 2: Predictors**			
**A. Risk of Bias**			
List and describe predictors included in the final model, e.g. definition and timing of assessment:<br><br>The predictors included in the model were: TNM, tumor size, nuclear grade and necrosis<br><br>All the predictors were measured after treatment			
		Dev	Val
2.1 Were predictors defined and assessed in a similar way for all participants?		PY	N/A
2.2 Were predictor assessments made without knowledge of outcome data?		PY	N/A
2.3 Are all predictors available at the time the model is intended to be used?		PY	N/A
**Risk of bias introduced by predictors or their assessment**	**RISK:**<br>*(low/ high/ unclear)*	Low	N/A
Rationale of bias rating:<br>*Microscopic slides from all tumour specimens were reviewed by a urological pathologist without knowledge of patient outcome. All the assessments were carried out in a similar way for all participants.*			
**B. Applicability**			
Concern that the definition, assessment or timing of predictors in the model do not match the review question	**CONCERN:**<br>*(low/ high/ unclear)*	Low	N/A
Rationale of applicability rating:<br>*No major issues identified.*			

**DOMAIN 3: Outcome**			
**A. Risk of Bias**			
Describe the outcome, how it was defined and determined, and the time interval between predictor assessment and outcome determination:<br>The outcome was cancer specific survival at 1, 3, 5, 7 and 10-years			
		Dev	Val
3.1 Was the outcome determined appropriately?		PY	N/A
3.2 Was a pre-specified or standard outcome definition used?		Y	N/A
3.3 Were predictors excluded from the outcome definition?		PY	N/A
3.4 Was the outcome defined and determined in a similar way for all participants?		PY	N/A
3.5 Was the outcome determined without knowledge of predictor information?		PY	N/A
3.6 Was the time interval between predictor assessment and outcome determination appropriate?		Y	N/A
**Risk of bias introduced by the outcome or its determination**	**RISK:**<br>*(low/ high/ unclear)*	Low	N/A
Rationale of bias rating:<br>*No major issues identified*			
**B. Applicability**			
At what time point was the outcome determined:<br>1, 3, 5, 7 and 10-years<br><br>*If a composite outcome was used, describe the relative frequency/distribution of each contributing outcome:*<br>*N/A*			
**Concern that the outcome, its definition, timing or determination do not match the review question**	**CONCERN:**<br>*(low/ high/ unclear)*	Low	N/A
Rationale of applicability rating:<br>*The outcome of the primary study matches the outcome of interest of the review*			

DOMAIN 4: Analysis

Risk of Bias

Describe numbers of participants, number of candidate predictors, outcome events and events per candidate predictor:

“Thus, 1,801 patients with sporadic unilateral clear cell renal cell carcinoma were avail- able for analysis”
The total number of predictors is 19
The number of events is 652

EPV=652/19=34.31

Describe how the model was developed (for example in regards to modelling technique (e.g. survival or logistic modelling), predictor selection, and risk group definition):

“Univariate and multivariate Cox proportional hazards models were fit to determine clinical, surgical and pathological features associated with death from clear cell renal cell carcinoma. Relationships between outcome and features were summarized with risk ratios and 95% CI. A candidate multivariate model was built using a stepwise selection procedure with p ⍰ 0.05 as a feature to enter or leave the model”

Describe whether and how the model was validated, either internally (e.g. bootstrapping, cross validation, random split sample) or externally (e.g. temporal validation, geographical validation, different setting, different type of participants):

“Features included in the final model were validated using bootstrap methodology”

Describe the performance measures of the model, e.g. (re)calibration, discrimination, (re)classification, net benefit, and whether they were adjusted for optimism:

“The predictive ability of the final model and comparisons between competing models were evaluated using a c (concor- dance) index proposed by Harrell et all”

Describe any participants who were excluded from the analysis:
“Patients with bilateral synchronous tumors (29), familial von Hippel-Lindau or tuberous sclerosis syn- dromes (24), or Wilms tumor and those who were younger than 18 years at surgery (6), denied access to medical records for research (7) or were missing data for tumor stage and size (29) were excluded from study”

Describe missing data on predictors and outcomes as well as methods used for missing data:
“Patients with bilateral synchronous tumors (29), familial von Hippel-Lindau or tuberous sclerosis syn- dromes (24), or Wilms tumor and those who were younger than 18 years at surgery (6), denied access to medical records for research (7) or were missing data for tumor stage and size (29) were excluded from study”

	Dev	Val
4.1 Were there a reasonable number of participants with the outcome?	Y	N/A
4.2 Were continuous and categorical predictors handled appropriately?	N	N/A
4.3 Were all enrolled participants included in the analysis?	N	N/A

4.4 Were participants with missing data handled appropriately?		N	N/A
4.5 Was selection of predictors based on univariable analysis avoided?		N	
4.6 Were complexities in the data (e.g. censoring, competing risks, sampling of controls) accounted for appropriately?		N	N/A
4.7 Were relevant model performance measures evaluated appropriately?		N	N/A
4.8 Were model overfitting and optimism in model performance accounted for?		Y	
4.9 Do predictors and their assigned weights in the final model correspond to the results from multivariable analysis?		PY	
**Risk of bias introduced by the analysis**	**RISK:** *(low/ high/ unclear)*	High	N/A
Rationale of bias rating: *They did categorizations. They excluded patients with missing data. They selected the predictors based on univariable. They didn't use competing risks. They didn´t do calibration*			

Step 4: Overall assessment

Use the following tables to reach overall judgements about risk of bias and concerns regarding applicability of the prediction model evaluation (development and/or validation) across all assessed domains.
Complete for each evaluation of a distinct model.

Reaching an overall judgement about risk of bias of the prediction model evaluation	
**Low risk of bias**	If all domains were rated low risk of bias.<br>If a prediction model was developed without any external validation, and it was rated as low risk of bias for all domains, consider downgrading to **high risk of bias**. Such a model can only be considered as low risk of bias, if the development was based on a very large data set and included some form of internal validation.
**High risk of bias**	If at least one domain is judged to be at **high risk of bias**.
**Unclear risk of bias**	If an unclear risk of bias was noted in at least one domain and it was low risk for all other domains.

Reaching an overall judgement about applicability of the prediction model evaluation	
**Low concerns regarding applicability**	If low concerns regarding applicability for all domains, the prediction model evaluation is judged to have **low concerns regarding applicability**.
**High concerns regarding applicability**	If high concerns regarding applicability for at least one domain, the prediction model evaluation is judged to have **high concerns regarding applicability**.
**Unclear concerns regarding applicability**	If unclear concerns (but no "high concern") regarding applicability for at least one domain, the prediction model evaluation is judged to have **unclear concerns regarding applicability** overall.

Overall judgement about risk of bias and applicability of the prediction model evaluation		
**Overall judgement of risk of bias**	**RISK:**<br>*(low/ high/ unclear)*	High
Summary of sources of potential bias:<br>Analysis domain shows several major issues		
**Overall judgement of applicability**	**CONCERN:**<br>*(low/ high/ unclear)*	Low
Summary of applicability concerns:<br><br>No major issues		

PROBAST

(Prediction model study Risk Of Bias Assessment Tool)

Published in Annals of Internal Medicine (freely available):

1. PROBAST: A Tool to Assess the Risk of Bias and Applicability of Prediction Model Studies
2. PROBAST: A Tool to Assess Risk of Bias and Applicability of Prediction Model Studies: Explanation and Elaboration

What does PROBAST assess?

PROBAST assesses both the *risk of bias* and *concerns regarding applicability* of a study that evaluates (develops, validates or updates) a multivariable diagnostic or prognostic prediction model. It is designed to assess primary studies included in a systematic review.

Bias occurs if systematic flaws or limitations in the design, conduct or analysis of a primary study distort the results. For the purpose of prediction modelling studies, we have defined *risk of bias* to occur when shortcomings in the study design, conduct or analysis lead to systematically distorted estimates of a model's predictive performance or to an inadequate model to address the research question. Model predictive performance is typically evaluated using calibration, discrimination and sometimes classification measures, and these are likely inaccurately estimated in studies with high risk of bias. *Applicability* refers to the extent to which the prediction model from the primary study matches your systematic review question, for example in terms of the participants, predictors or outcome of interest.

A primary study may include the development and/or validation or update of more than one prediction model. A PROBAST assessment should be completed for each distinct model that is developed, validated or updated (extended) for making individualised predictions. Where a publication assesses multiple prediction models, only complete a PROBAST assessment for those models that meet the inclusion criteria for your systematic review. Please note that subsequent use of the term "model" includes derivatives of models, such as simplified risk scores, nomograms, or recalibrations of models.

PROBAST is not designed for all multivariable diagnostic or prognostic studies. For example, studies using multivariable models to identify predictors associated with an outcome but not attempting to develop a model for making individualised predictions are not covered by PROBAST.

PROBAST includes four steps.

Step	Task	When to complete
**1**	Specify your systematic review question(s)	Once per systematic review
**2**	Classify the type of prediction model evaluation	Once for each model of interest in each publication being assessed, for each relevant outcome
**3**	Assess risk of bias and applicability	Once for each development and validation of each distinct prediction model in a publication
**4**	Overall judgment	Once for each development and validation of each distinct prediction model in a publication

If this is your first time using PROBAST, we strongly recommend reading the detailed explanation and elaboration (E&E, see link above) paper and to check the examples on www.probast.org

Step 1: Specify your systematic review question

State your systematic review question to facilitate the assessment of the applicability of the evaluated models to your question. *The following table should be completed once per systematic review.*

Criteria	Specify your systematic review question
Intended use of model:	*To predict cancer specific mortality in patients with renal cancer treated with partial or total nephrectomy regardless of TNM stage*
***Participants** including selection criteria and setting:*	*Patients with renal cancer treated with partial or total nephrectomy regardless of TNM stage*
***Predictors** (used in prediction modelling), including types of predictors (e.g. history, clinical examination, biochemical markers, imaging tests), time of measurement, specific measurement issues (e.g., any requirements/ prohibitions for specialized equipment):*	*Predictors used in clinical practice measured when a nephrectomy for renal cancer is indicated*
Outcome to be predicted:	*Cancer specific mortality*

Step 2: Classify the type of prediction model evaluation

Use the following table to classify the evaluation as model development, model validation or model update, or combination. Different signalling questions apply for different types of prediction model evaluation. If the evaluation does not fit one of these classifications then PROBAST should not be used.

**Classify the evaluation based on its aim**			
**Type of prediction study**	**PROBAST boxes to complete**	**Tick as appropriate**	**Definition for type of prediction model study**
Development only	Development	✓	Prediction model development without external validation. These studies may include internal validation methods, such as bootstrapping and cross-validation techniques.
Development and validation	Development and validation	✗	Prediction model development combined with external validation in other participants in the same article.
Validation only	Validation	✗	External validation of existing (previously developed) model in other participants.

This table should be completed once for each publication being assessed and for each relevant outcome in your review.

**Publication reference**	Margulis V, Shariat SF, Rapoport Y, Rink M, Sjoberg DD, Tannir NM, Abel EJ, Culp SH, Tamboli P, Wood CG. Development of accurate models for individualized prediction of survival after cytoreductive nephrectomy for metastatic renal cell carcinoma. Eur Urol. 2013; 63(5):947-52
**Models of interest**	Nomogram
**Outcome of interest**	*Cancer specific mortality*

Step 3: Assess risk of bias and applicability

PROBAST is structured as four key domains. Each domain is judged for risk of bias (low, high or unclear) and includes signalling questions to help make judgements. Signalling questions are rated as yes (Y), probably yes (PY), probably no (PN), no (N) or no information (NI). All signalling questions are phrased so that "yes" indicates absence of bias. Any signalling question rated as "no" or "probably no" flags the potential for bias; you will need to use your judgement to determine whether the domain should be rated as "high", "low" or "unclear" risk of bias. The guidance document contains further instructions and examples on rating signalling questions and risk of bias for each domain.

The first three domains are also rated for concerns regarding applicability (low/ high/ unclear) to your review question defined above.

Complete all domains separately for each evaluation of a distinct model. Shaded boxes indicate where signalling questions do not apply and should not be answered.

DOMAIN 1: Participants			
**A. Risk of Bias**			
Describe the sources of data and criteria for participant selection:<br><br>“With approval from the Institutional Review Board for the Protection of Human Subjects at the MD Anderson Cancer Center, the institutional cancer database was queried for patients with mRCC who underwent CN between 1991 and 2008, yielding a cohort of 601 patients”<br>“There were 27 patients excluded from postoperative model development because of lack of sufficient follow- up”			
		Dev	Val
1.1 Were appropriate data sources used, e.g. cohort, RCT or nested case-control study data?		Y	N/A
1.2 Were all inclusions and exclusions of participants appropriate?		N	N/A
**Risk of bias introduced by selection of participants**	**RISK:**<br>*(low/ high/ unclear)*	Unclea r	N/A
Rationale of bias rating:<br>They do not specify what they consider as a minimum follow-up time			
**B. Applicability**			
Describe included participants, setting and dates:<br>“With approval from the Institutional Review Board for the Protection of Human Subjects at the MD Anderson Cancer Center, the institutional cancer database was queried for patients with mRCC who underwent CN between 1991 and 2008, yielding a cohort of 601 patients”<br>“There were 27 patients excluded from postoperative model development because of lack of sufficient follow- up”			
**Concern that the included participants and setting do not match the review question**	**CONCERN:**<br>*(low/ high/ unclear)*	Low	N/A
Rationale of applicability rating:<br>They do not specify what they consider as a minimum follow-up time. The study is only applied to patients with metastasis, but the review question is focused on these patients			

DOMAIN 2: Predictors			
**A. Risk of Bias**			
List and describe predictors included in the final model, e.g. definition and timing of assessment:<br><br>The predictors included in the model were:<br>- Preoperative risk score: albumin and LDH<br>- Postoperative risk score: albumin, LDH, N stage, received blood transfusion, T stage ≥ 3			
		Dev	Val
2.1 Were predictors defined and assessed in a similar way for all participants?		PY	N/A
2.2 Were predictor assessments made without knowledge of outcome data?		PY	N/A
2.3 Are all predictors available at the time the model is intended to be used?		PY	N/A
**Risk of bias introduced by predictors or their assessment**	**RISK:**<br>*(low/ high/ unclear)*	Low	N/A
Rationale of bias rating:<br>*The blinding of measurement is unknown but all the predictors are objective*			
**B. Applicability**			
Concern that the definition, assessment or timing of predictors in the model do not match the review question	**CONCERN:**<br>*(low/ high/ unclear)*	Low	N/A
Rationale of applicability rating:<br>*No major issues identified*			

DOMAIN 3: Outcome			
**A. Risk of Bias**			
Describe the outcome, how it was defined and determined, and the time interval between predictor assessment and outcome determination:<br>The outcome was cancer specific survival at 2 and 6 months			
		Dev	Val
3.1 Was the outcome determined appropriately?		PY	N/A
3.2 Was a pre-specified or standard outcome definition used?		Y	N/A
3.3 Were predictors excluded from the outcome definition?		PY	N/A
3.4 Was the outcome defined and determined in a similar way for all participants?		PY	N/A
3.5 Was the outcome determined without knowledge of predictor information?		PY	N/A
3.6 Was the time interval between predictor assessment and outcome determination appropriate?		Y	N/A
**Risk of bias introduced by the outcome or its determination**	**RISK:**<br>*(low/ high/ unclear)*	Low	N/A
Rationale of bias rating:<br>*No major issues identified*			
**B. Applicability**			
At what time point was the outcome determined:<br>3 and 6 months<br><br>*If a composite outcome was used, describe the relative frequency/distribution of each contributing outcome:*<br>*N/A*			
**Concern that the outcome, its definition, timing or determination do not match the review question**	**CONCERN:**<br>*(low/ high/ unclear)*	Low	N/A
Rationale of applicability rating:<br>*The outcome of the primary study matches the outcome of interest of the review*			

DOMAIN 4: Analysis
**Risk of Bias**
Describe numbers of participants, number of candidate predictors, outcome events and events per candidate predictor:<br>"With approval from the Institutional Review Board for the Protection of Human Subjects at the MD Anderson Cancer Center, the institutional cancer database was queried for patients with mRCC who underwent CN between 1991 and 2008, yielding a cohort of 601 patients"<br>"There were 27 patients excluded from postoperative model development because of lack of sufficient follow- up"<br><br>The total number of predictors is:<br>- Preoperative model: 21<br>- Postoperative model: 30<br>EPV<br>- Preoperative model: 215/21=10.24<br>- Postoperative model: 215/30=7.16
Describe how the model was developed (for example in regards to modelling technique (e.g. survival or logistic modelling), predictor selection, and risk group definition):<br><br>"We began by examining all univariate models. The variable that exhibited the best discrimination was retained. Next, all two-variable models that included the first variable selected were examined. The variable with the best marginal improvement in discrimination was retained. This process was continued until no remaining variables increased the area under the curve by >1%. Variables considered in the preoperative model were number of metastatic organ sites; Eastern Cooperative Oncology Group performance status; time from diagnosis to surgery; preoperative glomerular filtration rate (calculated using the Modification of Diet in Renal Disease formula); serum levels of alkaline phosphatase, lactate dehydrogenase (LDH), corrected calcium, albumin, total and fractionated white blood cells, hemoglobin, platelets, and hematocrit; and Motzer criteria [12]. The postoperative model included the preoperative variables, as well as pathologic TN stage, lymph node density, lymphovascular invasion, tumor grade, operating room time, concomitant retroperitoneal lymphadenectomy, and receipt of a blood transfusion during surgery"
Describe whether and how the model was validated, either internally (e.g. bootstrapping, cross validation, random split sample) or externally (e.g. temporal validation, geographical validation, different setting, different type of participants):<br><br>"The discrimination, calibration, and decision curves were corrected for overfit using 10-fold crossvalidation that included the stepwise variable selection"
Describe the performance measures of the model, e.g. (re)calibration, discrimination, (re)classification, net benefit, and whether they were adjusted for optimism:<br><br>"The discrimination, calibration, and decision curves were corrected for overfit using 10-fold crossvalidation that included the stepwise variable selection"
Describe any participants who were excluded from the analysis:<br>"There were 27 patients excluded from postoperative model development because of lack of sufficient follow- up"
Describe missing data on predictors and outcomes as well as methods used for missing data:

Not indicated		
	Dev	Val
4.1 Were there a reasonable number of participants with the outcome?	N	N/A
4.2 Were continuous and categorical predictors handled appropriately?	Y	N/A
4.3 Were all enrolled participants included in the analysis?	NI	N/A
4.4 Were participants with missing data handled appropriately?	NI	N/A
4.5 Was selection of predictors based on univariable analysis avoided?	Y	
4.6 Were complexities in the data (e.g. censoring, competing risks, sampling of controls) accounted for appropriately?	N	N/A
4.7 Were relevant model performance measures evaluated appropriately?	Y	N/A
4.8 Were model overfitting and optimism in model performance accounted for?	Y	
4.9 Do predictors and their assigned weights in the final model correspond to the results from multivariable analysis?	PY	
**Risk of bias introduced by the analysis** — **RISK:** *(low/ high/ unclear)*	High	N/A
Rationale of bias rating: *They didn´t have enough patients with the outcome. They excluded patients without a minimum follow up. They didn´t say anything about with missing data. They didn´t do competing risk.*		

Step 4: Overall assessment

Use the following tables to reach overall judgements about risk of bias and concerns regarding applicability of the prediction model evaluation (development and/or validation) across all assessed domains.
Complete for each evaluation of a distinct model.

**Reaching an overall judgement about risk of bias of the prediction model evaluation**	
**Low risk of bias**	If all domains were rated low risk of bias.<br>If a prediction model was developed without any external validation, and it was rated as low risk of bias for all domains, consider downgrading to **high risk of bias**. Such a model can only be considered as low risk of bias, if the development was based on a very large data set and included some form of internal validation.
**High risk of bias**	If at least one domain is judged to be at **high risk of bias**.
**Unclear risk of bias**	If an unclear risk of bias was noted in at least one domain and it was low risk for all other domains.

**Reaching an overall judgement about applicability of the prediction model evaluation**	
**Low concerns regarding applicability**	If low concerns regarding applicability for all domains, the prediction model evaluation is judged to have **low concerns regarding applicability**.
**High concerns regarding applicability**	If high concerns regarding applicability for at least one domain, the prediction model evaluation is judged to have **high concerns regarding applicability**.
**Unclear concerns regarding applicability**	If unclear concerns (but no "high concern") regarding applicability for at least one domain, the prediction model evaluation is judged to have **unclear concerns regarding applicability** overall.

**Overall judgement about risk of bias and applicability of the prediction model evaluation**		
**Overall judgement of risk of bias**	**RISK:**<br>*(low/ high/ unclear)*	High
Summary of sources of potential bias:<br>Analysis and participants domains show several major issues		
**Overall judgement of applicability**	**CONCERN:**<br>*(low/ high/ unclear)*	Low
Summary of applicability concerns:<br><br>No major concerns.		

PROBAST

(Prediction model study Risk Of Bias Assessment Tool)

Published in Annals of Internal Medicine (freely available):

1. PROBAST: A Tool to Assess the Risk of Bias and Applicability of Prediction Model Studies
2. PROBAST: A Tool to Assess Risk of Bias and Applicability of Prediction Model Studies: Explanation and Elaboration

What does PROBAST assess?

PROBAST assesses both the *risk of bias* and *concerns regarding applicability* of a study that evaluates (develops, validates or updates) a multivariable diagnostic or prognostic prediction model. It is designed to assess primary studies included in a systematic review.

Bias occurs if systematic flaws or limitations in the design, conduct or analysis of a primary study distort the results. For the purpose of prediction modelling studies, we have defined *risk of bias* to occur when shortcomings in the study design, conduct or analysis lead to systematically distorted estimates of a model's predictive performance or to an inadequate model to address the research question. Model predictive performance is typically evaluated using calibration, discrimination and sometimes classification measures, and these are likely inaccurately estimated in studies with high risk of bias. *Applicability* refers to the extent to which the prediction model from the primary study matches your systematic review question, for example in terms of the participants, predictors or outcome of interest.

A primary study may include the development and/or validation or update of more than one prediction model. A PROBAST assessment should be completed for each distinct model that is developed, validated or updated (extended) for making individualised predictions. Where a publication assesses multiple prediction models, only complete a PROBAST assessment for those models that meet the inclusion criteria for your systematic review. Please note that subsequent use of the term "model" includes derivatives of models, such as simplified risk scores, nomograms, or recalibrations of models.

PROBAST is not designed for all multivariable diagnostic or prognostic studies. For example, studies using multivariable models to identify predictors associated with an outcome but not attempting to develop a model for making individualised predictions are not covered by PROBAST.

PROBAST includes four steps.

Step	Task	When to complete
**1**	Specify your systematic review question(s)	Once per systematic review
**2**	Classify the type of prediction model evaluation	Once for each model of interest in each publication being assessed, for each relevant outcome
**3**	Assess risk of bias and applicability	Once for each development and validation of each distinct prediction model in a publication
**4**	Overall judgment	Once for each development and validation of each distinct prediction model in a publication

If this is your first time using PROBAST, we strongly recommend reading the detailed explanation and elaboration (E&E, see link above) paper and to check the examples on www.probast.org

Step 1: Specify your systematic review question

State your systematic review question to facilitate the assessment of the applicability of the evaluated models to your question. *The following table should be completed once per systematic review.*

Criteria	Specify your systematic review question
Intended use of model:	*To predict cancer specific mortality in patients with renal cancer treated with partial or total nephrectomy regardless of TNM stage*
***Participants** including selection criteria and setting:*	*Patients with renal cancer treated with partial or total nephrectomy regardless of TNM stage*
***Predictors** (used in prediction modelling), including types of predictors (e.g. history, clinical examination, biochemical markers, imaging tests), time of measurement, specific measurement issues (e.g., any requirements/ prohibitions for specialized equipment):*	*Predictors used in clinical practice measured when a nephrectomy for renal cancer is indicated*
Outcome to be predicted:	*Cancer specific mortality*

Step 2: Classify the type of prediction model evaluation

Use the following table to classify the evaluation as model development, model validation or model update, or combination. Different signalling questions apply for different types of prediction model evaluation. If the evaluation does not fit one of these classifications then PROBAST should not be used.

**Classify the evaluation based on its aim**			
**Type of prediction study**	**PROBAST boxes to complete**	**Tick as appropriate**	**Definition for type of prediction model study**
Development only	Development	✓	Prediction model development without external validation. These studies may include internal validation methods, such as bootstrapping and cross-validation techniques.
Development and validation	Development and validation	✗	Prediction model development combined with external validation in other participants in the same article.
Validation only	Validation	✗	External validation of existing (previously developed) model in other participants.

This table should be completed once for each publication being assessed and for each relevant outcome in your review.

**Publication reference**	Wu K, Liu Z, Shao Y, Li X. Nomogram Predicting Survival to Assist Decision-Making of Metastasectomy in Patients With Metastatic Renal Cell Carcinoma. Front Oncol. 2020: 7;10:592243
**Models of interest**	Risk score and nomogram
**Outcome of interest**	*Cancer specific mortality*

Step 3: Assess risk of bias and applicability

PROBAST is structured as four key domains. Each domain is judged for risk of bias (low, high or unclear) and includes signalling questions to help make judgements. Signalling questions are rated as yes (Y), probably yes (PY), probably no (PN), no (N) or no information (NI). All signalling questions are phrased so that “yes” indicates absence of bias. Any signalling question rated as “no” or “probably no” flags the potential for bias; you will need to use your judgement to determine whether the domain should be rated as “high”, “low” or “unclear” risk of bias. The guidance document contains further instructions and examples on rating signalling questions and risk of bias for each domain.

The first three domains are also rated for concerns regarding applicability (low/ high/ unclear) to your review question defined above.

Complete all domains separately for each evaluation of a distinct model. Shaded boxes indicate where signalling questions do not apply and should not be answered.

DOMAIN 1: Participants			
**A. Risk of Bias**			
Describe the sources of data and criteria for participant selection:<br><br>"Inclusion criteria included: (1) patients with renal cancer labeled as the ICD-O-3 site codes (C649, 8000-8980); (2) patients who underwent cytoreductive nephrectomy; (3) patients with pathological confirmation; (4) patients aged 18–90 years at initial diagnosis. Patients with missing data on race, distant sites (lung, liver, bone, or brain), T stage, N stage, tumor grade, or survival status were excluded from the cohort. Likewise, cases with bilateral tumors, and those with unknown surgical information were also excluded"<br><br>"The nomogram for predicting CSM was built in patients who did not receive MSX treatment"			
		Dev	Val
1.1 Were appropriate data sources used, e.g. cohort, RCT or nested case-control study data?		Y	N/A
1.2 Were all inclusions and exclusions of participants appropriate?		N	N/A
**Risk of bias introduced by selection of participants**	**RISK:**<br>*(low/ high/ unclear)*	High	N/A
Rationale of bias rating:<br>They excluded patients without metastasis.<br>They excluded metastasic patients who received treatment for the metastasis.			
**B. Applicability**			
Describe included participants, setting and dates:<br>"Inclusion criteria included: (1) patients with renal cancer labeled as the ICD-O-3 site codes (C649, 8000-8980); (2) patients who underwent cytoreductive nephrectomy; (3) patients with pathological confirmation; (4) patients aged 18–90 years at initial diagnosis. Patients with missing data on race, distant sites (lung, liver, bone, or brain), T stage, N stage, tumor grade, or survival status were excluded from the cohort. Likewise, cases with bilateral tumors, and those with unknown surgical information were also excluded"<br><br>"The nomogram for predicting CSM was built in patients who did not receive MSX treatment"			
**Concern that the included participants and setting do not match the review question**	**CONCERN:**<br>*(low/ high/ unclear)*	Low	N/A
Rationale of applicability rating:<br>The study is only applied to patients with metastasis but the review question is focused on these patients			

DOMAIN 2: Predictors			
**A. Risk of Bias**			
List and describe predictors included in the final model, e.g. definition and timing of assessment:<br><br>The predictors included in the model were: TN stage, bone, brain, liver and lung metastasis and grade<br><br>All the predictors were measured at diagnosis			
		Dev	Val
2.1 Were predictors defined and assessed in a similar way for all participants?		PY	N/A
2.2 Were predictor assessments made without knowledge of outcome data?		PY	N/A
2.3 Are all predictors available at the time the model is intended to be used?		PY	N/A
**Risk of bias introduced by predictors or their assessment**	**RISK:**<br>*(low/ high/ unclear)*	Low	N/A
Rationale of bias rating:<br>*The blinding of measurement is unknown but all the predictors are objective.*			
**B. Applicability**			
Concern that the definition, assessment or timing of predictors in the model do not match the review question	**CONCERN:**<br>*(low/ high/ unclear)*	Low	N/A
Rationale of applicability rating:<br>*No major issues identified.*			

DOMAIN 3: Outcome			
**A. Risk of Bias**			
Describe the outcome, how it was defined and determined, and the time interval between predictor assessment and outcome determination:<br>The outcome was cancer specific survival at 1 and 5 years			
		Dev	Val
3.1 Was the outcome determined appropriately?		PY	N/A
3.2 Was a pre-specified or standard outcome definition used?		Y	N/A
3.3 Were predictors excluded from the outcome definition?		PY	N/A
3.4 Was the outcome defined and determined in a similar way for all participants?		PY	N/A
3.5 Was the outcome determined without knowledge of predictor information?		PY	N/A
3.6 Was the time interval between predictor assessment and outcome determination appropriate?		Y	N/A
**Risk of bias introduced by the outcome or its determination**	**RISK:**<br>*(low/ high/ unclear)*	Low	N/A
Rationale of bias rating:<br>*No major issues identified*			
**B. Applicability**			
At what time point was the outcome determined:<br>1 and 5 years<br><br>*If a composite outcome was used, describe the relative frequency/distribution of each contributing outcome:*<br>*N/A*			
**Concern that the outcome, its definition, timing or determination do not match the review question**	**CONCERN:**<br>*(low/ high/ unclear)*	Low	N/A
Rationale of applicability rating:<br>*The outcome of the primary study matches the outcome of interest of the review*			

DOMAIN 4: Analysis
**Risk of Bias**
Describe numbers of participants, number of candidate predictors, outcome events and events per candidate predictor:<br>"Overall, 2,911 eligible patients with mRCC were enrolled in this study. Among them, 579 (19.9%) cases underwent MSX, while 2,332 (80.1%) patients received non-MSX treatment. The median age of the entire patients at first diagnosis was 61 years (interquartile range [IQR] 54–69 years)"<br><br>The total number of predictors is 14 (table 2)<br>The number of events is unknown. They say the number of events in the total cohort but they don´t specify how many events occurred in the cohort without treatment of the metastasis<br><br>EPV= 1373/14 = 98
Describe how the model was developed (for example in regards to modelling technique (e.g. survival or logistic modelling), predictor selection, and risk group definition):<br><br>"Differences in demographic and clinical characteristics were examined using the Chi-square test for categorical variables. A univariate and multivariate Fine and Gray's competing risk analysis was performed to identify risk factors associated with cancer-specific mortality (CSM) and expressed as a hazard ratio (HR) with 95% confidence interval (CI). Non-kidney cancer- related death was considered as competing risk event for cancer- specific death"
Describe whether and how the model was validated, either internally (e.g. bootstrapping, cross validation, random split sample) or externally (e.g. temporal validation, geographical validation, different setting, different type of participants):<br><br>"A calibration curve (1,000 bootstrap resamples) was graphically generated to assess the calibration of the nomogram"
Describe the performance measures of the model, e.g. (re)calibration, discrimination, (re)classification, net benefit, and whether they were adjusted for optimism:<br><br>"Internal validation of the nomogram was performed using the concordance index (C index) value, which was used to estimate the discriminative performance of the model. A calibration curve (1,000 bootstrap resamples) was graphically generated to assess the calibration of the nomogram"
Describe any participants who were excluded from the analysis:<br><br>"Patients with missing data on race, distant sites (lung, liver, bone, or brain), T stage, N stage, tumor grade, or survival status were excluded from the cohort. Likewise, cases with bilateral tumors, and those with unknown surgical information were also excluded"<br><br>"The nomogram for predicting CSM was built in patients who did not receive MSX treatment"
Describe missing data on predictors and outcomes as well as methods used for missing data:

“Patients with missing data on race, distant sites (lung, liver, bone, or brain), T stage, N stage, tumor grade, or survival status were excluded from the cohort. Likewise, cases with bilateral tumors, and those with unknown surgical information were also excluded”

		Dev	Val
4.1 Were there a reasonable number of participants with the outcome?		Y	N/A
4.2 Were continuous and categorical predictors handled appropriately?		N	N/A
4.3 Were all enrolled participants included in the analysis?		N	N/A
4.4 Were participants with missing data handled appropriately?		N	N/A
4.5 Was selection of predictors based on univariable analysis avoided?		N	
4.6 Were complexities in the data (e.g. censoring, competing risks, sampling of controls) accounted for appropriately?		Y	N/A
4.7 Were relevant model performance measures evaluated appropriately?		Y	N/A
4.8 Were model overfitting and optimism in model performance accounted for?		Y	
4.9 Do predictors and their assigned weights in the final model correspond to the results from multivariable analysis?		PY	
**Risk of bias introduced by the analysis**	**RISK:** *(low/ high/ unclear)*	High	N/A
Rationale of bias rating: *They did categorizations. They excluded patients with missing data. They selected the predictors based on univariable.*			

Step 4: Overall assessment

Use the following tables to reach overall judgements about risk of bias and concerns regarding applicability of the prediction model evaluation (development and/or validation) across all assessed domains.
Complete for each evaluation of a distinct model.

**Reaching an overall judgement about risk of bias of the prediction model evaluation**	
**Low risk of bias**	If all domains were rated low risk of bias.<br>If a prediction model was developed without any external validation, and it was rated as low risk of bias for all domains, consider downgrading to **high risk of bias**. Such a model can only be considered as low risk of bias, if the development was based on a very large data set and included some form of internal validation.
**High risk of bias**	If at least one domain is judged to be at **high risk of bias**.
**Unclear risk of bias**	If an unclear risk of bias was noted in at least one domain and it was low risk for all other domains.

**Reaching an overall judgement about applicability of the prediction model evaluation**	
**Low concerns regarding applicability**	If low concerns regarding applicability for all domains, the prediction model evaluation is judged to have **low concerns regarding applicability**.
**High concerns regarding applicability**	If high concerns regarding applicability for at least one domain, the prediction model evaluation is judged to have **high concerns regarding applicability**.
**Unclear concerns regarding applicability**	If unclear concerns (but no "high concern") regarding applicability for at least one domain, the prediction model evaluation is judged to have **unclear concerns regarding applicability** overall.

**Overall judgement about risk of bias and applicability of the prediction model evaluation**		
**Overall judgement of risk of bias**	**RISK:**<br>*(low/ high/ unclear)*	High
Summary of sources of potential bias:<br>Analysis and participants domains show several major issues		
**Overall judgement of applicability**	**CONCERN:**<br>*(low/ high/ unclear)*	Low
Summary of applicability concerns:<br><br>No major issues identified		

PROBAST

(Prediction model study Risk Of Bias Assessment Tool)

Published in Annals of Internal Medicine (freely available):

1. PROBAST: A Tool to Assess the Risk of Bias and Applicability of Prediction Model Studies
2. PROBAST: A Tool to Assess Risk of Bias and Applicability of Prediction Model Studies: Explanation and Elaboration

What does PROBAST assess?

PROBAST assesses both the *risk of bias* and *concerns regarding applicability* of a study that evaluates (develops, validates or updates) a multivariable diagnostic or prognostic prediction model. It is designed to assess primary studies included in a systematic review.

Bias occurs if systematic flaws or limitations in the design, conduct or analysis of a primary study distort the results. For the purpose of prediction modelling studies, we have defined *risk of bias* to occur when shortcomings in the study design, conduct or analysis lead to systematically distorted estimates of a model's predictive performance or to an inadequate model to address the research question. Model predictive performance is typically evaluated using calibration, discrimination and sometimes classification measures, and these are likely inaccurately estimated in studies with high risk of bias. *Applicability* refers to the extent to which the prediction model from the primary study matches your systematic review question, for example in terms of the participants, predictors or outcome of interest.

A primary study may include the development and/or validation or update of more than one prediction model. A PROBAST assessment should be completed for each distinct model that is developed, validated or updated (extended) for making individualised predictions. Where a publication assesses multiple prediction models, only complete a PROBAST assessment for those models that meet the inclusion criteria for your systematic review. Please note that subsequent use of the term "model" includes derivatives of models, such as simplified risk scores, nomograms, or recalibrations of models.

PROBAST is not designed for all multivariable diagnostic or prognostic studies. For example, studies using multivariable models to identify predictors associated with an outcome but not attempting to develop a model for making individualised predictions are not covered by PROBAST.

PROBAST includes four steps.

Step	Task	When to complete
**1**	Specify your systematic review question(s)	Once per systematic review
**2**	Classify the type of prediction model evaluation	Once for each model of interest in each publication being assessed, for each relevant outcome
**3**	Assess risk of bias and applicability	Once for each development and validation of each distinct prediction model in a publication
**4**	Overall judgment	Once for each development and validation of each distinct prediction model in a publication

If this is your first time using PROBAST, we strongly recommend reading the detailed explanation and elaboration (E&E, see link above) paper and to check the examples on www.probast.org

Step 1: Specify your systematic review question

State your systematic review question to facilitate the assessment of the applicability of the evaluated models to your question. *The following table should be completed once per systematic review.*

Criteria	Specify your systematic review question
Intended use of model:	*To predict cancer specific mortality in patients with renal cancer treated with partial or total nephrectomy regardless of TNM stage*
***Participants** including selection criteria and setting:*	*Patients with renal cancer treated with partial or total nephrectomy regardless of TNM stage*
***Predictors** (used in prediction modelling), including types of predictors (e.g. history, clinical examination, biochemical markers, imaging tests), time of measurement, specific measurement issues (e.g., any requirements/ prohibitions for specialized equipment):*	*Predictors used in clinical practice measured when a nephrectomy for renal cancer is indicated*
Outcome to be predicted:	*Cancer specific mortality*

Step 2: Classify the type of prediction model evaluation

Use the following table to classify the evaluation as model development, model validation or model update, or combination. Different signalling questions apply for different types of prediction model evaluation. If the evaluation does not fit one of these classifications then PROBAST should not be used.

**Classify the evaluation based on its aim**			
**Type of prediction study**	**PROBAST boxes to complete**	**Tick as appropriate**	**Definition for type of prediction model study**
Development only	Development	✗	Prediction model development without external validation. These studies may include internal validation methods, such as bootstrapping and cross-validation techniques.
Development and validation	Development and validation	✓	Prediction model development combined with external validation in other participants in the same article.
Validation only	Validation	✗	External validation of existing (previously developed) model in other participants.

This table should be completed once for each publication being assessed and for each relevant outcome in your review.

**Publication reference**	Zhanghuang C, Wang J, Zhang Z, Jin L, Tan X, Mi T, Liu J, Li M and He D. A Web-Based Prediction Model for Cancer-Specific Survival of Elderly Patients With Clear Cell Renal Cell Carcinoma: A Population-Based Study. Front. Public Health 9; 2022. 833970. doi: 10.3389/fpubh.2021.833970
**Models of interest**	Nomogram
**Outcome of interest**	*Cancer specific mortality*

Step 3: Assess risk of bias and applicability

PROBAST is structured as four key domains. Each domain is judged for risk of bias (low, high or unclear) and includes signalling questions to help make judgements. Signalling questions are rated as yes (Y), probably yes (PY), probably no (PN), no (N) or no information (NI). All signalling questions are phrased so that “yes” indicates absence of bias. Any signalling question rated as “no” or “probably no” flags the potential for bias; you will need to use your judgement to determine whether the domain should be rated as “high”, “low” or “unclear” risk of bias. The guidance document contains further instructions and examples on rating signalling questions and risk of bias for each domain.

The first three domains are also rated for concerns regarding applicability (low/ high/ unclear) to your review question defined above.

Complete all domains separately for each evaluation of a distinct model. Shaded boxes indicate where signalling questions do not apply and should not be answered.

DOMAIN 1: Participants			
**A. Risk of Bias**			
Describe the sources of data and criteria for participant selection:<br><br>*"All elderly patients with ccRCC from 2004 to 2018 from the SEER program of the National Cancer Institute"*<br>*"All the demographic information of ccRCC in elderly patients (age, sex, race, year of diagnosis, and marriage), clinical pathologic information (laterality, tumor size, histological grade, and tumor, nodes, and metastases [TNM] stage), treatment (surgery, radiation therapy, and chemotherapy), follow-up information (survival status, survival time, and the cause of death) were collected. Inclusion criteria were as follows: (1) pathological diagnosis of ccRCC (International Classification of Diseases for Oncology [ICD-O]-3 codes, 8310); (2) age ≥65; (3) the years of diagnosis were 2004–2018; and (4) unilateral renal tumor. Exclusion criteria were as follows:(1) tumor histological grade is unknown; (2) tumor size is unknown; (3) unknown surgical method; (4) survival time <1 month; and (5) incomplete follow-up information."*			
		Dev	Val
1.1 Were appropriate data sources used, e.g. cohort, RCT or nested case-control study data?		Y	Y
1.2 Were all inclusions and exclusions of participants appropriate?		Y	Y
**Risk of bias introduced by selection of participants**	**RISK:**<br>*(low/ high/ unclear)*	Low	Low
Rationale of bias rating:<br>*No major issues identified*			
**B. Applicability**			
Describe included participants, setting and dates:<br>*"All elderly patients with ccRCC from 2004 to 2018 from the SEER program of the National Cancer Institute"*<br>*"All the demographic information of ccRCC in elderly patients (age, sex, race, year of diagnosis, and marriage), clinical pathologic information (laterality, tumor size, histological grade, and tumor, nodes, and metastases [TNM] stage), treatment (surgery, radiation therapy, and chemotherapy), follow-up information (survival status, survival time, and the cause of death) were collected. Inclusion criteria were as follows: (1) pathological diagnosis of ccRCC (International Classification of Diseases for Oncology [ICD-O]-3 codes, 8310); (2) age ≥65; (3) the years of diagnosis were 2004–2018; and (4) unilateral renal tumor. Exclusion criteria were as follows:(1) tumor histological grade is unknown; (2) tumor size is unknown; (3) unknown surgical method; (4) survival time <1 month; and (5) incomplete follow-up information."*			
**Concern that the included participants and setting do not match the review question**	**CONCERN:**<br>*(low/ high/ unclear)*	Low	Low
Rationale of applicability rating:<br>*No major issues identified*			

DOMAIN 2: Predictors			
**A. Risk of Bias**			
List and describe predictors included in the final model, e.g. definition and timing of assessment:<br><br>The predictors included in the model were: age, sex, race, marriage, grade, TNM stage, surgery, tumor size. The predictors were measured before and after treatment			
		Dev	Val
2.1 Were predictors defined and assessed in a similar way for all participants?		PY	PY
2.2 Were predictor assessments made without knowledge of outcome data?		PY	PY
2.3 Are all predictors available at the time the model is intended to be used?		PY	PY
**Risk of bias introduced by predictors or their assessment**	**RISK:**<br>*(low/ high/ unclear)*	Low	Low
Rationale of bias rating:<br>The blinding of measurement is unknown but all the predictors are objectives			
**B. Applicability**			
Concern that the definition, assessment or timing of predictors in the model do not match the review question	**CONCERN:**<br>*(low/ high/ unclear)*	Low	Low
Rationale of applicability rating:<br>*No major issues identified.*			

DOMAIN 3: Outcome			
**A. Risk of Bias**			
Describe the outcome, how it was defined and determined, and the time interval between predictor assessment and outcome determination:<br><br>The outcome was cancer specific survival at 1, 3 and 5 years			
		Dev	Val
3.1 Was the outcome determined appropriately?		Y	Y
3.2 Was a pre-specified or standard outcome definition used?		Y	Y
3.3 Were predictors excluded from the outcome definition?		PY	PY
3.4 Was the outcome defined and determined in a similar way for all participants?		PY	PY
3.5 Was the outcome determined without knowledge of predictor information?		PY	PY
3.6 Was the time interval between predictor assessment and outcome determination appropriate?		Y	Y
**Risk of bias introduced by the outcome or its determination**	**RISK:**<br>*(low/ high/ unclear)*	Low	Low
Rationale of bias rating:<br>*No major issues identified*			
**B. Applicability**			
At what time point was the outcome determined:<br>1, 3 and 5 years<br><br>*If a composite outcome was used, describe the relative frequency/distribution of each contributing outcome:*<br>*N/A*			
**Concern that the outcome, its definition, timing or determination do not match the review question**	**CONCERN:**<br>*(low/ high/ unclear)*	Low	Low
Rationale of applicability rating:<br>*The outcome of the primary study matches the outcome of interest of the review*			

DOMAIN 4: Analysis		
**Risk of Bias**		
Describe numbers of participants, number of candidate predictors, outcome events and events per candidate predictor:<br><br>*"A total of 33,509 elderly patients with ccRCC were enrolled. All patients were randomly divided into the training cohort (N = 23,412) and the validation cohort (N = 10,097)"*<br><br>*A total of 20 predictors were included (Table 2)*<br>*The number of events is unknown*<br><br>*EPV= unknown*		
Describe how the model was developed (for example in regards to modelling technique (e.g. survival or logistic modelling), predictor selection, and risk group definition):<br><br>*"Univariate Cox regression models were used in the training cohort to analyze risk factors associated with in patients with CSS. According to univariate Cox regression analysis, a multivariate Cox regression model was established to identify the independent risk factors for CSS"*		
Describe whether and how the model was validated, either internally (e.g. bootstrapping, cross validation, random split sample) or externally (e.g. temporal validation, geographical validation, different setting, different type of participants):<br><br>*"Calibration curves of 1,000 bootstrap samples were used to test the accuracy of the prediction model"*		
Describe the performance measures of the model, e.g. (re)calibration, discrimination, (re)classification, net benefit, and whether they were adjusted for optimism:<br><br>*"Subsequently, we used the consistency index (C-index) to test the accuracy and discrimination of the prediction model in the training cohort and validation cohort".*<br>*"The area under the receiver operating curve (AUC) was used to test the model's prediction accuracy at 1-, 3-, and 5 years".*<br>*"We used the decision analysis curve (DCA) to validate the clinical potential of the predictive model".*		
Describe any participants who were excluded from the analysis:<br>*"Exclusion criteria were as follows:(1) tumor histological grade is unknown; (2) tumor size is unknown; (3) unknown surgical method; (4) survival time <1 month; and (5) incomplete follow-up information".*		
Describe missing data on predictors and outcomes as well as methods used for missing data:<br>*"Exclusion criteria were as follows:(1) tumor histological grade is unknown; (2) tumor size is unknown; (3) unknown surgical method; (4) survival time <1 month; and (5) incomplete follow-up information"*		
	Dev	Val
4.1 Were there a reasonable number of participants with the outcome?	NI	PY
4.2 Were continuous and categorical predictors handled appropriately?	Y	Y
4.3 Were all enrolled participants included in the analysis?	Y	Y
4.4 Were participants with missing data handled appropriately?	N	N
4.5 Was selection of predictors based on univariable analysis avoided?	N	
4.6 Were complexities in the data (e.g. censoring, competing risks, sampling of controls) accounted for appropriately?	N	N

4.7 Were relevant model performance measures evaluated appropriately?		Y	Y
4.8 Were model overfitting and optimism in model performance accounted for?		Y	
4.9 Do predictors and their assigned weights in the final model correspond to the results from multivariable analysis?		PY	
**Risk of bias introduced by the analysis**	**RISK:** *(low/ high/ unclear)*	High	High
Rationale of bias rating: *They didn´t say the number of events in each cohort, but according the number of total events probably the are enough events per variable on the validation cohort. They excluded patients with missing data. They selected the predictors based on univariable. They didn't use competing risks.*			

Step 4: Overall assessment

Use the following tables to reach overall judgements about risk of bias and concerns regarding applicability of the prediction model evaluation (development and/or validation) across all assessed domains.
Complete for each evaluation of a distinct model.

Reaching an overall judgement about risk of bias of the prediction model evaluation	
**Low risk of bias**	If all domains were rated low risk of bias.<br>If a prediction model was developed without any external validation, and it was rated as low risk of bias for all domains, consider downgrading to **high risk of bias**. Such a model can only be considered as low risk of bias, if the development was based on a very large data set and included some form of internal validation.
**High risk of bias**	If at least one domain is judged to be at **high risk of bias**.
**Unclear risk of bias**	If an unclear risk of bias was noted in at least one domain and it was low risk for all other domains.

Reaching an overall judgement about applicability of the prediction model evaluation	
**Low concerns regarding applicability**	If low concerns regarding applicability for all domains, the prediction model evaluation is judged to have **low concerns regarding applicability**.
**High concerns regarding applicability**	If high concerns regarding applicability for at least one domain, the prediction model evaluation is judged to have **high concerns regarding applicability**.
**Unclear concerns regarding applicability**	If unclear concerns (but no "high concern") regarding applicability for at least one domain, the prediction model evaluation is judged to have **unclear concerns regarding applicability** overall.

Overall judgement about risk of bias and applicability of the prediction model evaluation		
**Overall judgement of risk of bias**	**RISK:**<br>*(low/ high/ unclear)*	High
Summary of sources of potential bias:<br>Analysis domain shows several major issues		
**Overall judgement of applicability**	**CONCERN:**<br>*(low/ high/ unclear)*	Low
Summary of applicability concerns:<br><br>No major issues		

PROBAST

(Prediction model study Risk Of Bias Assessment Tool)

Published in Annals of Internal Medicine (freely available):

1. PROBAST: A Tool to Assess the Risk of Bias and Applicability of Prediction Model Studies
2. PROBAST: A Tool to Assess Risk of Bias and Applicability of Prediction Model Studies: Explanation and Elaboration

What does PROBAST assess?

PROBAST assesses both the *risk of bias* and *concerns regarding applicability* of a study that evaluates (develops, validates or updates) a multivariable diagnostic or prognostic prediction model. It is designed to assess primary studies included in a systematic review.

Bias occurs if systematic flaws or limitations in the design, conduct or analysis of a primary study distort the results. For the purpose of prediction modelling studies, we have defined *risk of bias* to occur when shortcomings in the study design, conduct or analysis lead to systematically distorted estimates of a model's predictive performance or to an inadequate model to address the research question. Model predictive performance is typically evaluated using calibration, discrimination and sometimes classification measures, and these are likely inaccurately estimated in studies with high risk of bias. *Applicability* refers to the extent to which the prediction model from the primary study matches your systematic review question, for example in terms of the participants, predictors or outcome of interest.

A primary study may include the development and/or validation or update of more than one prediction model. A PROBAST assessment should be completed for each distinct model that is developed, validated or updated (extended) for making individualised predictions. Where a publication assesses multiple prediction models, only complete a PROBAST assessment for those models that meet the inclusion criteria for your systematic review. Please note that subsequent use of the term "model" includes derivatives of models, such as simplified risk scores, nomograms, or recalibrations of models.

PROBAST is not designed for all multivariable diagnostic or prognostic studies. For example, studies using multivariable models to identify predictors associated with an outcome but not attempting to develop a model for making individualised predictions are not covered by PROBAST.

PROBAST includes four steps.

Step	Task	When to complete
**1**	Specify your systematic review question(s)	Once per systematic review
**2**	Classify the type of prediction model evaluation	Once for each model of interest in each publication being assessed, for each relevant outcome
**3**	Assess risk of bias and applicability	Once for each development and validation of each distinct prediction model in a publication
**4**	Overall judgment	Once for each development and validation of each distinct prediction model in a publication

If this is your first time using PROBAST, we strongly recommend reading the detailed explanation and elaboration (E&E, see link above) paper and to check the examples on www.probast.org

Step 1: Specify your systematic review question

State your systematic review question to facilitate the assessment of the applicability of the evaluated models to your question. *The following table should be completed once per systematic review.*

Criteria	Specify your systematic review question
Intended use of model:	*To predict cancer specific mortality in patients with renal cancer treated with partial or total nephrectomy regardless of TNM stage*
***Participants** including selection criteria and setting:*	*Patients with renal cancer treated with partial or total nephrectomy regardless of TNM stage*
***Predictors** (used in prediction modelling), including types of predictors (e.g. history, clinical examination, biochemical markers, imaging tests), time of measurement, specific measurement issues (e.g., any requirements/ prohibitions for specialized equipment):*	*Predictors used in clinical practice measured when a nephrectomy for renal cancer is indicated*
Outcome to be predicted:	*Cancer specific mortality*

Step 2: Classify the type of prediction model evaluation

Use the following table to classify the evaluation as model development, model validation or model update, or combination. Different signalling questions apply for different types of prediction model evaluation. If the evaluation does not fit one of these classifications then PROBAST should not be used.

**Classify the evaluation based on its aim**			
**Type of prediction study**	**PROBAST boxes to complete**	**Tick as appropriate**	**Definition for type of prediction model study**
Development only	Development	✗	Prediction model development without external validation. These studies may include internal validation methods, such as bootstrapping and cross-validation techniques.
Development and validation	Development and validation	✓	Prediction model development combined with external validation in other participants in the same article.
Validation only	Validation	✗	External validation of existing (previously developed) model in other participants.

This table should be completed once for each publication being assessed and for each relevant outcome in your review.

**Publication reference**	Huang G, Liao J, Cai S, Chen Z, Qin X, Ba L, Rao J, Zhong W, Lin Y, Liang Y, Wei L, Li J, Deng K, Li X, Guo Z, Wang L and Zhuo Y. Development and validation of a prognostic nomogram for predicting cancer-specific survival in patients with metastatic clear cell renal carcinoma: A study based on SEER database. Front. Oncol. 2022; 12:949058. doi: 10.3389/fonc.2022.949058
**Models of interest**	Nomogram and risk score
**Outcome of interest**	*Cancer specific mortality*

Step 3: Assess risk of bias and applicability

PROBAST is structured as four key domains. Each domain is judged for risk of bias (low, high or unclear) and includes signalling questions to help make judgements. Signalling questions are rated as yes (Y), probably yes (PY), probably no (PN), no (N) or no information (NI). All signalling questions are phrased so that "yes" indicates absence of bias. Any signalling question rated as "no" or "probably no" flags the potential for bias; you will need to use your judgement to determine whether the domain should be rated as "high", "low" or "unclear" risk of bias. The guidance document contains further instructions and examples on rating signalling questions and risk of bias for each domain.

The first three domains are also rated for concerns regarding applicability (low/ high/ unclear) to your review question defined above.

Complete all domains separately for each evaluation of a distinct model. Shaded boxes indicate where signalling questions do not apply and should not be answered.

**DOMAIN 1: Participants**			
**A. Risk of Bias**			
Describe the sources of data and criteria for participant selection:<br><br>*“Data of patients diagnosed with mccRCC were collected from the Surveillance, Epidemiology, and End Results (SEER) database (https://seer.cancer.gov/) according to the International Classification of Tumor Diseases Third Edition (ICD-O-3). The inclusion criteria were as follows (1): The first diagnosis was primary clear cell renal cell carcinoma; (2) Distant organ metastasis, including lung, liver, brain, and bone metastases;*<br>*(3) Unilateral primary clear cell renal cell carcinoma; (4) The age of diagnosis was 18 years or older; (5) The registration information is complete. The exclusion criteria were as follows: (1) Non primary clear cell renal cell carcinoma; (2) Bilateral or lateral unclear primary metastatic clear cell renal cell carcinoma; (3) Other organ metastases (non-lung, non-liver, non-brain, non-bone, and other organ metastasis); (4) Race, T-stage, N-stage or histological grade were unknown; (5) Cases with incomplete information. After screening, 1790 eligible mccRCC patients were finally included in the cohort”*			
		Dev	Val
1.1 Were appropriate data sources used, e.g. cohort, RCT or nested case-control study data?		Y	Y
1.2 Were all inclusions and exclusions of participants appropriate?		Y	Y
**Risk of bias introduced by selection of participants**	**RISK:**<br>*(low/ high/ unclear)*	Low	Low
Rationale of bias rating:<br>*No major issues identified*			
**B. Applicability**			
Describe included participants, setting and dates:<br>*“Data of patients diagnosed with mccRCC were collected from the Surveillance, Epidemiology, and End Results (SEER) database (https://seer.cancer.gov/) according to the International Classification of Tumor Diseases Third Edition (ICD-O-3). The inclusion criteria were as follows (1): The first diagnosis was primary clear cell renal cell carcinoma; (2) Distant organ metastasis, including lung, liver, brain, and bone metastases;*<br>*(3) Unilateral primary clear cell renal cell carcinoma; (4) The age of diagnosis was 18 years or older; (5) The registration information is complete. The exclusion criteria were as follows: (1) Non primary clear cell renal cell carcinoma; (2) Bilateral or lateral unclear primary metastatic clear cell renal cell carcinoma; (3) Other organ metastases (non-lung, non-liver, non-brain, non-bone, and other organ metastasis); (4) Race, T-stage, N-stage or histological grade were unknown; (5) Cases with incomplete information. After screening, 1790 eligible mccRCC patients were finally included in the cohort”*			
**Concern that the included participants and setting do not match the review question**	**CONCERN:**<br>*(low/ high/ unclear)*	High	High
Rationale of applicability rating:<br>*Cohort study not included patients with other organ metastases (non-lung, non-liver, non-brain, non-bone, and other organ metastasis)*			

DOMAIN 2: Predictors			
**A. Risk of Bias**			
List and describe predictors included in the final model, e.g. definition and timing of assessment:<br><br>The predictors included in the model were: age, TN stage, bone, brain, liver and lung metastases, radiotherapy, surgery and histological grade.<br><br>The predictors were measured before and after treatment			
		Dev	Val
2.1 Were predictors defined and assessed in a similar way for all participants?		PY	PY
2.2 Were predictor assessments made without knowledge of outcome data?		PY	PY
2.3 Are all predictors available at the time the model is intended to be used?		PY	PY
**Risk of bias introduced by predictors or their assessment**	**RISK:**<br>*(low/ high/ unclear)*	Low	Low
Rationale of bias rating:<br>*The blinding of measurement is unknown but all the predictors are objective.*			
**B. Applicability**			
Concern that the definition, assessment or timing of predictors in the model do not match the review question	**CONCERN:**<br>*(low/ high/ unclear)*	Low	Low
Rationale of applicability rating:<br>*No major issues identified.*			

DOMAIN 3: Outcome			
**A. Risk of Bias**			
Describe the outcome, how it was defined and determined, and the time interval between predictor assessment and outcome determination:<br>The outcome was cancer specific survival at 1, 3 and 5 years			
		Dev	Val
3.1 Was the outcome determined appropriately?		PY	PY
3.2 Was a pre-specified or standard outcome definition used?		Y	Y
3.3 Were predictors excluded from the outcome definition?		PY	PY
3.4 Was the outcome defined and determined in a similar way for all participants?		PY	PY
3.5 Was the outcome determined without knowledge of predictor information?		PY	PY
3.6 Was the time interval between predictor assessment and outcome determination appropriate?		Y	Y
**Risk of bias introduced by the outcome or its determination**	**RISK:**<br>*(low/ high/ unclear)*	Low	Low
Rationale of bias rating:<br>*No major issues identified*			
**B. Applicability**			
At what time point was the outcome determined:<br>1, 3 and 5 years<br><br>*If a composite outcome was used, describe the relative frequency/distribution of each contributing outcome:*<br>*N/A*			
**Concern that the outcome, its definition, timing or determination do not match the review question**	**CONCERN:**<br>*(low/ high/ unclear)*	Low	Low
Rationale of applicability rating:<br>*The outcome of the primary study matches the outcome of interest of the review*			

DOMAIN 4: Analysis
**Risk of Bias**
Describe numbers of participants, number of candidate predictors, outcome events and events per candidate predictor:<br>*"After screening, 1790 eligible mccRCC patients were finally included in the cohort. The process of data was shown in Figure 1. Patients were randomly divided into two sets (training set, n = 1253, and validation set, n = 537) based on the ratio of 7:3"*<br><br>*A total of 18 predictors were included (Table 2)*<br>*The number of events is unknown*<br><br>*EPV= unknown*
Describe how the model was developed (for example in regards to modelling technique (e.g. survival or logistic modelling), predictor selection, and risk group definition):<br><br>*"The univariate and multivariate Cox proportional risk regression analyses were performed to demonstrate the association between the selected variables and oncologic outcomes, the univariate and multivariate Cox regression analysis of included variables for CSS in training set are shown in Table 2. The univariate Cox regression analysis identified nine variables (T-stage, N-stage, brain metastases, liver metastases, lung metastases, chemotherapy, radiotherapy, surgery, and histological grade) as factors associated with CSS. Although the statistical analysis showed that age and bone metastases were not statistically significant, they were included in the multivariate analysis together, considering their influence on patient prognosis in terms of professional significance"*
Describe whether and how the model was validated, either internally (e.g. bootstrapping, cross validation, random split sample) or externally (e.g. temporal validation, geographical validation, different setting, different type of participants):<br><br>*"A nomogram was built using the significant prognostic factors for 1-, 3-, and 5-year CSS (Figure 3), which was then validated internally using data from the validation set"*
Describe the performance measures of the model, e.g. (re)calibration, discrimination, (re)classification, net benefit, and whether they were adjusted for optimism:<br><br>*"The predictive discriminative ability of the nomogram was determined by Harrell's concordance index (C-index) and the receiver operating characteristics (ROC) curves, the area under the curve (AUC). The accuracy of the nomogram in predicting 1 -, 3 - and 5-year CSS was evaluated by Calibration plots. In addition, the net reclassification improvement (NRI) and integrated discrimination improvement (IDI) were used to assess whether the nomogram was more accurate than the AJCC TNM staging system or not. And decision curve analysis (DCA) was used to evaluate the clinical utility of the nomogram"*
Describe any participants who were excluded from the analysis:<br>*"The exclusion criteria were as follows: (1) Non primary clear cell renal cell carcinoma; (2) Bilateral or lateral unclear primary metastatic clear cell renal cell carcinoma; (3) Other organ metastases (non-lung, non-liver, non-brain, non-bone, and other organ metastasis); (4) Race, T- stage, N-stage or histological grade were unknown; (5) Cases with incomplete information. After screening, 1790 eligible mccRCC patients were finally included in the cohort"*

Describe missing data on predictors and outcomes as well as methods used for missing data:<br>*"The exclusion criteria were as follows: (1) Non primary clear cell renal cell carcinoma; (2) Bilateral or lateral unclear primary metastatic clear cell renal cell carcinoma; (3) Other organ metastases (non-lung, non-liver, non-brain, non-bone, and other organ metastasis); (4) Race, T- stage, N-stage or histological grade were unknown; (5) Cases with incomplete information. After screening, 1790 eligible mccRCC patients were finally included in the cohort"*

		Dev	Val
4.1 Were there a reasonable number of participants with the outcome?		NI	PY
4.2 Were continuous and categorical predictors handled appropriately?		N	N
4.3 Were all enrolled participants included in the analysis?		PY	PY
4.4 Were participants with missing data handled appropriately?		N	N
4.5 Was selection of predictors based on univariable analysis avoided?		N	
4.6 Were complexities in the data (e.g. censoring, competing risks, sampling of controls) accounted for appropriately?		N	N
4.7 Were relevant model performance measures evaluated appropriately?		Y	Y
4.8 Were model overfitting and optimism in model performance accounted for?		N	
4.9 Do predictors and their assigned weights in the final model correspond to the results from multivariable analysis?		PY	
**Risk of bias introduced by the analysis**	**RISK:**<br>*(low/ high/ unclear)*	High	High

Rationale of bias rating:<br>*They didn´t say the number of events. They did categorizations. They excluded patients with missing data. They selected the predictors based on univariable. They didn't use competing risks. They didn´t do bootstrapping*

Step 4: Overall assessment

Use the following tables to reach overall judgements about risk of bias and concerns regarding applicability of the prediction model evaluation (development and/or validation) across all assessed domains.
Complete for each evaluation of a distinct model.

Reaching an overall judgement about risk of bias of the prediction model evaluation	
**Low risk of bias**	If all domains were rated low risk of bias.<br>If a prediction model was developed without any external validation, and it was rated as low risk of bias for all domains, consider downgrading to **high risk of bias**. Such a model can only be considered as low risk of bias, if the development was based on a very large data set and included some form of internal validation.
**High risk of bias**	If at least one domain is judged to be at **high risk of bias**.
**Unclear risk of bias**	If an unclear risk of bias was noted in at least one domain and it was low risk for all other domains.

Reaching an overall judgement about applicability of the prediction model evaluation	
**Low concerns regarding applicability**	If low concerns regarding applicability for all domains, the prediction model evaluation is judged to have **low concerns regarding applicability**.
**High concerns regarding applicability**	If high concerns regarding applicability for at least one domain, the prediction model evaluation is judged to have **high concerns regarding applicability**.
**Unclear concerns regarding applicability**	If unclear concerns (but no "high concern") regarding applicability for at least one domain, the prediction model evaluation is judged to have **unclear concerns regarding applicability** overall.

Overall judgement about risk of bias and applicability of the prediction model evaluation		
**Overall judgement of risk of bias**	**RISK:** *(low/ high/ unclear)*	High
Summary of sources of potential bias:<br>Analysis domain shows several major issues		
**Overall judgement of applicability**	**CONCERN:** *(low/ high/ unclear)*	High
Summary of applicability concerns:<br><br>Participants domain shows several major issues		

PROBAST

(Prediction model study Risk Of Bias Assessment Tool)

Published in Annals of Internal Medicine (freely available):

1. PROBAST: A Tool to Assess the Risk of Bias and Applicability of Prediction Model Studies
2. PROBAST: A Tool to Assess Risk of Bias and Applicability of Prediction Model Studies: Explanation and Elaboration

What does PROBAST assess?

PROBAST assesses both the *risk of bias* and *concerns regarding applicability* of a study that evaluates (develops, validates or updates) a multivariable diagnostic or prognostic prediction model. It is designed to assess primary studies included in a systematic review.

Bias occurs if systematic flaws or limitations in the design, conduct or analysis of a primary study distort the results. For the purpose of prediction modelling studies, we have defined *risk of bias* to occur when shortcomings in the study design, conduct or analysis lead to systematically distorted estimates of a model's predictive performance or to an inadequate model to address the research question. Model predictive performance is typically evaluated using calibration, discrimination and sometimes classification measures, and these are likely inaccurately estimated in studies with high risk of bias. *Applicability* refers to the extent to which the prediction model from the primary study matches your systematic review question, for example in terms of the participants, predictors or outcome of interest.

A primary study may include the development and/or validation or update of more than one prediction model. A PROBAST assessment should be completed for each distinct model that is developed, validated or updated (extended) for making individualised predictions. Where a publication assesses multiple prediction models, only complete a PROBAST assessment for those models that meet the inclusion criteria for your systematic review. Please note that subsequent use of the term "model" includes derivatives of models, such as simplified risk scores, nomograms, or recalibrations of models.

PROBAST is not designed for all multivariable diagnostic or prognostic studies. For example, studies using multivariable models to identify predictors associated with an outcome but not attempting to develop a model for making individualised predictions are not covered by PROBAST.

PROBAST includes four steps.

Step	Task	When to complete
**1**	Specify your systematic review question(s)	Once per systematic review
**2**	Classify the type of prediction model evaluation	Once for each model of interest in each publication being assessed, for each relevant outcome
**3**	Assess risk of bias and applicability	Once for each development and validation of each distinct prediction model in a publication
**4**	Overall judgment	Once for each development and validation of each distinct prediction model in a publication

If this is your first time using PROBAST, we strongly recommend reading the detailed explanation and elaboration (E&E, see link above) paper and to check the examples on www.probast.org

Step 1: Specify your systematic review question

State your systematic review question to facilitate the assessment of the applicability of the evaluated models to your question. *The following table should be completed once per systematic review.*

Criteria	Specify your systematic review question
Intended use of model:	*To predict cancer specific mortality in patients with renal cancer treated with partial or total nephrectomy regardless of TNM stage*
***Participants** including selection criteria and setting:*	*Patients with renal cancer treated with partial or total nephrectomy regardless of TNM stage*
***Predictors** (used in prediction modelling), including types of predictors (e.g. history, clinical examination, biochemical markers, imaging tests), time of measurement, specific measurement issues (e.g., any requirements/ prohibitions for specialized equipment):*	*Predictors used in clinical practice measured when a nephrectomy for renal cancer is indicated*
Outcome to be predicted:	*Cancer specific mortality*

Step 2: Classify the type of prediction model evaluation

Use the following table to classify the evaluation as model development, model validation or model update, or combination. Different signalling questions apply for different types of prediction model evaluation. If the evaluation does not fit one of these classifications then PROBAST should not be used.

Classify the evaluation based on its aim			
**Type of prediction study**	**PROBAST boxes to complete**	**Tick as appropriate**	**Definition for type of prediction model study**
Development only	Development	✗	Prediction model development without external validation. These studies may include internal validation methods, such as bootstrapping and cross-validation techniques.
Development and validation	Development and validation	✓	Prediction model development combined with external validation in other participants in the same article.
Validation only	Validation	✗	External validation of existing (previously developed) model in other participants.

This table should be completed once for each publication being assessed and for each relevant outcome in your review.

**Publication reference**	Zhou Y, Zhang R, Ding Y, et al. Prognostic nomograms and Aggtrmmns scoring system for predicting overall survival and cancerspecific survival of patients with kidney cancer. Cancer Med. 2020;00:1–13. https ://doi.org/10.1002/cam4.2916
**Models of interest**	Nomogram and risk score
**Outcome of interest**	*Cancer specific mortality*

Step 3: Assess risk of bias and applicability

PROBAST is structured as four key domains. Each domain is judged for risk of bias (low, high or unclear) and includes signalling questions to help make judgements. Signalling questions are rated as yes (Y), probably yes (PY), probably no (PN), no (N) or no information (NI). All signalling questions are phrased so that “yes” indicates absence of bias. Any signalling question rated as “no” or “probably no” flags the potential for bias; you will need to use your judgement to determine whether the domain should be rated as “high”, “low” or “unclear” risk of bias. The guidance document contains further instructions and examples on rating signalling questions and risk of bias for each domain.

The first three domains are also rated for concerns regarding applicability (low/ high/ unclear) to your review question defined above.

Complete all domains separately for each evaluation of a distinct model. Shaded boxes indicate where signalling questions do not apply and should not be answered.

DOMAIN 1: Participants			
**A. Risk of Bias**			
Describe the sources of data and criteria for participant selection:<br><br>*"Patients´ data in this study were obtained from Surveillance,* Epidemiology, and End Results (SEER) database"<br>"Patients diagnosed with kidney cancer between 2005 and 2015 from the SEER database were selected for this study. All data on kidney cancer patients were collected from hospitals, and no patient had a history of another cancer. Patients without confirmed pathological results were excluded. Available patient information, including age, gender, race, marital status, pathological grade, surgery, T (extent of tumor invasion) stage, N (regional lymph node) stage, and M (distant metastasis) stage was collected. We excluded patients in which the information mentioned above was missing"			
		Dev	Val
1.1 Were appropriate data sources used, e.g. cohort, RCT or nested case-control study data?		Y	Y
1.2 Were all inclusions and exclusions of participants appropriate?		Y	Y
**Risk of bias introduced by selection of participants**	**RISK:**<br>*(low/ high/ unclear)*	Low	Low
Rationale of bias rating:<br>*No major issues*			
**B. Applicability**			
Describe included participants, setting and dates:<br>*"Patients´ data in this study were obtained from Surveillance,* Epidemiology, and End Results (SEER) database"<br>"Patients diagnosed with kidney cancer between 2005 and 2015 from the SEER database were selected for this study. All data on kidney cancer patients were collected from hospitals, and no patient had a history of another cancer. Patients without confirmed pathological results were excluded. Available patient information, including age, gender, race, marital status, pathological grade, surgery, T (extent of tumor invasion) stage, N (regional lymph node) stage, and M (distant metastasis) stage was collected. We excluded patients in which the information mentioned above was missing"			
**Concern that the included participants and setting do not match the review question**	**CONCERN:**<br>*(low/ high/ unclear)*	Low	Low
Rationale of applicability rating:<br>*Cohort study with clear inclusion and exclusion criterial*			

DOMAIN 2: Predictors			
**A. Risk of Bias**			
List and describe predictors included in the final model, e.g. definition and timing of assessment:<br><br>The predictors included in the model were: surgery, T and M stage, grade, age, sex and marital status.<br><br>The predictors were measured before and after treatment			
		Dev	Val
2.1 Were predictors defined and assessed in a similar way for all participants?		PY	PY
2.2 Were predictor assessments made without knowledge of outcome data?		PY	PY
2.3 Are all predictors available at the time the model is intended to be used?		PY	PY
**Risk of bias introduced by predictors or their assessment**	**RISK:** *(low/ high/ unclear)*	Low	Low
Rationale of bias rating:<br>*The blinding of measurement is unknown but all the predictors are objective.*			
**B. Applicability**			
Concern that the definition, assessment or timing of predictors in the model do not match the review question	**CONCERN:** *(low/ high/ unclear)*	Low	Low
Rationale of applicability rating:<br>*No major issues identified.*			

DOMAIN 3: Outcome			
**A. Risk of Bias**			
Describe the outcome, how it was defined and determined, and the time interval between predictor assessment and outcome determination:<br>The outcome was cancer specific survival at 1, 3 and 5 years			
		Dev	Val
3.1 Was the outcome determined appropriately?		PY	PY
3.2 Was a pre-specified or standard outcome definition used?		Y	Y
3.3 Were predictors excluded from the outcome definition?		PY	PY
3.4 Was the outcome defined and determined in a similar way for all participants?		PY	PY
3.5 Was the outcome determined without knowledge of predictor information?		PY	PY
3.6 Was the time interval between predictor assessment and outcome determination appropriate?		Y	Y
**Risk of bias introduced by the outcome or its determination**	**RISK:**<br>*(low/ high/ unclear)*	Low	Low
Rationale of bias rating:<br>*No major issues identified*			
**B. Applicability**			
At what time point was the outcome determined:<br>1, 3 and 5 years<br><br>*If a composite outcome was used, describe the relative frequency/distribution of each contributing outcome:*<br>*N/A*			
**Concern that the outcome, its definition, timing or determination do not match the review question**	**CONCERN:**<br>*(low/ high/ unclear)*	Low	Low
Rationale of applicability rating:<br>*The outcome of the primary study matches the outcome of interest of the review*			

DOMAIN 4: Analysis
**Risk of Bias**
Describe numbers of participants, number of candidate predictors, outcome events and events per candidate predictor:<br>*"A total of 70 481 patients diagnosed with kidney cancer between 2005 and 2015 were screened from the SEER database. Patients diagnosed between 2005 and 2011 (n = 42 890), as the primary cohort, were used to establish the nomograms. Patients diagnosed between 2012 and 2013 (n = 13 094) and between 2014 and 2015 (n = 14 497) were used for the external validation of 3- and 1-year survival, respectively"*<br><br>*A total of 23 predictors were included (Table 3)*<br>*The number of events is unknown*<br><br>*EPV= unknown*
Describe how the model was developed (for example in regards to modelling technique (e.g. survival or logistic modelling), predictor selection, and risk group definition):<br><br>*"Univariate and multivariate Cox analyses were used to determine the independent prognostic factors. In the univariate Cox proportional hazard model, variables with P < .05 were further analyzed in the multivariate Cox proportional hazard model. Significant prognostic factors were used to establish nomograms to predict the 1-, 3-, and 5-year OS and CSS rates, and the ability for survival prediction of the factors was tested by receiver operating characteristic (ROC) curve"*
Describe whether and how the model was validated, either internally (e.g. bootstrapping, cross validation, random split sample) or externally (e.g. temporal validation, geographical validation, different setting, different type of participants):<br><br>*"Internal and external validations were used to evaluate the prognostic accuracy of the nomograms"*<br>*"Patients diagnosed between 2012 and 2015 were used as the validation cohort for external validation"*
Describe the performance measures of the model, e.g. (re)calibration, discrimination, (re)classification, net benefit, and whether they were adjusted for optimism:<br><br>*"We used calibration curve and concordance index (C-index) to internal and external evaluate the predictive accuracy of the nomograms (bootstraps with 1000 resample)"*<br>*"We also used propensity score matching (PSM) to balance the clinical information and reduce statistical bias"*
Describe any participants who were excluded from the analysis:<br>*"Patients without confirmed pathological results were excluded. Available patient information, including age, gender, race, marital sta- tus, pathological grade, surgery, T (extent of tumor invasion) stage, N (regional lymph node) stage, and M (distant metastasis) stage was collected. We excluded patients in which the information mentioned above was missing"*
Describe missing data on predictors and outcomes as well as methods used for missing data:<br>*"Patients without confirmed pathological results were excluded. Available patient information, including age, gender, race, marital sta- tus, pathological grade, surgery, T (extent of tumor invasion) stage, N (regional lymph node) stage, and M (distant metastasis) stage was collected. We excluded patients in*

which the information mentioned above was missing"			
		Dev	Val
4.1 Were there a reasonable number of participants with the outcome?		NI	PY
4.2 Were continuous and categorical predictors handled appropriately?		N	N
4.3 Were all enrolled participants included in the analysis?		PY	PY
4.4 Were participants with missing data handled appropriately?		N	N
4.5 Was selection of predictors based on univariable analysis avoided?		N	
4.6 Were complexities in the data (e.g. censoring, competing risks, sampling of controls) accounted for appropriately?		N	N
4.7 Were relevant model performance measures evaluated appropriately?		Y	Y
4.8 Were model overfitting and optimism in model performance accounted for?		Y	
4.9 Do predictors and their assigned weights in the final model correspond to the results from multivariable analysis?		PY	
**Risk of bias introduced by the analysis**	**RISK:** *(low/ high/ unclear)*	High	High
Rationale of bias rating: *They didn´t say the number of events. They did categorizations. They excluded patients with missing data. They selected the predictors based on univariable. They didn't use competing risks.*			

Step 4: Overall assessment

Use the following tables to reach overall judgements about risk of bias and concerns regarding applicability of the prediction model evaluation (development and/or validation) across all assessed domains.
Complete for each evaluation of a distinct model.

**Reaching an overall judgement about risk of bias of the prediction model evaluation**	
**Low risk of bias**	If all domains were rated low risk of bias.<br>If a prediction model was developed without any external validation, and it was rated as low risk of bias for all domains, consider downgrading to **high risk of bias**. Such a model can only be considered as low risk of bias, if the development was based on a very large data set and included some form of internal validation.
**High risk of bias**	If at least one domain is judged to be at **high risk of bias**.
**Unclear risk of bias**	If an unclear risk of bias was noted in at least one domain and it was low risk for all other domains.

**Reaching an overall judgement about applicability of the prediction model evaluation**	
**Low concerns regarding applicability**	If low concerns regarding applicability for all domains, the prediction model evaluation is judged to have **low concerns regarding applicability**.
**High concerns regarding applicability**	If high concerns regarding applicability for at least one domain, the prediction model evaluation is judged to have **high concerns regarding applicability**.
**Unclear concerns regarding applicability**	If unclear concerns (but no "high concern") regarding applicability for at least one domain, the prediction model evaluation is judged to have **unclear concerns regarding applicability** overall.

**Overall judgement about risk of bias and applicability of the prediction model evaluation**		
**Overall judgement of risk of bias**	**RISK:**<br>*(low/ high/ unclear)*	High
Summary of sources of potential bias:<br>Analysis domain shows several major issues		
**Overall judgement of applicability**	**CONCERN:**<br>*(low/ high/ unclear)*	Low
Summary of applicability concerns:<br><br>No major issues		

PROBAST

(Prediction model study Risk Of Bias Assessment Tool)

Published in Annals of Internal Medicine (freely available):

1. PROBAST: A Tool to Assess the Risk of Bias and Applicability of Prediction Model Studies
2. PROBAST: A Tool to Assess Risk of Bias and Applicability of Prediction Model Studies: Explanation and Elaboration

What does PROBAST assess?

PROBAST assesses both the *risk of bias* and *concerns regarding applicability* of a study that evaluates (develops, validates or updates) a multivariable diagnostic or prognostic prediction model. It is designed to assess primary studies included in a systematic review.

Bias occurs if systematic flaws or limitations in the design, conduct or analysis of a primary study distort the results. For the purpose of prediction modelling studies, we have defined *risk of bias* to occur when shortcomings in the study design, conduct or analysis lead to systematically distorted estimates of a model's predictive performance or to an inadequate model to address the research question. Model predictive performance is typically evaluated using calibration, discrimination and sometimes classification measures, and these are likely inaccurately estimated in studies with high risk of bias. *Applicability* refers to the extent to which the prediction model from the primary study matches your systematic review question, for example in terms of the participants, predictors or outcome of interest.

A primary study may include the development and/or validation or update of more than one prediction model. A PROBAST assessment should be completed for each distinct model that is developed, validated or updated (extended) for making individualised predictions. Where a publication assesses multiple prediction models, only complete a PROBAST assessment for those models that meet the inclusion criteria for your systematic review. Please note that subsequent use of the term "model" includes derivatives of models, such as simplified risk scores, nomograms, or recalibrations of models.

PROBAST is not designed for all multivariable diagnostic or prognostic studies. For example, studies using multivariable models to identify predictors associated with an outcome but not attempting to develop a model for making individualised predictions are not covered by PROBAST.

PROBAST includes four steps.

Step	Task	When to complete
**1**	Specify your systematic review question(s)	Once per systematic review
**2**	Classify the type of prediction model evaluation	Once for each model of interest in each publication being assessed, for each relevant outcome
**3**	Assess risk of bias and applicability	Once for each development and validation of each distinct prediction model in a publication
**4**	Overall judgment	Once for each development and validation of each distinct prediction model in a publication

If this is your first time using PROBAST, we strongly recommend reading the detailed explanation and elaboration (E&E, see link above) paper and to check the examples on www.probast.org

Step 1: Specify your systematic review question

State your systematic review question to facilitate the assessment of the applicability of the evaluated models to your question. *The following table should be completed once per systematic review.*

Criteria	Specify your systematic review question
Intended use of model:	*To predict cancer specific mortality in patients with renal cancer treated with partial or total nephrectomy regardless of TNM stage*
***Participants** including selection criteria and setting:*	*Patients with renal cancer treated with partial or total nephrectomy regardless of TNM stage*
***Predictors** (used in prediction modelling), including types of predictors (e.g. history, clinical examination, biochemical markers, imaging tests), time of measurement, specific measurement issues (e.g., any requirements/ prohibitions for specialized equipment):*	*Predictors used in clinical practice measured when a nephrectomy for renal cancer is indicated*
Outcome to be predicted:	*Cancer specific mortality*

Step 2: Classify the type of prediction model evaluation

Use the following table to classify the evaluation as model development, model validation or model update, or combination. Different signalling questions apply for different types of prediction model evaluation. If the evaluation does not fit one of these classifications then PROBAST should not be used.

Classify the evaluation based on its aim			
**Type of prediction study**	**PROBAST boxes to complete**	**Tick as appropriate**	**Definition for type of prediction model study**
Development only	Development	✗	Prediction model development without external validation. These studies may include internal validation methods, such as bootstrapping and cross-validation techniques.
Development and validation	Development and validation	✓	Prediction model development combined with external validation in other participants in the same article.
Validation only	Validation	✗	External validation of existing (previously developed) model in other participants.

This table should be completed once for each publication being assessed and for each relevant outcome in your review.

**Publication reference**	Zheng J, Li S, Zhao Y, et al. Nomograms for predicting overall and cancer-specific survival of patients with chromophobe renal cell carcinoma after nephrectomy: a retrospective SEER-based study. BMJ Open 2022;**12**:e062129. doi:10.1136/bmjopen-2022-062129
**Models of interest**	Nomogram
**Outcome of interest**	*Cancer specific mortality*

Step 3: Assess risk of bias and applicability

PROBAST is structured as four key domains. Each domain is judged for risk of bias (low, high or unclear) and includes signalling questions to help make judgements. Signalling questions are rated as yes (Y), probably yes (PY), probably no (PN), no (N) or no information (NI). All signalling questions are phrased so that "yes" indicates absence of bias. Any signalling question rated as "no" or "probably no" flags the potential for bias; you will need to use your judgement to determine whether the domain should be rated as "high", "low" or "unclear" risk of bias. The guidance document contains further instructions and examples on rating signalling questions and risk of bias for each domain.

The first three domains are also rated for concerns regarding applicability (low/ high/ unclear) to your review question defined above.

Complete all domains separately for each evaluation of a distinct model. Shaded boxes indicate where signalling questions do not apply and should not be answered.

**DOMAIN 1: Participants**			
**A. Risk of Bias**			
Describe the sources of data and criteria for participant selection:<br><br>*"In our study, we retrospectively collected chRCC patients after nephrectomy diagnosed between 2010 and 2015 from the SEER 18 database by using SEER*Stat software V.8.3.8, according to the International Classification of Diseases for Oncology third edition, primary site codes C64.9 and C65.9 (kidney and renal pelvis) and histological/behaviour codes 8270/0, 8270/1 and 8270/3 (chromophobe carcinoma). The inclusion criteria were as follows: (1) patients who underwent nephrectomy with explicit surgery method; (2) patients diagnosed at least 18 years old; (3) patients with active follow-up; (4) patients histologically diagnosed with the first malignant tumour; (5) patients for whom the cause of death was available; (6) patients with known clinical data and (7) survival time is at least 1 month"*			
		Dev	Val
1.1 Were appropriate data sources used, e.g. cohort, RCT or nested case-control study data?		Y	Y
1.2 Were all inclusions and exclusions of participants appropriate?		Y	Y
**Risk of bias introduced by selection of participants**	**RISK:**<br>*(low/ high/ unclear)*	Low	Low
Rationale of bias rating:<br>*No major issues identified*			
**B. Applicability**			
Describe included participants, setting and dates:<br>*"In our study, we retrospectively collected chRCC patients after nephrectomy diagnosed between 2010 and 2015 from the SEER 18 database by using SEER*Stat software V.8.3.8, according to the International Classification of Diseases for Oncology third edition, primary site codes C64.9 and C65.9 (kidney and renal pelvis) and histological/behaviour codes 8270/0, 8270/1 and 8270/3 (chromophobe carcinoma). The inclusion criteria were as follows: (1) patients who underwent nephrectomy with explicit surgery method; (2) patients diagnosed at least 18 years old; (3) patients with active follow-up; (4) patients histologically diagnosed with the first malignant tumour; (5) patients for whom the cause of death was available; (6) patients with known clinical data and (7) survival time is at least 1 month"*			
**Concern that the included participants and setting do not match the review question**	**CONCERN:**<br>*(low/ high/ unclear)*	Low	Low
Rationale of applicability rating:<br>*No major issues identified*			

DOMAIN 2: Predictors			
**A. Risk of Bias**			
List and describe predictors included in the final model, e.g. definition and timing of assessment:<br><br>The predictors included in the model were: age, TN stage, surgery and tumor size.<br><br>The predictors were measured before and after treatment			
		Dev	Val
2.1 Were predictors defined and assessed in a similar way for all participants?		PY	PY
2.2 Were predictor assessments made without knowledge of outcome data?		PY	PY
2.3 Are all predictors available at the time the model is intended to be used?		PY	PY
**Risk of bias introduced by predictors or their assessment**	**RISK:**<br>*(low/ high/ unclear)*	Low	Low
Rationale of bias rating:<br>*The blinding of measurement is unknown but all the predictors are objective.*			
**B. Applicability**			
Concern that the definition, assessment or timing of predictors in the model do not match the review question	**CONCERN:**<br>*(low/ high/ unclear)*	Low	Low
Rationale of applicability rating:<br>*No major issues identified.*			

DOMAIN 3: Outcome			
**A. Risk of Bias**			
Describe the outcome, how it was defined and determined, and the time interval between predictor assessment and outcome determination:<br>The outcome was cancer specific survival at 3 and 5 years			
		Dev	Val
3.1 Was the outcome determined appropriately?		PY	PY
3.2 Was a pre-specified or standard outcome definition used?		Y	Y
3.3 Were predictors excluded from the outcome definition?		PY	PY
3.4 Was the outcome defined and determined in a similar way for all participants?		PY	PY
3.5 Was the outcome determined without knowledge of predictor information?		PY	PY
3.6 Was the time interval between predictor assessment and outcome determination appropriate?		Y	Y
**Risk of bias introduced by the outcome or its determination**	**RISK:**<br>*(low/ high/ unclear)*	Low	Low
Rationale of bias rating:<br>*No major issues identified*			
**B. Applicability**			
At what time point was the outcome determined:<br>3 and 5 years<br><br>*If a composite outcome was used, describe the relative frequency/distribution of each contributing outcome:*<br>*N/A*			
**Concern that the outcome, its definition, timing or determination do not match the review question**	**CONCERN:**<br>*(low/ high/ unclear)*	Low	Low
Rationale of applicability rating:<br>*The outcome of the primary study matches the outcome of interest of the review*			

**DOMAIN 4: Analysis**
**Risk of Bias**
Describe numbers of participants, number of candidate predictors, outcome events and events per candidate predictor:<br>*"After screening by the above criteria, 2810 eligible patients with chRCC were included in the study. Subsequently, the patients were randomly divided into two cohorts, including the training cohort with 70% of patients (n=1970) and the validation cohort with 30% of patients (n=840). For the purpose of testing our results further, we validated them in another single-centre external validation cohort from China. There were 124 patients with chRCC after nephrectomy diagnosed between 1 September 2010 and 31 December 2020 who met the inclusion criteria above"*<br><br>*A total of 24 predictors were included (Table 1)*<br><br>*EPV develop the model = 37/24=1.54*<br>*EPV internal validation = 19/24=0.79*<br>*EPV external validation = 5/24=0.2*
Describe how the model was developed (for example in regards to modelling technique (e.g. survival or logistic modelling), predictor selection, and risk group definition):<br><br>*"Continuous variables in our study, including age and tumour size, were divided into three categories using X-tile software (V.3.6.1, Yale University of Medicine, USA), which is useful software to calculate the optimal cut-off points for continuous data. The least absolute shrinkage and selection operator (LASSO) regression was performed to screen the prognostic factors according the data from the training cohort. Variables screened by LASSO regression analyses were included in nomograms to predict 3-year and 5-year OS and CSS in patients with chRCC after nephrectomy"*
Describe whether and how the model was validated, either internally (e.g. bootstrapping, cross validation, random split sample) or externally (e.g. temporal validation, geographical validation, different setting, different type of participants):<br><br>*Not indicated*
Describe the performance measures of the model, e.g. (re)calibration, discrimination, (re)classification, net benefit, and whether they were adjusted for optimism:<br><br>*"Receiver operating characteristic (ROC) curves and the area under the curve (AUC) were used to evaluate the sensitivity and specificity of the two nomograms. In addition, concordance index (C-index) and calibration curves were performed to evaluate the discriminative and accuracy ability of the two nomograms"*
Describe any participants who were excluded from the analysis:<br>*"The inclusion criteria were as follows: (1) patients who underwent nephrectomy with explicit surgery method; (2) patients diagnosed at least 18 years old; (3) patients with active follow-up; (4) patients histologically diagnosed with the first malignant tumour; (5) patients for whom the cause of death was available; (6) patients with known clinical data and (7) survival time is at least 1 month"*
Describe missing data on predictors and outcomes as well as methods used for missing data:<br>*"The inclusion criteria were as follows: (1) patients who underwent nephrectomy with explicit surgery*

method; (2) patients diagnosed at least 18 years old; (3) patients with active follow-up; (4) patients histologically diagnosed with the first malignant tumour; (5) patients for whom the cause of death was available; (6) patients with known clinical data and (7) survival time is at least 1 month"			
		Dev	Val
4.1 Were there a reasonable number of participants with the outcome?		N	N
4.2 Were continuous and categorical predictors handled appropriately?		N	N
4.3 Were all enrolled participants included in the analysis?		PY	PY
4.4 Were participants with missing data handled appropriately?		N	N
4.5 Was selection of predictors based on univariable analysis avoided?		N	
4.6 Were complexities in the data (e.g. censoring, competing risks, sampling of controls) accounted for appropriately?		N	N
4.7 Were relevant model performance measures evaluated appropriately?		Y	Y
4.8 Were model overfitting and optimism in model performance accounted for?		N	
4.9 Do predictors and their assigned weights in the final model correspond to the results from multivariable analysis?		PY	
**Risk of bias introduced by the analysis**	**RISK:** *(low/ high/ unclear)*	High	High
Rationale of bias rating: *They did categorizations. They excluded patients with missing data. They selected the predictors based on univariable. They didn't use competing risks. They didn´t do bootstrapping*			

Step 4: Overall assessment

Use the following tables to reach overall judgements about risk of bias and concerns regarding applicability of the prediction model evaluation (development and/or validation) across all assessed domains.

Complete for each evaluation of a distinct model.

Reaching an overall judgement about risk of bias of the prediction model evaluation	
**Low risk of bias**	If all domains were rated low risk of bias.<br>If a prediction model was developed without any external validation, and it was rated as low risk of bias for all domains, consider downgrading to **high risk of bias**. Such a model can only be considered as low risk of bias, if the development was based on a very large data set and included some form of internal validation.
**High risk of bias**	If at least one domain is judged to be at **high risk of bias**.
**Unclear risk of bias**	If an unclear risk of bias was noted in at least one domain and it was low risk for all other domains.

Reaching an overall judgement about applicability of the prediction model evaluation	
**Low concerns regarding applicability**	If low concerns regarding applicability for all domains, the prediction model evaluation is judged to have **low concerns regarding applicability**.
**High concerns regarding applicability**	If high concerns regarding applicability for at least one domain, the prediction model evaluation is judged to have **high concerns regarding applicability**.
**Unclear concerns regarding applicability**	If unclear concerns (but no "high concern") regarding applicability for at least one domain, the prediction model evaluation is judged to have **unclear concerns regarding applicability** overall.

Overall judgement about risk of bias and applicability of the prediction model evaluation		
**Overall judgement of risk of bias**	**RISK:**<br>*(low/ high/ unclear)*	High
Summary of sources of potential bias:<br>Analysis domain shows several major issues		
**Overall judgement of applicability**	**CONCERN:**<br>*(low/ high/ unclear)*	Low
Summary of applicability concerns:<br><br>No major issues		

PROBAST

(Prediction model study Risk Of Bias Assessment Tool)

Published in Annals of Internal Medicine (freely available):

1. PROBAST: A Tool to Assess the Risk of Bias and Applicability of Prediction Model Studies
2. PROBAST: A Tool to Assess Risk of Bias and Applicability of Prediction Model Studies: Explanation and Elaboration

What does PROBAST assess?

PROBAST assesses both the *risk of bias* and *concerns regarding applicability* of a study that evaluates (develops, validates or updates) a multivariable diagnostic or prognostic prediction model. It is designed to assess primary studies included in a systematic review.

Bias occurs if systematic flaws or limitations in the design, conduct or analysis of a primary study distort the results. For the purpose of prediction modelling studies, we have defined *risk of bias* to occur when shortcomings in the study design, conduct or analysis lead to systematically distorted estimates of a model's predictive performance or to an inadequate model to address the research question. Model predictive performance is typically evaluated using calibration, discrimination and sometimes classification measures, and these are likely inaccurately estimated in studies with high risk of bias. *Applicability* refers to the extent to which the prediction model from the primary study matches your systematic review question, for example in terms of the participants, predictors or outcome of interest.

A primary study may include the development and/or validation or update of more than one prediction model. A PROBAST assessment should be completed for each distinct model that is developed, validated or updated (extended) for making individualised predictions. Where a publication assesses multiple prediction models, only complete a PROBAST assessment for those models that meet the inclusion criteria for your systematic review. Please note that subsequent use of the term "model" includes derivatives of models, such as simplified risk scores, nomograms, or recalibrations of models.

PROBAST is not designed for all multivariable diagnostic or prognostic studies. For example, studies using multivariable models to identify predictors associated with an outcome but not attempting to develop a model for making individualised predictions are not covered by PROBAST.

PROBAST includes four steps.

Step	Task	When to complete
**1**	Specify your systematic review question(s)	Once per systematic review
**2**	Classify the type of prediction model evaluation	Once for each model of interest in each publication being assessed, for each relevant outcome
**3**	Assess risk of bias and applicability	Once for each development and validation of each distinct prediction model in a publication
**4**	Overall judgment	Once for each development and validation of each distinct prediction model in a publication

If this is your first time using PROBAST, we strongly recommend reading the detailed explanation and elaboration (E&E, see link above) paper and to check the examples on www.probast.org

Step 1: Specify your systematic review question

State your systematic review question to facilitate the assessment of the applicability of the evaluated models to your question. *The following table should be completed once per systematic review.*

Criteria	Specify your systematic review question
Intended use of model:	*To predict cancer specific mortality in patients with renal cancer treated with partial or total nephrectomy regardless of TNM stage*
***Participants** including selection criteria and setting:*	*Patients with renal cancer treated with partial or total nephrectomy regardless of TNM stage*
***Predictors** (used in prediction modelling), including types of predictors (e.g. history, clinical examination, biochemical markers, imaging tests), time of measurement, specific measurement issues (e.g., any requirements/ prohibitions for specialized equipment):*	*Predictors used in clinical practice measured when a nephrectomy for renal cancer is indicated*
Outcome to be predicted:	*Cancer specific mortality*

Step 2: Classify the type of prediction model evaluation

Use the following table to classify the evaluation as model development, model validation or model update, or combination. Different signalling questions apply for different types of prediction model evaluation. If the evaluation does not fit one of these classifications then PROBAST should not be used.

**Classify the evaluation based on its aim**			
**Type of prediction study**	**PROBAST boxes to complete**	**Tick as appropriate**	**Definition for type of prediction model study**
Development only	Development	✗	Prediction model development without external validation. These studies may include internal validation methods, such as bootstrapping and cross-validation techniques.
Development and validation	Development and validation	✓	Prediction model development combined with external validation in other participants in the same article.
Validation only	Validation	✗	External validation of existing (previously developed) model in other participants.

This table should be completed once for each publication being assessed and for each relevant outcome in your review.

**Publication reference**	Laukhtina E, Schuettfort VM, D'Andrea D, Pradere B, Quhal F, Mori K, Sari Motlagh R, Mostafaei H, Katayama S, Grossmann NC, Rajwa P, Karakiewicz PI, Schmidinger M, Fajkovic H, Enikeev D, Shariat SF. Selection and evaluation of preoperative systemic inflammatory response biomarkers model prior to cytoreductive nephrectomy using a machine-learning approach. World J Urol. 2022 Mar;40(3):747-754. doi: 10.1007/s00345-021-03844-w. Epub 2021 Oct 20. PMID: 34671856; PMCID: PMC8948147.
**Models of interest**	Nomogram
**Outcome of interest**	*Cancer specific mortality*

Step 3: Assess risk of bias and applicability

PROBAST is structured as four key domains. Each domain is judged for risk of bias (low, high or unclear) and includes signalling questions to help make judgements. Signalling questions are rated as yes (Y), probably yes (PY), probably no (PN), no (N) or no information (NI). All signalling questions are phrased so that “yes” indicates absence of bias. Any signalling question rated as “no” or “probably no” flags the potential for bias; you will need to use your judgement to determine whether the domain should be rated as “high”, “low” or “unclear” risk of bias. The guidance document contains further instructions and examples on rating signalling questions and risk of bias for each domain.

The first three domains are also rated for concerns regarding applicability (low/ high/ unclear) to your review question defined above.

Complete all domains separately for each evaluation of a distinct model. Shaded boxes indicate where signalling questions do not apply and should not be answered.

<table>
<tr><td colspan="4">DOMAIN 1: Participants</td></tr>
<tr><td colspan="4">A. Risk of Bias</td></tr>
<tr><td colspan="4">Describe the sources of data and criteria for participant selection:
“We retrospectively reviewed our established international multicenter database to identify mRCC patients treated with CN at tertiary centers in the USA and Europe. We excluded patients with other malignant primary tumors. However, concomitant hematologic or liver diseases, chronic inflammatory disease including autoimmune disorder and infection within the last 12 months were not excluded”</td></tr>
<tr><td colspan="2"></td><td>Dev</td><td>Val</td></tr>
<tr><td colspan="2">1.1 Were appropriate data sources used, e.g. cohort, RCT or nested case-control study data?</td><td>Y</td><td>Y</td></tr>
<tr><td colspan="2">1.2 Were all inclusions and exclusions of participants appropriate?</td><td>N</td><td>N</td></tr>
<tr><td>Risk of bias introduced by selection of participants</td><td>RISK:
(low/ high/ unclear)</td><td>High</td><td>High</td></tr>
<tr><td colspan="4">Rationale of bias rating:
Cohort study not included patients with another primary tumors</td></tr>
<tr><td colspan="4">B. Applicability</td></tr>
<tr><td colspan="4">Describe included participants, setting and dates:
“We retrospectively reviewed our established international multicenter database to identify mRCC patients treated with CN at tertiary centers in the USA and Europe. We excluded patients with other malignant primary tumors. However, concomitant hematologic or liver diseases, chronic inflammatory disease including autoimmune disorder and infection within the last 12 months were not excluded”</td></tr>
<tr><td>Concern that the included participants and setting do not match the review question</td><td>CONCERN:
(low/ high/ unclear)</td><td>High</td><td>High</td></tr>
<tr><td colspan="4">Rationale of applicability rating:
Cohort study not included patients with another primary tumors</td></tr>
</table>

**DOMAIN 2: Predictors**			
**A. Risk of Bias**			
List and describe predictors included in the final model, e.g. definition and timing of assessment:<br><br>The predictors included in the model were: AGR, SII, TheRitis, Abnormal hemoglobine, lymph node involment, multiples metastatic sites, clear cell carcinoma histology (Figure 1)<br><br>All the predictors were measured before and after treatment.<br>All laboratory tests were done within 1 month prior to the CN.			
		Dev	Val
2.1 Were predictors defined and assessed in a similar way for all participants?		PY	PY
2.2 Were predictor assessments made without knowledge of outcome data?		PY	PY
2.3 Are all predictors available at the time the model is intended to be used?		PY	PY
**Risk of bias introduced by predictors or their assessment**	**RISK:**<br>*(low/ high/ unclear)*	Low	Low
Rationale of bias rating:<br>*The blinding of measurement is unknown but all the predictors are objective.*			
**B. Applicability**			
Concern that the definition, assessment or timing of predictors in the model do not match the review question	**CONCERN:**<br>*(low/ high/ unclear)*	Low	Low
Rationale of applicability rating:<br>*No major issues identified.*			

DOMAIN 3: Outcome			
**A. Risk of Bias**			
Describe the outcome, how it was defined and determined, and the time interval between predictor assessment and outcome determination:<br>The outcome was cancer specific survival 2 years			
		Dev	Val
3.1 Was the outcome determined appropriately?		PY	PY
3.2 Was a pre-specified or standard outcome definition used?		Y	Y
3.3 Were predictors excluded from the outcome definition?		PY	PY
3.4 Was the outcome defined and determined in a similar way for all participants?		PY	PY
3.5 Was the outcome determined without knowledge of predictor information?		PY	PY
3.6 Was the time interval between predictor assessment and outcome determination appropriate?		Y	Y
**Risk of bias introduced by the outcome or its determination**	**RISK:**<br>*(low/ high/ unclear)*	Low	Low
Rationale of bias rating:<br>*No major issues identified*			
**B. Applicability**			
At what time point was the outcome determined:<br>2 years<br><br>*If a composite outcome was used, describe the relative frequency/distribution of each contributing outcome:*<br>*N/A*			
**Concern that the outcome, its definition, timing or determination do not match the review question**	**CONCERN:**<br>*(low/ high/ unclear)*	Low	Low
Rationale of applicability rating:<br>*The outcome of the primary study matches the outcome of interest of the review*			

DOMAIN 4: Analysis		
**Risk of Bias**		
Describe numbers of participants, number of candidate predictors, outcome events and events per candidate predictor:<br><br>*"A total of 472 (77%) patients died, and 99% of deaths were due to mRCC"*<br>*They didn´t say how many in the development or validation cohort occurred*<br><br>*A total of 8 predictors were included (supplementary table 2)*<br><br>*N= 400 (E= unknown) to develop the model and 213 to validate it (E= unknown)*<br>*EPV= unknown*		
Describe how the model was developed (for example in regards to modelling technique (e.g. survival or logistic modelling), predictor selection, and risk group definition):<br><br>*"The absolute shrinkage and selection operator (LASSO) approach and tenfold cross-validation were used for fitting of the most informative, yet parsimonious multivariable model with respect to prediction/prognosis of CSS"*<br>*"The selected variables were then used to fit the multivariable Cox model"*		
Describe whether and how the model was validated, either internally (e.g. bootstrapping, cross validation, random split sample) or externally (e.g. temporal validation, geographical validation, different setting, different type of participants):<br><br>*"Validation was performed using 200 bootstrap re-samples as a means of calculating the most unbiased predictive accuracy"*		
Describe the performance measures of the model, e.g. (re)calibration, discrimination, (re)classification, net benefit, and whether they were adjusted for optimism:<br>*"The discrimination ability of this model was assessed by calculating the C-index (Harrell's concord- ance index, an approximation of the AUC in censored data) for both the training and the testing cohorts. To assess the additional discriminatory power of the biomarkers, a refer- ence model was fitted that did not include the previously selected SIR-biomarkers. Calibration plots graphically explored the association between predicted probabilities and the observed proportions"*<br><br>*"Finally, the decision curve analysis (DCA) was used to evaluate the clinical net benefit of the model for both the training and testing cohorts"*		
Describe any participants who were excluded from the analysis:<br>*"We excluded patients with other malignant primary tumors. However, concomitant hematologic or liver diseases, chronic inflammatory disease including autoimmune disorder and infection within the last 12 months were not excluded"*		
Describe missing data on predictors and outcomes as well as methods used for missing data:<br>*Not indicated*		
	Dev	Val
4.1 Were there a reasonable number of participants with the outcome?	NI	PY

4.2 Were continuous and categorical predictors handled appropriately?			N	N
4.3 Were all enrolled participants included in the analysis?			Y	Y
4.4 Were participants with missing data handled appropriately?			NI	NI
4.5 Was selection of predictors based on univariable analysis avoided?			Y	
4.6 Were complexities in the data (e.g. censoring, competing risks, sampling of controls) accounted for appropriately?			N	N
4.7 Were relevant model performance measures evaluated appropriately?			Y	Y
4.8 Were model overfitting and optimism in model performance accounted for?			Y	
4.9 Do predictors and their assigned weights in the final model correspond to the results from multivariable analysis?			PY	
**Risk of bias introduced by the analysis**		**RISK:** *(low/ high/ unclear)*	High	High
Rationale of bias rating: *They didn´t say the number of events in each cohort. They did categorisation. Not information about missing data. They didn't use competing risks.*				

Step 4: Overall assessment

Use the following tables to reach overall judgements about risk of bias and concerns regarding applicability of the prediction model evaluation (development and/or validation) across all assessed domains.
Complete for each evaluation of a distinct model.

Reaching an overall judgement about risk of bias of the prediction model evaluation	
**Low risk of bias**	If all domains were rated low risk of bias.<br>If a prediction model was developed without any external validation, and it was rated as low risk of bias for all domains, consider downgrading to **high risk of bias**. Such a model can only be considered as low risk of bias, if the development was based on a very large data set and included some form of internal validation.
**High risk of bias**	If at least one domain is judged to be at **high risk of bias**.
**Unclear risk of bias**	If an unclear risk of bias was noted in at least one domain and it was low risk for all other domains.

Reaching an overall judgement about applicability of the prediction model evaluation	
**Low concerns regarding applicability**	If low concerns regarding applicability for all domains, the prediction model evaluation is judged to have **low concerns regarding applicability**.
**High concerns regarding applicability**	If high concerns regarding applicability for at least one domain, the prediction model evaluation is judged to have **high concerns regarding applicability**.
**Unclear concerns regarding applicability**	If unclear concerns (but no "high concern") regarding applicability for at least one domain, the prediction model evaluation is judged to have **unclear concerns regarding applicability** overall.

Overall judgement about risk of bias and applicability of the prediction model evaluation		
**Overall judgement of risk of bias**	**RISK:**<br>*(low/ high/ unclear)*	High
Summary of sources of potential bias:<br>Analysis and participants domains show several major issues		
**Overall judgement of applicability**	**CONCERN:**<br>*(low/ high/ unclear)*	High
Summary of applicability concerns:<br><br>Participants domain shows several major issues		

PROBAST

(Prediction model study Risk Of Bias Assessment Tool)

Published in Annals of Internal Medicine (freely available):

1. PROBAST: A Tool to Assess the Risk of Bias and Applicability of Prediction Model Studies
2. PROBAST: A Tool to Assess Risk of Bias and Applicability of Prediction Model Studies: Explanation and Elaboration

What does PROBAST assess?

PROBAST assesses both the *risk of bias* and *concerns regarding applicability* of a study that evaluates (develops, validates or updates) a multivariable diagnostic or prognostic prediction model. It is designed to assess primary studies included in a systematic review.

Bias occurs if systematic flaws or limitations in the design, conduct or analysis of a primary study distort the results. For the purpose of prediction modelling studies, we have defined *risk of bias* to occur when shortcomings in the study design, conduct or analysis lead to systematically distorted estimates of a model's predictive performance or to an inadequate model to address the research question. Model predictive performance is typically evaluated using calibration, discrimination and sometimes classification measures, and these are likely inaccurately estimated in studies with high risk of bias. *Applicability* refers to the extent to which the prediction model from the primary study matches your systematic review question, for example in terms of the participants, predictors or outcome of interest.

A primary study may include the development and/or validation or update of more than one prediction model. A PROBAST assessment should be completed for each distinct model that is developed, validated or updated (extended) for making individualised predictions. Where a publication assesses multiple prediction models, only complete a PROBAST assessment for those models that meet the inclusion criteria for your systematic review. Please note that subsequent use of the term "model" includes derivatives of models, such as simplified risk scores, nomograms, or recalibrations of models.

PROBAST is not designed for all multivariable diagnostic or prognostic studies. For example, studies using multivariable models to identify predictors associated with an outcome but not attempting to develop a model for making individualised predictions are not covered by PROBAST.

PROBAST includes four steps.

Step	Task	When to complete
**1**	Specify your systematic review question(s)	Once per systematic review
**2**	Classify the type of prediction model evaluation	Once for each model of interest in each publication being assessed, for each relevant outcome
**3**	Assess risk of bias and applicability	Once for each development and validation of each distinct prediction model in a publication
**4**	Overall judgment	Once for each development and validation of each distinct prediction model in a publication

If this is your first time using PROBAST, we strongly recommend reading the detailed explanation and elaboration (E&E, see link above) paper and to check the examples on www.probast.org

Step 1: Specify your systematic review question

State your systematic review question to facilitate the assessment of the applicability of the evaluated models to your question. *The following table should be completed once per systematic review.*

Criteria	Specify your systematic review question
Intended use of model:	*To predict cancer specific mortality in patients with renal cancer treated with partial or total nephrectomy regardless of TNM stage*
***Participants** including selection criteria and setting:*	*Patients with renal cancer treated with partial or total nephrectomy regardless of TNM stage*
***Predictors** (used in prediction modelling), including types of predictors (e.g. history, clinical examination, biochemical markers, imaging tests), time of measurement, specific measurement issues (e.g., any requirements/ prohibitions for specialized equipment):*	*Predictors used in clinical practice measured when a nephrectomy for renal cancer is indicated*
Outcome to be predicted:	*Cancer specific mortality*

Step 2: Classify the type of prediction model evaluation

Use the following table to classify the evaluation as model development, model validation or model update, or combination. Different signalling questions apply for different types of prediction model evaluation. If the evaluation does not fit one of these classifications then PROBAST should not be used.

**Classify the evaluation based on its aim**			
**Type of prediction study**	**PROBAST boxes to complete**	**Tick as appropriate**	**Definition for type of prediction model study**
Development only	Development	✗	Prediction model development without external validation. These studies may include internal validation methods, such as bootstrapping and cross-validation techniques.
Development and validation	Development and validation	✓	Prediction model development combined with external validation in other participants in the same article.
Validation only	Validation	✗	External validation of existing (previously developed) model in other participants.

This table should be completed once for each publication being assessed and for each relevant outcome in your review.

**Publication reference**	Lu Z, He W, Zhou J, Yang C, Xiang R. Construction and validation of a novel prognostic nomogram for patients with metastatic renal cell carcinoma: a SEER-based study. J Int Med Res. 2022; 50(6): 03000605221105367.
**Models of interest**	Nomogram
**Outcome of interest**	*Cancer specific mortality*

Step 3: Assess risk of bias and applicability

PROBAST is structured as four key domains. Each domain is judged for risk of bias (low, high or unclear) and includes signalling questions to help make judgements. Signalling questions are rated as yes (Y), probably yes (PY), probably no (PN), no (N) or no information (NI). All signalling questions are phrased so that "yes" indicates absence of bias. Any signalling question rated as "no" or "probably no" flags the potential for bias; you will need to use your judgement to determine whether the domain should be rated as "high", "low" or "unclear" risk of bias. The guidance document contains further instructions and examples on rating signalling questions and risk of bias for each domain.

The first three domains are also rated for concerns regarding applicability (low/ high/ unclear) to your review question defined above.

Complete all domains separately for each evaluation of a distinct model. Shaded boxes indicate where signalling questions do not apply and should not be answered.

**DOMAIN 1: Participants**			
**A. Risk of Bias**			
Describe the sources of data and criteria for participant selection:<br><br>*"Specific clinical parameters and prognostic outcomes of patients with MRCC from 2010 to 2015 were collected from the SEER database using reference number 14622-Nov2017"*<br>*"We excluded the following patients: unknown histological type (n= 89), unknown treatment (n= 6), unknown race (n= 4), bilateral renal tumor (n= 3), or tumor size >180 mm (n= 73)"*			
		Dev	Val
1.1 Were appropriate data sources used, e.g. cohort, RCT or nested case-control study data?		Y	Y
1.2 Were all inclusions and exclusions of participants appropriate?		N	N
**Risk of bias introduced by selection of participants**	**RISK:**<br>*(low/ high/ unclear)*	High	High
Rationale of bias rating:<br>*They excluded patients with large and bilateral tumors*			
**B. Applicability**			
Describe included participants, setting and dates:<br>*"Specific clinical parameters and prognostic outcomes of patients with MRCC from 2010 to 2015 were collected from the SEER database using reference number 14622-Nov2017"*<br>*"We excluded the following patients: unknown histological type (n= 89), unknown treatment (n= 6), unknown race (n= 4), bilateral renal tumor (n= 3), or tumor size >180 mm (n= 73)"*			
**Concern that the included participants and setting do not match the review question**	**CONCERN:**<br>*(low/ high/ unclear)*	High	High
Rationale of applicability rating:<br>*They excluded patients with large and bilateral tumors*			

DOMAIN 2: Predictors			
**A. Risk of Bias**			
List and describe predictors included in the final model, e.g. definition and timing of assessment:<br><br>The predictors included in the model were: patology, TN stage, surgery, age, rural and brain, liver, bone, lung or other metastasis<br>The predictors were measured before and after treatment			
		Dev	Val
2.1 Were predictors defined and assessed in a similar way for all participants?		PY	PY
2.2 Were predictor assessments made without knowledge of outcome data?		PY	PY
2.3 Are all predictors available at the time the model is intended to be used?		PY	PY
**Risk of bias introduced by predictors or their assessment**	**RISK:**<br>*(low/ high/ unclear)*	Low	Low
Rationale of bias rating:<br>The blinding of measurement is unknown but all the predictors are objectives			
**B. Applicability**			
Concern that the definition, assessment or timing of predictors in the model do not match the review question	**CONCERN:**<br>*(low/ high/ unclear)*	Low	Low
Rationale of applicability rating:<br>*No major issues identified.*			

**DOMAIN 3: Outcome**			
**A. Risk of Bias**			
Describe the outcome, how it was defined and determined, and the time interval between predictor assessment and outcome determination:<br><br>The outcome was cancer specific survival at 1, 2 and 3 years			
		Dev	Val
3.1 Was the outcome determined appropriately?		Y	Y
3.2 Was a pre-specified or standard outcome definition used?		Y	Y
3.3 Were predictors excluded from the outcome definition?		PY	PY
3.4 Was the outcome defined and determined in a similar way for all participants?		PY	PY
3.5 Was the outcome determined without knowledge of predictor information?		PY	PY
3.6 Was the time interval between predictor assessment and outcome determination appropriate?		Y	Y
**Risk of bias introduced by the outcome or its determination**	**RISK:**<br>*(low/ high/ unclear)*	Low	Low
Rationale of bias rating:<br>*No major issues identified*			
**B. Applicability**			
At what time point was the outcome determined:<br>1, 2 and 3 years<br><br>*If a composite outcome was used, describe the relative frequency/distribution of each contributing outcome:*<br>*N/A*			
**Concern that the outcome, its definition, timing or determination do not match the review question**	**CONCERN:**<br>*(low/ high/ unclear)*	Low	Low
Rationale of applicability rating:<br>*The outcome of the primary study matches the outcome of interest of the review*			

**DOMAIN 4: Analysis**		
**Risk of Bias**		
Describe numbers of participants, number of candidate predictors, outcome events and events per candidate predictor:<br><br>*"A total of 1201 eligible patients were selected as the training cohort (n= 601) and validation group (n= 600)"*<br><br>*A total of 31 predictors were included (Table 3)*<br>*The number of events is unknown*<br><br>*EPV= unknown*		
Describe how the model was developed (for example in regards to modelling technique (e.g. survival or logistic modelling), predictor selection, and risk group definition):<br><br>*"The independent prognostic factors of OS and CSS were determined by univariate and multivariate Cox analyses of the training cohort. Variables in the univariate Cox regression analysis with P<0.1 were included in the multivariate Cox regression analysis. On the basis of the outcomes of multivariate Cox regression analysis in the training cohort, we constructed two nomograms of OS and CSS using the "rms" and "survival" packages in R (www.r-project.org)"*		
Describe whether and how the model was validated, either internally (e.g. bootstrapping, cross validation, random split sample) or externally (e.g. temporal validation, geographical validation, different setting, different type of participants):<br><br>*"Bootstrapping with 1000 resamples was performed for the C-index and receiver operating characteristic curve evaluations"*		
Describe the performance measures of the model, e.g. (re)calibration, discrimination, (re)classification, net benefit, and whether they were adjusted for optimism:<br><br>*"The concordance index (C-index) was determined to evaluate discriminative ability. The area under the time-dependent receiver operating characteristic curve (time-dependent AUC) was applied to determine the sensitivity and specificity of nomograms"*<br><br>*"Furthermore, decision curve analysis (DCA) was used to estimate the clinical benefit of alternative models by quantifying net benefits at various threshold probabilities and assess the use of two nomograms compared with the AJCC staging system in this study"*		
Describe any participants who were excluded from the analysis:<br>*"We excluded the following patients: unknown histological type (n= 89), unknown treatment (n= 6), unknown race (n= 4), bilateral renal tumor (n= 3), or tumor size >180 mm (n= 73)"*		
Describe missing data on predictors and outcomes as well as methods used for missing data:<br>*"We excluded the following patients: unknown histological type (n= 89), unknown treatment (n= 6), unknown race (n= 4), bilateral renal tumor (n= 3), or tumor size >180 mm (n= 73)"*		
	Dev	Val
4.1 Were there a reasonable number of participants with the outcome?	NI	PY
4.2 Were continuous and categorical predictors handled appropriately?	N	N
4.3 Were all enrolled participants included in the analysis?	Y	Y
4.4 Were participants with missing data handled appropriately?	N	N

4.5 Was selection of predictors based on univariable analysis avoided?		N	
4.6 Were complexities in the data (e.g. censoring, competing risks, sampling of controls) accounted for appropriately?		N	N
4.7 Were relevant model performance measures evaluated appropriately?		Y	Y
4.8 Were model overfitting and optimism in model performance accounted for?		Y	
4.9 Do predictors and their assigned weights in the final model correspond to the results from multivariable analysis?		PY	
**Risk of bias introduced by the analysis**	**RISK:** *(low/ high/ unclear)*	High	High
Rationale of bias rating: *They didn´t say the number of events in each cohort, but according the number of total events probably the are enough events per variable on the validation cohort. They excluded patients with missing data. They selected the predictors based on univariable. They didn't use competing risks. They did categorization*			

Step 4: Overall assessment

Use the following tables to reach overall judgements about risk of bias and concerns regarding applicability of the prediction model evaluation (development and/or validation) across all assessed domains.
Complete for each evaluation of a distinct model.

**Reaching an overall judgement about risk of bias of the prediction model evaluation**	
**Low risk of bias**	If all domains were rated low risk of bias.<br>If a prediction model was developed without any external validation, and it was rated as low risk of bias for all domains, consider downgrading to **high risk of bias**. Such a model can only be considered as low risk of bias, if the development was based on a very large data set and included some form of internal validation.
**High risk of bias**	If at least one domain is judged to be at **high risk of bias**.
**Unclear risk of bias**	If an unclear risk of bias was noted in at least one domain and it was low risk for all other domains.

**Reaching an overall judgement about applicability of the prediction model evaluation**	
**Low concerns regarding applicability**	If low concerns regarding applicability for all domains, the prediction model evaluation is judged to have **low concerns regarding applicability**.
**High concerns regarding applicability**	If high concerns regarding applicability for at least one domain, the prediction model evaluation is judged to have **high concerns regarding applicability**.
**Unclear concerns regarding applicability**	If unclear concerns (but no "high concern") regarding applicability for at least one domain, the prediction model evaluation is judged to have **unclear concerns regarding applicability** overall.

**Overall judgement about risk of bias and applicability of the prediction model evaluation**		
**Overall judgement of risk of bias**	**RISK:**<br>*(low/ high/ unclear)*	High
Summary of sources of potential bias:<br>Analysis and participants domain show several major issues		
**Overall judgement of applicability**	**CONCERN:**<br>*(low/ high/ unclear)*	High
Summary of applicability concerns:<br>Participant domain shows several major issues		

PROBAST

(Prediction model study Risk Of Bias Assessment Tool)

Published in Annals of Internal Medicine (freely available):

1. PROBAST: A Tool to Assess the Risk of Bias and Applicability of Prediction Model Studies
2. PROBAST: A Tool to Assess Risk of Bias and Applicability of Prediction Model Studies: Explanation and Elaboration

What does PROBAST assess?

PROBAST assesses both the *risk of bias* and *concerns regarding applicability* of a study that evaluates (develops, validates or updates) a multivariable diagnostic or prognostic prediction model. It is designed to assess primary studies included in a systematic review.

Bias occurs if systematic flaws or limitations in the design, conduct or analysis of a primary study distort the results. For the purpose of prediction modelling studies, we have defined *risk of bias* to occur when shortcomings in the study design, conduct or analysis lead to systematically distorted estimates of a model's predictive performance or to an inadequate model to address the research question. Model predictive performance is typically evaluated using calibration, discrimination and sometimes classification measures, and these are likely inaccurately estimated in studies with high risk of bias. *Applicability* refers to the extent to which the prediction model from the primary study matches your systematic review question, for example in terms of the participants, predictors or outcome of interest.

A primary study may include the development and/or validation or update of more than one prediction model. A PROBAST assessment should be completed for each distinct model that is developed, validated or updated (extended) for making individualised predictions. Where a publication assesses multiple prediction models, only complete a PROBAST assessment for those models that meet the inclusion criteria for your systematic review. Please note that subsequent use of the term "model" includes derivatives of models, such as simplified risk scores, nomograms, or recalibrations of models.

PROBAST is not designed for all multivariable diagnostic or prognostic studies. For example, studies using multivariable models to identify predictors associated with an outcome but not attempting to develop a model for making individualised predictions are not covered by PROBAST.

PROBAST includes four steps.

Step	Task	When to complete
**1**	Specify your systematic review question(s)	Once per systematic review
**2**	Classify the type of prediction model evaluation	Once for each model of interest in each publication being assessed, for each relevant outcome
**3**	Assess risk of bias and applicability	Once for each development and validation of each distinct prediction model in a publication
**4**	Overall judgment	Once for each development and validation of each distinct prediction model in a publication

If this is your first time using PROBAST, we strongly recommend reading the detailed explanation and elaboration (E&E, see link above) paper and to check the examples on www.probast.org

Step 1: Specify your systematic review question

State your systematic review question to facilitate the assessment of the applicability of the evaluated models to your question. *The following table should be completed once per systematic review.*

Criteria	Specify your systematic review question
Intended use of model:	*To predict cancer specific mortality in patients with renal cancer treated with partial or total nephrectomy regardless of TNM stage*
***Participants** including selection criteria and setting:*	*Patients with renal cancer treated with partial or total nephrectomy regardless of TNM stage*
***Predictors** (used in prediction modelling), including types of predictors (e.g. history, clinical examination, biochemical markers, imaging tests), time of measurement, specific measurement issues (e.g., any requirements/ prohibitions for specialized equipment):*	*Predictors used in clinical practice measured when a nephrectomy for renal cancer is indicated*
Outcome to be predicted:	*Cancer specific mortality*

Step 2: Classify the type of prediction model evaluation

Use the following table to classify the evaluation as model development, model validation or model update, or combination. Different signalling questions apply for different types of prediction model evaluation. If the evaluation does not fit one of these classifications then PROBAST should not be used.

**Classify the evaluation based on its aim**			
**Type of prediction study**	**PROBAST boxes to complete**	**Tick as appropriate**	**Definition for type of prediction model study**
Development only	Development	✓	Prediction model development without external validation. These studies may include internal validation methods, such as bootstrapping and cross-validation techniques.
Development and validation	Development and validation	✗	Prediction model development combined with external validation in other participants in the same article.
Validation only	Validation	✗	External validation of existing (previously developed) model in other participants.

This table should be completed once for each publication being assessed and for each relevant outcome in your review.

**Publication reference**	Ni J, Wang Y, Zhang H, Wang K, Song W, Luo M, Che J, Geng J, Xu Y, Yao X, Zheng J, Chen M, Peng B, Mao W. Combination of preoperative plasma fibrinogen and neutrophil-to-lymphocyte ratio to predict the prognosis for patients undergoing laparoscopic nephrectomy for renal cell carcinoma. Am J Cancer Res 2022;12(8):3713-3728
**Models of interest**	Nomogram
**Outcome of interest**	*Cancer specific mortality*

Step 3: Assess risk of bias and applicability

PROBAST is structured as four key domains. Each domain is judged for risk of bias (low, high or unclear) and includes signalling questions to help make judgements. Signalling questions are rated as yes (Y), probably yes (PY), probably no (PN), no (N) or no information (NI). All signalling questions are phrased so that "yes" indicates absence of bias. Any signalling question rated as "no" or "probably no" flags the potential for bias; you will need to use your judgement to determine whether the domain should be rated as "high", "low" or "unclear" risk of bias. The guidance document contains further instructions and examples on rating signalling questions and risk of bias for each domain.

The first three domains are also rated for concerns regarding applicability (low/ high/ unclear) to your review question defined above.

Complete all domains separately for each evaluation of a distinct model. Shaded boxes indicate where signalling questions do not apply and should not be answered.

<table>
<tr><td colspan="4">DOMAIN 1: Participants</td></tr>
<tr><td colspan="4">A. Risk of Bias</td></tr>
<tr><td colspan="4">Describe the sources of data and criteria for participant selection:

“The retrospective study included 590 patients with RCC who underwent laparoscopic nephrectomy at Zhongda Hospital Southeast University, Shanghai Tenth People’s Hospital and Shidong Hospital between January 2014 and December 2019. This study complied with the criteria outlined in the Declaration of Helsinki (as revised in 2013) and was approved by the Ethics Committee and Institutional Review Board of all participating institutions (SHSY-IECKY-4.0/18- 68/01 and ZDKYSB077). Signed informed consent was provided by all patients and their relatives who enrolled in this study.”
“The patient inclusion criteria were as follows: 1. age over 18 years; 2. pathologically diagnosed with RCC; 3. received laparoscopic nephrectomy. The exclusion criteria were as follows: 1. received other anticancer treatments prior to nephrectomy such as transcatheter arterial chemoembolization; 2. diagnosed with other malignancies that could seriously affect survival; 3. had incomplete medical records or follow-up data were missing. Based on the above criteria, 165 patients were excluded, and 425 patients were eventually included in this study.”</td></tr>
<tr><td colspan="2"></td><td>Dev</td><td>Val</td></tr>
<tr><td colspan="2">1.1 Were appropriate data sources used, e.g. cohort, RCT or nested case-control study data?</td><td>Y</td><td>N/A</td></tr>
<tr><td colspan="2">1.2 Were all inclusions and exclusions of participants appropriate?</td><td>N</td><td>N/A</td></tr>
<tr><td>Risk of bias introduced by selection of participants</td><td>RISK:
(low/ high/ unclear)</td><td>High</td><td>N/A</td></tr>
<tr><td colspan="4">Rationale of bias rating:
The only included patients with laparoscopic surgery.</td></tr>
<tr><td colspan="4">B. Applicability</td></tr>
<tr><td colspan="4">Describe included participants, setting and dates:

“The retrospective study included 590 patients with RCC who underwent laparoscopic nephrectomy at Zhongda Hospital Southeast University, Shanghai Tenth People’s Hospital and Shidong Hospital between January 2014 and December 2019. This study complied with the criteria outlined in the Declaration of Helsinki (as revised in 2013) and was approved by the Ethics Committee and Institutional Review Board of all participating institutions (SHSY-IECKY-4.0/18- 68/01 and ZDKYSB077). Signed informed consent was provided by all patients and their relatives who enrolled in this study.”
“The patient inclusion criteria were as follows: 1. age over 18 years; 2. pathologically diagnosed with RCC; 3. received laparoscopic nephrectomy. The exclusion criteria were as follows: 1. received other anticancer treatments prior to nephrectomy such as transcatheter arterial chemoembolization; 2. diagnosed with other malignancies that could seriously affect survival; 3. had incomplete medical records or follow-up data were missing. Based on the above criteria, 165 patients were excluded, and 425 patients were eventually included in this study.”</td></tr>
<tr><td>Concern that the included participants and setting do not match the review question</td><td>CONCERN:
(low/ high/ unclear)</td><td>High</td><td>N/A</td></tr>
<tr><td colspan="4">Rationale of applicability rating:
The only included patients with laparoscopic surgery.</td></tr>
</table>

DOMAIN 2: Predictors			
**A. Risk of Bias**			
List and describe predictors included in the final model, e.g. definition and timing of assessment:<br><br>The predictors included in the model were: T and M stage and F-NLR stage.<br><br>All the predictors were measured before and after the treatment			
		Dev	Val
2.1 Were predictors defined and assessed in a similar way for all participants?		PY	N/A
2.2 Were predictor assessments made without knowledge of outcome data?		PY	N/A
2.3 Are all predictors available at the time the model is intended to be used?		PY	N/A
**Risk of bias introduced by predictors or their assessment**	**RISK:**<br>*(low/ high/ unclear)*	Low	N/A
Rationale of bias rating:<br>*The blinding of measurement is unknown but all the predictors are objective.*			
**B. Applicability**			
Concern that the definition, assessment or timing of predictors in the model do not match the review question	**CONCERN:**<br>*(low/ high/ unclear)*	Low	N/A
Rationale of applicability rating:<br>*No major issues identified.*			

DOMAIN 3: Outcome			
**A. Risk of Bias**			
Describe the outcome, how it was defined and determined, and the time interval between predictor assessment and outcome determination:<br>The outcome was cancer specific survival at 3 and 5- years			
		Dev	Val
3.1 Was the outcome determined appropriately?		PY	N/A
3.2 Was a pre-specified or standard outcome definition used?		Y	N/A
3.3 Were predictors excluded from the outcome definition?		PY	N/A
3.4 Was the outcome defined and determined in a similar way for all participants?		PY	N/A
3.5 Was the outcome determined without knowledge of predictor information?		PY	N/A
3.6 Was the time interval between predictor assessment and outcome determination appropriate?		Y	N/A
**Risk of bias introduced by the outcome or its determination**	**RISK:**<br>*(low/ high/ unclear)*	Low	N/A
Rationale of bias rating:<br>*No major issues identified*			
**B. Applicability**			
At what time point was the outcome determined:<br>3 and 5 years<br><br>*If a composite outcome was used, describe the relative frequency/distribution of each contributing outcome:*<br>*N/A*			
**Concern that the outcome, its definition, timing or determination do not match the review question**	**CONCERN:**<br>*(low/ high/ unclear)*	Low	N/A
Rationale of applicability rating:<br>*The outcome of the primary study matches the outcome of interest of the review*			

DOMAIN 4: Analysis		
**Risk of Bias**		
Describe numbers of participants, number of candidate predictors, outcome events and events per candidate predictor:<br><br>"Based on the above cri- teria, 165 patients were excluded, and 425 patients were eventually included in this study."<br><br>The total number of predictors is 21 (Table 6)<br>The number of events unknown<br><br>EPV= unknown		
Describe how the model was developed (for example in regards to modelling technique (e.g. survival or logistic modelling), predictor selection, and risk group definition):<br>"Univariate and multivariate Cox regression models were applied to evaluate the risk factors for OS and CSS. Multivariate Cox regression analysis was used to determine the relationship of fibrinogen level, NLR, and F-NLR score with OS and CSS by constructing three models and calculating the associated unadjusted hazard ratio (HR) and 95% confi- dence interval (CI)"		
Describe whether and how the model was validated, either internally (e.g. bootstrapping, cross validation, random split sample) or externally (e.g. temporal validation, geographical validation, different setting, different type of participants):<br><br>*"Bootstrap resampling and 10-fold cross-validation were used for internal and external verification of the nomogram"*		
Describe the performance measures of the model, e.g. (re)calibration, discrimination, (re)classification, net benefit, and whether they were adjusted for optimism:<br><br>*"Harrell's concordance index (C-index) and calibration curve were used to evaluate the performance of the nomogram"*<br>*"The predictive performance of the nomogram was evaluated by applying decision curve analysis (DCA) and ROC curve analysis"*		
Describe any participants who were excluded from the analysis:<br>*"The exclusion criteria were as follows: 1. received other anticancer treatments prior to nephrectomy such as transcatheter arterial chemoembolization; 2. diagnosed with other malignancies that could seriously affect survival; 3. had incomplete medical records or follow-up data were missing"*		
Describe missing data on predictors and outcomes as well as methods used for missing data:<br>*"The exclusion criteria were as follows: 1. received other anticancer treatments prior to nephrectomy such as transcatheter arterial chemoembolization; 2. diagnosed with other malignancies that could seriously affect survival; 3. had incomplete medical records or follow-up data were missing"*		
	Dev	Val
4.1 Were there a reasonable number of participants with the outcome?	NI	N/A
4.2 Were continuous and categorical predictors handled appropriately?	N	N/A

4.3 Were all enrolled participants included in the analysis?		NI	N/A
4.4 Were participants with missing data handled appropriately?		N	N/A
4.5 Was selection of predictors based on univariable analysis avoided?		N	
4.6 Were complexities in the data (e.g. censoring, competing risks, sampling of controls) accounted for appropriately?		N	N/A
4.7 Were relevant model performance measures evaluated appropriately?		Y	N/A
4.8 Were model overfitting and optimism in model performance accounted for?		Y	
4.9 Do predictors and their assigned weights in the final model correspond to the results from multivariable analysis?		PY	
**Risk of bias introduced by the analysis**	**RISK:** *(low/ high/ unclear)*	High	N/A
Rationale of bias rating: They didn´t say the number of events. They did categorizations. They excluded patients with missing data. They selected the predictors based on univariable. They did categorizations. They didn't use competing risks.			

Step 4: Overall assessment

Use the following tables to reach overall judgements about risk of bias and concerns regarding applicability of the prediction model evaluation (development and/or validation) across all assessed domains.
Complete for each evaluation of a distinct model.

Reaching an overall judgement about risk of bias of the prediction model evaluation	
**Low risk of bias**	If all domains were rated low risk of bias.<br>If a prediction model was developed without any external validation, and it was rated as low risk of bias for all domains, consider downgrading to **high risk of bias**. Such a model can only be considered as low risk of bias, if the development was based on a very large data set and included some form of internal validation.
**High risk of bias**	If at least one domain is judged to be at **high risk of bias**.
**Unclear risk of bias**	If an unclear risk of bias was noted in at least one domain and it was low risk for all other domains.

Reaching an overall judgement about applicability of the prediction model evaluation	
**Low concerns regarding applicability**	If low concerns regarding applicability for all domains, the prediction model evaluation is judged to have **low concerns regarding applicability**.
**High concerns regarding applicability**	If high concerns regarding applicability for at least one domain, the prediction model evaluation is judged to have **high concerns regarding applicability**.
**Unclear concerns regarding applicability**	If unclear concerns (but no “high concern”) regarding applicability for at least one domain, the prediction model evaluation is judged to have **unclear concerns regarding applicability** overall.

Overall judgement about risk of bias and applicability of the prediction model evaluation		
**Overall judgement of risk of bias**	**RISK:**<br>*(low/ high/ unclear)*	High
Summary of sources of potential bias:<br>Analysis and participants domains show several major issues		
**Overall judgement of applicability**	**CONCERN:**<br>*(low/ high/ unclear)*	High
Summary of applicability concerns:<br><br>Participants domain shows several major issues		

PROBAST

(Prediction model study Risk Of Bias Assessment Tool)

Published in Annals of Internal Medicine (freely available):

1. PROBAST: A Tool to Assess the Risk of Bias and Applicability of Prediction Model Studies
2. PROBAST: A Tool to Assess Risk of Bias and Applicability of Prediction Model Studies: Explanation and Elaboration

What does PROBAST assess?

PROBAST assesses both the *risk of bias* and *concerns regarding applicability* of a study that evaluates (develops, validates or updates) a multivariable diagnostic or prognostic prediction model. It is designed to assess primary studies included in a systematic review.

Bias occurs if systematic flaws or limitations in the design, conduct or analysis of a primary study distort the results. For the purpose of prediction modelling studies, we have defined *risk of bias* to occur when shortcomings in the study design, conduct or analysis lead to systematically distorted estimates of a model's predictive performance or to an inadequate model to address the research question. Model predictive performance is typically evaluated using calibration, discrimination and sometimes classification measures, and these are likely inaccurately estimated in studies with high risk of bias. *Applicability* refers to the extent to which the prediction model from the primary study matches your systematic review question, for example in terms of the participants, predictors or outcome of interest.

A primary study may include the development and/or validation or update of more than one prediction model. A PROBAST assessment should be completed for each distinct model that is developed, validated or updated (extended) for making individualised predictions. Where a publication assesses multiple prediction models, only complete a PROBAST assessment for those models that meet the inclusion criteria for your systematic review. Please note that subsequent use of the term "model" includes derivatives of models, such as simplified risk scores, nomograms, or recalibrations of models.

PROBAST is not designed for all multivariable diagnostic or prognostic studies. For example, studies using multivariable models to identify predictors associated with an outcome but not attempting to develop a model for making individualised predictions are not covered by PROBAST.

PROBAST includes four steps.

Step	Task	When to complete
**1**	Specify your systematic review question(s)	Once per systematic review
**2**	Classify the type of prediction model evaluation	Once for each model of interest in each publication being assessed, for each relevant outcome
**3**	Assess risk of bias and applicability	Once for each development and validation of each distinct prediction model in a publication
**4**	Overall judgment	Once for each development and validation of each distinct prediction model in a publication

If this is your first time using PROBAST, we strongly recommend reading the detailed explanation and elaboration (E&E, see link above) paper and to check the examples on www.probast.org

Step 1: Specify your systematic review question

State your systematic review question to facilitate the assessment of the applicability of the evaluated models to your question. *The following table should be completed once per systematic review.*

Criteria	Specify your systematic review question
Intended use of model:	*To predict cancer specific mortality in patients with renal cancer treated with partial or total nephrectomy regardless of TNM stage*
***Participants** including selection criteria and setting:*	*Patients with renal cancer treated with partial or total nephrectomy regardless of TNM stage*
***Predictors** (used in prediction modelling), including types of predictors (e.g. history, clinical examination, biochemical markers, imaging tests), time of measurement, specific measurement issues (e.g., any requirements/ prohibitions for specialized equipment):*	*Predictors used in clinical practice measured when a nephrectomy for renal cancer is indicated*
Outcome to be predicted:	*Cancer specific mortality*

Step 2: Classify the type of prediction model evaluation

Use the following table to classify the evaluation as model development, model validation or model update, or combination. Different signalling questions apply for different types of prediction model evaluation. If the evaluation does not fit one of these classifications then PROBAST should not be used.

**Classify the evaluation based on its aim**			
**Type of prediction study**	**PROBAST boxes to complete**	**Tick as appropriate**	**Definition for type of prediction model study**
Development only	Development	✗	Prediction model development without external validation. These studies may include internal validation methods, such as bootstrapping and cross-validation techniques.
Development and validation	Development and validation	✓	Prediction model development combined with external validation in other participants in the same article.
Validation only	Validation	✗	External validation of existing (previously developed) model in other participants.

This table should be completed once for each publication being assessed and for each relevant outcome in your review.

**Publication reference**	*Wang J, Zhanghuang C, Tan X, Mi T, Liu J, Jin L, Li M, Zhang Z and He D (2022) Development and Validation of a Competitive Risk Model in Elderly Patients With Chromophobe Cell Renal Carcinoma: A Population-Based Study. Front. Public Health 10:840525. doi: 10.3389/fpubh.2022.840525*
**Models of interest**	Nomogram
**Outcome of interest**	*Cancer specific mortality*

Step 3: Assess risk of bias and applicability

PROBAST is structured as four key domains. Each domain is judged for risk of bias (low, high or unclear) and includes signalling questions to help make judgements. Signalling questions are rated as yes (Y), probably yes (PY), probably no (PN), no (N) or no information (NI). All signalling questions are phrased so that "yes" indicates absence of bias. Any signalling question rated as "no" or "probably no" flags the potential for bias; you will need to use your judgement to determine whether the domain should be rated as "high", "low" or "unclear" risk of bias. The guidance document contains further instructions and examples on rating signalling questions and risk of bias for each domain.

The first three domains are also rated for concerns regarding applicability (low/ high/ unclear) to your review question defined above.

Complete all domains separately for each evaluation of a distinct model. Shaded boxes indicate where signalling questions do not apply and should not be answered.

**DOMAIN 1: Participants**			
**A. Risk of Bias**			
Describe the sources of data and criteria for participant selection:<br><br>*“We collected clinicopathological data from patients in the Surveillance, Epidemiology, and End Results (SEER) program of the National Cancer Institute”*<br>*“Inclusion criteria: (1) Pathological diagnosis of chromophobe renal cell carcinoma (ICD-O-3 code: 8317); (2) Age ≥ 65; (3) Unilateral renal tumor. Exclusion criteria: (1) TNM staging is unknown; (2) Unknown tumor size; (3) Unknown surgical method; (4) Survival time <1 month”*			
		Dev	Val
1.1 Were appropriate data sources used, e.g. cohort, RCT or nested case-control study data?		Y	Y
1.2 Were all inclusions and exclusions of participants appropriate?		Y	Y
**Risk of bias introduced by selection of participants**	**RISK:**<br>*(low/ high/ unclear)*	Low	Low
Rationale of bias rating:<br>*No major issues identified*			
**B. Applicability**			
Describe included participants, setting and dates:<br>*“We collected clinicopathological data from patients in the Surveillance, Epidemiology, and End Results (SEER) program of the National Cancer Institute”*<br>*“Inclusion criteria: (1) Pathological diagnosis of chromophobe renal cell carcinoma (ICD-O-3 code: 8317); (2) Age ≥ 65; (3) Unilateral renal tumor. Exclusion criteria: (1) TNM staging is unknown; (2) Unknown tumor size; (3) Unknown surgical method; (4) Survival time <1 month”*			
**Concern that the included participants and setting do not match the review question**	**CONCERN:**<br>*(low/ high/ unclear)*	Low	Low
Rationale of applicability rating:<br>*No major issues identified*			

DOMAIN 2: Predictors			
**A. Risk of Bias**			
List and describe predictors included in the final model, e.g. definition and timing of assessment:<br><br>The predictors included in the model were: surgery, tumor size, TNM stage, race and age.<br><br>The predictors were measured before and after treatment			
		Dev	Val
2.1 Were predictors defined and assessed in a similar way for all participants?		PY	PY
2.2 Were predictor assessments made without knowledge of outcome data?		PY	PY
2.3 Are all predictors available at the time the model is intended to be used?		PY	PY
**Risk of bias introduced by predictors or their assessment**	**RISK:**<br>*(low/ high/ unclear)*	Low	Low
Rationale of bias rating:<br>*The blinding of measurement is unknown but all the predictors are objective.*			
**B. Applicability**			
Concern that the definition, assessment or timing of predictors in the model do not match the review question	**CONCERN:**<br>*(low/ high/ unclear)*	Low	Low
Rationale of applicability rating:<br>*No major issues identified.*			

**DOMAIN 3: Outcome**			
**A. Risk of Bias**			
Describe the outcome, how it was defined and determined, and the time interval between predictor assessment and outcome determination:<br>The outcome was cancer specific survival at 1, 3 and 5 years			
		Dev	Val
3.1 Was the outcome determined appropriately?		PY	PY
3.2 Was a pre-specified or standard outcome definition used?		Y	Y
3.3 Were predictors excluded from the outcome definition?		PY	PY
3.4 Was the outcome defined and determined in a similar way for all participants?		PY	PY
3.5 Was the outcome determined without knowledge of predictor information?		PY	PY
3.6 Was the time interval between predictor assessment and outcome determination appropriate?		Y	Y
**Risk of bias introduced by the outcome or its determination**	**RISK:**<br>*(low/ high/ unclear)*	Low	Low
Rationale of bias rating:<br>*No major issues identified*			
**B. Applicability**			
At what time point was the outcome determined:<br>1, 3 and 5 years<br><br>*If a composite outcome was used, describe the relative frequency/distribution of each contributing outcome:*<br>*N/A*			
**Concern that the outcome, its definition, timing or determination do not match the review question**	**CONCERN:**<br>*(low/ high/ unclear)*	Low	Low
Rationale of applicability rating:<br>*The outcome of the primary study matches the outcome of interest of the review*			

DOMAIN 4: Analysis

Risk of Bias

Describe numbers of participants, number of candidate predictors, outcome events and events per candidate predictor:
"A total of 3,522 elderly patients with chRCC were included in the analysis. Patients were randomly assigned to either the training cohort (N = 2,474) or the validation cohort (N = 1,048)"

A total of 18 predictors were included (Table 2)
The number of events is unknown

EPV= unknown

Describe how the model was developed (for example in regards to modelling technique (e.g. survival or logistic modelling), predictor selection, and risk group definition):

"At present, the main research methods of competitive risk include cause-specific risk model and cumulative risk model"

Describe whether and how the model was validated, either internally (e.g. bootstrapping, cross validation, random split sample) or externally (e.g. temporal validation, geographical validation, different setting, different type of participants):

Not indicated

Describe the performance measures of the model, e.g. (re)calibration, discrimination, (re)classification, net benefit, and whether they were adjusted for optimism:

"Consistency index (C-index), the area under the receiver operating curve (AUC), calibration curve were used to validate the accuracy and discrimination of the model"
"Decision curve analysis (DCA) was used to validate the clinical value of the model"

Describe any participants who were excluded from the analysis:
"Exclusion criteria; (1) TNM staging is unknown; (2) Unknown tumor size; (3) Unknown surgical method; (4) Survival time <1 month"

Describe missing data on predictors and outcomes as well as methods used for missing data:
"Exclusion criteria; (1) TNM staging is unknown; (2) Unknown tumor size; (3) Unknown surgical method; (4) Survival time <1 month"

	Dev	Val
4.1 Were there a reasonable number of participants with the outcome?	NI	PY
4.2 Were continuous and categorical predictors handled appropriately?	Y	Y
4.3 Were all enrolled participants included in the analysis?	PY	PY
4.4 Were participants with missing data handled appropriately?	N	N
4.5 Was selection of predictors based on univariable analysis avoided?	N	
4.6 Were complexities in the data (e.g. censoring, competing risks, sampling of controls) accounted for appropriately?	Y	Y
4.7 Were relevant model performance measures evaluated appropriately?	Y	Y
4.8 Were model overfitting and optimism in model performance accounted for?	N	

4.9 Do predictors and their assigned weights in the final model correspond to the results from multivariable analysis?		PY	
**Risk of bias introduced by the analysis**	**RISK:** *(low/ high/ unclear)*	High	High
Rationale of bias rating: *They didn´t say the number of events. They excluded patients with missing data. They selected the predictors based on univariable. They didn´t do bootstrapping*			

Step 4: Overall assessment

Use the following tables to reach overall judgements about risk of bias and concerns regarding applicability of the prediction model evaluation (development and/or validation) across all assessed domains.
Complete for each evaluation of a distinct model.

**Reaching an overall judgement about risk of bias of the prediction model evaluation**	
**Low risk of bias**	If all domains were rated low risk of bias.<br>If a prediction model was developed without any external validation, and it was rated as low risk of bias for all domains, consider downgrading to **high risk of bias**. Such a model can only be considered as low risk of bias, if the development was based on a very large data set and included some form of internal validation.
**High risk of bias**	If at least one domain is judged to be at **high risk of bias**.
**Unclear risk of bias**	If an unclear risk of bias was noted in at least one domain and it was low risk for all other domains.

**Reaching an overall judgement about applicability of the prediction model evaluation**	
**Low concerns regarding applicability**	If low concerns regarding applicability for all domains, the prediction model evaluation is judged to have **low concerns regarding applicability**.
**High concerns regarding applicability**	If high concerns regarding applicability for at least one domain, the prediction model evaluation is judged to have **high concerns regarding applicability**.
**Unclear concerns regarding applicability**	If unclear concerns (but no "high concern") regarding applicability for at least one domain, the prediction model evaluation is judged to have **unclear concerns regarding applicability** overall.

**Overall judgement about risk of bias and applicability of the prediction model evaluation**		
**Overall judgement of risk of bias**	**RISK:**<br>*(low/ high/ unclear)*	High
Summary of sources of potential bias:<br>Analysis domain shows several major issues		
**Overall judgement of applicability**	**CONCERN:**<br>*(low/ high/ unclear)*	Low
Summary of applicability concerns:<br><br>No major issues		

PROBAST

(Prediction model study Risk Of Bias Assessment Tool)

Published in Annals of Internal Medicine (freely available):

1. PROBAST: A Tool to Assess the Risk of Bias and Applicability of Prediction Model Studies
2. PROBAST: A Tool to Assess Risk of Bias and Applicability of Prediction Model Studies: Explanation and Elaboration

What does PROBAST assess?

PROBAST assesses both the *risk of bias* and *concerns regarding applicability* of a study that evaluates (develops, validates or updates) a multivariable diagnostic or prognostic prediction model. It is designed to assess primary studies included in a systematic review.

Bias occurs if systematic flaws or limitations in the design, conduct or analysis of a primary study distort the results. For the purpose of prediction modelling studies, we have defined *risk of bias* to occur when shortcomings in the study design, conduct or analysis lead to systematically distorted estimates of a model's predictive performance or to an inadequate model to address the research question. Model predictive performance is typically evaluated using calibration, discrimination and sometimes classification measures, and these are likely inaccurately estimated in studies with high risk of bias. *Applicability* refers to the extent to which the prediction model from the primary study matches your systematic review question, for example in terms of the participants, predictors or outcome of interest.

A primary study may include the development and/or validation or update of more than one prediction model. A PROBAST assessment should be completed for each distinct model that is developed, validated or updated (extended) for making individualised predictions. Where a publication assesses multiple prediction models, only complete a PROBAST assessment for those models that meet the inclusion criteria for your systematic review. Please note that subsequent use of the term "model" includes derivatives of models, such as simplified risk scores, nomograms, or recalibrations of models.

PROBAST is not designed for all multivariable diagnostic or prognostic studies. For example, studies using multivariable models to identify predictors associated with an outcome but not attempting to develop a model for making individualised predictions are not covered by PROBAST.

PROBAST includes four steps.

Step	Task	When to complete
**1**	Specify your systematic review question(s)	Once per systematic review
**2**	Classify the type of prediction model evaluation	Once for each model of interest in each publication being assessed, for each relevant outcome
**3**	Assess risk of bias and applicability	Once for each development and validation of each distinct prediction model in a publication
**4**	Overall judgment	Once for each development and validation of each distinct prediction model in a publication

If this is your first time using PROBAST, we strongly recommend reading the detailed explanation and elaboration (E&E, see link above) paper and to check the examples on www.probast.org

Step 1: Specify your systematic review question

State your systematic review question to facilitate the assessment of the applicability of the evaluated models to your question. *The following table should be completed once per systematic review.*

Criteria	Specify your systematic review question
Intended use of model:	*To predict cancer specific mortality in patients with renal cancer treated with partial or total nephrectomy regardless of TNM stage*
***Participants** including selection criteria and setting:*	*Patients with renal cancer treated with partial or total nephrectomy regardless of TNM stage*
***Predictors** (used in prediction modelling), including types of predictors (e.g. history, clinical examination, biochemical markers, imaging tests), time of measurement, specific measurement issues (e.g., any requirements/ prohibitions for specialized equipment):*	*Predictors used in clinical practice measured when a nephrectomy for renal cancer is indicated*
Outcome to be predicted:	*Cancer specific mortality*

Step 2: Classify the type of prediction model evaluation

Use the following table to classify the evaluation as model development, model validation or model update, or combination. Different signalling questions apply for different types of prediction model evaluation. If the evaluation does not fit one of these classifications then PROBAST should not be used.

**Classify the evaluation based on its aim**			
**Type of prediction study**	**PROBAST boxes to complete**	**Tick as appropriate**	**Definition for type of prediction model study**
Development only	Development	✗	Prediction model development without external validation. These studies may include internal validation methods, such as bootstrapping and cross-validation techniques.
Development and validation	Development and validation	✓	Prediction model development combined with external validation in other participants in the same article.
Validation only	Validation	✗	External validation of existing (previously developed) model in other participants.

This table should be completed once for each publication being assessed and for each relevant outcome in your review.

**Publication reference**	*Tang J, Wang J, Pan X, Liu X and Zhao B (2022) A Web-Based Prediction Model for Cancer-Specific Survival of Middle-Aged Patients With Non-metastatic Renal Cell Carcinoma: A Population-Based Study. Front. Public Health 10:822808. doi: 10.3389/fpubh.2022.822808*
**Models of interest**	Nomogram and risk score
**Outcome of interest**	*Cancer specific mortality*

Step 3: Assess risk of bias and applicability

PROBAST is structured as four key domains. Each domain is judged for risk of bias (low, high or unclear) and includes signalling questions to help make judgements. Signalling questions are rated as yes (Y), probably yes (PY), probably no (PN), no (N) or no information (NI). All signalling questions are phrased so that "yes" indicates absence of bias. Any signalling question rated as "no" or "probably no" flags the potential for bias; you will need to use your judgement to determine whether the domain should be rated as "high", "low" or "unclear" risk of bias. The guidance document contains further instructions and examples on rating signalling questions and risk of bias for each domain.

The first three domains are also rated for concerns regarding applicability (low/ high/ unclear) to your review question defined above.

Complete all domains separately for each evaluation of a distinct model. Shaded boxes indicate where signalling questions do not apply and should not be answered.

DOMAIN 1: Participants			
**A. Risk of Bias**			
Describe the sources of data and criteria for participant selection:<br><br>*“We downloaded the clinical-pathological data of the patients from the National Cancer Institute’s Surveillance, Epidemiology, and Final Results (SEER) project, including patients who were diagnosed with nmRCC in the United States from 2010 to 2018 between 40 and 60 years old”*<br>*“Inclusion criteria: (1) age 40-60 years; (2) pathological diagnosis of renal cell carcinoma (ICD-O-3 codes 8260, 8310, 8312, 8317); (3) diagnosis year 2010-2018. Exclusion criteria: (1) unknown race; (2) unknown tumor size; (3) unknown surgical method; (4) unknown T stage; (5) survival time <1 month; (6) unknown cause of death”*			
		Dev	Val
1.1 Were appropriate data sources used, e.g. cohort, RCT or nested case-control study data?		Y	Y
1.2 Were all inclusions and exclusions of participants appropriate?		Y	Y
**Risk of bias introduced by selection of participants**	**RISK:**<br>*(low/ high/ unclear)*	Low	Low
Rationale of bias rating:<br>*No major issues identified*			
**B. Applicability**			
Describe included participants, setting and dates:<br><br>*“We downloaded the clinical-pathological data of the patients from the National Cancer Institute’s Surveillance, Epidemiology, and Final Results (SEER) project, including patients who were diagnosed with nmRCC in the United States from 2010 to 2018 between 40 and 60 years old”*<br>*“Inclusion criteria: (1) age 40-60 years; (2) pathological diagnosis of renal cell carcinoma (ICD-O-3 codes 8260, 8310, 8312, 8317); (3) diagnosis year 2010-2018. Exclusion criteria: (1) unknown race; (2) unknown tumor size; (3) unknown surgical method; (4) unknown T stage; (5) survival time <1 month; (6) unknown cause of death”*			
**Concern that the included participants and setting do not match the review question**	**CONCERN:**<br>*(low/ high/ unclear)*	Low	Low
Rationale of applicability rating:<br>*No major issues identified*			

DOMAIN 2: Predictors			
**A. Risk of Bias**			
List and describe predictors included in the final model, e.g. definition and timing of assessment:<br><br>The predictors included in the model were: age, sex, grade, T stage, surgery and tumor size<br><br>The predictors were measured before and after treatment			
		Dev	Val
2.1 Were predictors defined and assessed in a similar way for all participants?		PY	PY
2.2 Were predictor assessments made without knowledge of outcome data?		PY	PY
2.3 Are all predictors available at the time the model is intended to be used?		PY	PY
**Risk of bias introduced by predictors or their assessment**	**RISK:**<br>*(low/ high/ unclear)*	Low	Low
Rationale of bias rating:<br>*The blinding of measurement is unknown but all the predictors are objective.*			
**B. Applicability**			
Concern that the definition, assessment or timing of predictors in the model do not match the review question	**CONCERN:**<br>*(low/ high/ unclear)*	Low	Low
Rationale of applicability rating:<br>*No major issues identified.*			

DOMAIN 3: Outcome			
**A. Risk of Bias**			
Describe the outcome, how it was defined and determined, and the time interval between predictor assessment and outcome determination:<br>The outcome was cancer specific survival at 1, 3 and 5 years			
		Dev	Val
3.1 Was the outcome determined appropriately?		PY	PY
3.2 Was a pre-specified or standard outcome definition used?		Y	Y
3.3 Were predictors excluded from the outcome definition?		PY	PY
3.4 Was the outcome defined and determined in a similar way for all participants?		PY	PY
3.5 Was the outcome determined without knowledge of predictor information?		PY	PY
3.6 Was the time interval between predictor assessment and outcome determination appropriate?		Y	Y
**Risk of bias introduced by the outcome or its determination**	**RISK:**<br>*(low/ high/ unclear)*	Low	Low
Rationale of bias rating:<br>*No major issues identified*			
**B. Applicability**			
At what time point was the outcome determined:<br>1, 3 and 5 years<br><br>*If a composite outcome was used, describe the relative frequency/distribution of each contributing outcome:*<br>*N/A*			
**Concern that the outcome, its definition, timing or determination do not match the review question**	**CONCERN:**<br>*(low/ high/ unclear)*	Low	Low
Rationale of applicability rating:<br>*The outcome of the primary study matches the outcome of interest of the review*			

DOMAIN 4: Analysis
**Risk of Bias**
Describe numbers of participants, number of candidate predictors, outcome events and events per candidate predictor:<br>*"According to the inclusion and exclusion criteria, a total of 27,073 patients were included in the study. These patients were randomly divided into a training set (N = 18,990) and a validation set (N = 8,083)"*<br><br>*A total of 22 predictors were included (Table 2)*<br>*The number of events is unknown*<br><br>*EPV= unknown*
Describe how the model was developed (for example in regards to modelling technique (e.g. survival or logistic modelling), predictor selection, and risk group definition):<br><br>*"In the training set, univariate and multivariate Cox regression models were used to analyze independent risk factors for survival of nmRCC patients, and the hazard ratio (HR) and 95% confidence interval (CI) were recorded"*
Describe whether and how the model was validated, either internally (e.g. bootstrapping, cross validation, random split sample) or externally (e.g. temporal validation, geographical validation, different setting, different type of participants):<br>*"The calibration curve was used to test the accuracy of the prediction model, and we used 1,000 bootstrap samples for internal validation"*
Describe the performance measures of the model, e.g. (re)calibration, discrimination, (re)classification, net benefit, and whether they were adjusted for optimism:<br><br>*"The 1-, 3-, and 5-year areas under the receiver operating curve (AUC) of the training set and the validation set were used to test the accuracy and discrimination of the prediction model. Similarly, we used the consistency index (C- index) to test the discriminative power of the model"*<br>*"Decision curve analysis (DCA) is a new calculation method that estimates the net benefits under various risk thresholds to evaluate the clinical value of the model"*
Describe any participants who were excluded from the analysis:<br>*"Exclusion criteria: (1) unknown race; (2) unknown tumor size; (3) unknown surgical method; (4) unknown T stage; (5) survival time <1 month; (6) unknown cause of death"*
Describe missing data on predictors and outcomes as well as methods used for missing data:<br>*"Exclusion criteria: (1) unknown race; (2) unknown tumor size; (3) unknown surgical method; (4) unknown T stage; (5) survival time <1 month; (6) unknown cause of death"*

	Dev	Val
4.1 Were there a reasonable number of participants with the outcome?	NI	PY
4.2 Were continuous and categorical predictors handled appropriately?	Y	Y
4.3 Were all enrolled participants included in the analysis?	PY	PY
4.4 Were participants with missing data handled appropriately?	N	N
4.5 Was selection of predictors based on univariable analysis avoided?	N	
4.6 Were complexities in the data (e.g. censoring, competing risks, sampling of controls)	N	N

accounted for appropriately?			
4.7 Were relevant model performance measures evaluated appropriately?		Y	Y
4.8 Were model overfitting and optimism in model performance accounted for?		Y	
4.9 Do predictors and their assigned weights in the final model correspond to the results from multivariable analysis?		PY	
**Risk of bias introduced by the analysis**	**RISK:** *(low/ high/ unclear)*	High	High
Rationale of bias rating: *They didn´t say the number of events. They excluded patients with missing data. They selected the predictors based on univariable. They didn´t do competing risk.*			

Step 4: Overall assessment

Use the following tables to reach overall judgements about risk of bias and concerns regarding applicability of the prediction model evaluation (development and/or validation) across all assessed domains.
Complete for each evaluation of a distinct model.

**Reaching an overall judgement about risk of bias of the prediction model evaluation**	
**Low risk of bias**	If all domains were rated low risk of bias.<br>If a prediction model was developed without any external validation, and it was rated as low risk of bias for all domains, consider downgrading to **high risk of bias**. Such a model can only be considered as low risk of bias, if the development was based on a very large data set and included some form of internal validation.
**High risk of bias**	If at least one domain is judged to be at **high risk of bias**.
**Unclear risk of bias**	If an unclear risk of bias was noted in at least one domain and it was low risk for all other domains.

**Reaching an overall judgement about applicability of the prediction model evaluation**	
**Low concerns regarding applicability**	If low concerns regarding applicability for all domains, the prediction model evaluation is judged to have **low concerns regarding applicability**.
**High concerns regarding applicability**	If high concerns regarding applicability for at least one domain, the prediction model evaluation is judged to have **high concerns regarding applicability**.
**Unclear concerns regarding applicability**	If unclear concerns (but no "high concern") regarding applicability for at least one domain, the prediction model evaluation is judged to have **unclear concerns regarding applicability** overall.

**Overall judgement about risk of bias and applicability of the prediction model evaluation**		
**Overall judgement of risk of bias**	**RISK:**<br>*(low/ high/ unclear)*	High
Summary of sources of potential bias:<br>Analysis domain shows several major issues		
**Overall judgement of applicability**	**CONCERN:**<br>*(low/ high/ unclear)*	Low
Summary of applicability concerns:<br><br>No major issues		

PROBAST

(Prediction model study Risk Of Bias Assessment Tool)

Published in Annals of Internal Medicine (freely available):

1. PROBAST: A Tool to Assess the Risk of Bias and Applicability of Prediction Model Studies
2. PROBAST: A Tool to Assess Risk of Bias and Applicability of Prediction Model Studies: Explanation and Elaboration

What does PROBAST assess?

PROBAST assesses both the *risk of bias* and *concerns regarding applicability* of a study that evaluates (develops, validates or updates) a multivariable diagnostic or prognostic prediction model. It is designed to assess primary studies included in a systematic review.

Bias occurs if systematic flaws or limitations in the design, conduct or analysis of a primary study distort the results. For the purpose of prediction modelling studies, we have defined *risk of bias* to occur when shortcomings in the study design, conduct or analysis lead to systematically distorted estimates of a model's predictive performance or to an inadequate model to address the research question. Model predictive performance is typically evaluated using calibration, discrimination and sometimes classification measures, and these are likely inaccurately estimated in studies with high risk of bias. *Applicability* refers to the extent to which the prediction model from the primary study matches your systematic review question, for example in terms of the participants, predictors or outcome of interest.

A primary study may include the development and/or validation or update of more than one prediction model. A PROBAST assessment should be completed for each distinct model that is developed, validated or updated (extended) for making individualised predictions. Where a publication assesses multiple prediction models, only complete a PROBAST assessment for those models that meet the inclusion criteria for your systematic review. Please note that subsequent use of the term "model" includes derivatives of models, such as simplified risk scores, nomograms, or recalibrations of models.

PROBAST is not designed for all multivariable diagnostic or prognostic studies. For example, studies using multivariable models to identify predictors associated with an outcome but not attempting to develop a model for making individualised predictions are not covered by PROBAST.

PROBAST includes four steps.

Step	Task	When to complete
**1**	Specify your systematic review question(s)	Once per systematic review
**2**	Classify the type of prediction model evaluation	Once for each model of interest in each publication being assessed, for each relevant outcome
**3**	Assess risk of bias and applicability	Once for each development and validation of each distinct prediction model in a publication
**4**	Overall judgment	Once for each development and validation of each distinct prediction model in a publication

If this is your first time using PROBAST, we strongly recommend reading the detailed explanation and elaboration (E&E, see link above) paper and to check the examples on www.probast.org

Step 1: Specify your systematic review question

State your systematic review question to facilitate the assessment of the applicability of the evaluated models to your question. *The following table should be completed once per systematic review.*

Criteria	Specify your systematic review question
Intended use of model:	*To predict cancer specific mortality in patients with renal cancer treated with partial or total nephrectomy regardless of TNM stage*
***Participants** including selection criteria and setting:*	*Patients with renal cancer treated with partial or total nephrectomy regardless of TNM stage*
***Predictors** (used in prediction modelling), including types of predictors (e.g. history, clinical examination, biochemical markers, imaging tests), time of measurement, specific measurement issues (e.g., any requirements/ prohibitions for specialized equipment):*	*Predictors used in clinical practice measured when a nephrectomy for renal cancer is indicated*
Outcome to be predicted:	*Cancer specific mortality*

Step 2: Classify the type of prediction model evaluation

Use the following table to classify the evaluation as model development, model validation or model update, or combination. Different signalling questions apply for different types of prediction model evaluation. If the evaluation does not fit one of these classifications then PROBAST should not be used.

**Classify the evaluation based on its aim**			
**Type of prediction study**	**PROBAST boxes to complete**	**Tick as appropriate**	**Definition for type of prediction model study**
Development only	Development	✗	Prediction model development without external validation. These studies may include internal validation methods, such as bootstrapping and cross-validation techniques.
Development and validation	Development and validation	✓	Prediction model development combined with external validation in other participants in the same article.
Validation only	Validation	✗	External validation of existing (previously developed) model in other participants.

This table should be completed once for each publication being assessed and for each relevant outcome in your review.

**Publication reference**	*Zhanghuang C, Wang J, Yao Z, Li L, Xie Y, Tang H, Zhang K, Wu C, Yang Z and Yan B (2022) Development and Validation of a Nomogram to Predict Cancer-Specific Survival in Elderly Patients With Papillary Renal Cell Carcinoma. Front. Public Health 10:874427. doi: 10.3389/fpubh.2022.874427*
**Models of interest**	Nomogram and risk score
**Outcome of interest**	*Cancer specific mortality*

Step 3: Assess risk of bias and applicability

PROBAST is structured as four key domains. Each domain is judged for risk of bias (low, high or unclear) and includes signalling questions to help make judgements. Signalling questions are rated as yes (Y), probably yes (PY), probably no (PN), no (N) or no information (NI). All signalling questions are phrased so that "yes" indicates absence of bias. Any signalling question rated as "no" or "probably no" flags the potential for bias; you will need to use your judgement to determine whether the domain should be rated as "high", "low" or "unclear" risk of bias. The guidance document contains further instructions and examples on rating signalling questions and risk of bias for each domain.

The first three domains are also rated for concerns regarding applicability (low/ high/ unclear) to your review question defined above.

Complete all domains separately for each evaluation of a distinct model. Shaded boxes indicate where signalling questions do not apply and should not be answered.

DOMAIN 1: Participants			
**A. Risk of Bias**			
Describe the sources of data and criteria for participant selection:<br><br>*"We downloaded clinicopathological information of all patients with pRCC from 2004 to 2018 to the SEER database"*<br>*"Inclusion criteria:(1) pathological diagnosis of papillary renal cell carcinoma (ICD-O-3 code, 8260); (2) Age ≥65; (3) Unilateral renal tumor. Exclusion criteria:(1) TNM staging is unknown; (2) Tumor size is unknown; (3) Unknown surgical method; (4) Survival time <1 month"*			
		Dev	Val
1.1 Were appropriate data sources used, e.g. cohort, RCT or nested case-control study data?		Y	Y
1.2 Were all inclusions and exclusions of participants appropriate?		Y	Y
**Risk of bias introduced by selection of participants**	**RISK:**<br>*(low/ high/ unclear)*	Low	Low
Rationale of bias rating:<br>*No major issues identified*			
**B. Applicability**			
Describe included participants, setting and dates:<br><br>*"We downloaded clinicopathological information of all patients with pRCC from 2004 to 2018 to the SEER database"*<br>*"Inclusion criteria:(1) pathological diagnosis of papillary renal cell carcinoma (ICD-O-3 code, 8260); (2) Age ≥65; (3) Unilateral renal tumor. Exclusion criteria:(1) TNM staging is unknown; (2) Tumor size is unknown; (3) Unknown surgical method; (4) Survival time <1 month"*			
**Concern that the included participants and setting do not match the review question**	**CONCERN:**<br>*(low/ high/ unclear)*	Low	Low
Rationale of applicability rating:<br>*No major issues identified*			

DOMAIN 2: Predictors			
**A. Risk of Bias**			
List and describe predictors included in the final model, e.g. definition and timing of assessment:<br><br>The predictors included in the model were: radiation, surgery, tumor size, TNM stage, grade, sex and age<br><br>The predictors were measured before and after treatment			
		Dev	Val
2.1 Were predictors defined and assessed in a similar way for all participants?		PY	PY
2.2 Were predictor assessments made without knowledge of outcome data?		PY	PY
2.3 Are all predictors available at the time the model is intended to be used?		PY	PY
**Risk of bias introduced by predictors or their assessment**	**RISK:**<br>*(low/ high/ unclear)*	Low	Low
Rationale of bias rating:<br>*The blinding of measurement is unknown but all the predictors are objective.*			
**B. Applicability**			
Concern that the definition, assessment or timing of predictors in the model do not match the review question	**CONCERN:**<br>*(low/ high/ unclear)*	Low	Low
Rationale of applicability rating:<br>*No major issues identified.*			

<table>
<tr><td colspan="4">DOMAIN 3: Outcome</td></tr>
<tr><td colspan="4">A. Risk of Bias</td></tr>
<tr><td colspan="4">Describe the outcome, how it was defined and determined, and the time interval between predictor assessment and outcome determination:
The outcome was cancer specific survival at 1, 3 and 5 years</td></tr>
<tr><td colspan="2"></td><td>Dev</td><td>Val</td></tr>
<tr><td colspan="2">3.1 Was the outcome determined appropriately?</td><td>PY</td><td>PY</td></tr>
<tr><td colspan="2">3.2 Was a pre-specified or standard outcome definition used?</td><td>Y</td><td>Y</td></tr>
<tr><td colspan="2">3.3 Were predictors excluded from the outcome definition?</td><td>PY</td><td>PY</td></tr>
<tr><td colspan="2">3.4 Was the outcome defined and determined in a similar way for all participants?</td><td>PY</td><td>PY</td></tr>
<tr><td colspan="2">3.5 Was the outcome determined without knowledge of predictor information?</td><td>PY</td><td>PY</td></tr>
<tr><td colspan="2">3.6 Was the time interval between predictor assessment and outcome determination appropriate?</td><td>Y</td><td>Y</td></tr>
<tr><td>Risk of bias introduced by the outcome or its determination</td><td>RISK:
(low/ high/ unclear)</td><td>Low</td><td>Low</td></tr>
<tr><td colspan="4">Rationale of bias rating:
No major issues identified</td></tr>
<tr><td colspan="4">B. Applicability</td></tr>
<tr><td colspan="4">At what time point was the outcome determined:
1, 3 and 5 years

If a composite outcome was used, describe the relative frequency/distribution of each contributing outcome:
N/A</td></tr>
<tr><td>Concern that the outcome, its definition, timing or determination do not match the review question</td><td>CONCERN:
(low/ high/ unclear)</td><td>Low</td><td>Low</td></tr>
<tr><td colspan="4">Rationale of applicability rating:
The outcome of the primary study matches the outcome of interest of the review</td></tr>
</table>

DOMAIN 4: Analysis		
**Risk of Bias**		
Describe numbers of participants, number of candidate predictors, outcome events and events per candidate predictor:<br>*"Based on inclusion and exclusion criteria, a total of 13,105 elderly patients with pRCC were included. All patients were divided into a training cohort (N = 9250) and a validation cohort (N = 3855)"*<br><br>*A total of 26 predictors were included (Table 2)*<br>*The number of events is unknown*<br><br>*EPV= unknown*		
Describe how the model was developed (for example in regards to modelling technique (e.g. survival or logistic modelling), predictor selection, and risk group definition):<br><br>*"In the training cohort, we used a univariate Cox regression model to pre- screen the influencing factors of patients' prognoses. We then used a multivariate Cox proportional risk regression model to determine the independent risk factors for CSS in patients"*		
Describe whether and how the model was validated, either internally (e.g. bootstrapping, cross validation, random split sample) or externally (e.g. temporal validation, geographical validation, different setting, different type of participants):<br>*"Calibration curves of 1,000 bootstrap samples were used to validate the model's accuracy"*		
Describe the performance measures of the model, e.g. (re)calibration, discrimination, (re)classification, net benefit, and whether they were adjusted for optimism:<br><br>*"We used consistency index (C-index) and area under the receiver operating curve (AUC) to test the model's discrimination"*<br>*"DCA was used to validate the clinical utility of the nomogram"*		
Describe any participants who were excluded from the analysis:<br>*"Exclusion criteria:(1) TNM staging is unknown; (2) Tumor size is unknown; (3) Unknown surgical method; (4) Survival time <1 month"*		
Describe missing data on predictors and outcomes as well as methods used for missing data:<br>*"Exclusion criteria:(1) TNM staging is unknown; (2) Tumor size is unknown; (3) Unknown surgical method; (4) Survival time <1 month"*		
	Dev	Val
4.1 Were there a reasonable number of participants with the outcome?	NI	PY
4.2 Were continuous and categorical predictors handled appropriately?	N	N
4.3 Were all enrolled participants included in the analysis?	PY	PY
4.4 Were participants with missing data handled appropriately?	N	N
4.5 Was selection of predictors based on univariable analysis avoided?	N	
4.6 Were complexities in the data (e.g. censoring, competing risks, sampling of controls) accounted for appropriately?	N	N
4.7 Were relevant model performance measures evaluated appropriately?	Y	Y
4.8 Were model overfitting and optimism in model performance accounted for?	Y	
4.9 Do predictors and their assigned weights in the final model correspond to the	PY	

results from multivariable analysis?			
**Risk of bias introduced by the analysis**	**RISK:** *(low/ high/ unclear)*	High	High

Rationale of bias rating:
They didn´t say the number of events. They did categorisation. They excluded patients with missing data. They selected the predictors based on univariable. They didn´t do competing risk.

Step 4: Overall assessment

Use the following tables to reach overall judgements about risk of bias and concerns regarding applicability of the prediction model evaluation (development and/or validation) across all assessed domains.
Complete for each evaluation of a distinct model.

Reaching an overall judgement about risk of bias of the prediction model evaluation	
**Low risk of bias**	If all domains were rated low risk of bias.<br>If a prediction model was developed without any external validation, and it was rated as low risk of bias for all domains, consider downgrading to **high risk of bias**. Such a model can only be considered as low risk of bias, if the development was based on a very large data set and included some form of internal validation.
**High risk of bias**	If at least one domain is judged to be at **high risk of bias**.
**Unclear risk of bias**	If an unclear risk of bias was noted in at least one domain and it was low risk for all other domains.

Reaching an overall judgement about applicability of the prediction model evaluation	
**Low concerns regarding applicability**	If low concerns regarding applicability for all domains, the prediction model evaluation is judged to have **low concerns regarding applicability**.
**High concerns regarding applicability**	If high concerns regarding applicability for at least one domain, the prediction model evaluation is judged to have **high concerns regarding applicability**.
**Unclear concerns regarding applicability**	If unclear concerns (but no "high concern") regarding applicability for at least one domain, the prediction model evaluation is judged to have **unclear concerns regarding applicability** overall.

Overall judgement about risk of bias and applicability of the prediction model evaluation		
**Overall judgement of risk of bias**	**RISK:**<br>*(low/ high/ unclear)*	High
Summary of sources of potential bias:<br>Analysis domain shows several major issues		
**Overall judgement of applicability**	**CONCERN:**<br>*(low/ high/ unclear)*	Low
Summary of applicability concerns:<br><br>No major issues		

PROBAST

(Prediction model study Risk Of Bias Assessment Tool)

Published in Annals of Internal Medicine (freely available):

1. PROBAST: A Tool to Assess the Risk of Bias and Applicability of Prediction Model Studies
2. PROBAST: A Tool to Assess Risk of Bias and Applicability of Prediction Model Studies: Explanation and Elaboration

What does PROBAST assess?

PROBAST assesses both the *risk of bias* and *concerns regarding applicability* of a study that evaluates (develops, validates or updates) a multivariable diagnostic or prognostic prediction model. It is designed to assess primary studies included in a systematic review.

Bias occurs if systematic flaws or limitations in the design, conduct or analysis of a primary study distort the results. For the purpose of prediction modelling studies, we have defined *risk of bias* to occur when shortcomings in the study design, conduct or analysis lead to systematically distorted estimates of a model's predictive performance or to an inadequate model to address the research question. Model predictive performance is typically evaluated using calibration, discrimination and sometimes classification measures, and these are likely inaccurately estimated in studies with high risk of bias. *Applicability* refers to the extent to which the prediction model from the primary study matches your systematic review question, for example in terms of the participants, predictors or outcome of interest.

A primary study may include the development and/or validation or update of more than one prediction model. A PROBAST assessment should be completed for each distinct model that is developed, validated or updated (extended) for making individualised predictions. Where a publication assesses multiple prediction models, only complete a PROBAST assessment for those models that meet the inclusion criteria for your systematic review. Please note that subsequent use of the term "model" includes derivatives of models, such as simplified risk scores, nomograms, or recalibrations of models.

PROBAST is not designed for all multivariable diagnostic or prognostic studies. For example, studies using multivariable models to identify predictors associated with an outcome but not attempting to develop a model for making individualised predictions are not covered by PROBAST.

PROBAST includes four steps.

Step	Task	When to complete
**1**	Specify your systematic review question(s)	Once per systematic review
**2**	Classify the type of prediction model evaluation	Once for each model of interest in each publication being assessed, for each relevant outcome
**3**	Assess risk of bias and applicability	Once for each development and validation of each distinct prediction model in a publication
**4**	Overall judgment	Once for each development and validation of each distinct prediction model in a publication

If this is your first time using PROBAST, we strongly recommend reading the detailed explanation and elaboration (E&E, see link above) paper and to check the examples on www.probast.org

Step 1: Specify your systematic review question

State your systematic review question to facilitate the assessment of the applicability of the evaluated models to your question. *The following table should be completed once per systematic review.*

Criteria	Specify your systematic review question
Intended use of model:	*To predict cancer specific mortality in patients with renal cancer treated with partial or total nephrectomy regardless of TNM stage*
***Participants** including selection criteria and setting:*	*Patients with renal cancer treated with partial or total nephrectomy regardless of TNM stage*
***Predictors** (used in prediction modelling), including types of predictors (e.g. history, clinical examination, biochemical markers, imaging tests), time of measurement, specific measurement issues (e.g., any requirements/ prohibitions for specialized equipment):*	*Predictors used in clinical practice measured when a nephrectomy for renal cancer is indicated*
Outcome to be predicted:	*Cancer specific mortality*

Step 2: Classify the type of prediction model evaluation

Use the following table to classify the evaluation as model development, model validation or model update, or combination. Different signalling questions apply for different types of prediction model evaluation. If the evaluation does not fit one of these classifications then PROBAST should not be used.

**Classify the evaluation based on its aim**			
**Type of prediction study**	**PROBAST boxes to complete**	**Tick as appropriate**	**Definition for type of prediction model study**
Development only	Development	✗	Prediction model development without external validation. These studies may include internal validation methods, such as bootstrapping and cross-validation techniques.
Development and validation	Development and validation	✓	Prediction model development combined with external validation in other participants in the same article.
Validation only	Validation	✗	External validation of existing (previously developed) model in other participants.

This table should be completed once for each publication being assessed and for each relevant outcome in your review.

**Publication reference**	Guo P, Wang Y, Han Y, Wei D, Zhao J, Li M, Jiang Y, Luo Y. Development and validation of a nomogram to predict postoperative cancer-specific survival of patients with nonmetastatic T3a renal cell carcinoma. Urol Oncol. 2021 Dec;39(12):835.e19-835.e27. doi: 10.1016/j.urolonc.2021.06.014. Epub 2021 Oct 4. PMID: 34620554.
**Models of interest**	Nomogram
**Outcome of interest**	*Cancer specific mortality*

Step 3: Assess risk of bias and applicability

PROBAST is structured as four key domains. Each domain is judged for risk of bias (low, high or unclear) and includes signalling questions to help make judgements. Signalling questions are rated as yes (Y), probably yes (PY), probably no (PN), no (N) or no information (NI). All signalling questions are phrased so that “yes” indicates absence of bias. Any signalling question rated as “no” or “probably no” flags the potential for bias; you will need to use your judgement to determine whether the domain should be rated as “high”, “low” or “unclear” risk of bias. The guidance document contains further instructions and examples on rating signalling questions and risk of bias for each domain.

The first three domains are also rated for concerns regarding applicability (low/ high/ unclear) to your review question defined above.

Complete all domains separately for each evaluation of a distinct model. Shaded boxes indicate where signalling questions do not apply and should not be answered.

<table>
<tr><th colspan="4">DOMAIN 1: Participants</th></tr>
<tr><th colspan="4">A. Risk of Bias</th></tr>
<tr><td colspan="4">Describe the sources of data and criteria for participant selection:

“After approval from the SEER office (username: 25738- Nov2019), we searched the SEER-18 registries database to identify the patients diagnosed with T1-3aN0-1M0 RCC (C64.9) between 2010 and 2018, based on the seventh or eighth edition of the AJCC TNM staging system”
“Only patients with restaged T3aN0-1M0 RCC were included. Patients who were ≥-18-years-old at diagnosis, underwent partial or radical nephrectomy (PN or RN, RX Summ—Surg Prim Site [1988+]: 30 and 50) and had available information on gender, ethnicity, laterality, size, Fuhrman grade, sarcomatoid, and number of nodes removed were selected. Withal, only clear-cell, papillary, and chromophobe RCC were included. Patients with unknown cause of death or unreliable information on the aforementioned variables were excluded. Only cases with more than 1-month follow-up were included”</td></tr>
<tr><td colspan="2"></td><td>Dev</td><td>Val</td></tr>
<tr><td colspan="2">1.1 Were appropriate data sources used, e.g. cohort, RCT or nested case-control study data?</td><td>Y</td><td>Y</td></tr>
<tr><td colspan="2">1.2 Were all inclusions and exclusions of participants appropriate?</td><td>Y</td><td>Y</td></tr>
<tr><td>Risk of bias introduced by selection of participants</td><td>RISK:
(low/ high/ unclear)</td><td>Low</td><td>Low</td></tr>
<tr><td colspan="4">Rationale of bias rating:
No major issues identified</td></tr>
<tr><th colspan="4">B. Applicability</th></tr>
<tr><td colspan="4">Describe included participants, setting and dates:
“After approval from the SEER office (username: 25738- Nov2019), we searched the SEER-18 registries database to identify the patients diagnosed with T1-3aN0-1M0 RCC (C64.9) between 2010 and 2018, based on the seventh or eighth edition of the AJCC TNM staging system”
“Only patients with restaged T3aN0-1M0 RCC were included. Patients who were ≥-18-years-old at diagnosis, underwent partial or radical nephrectomy (PN or RN, RX Summ—Surg Prim Site [1988+]: 30 and 50) and had available information on gender, ethnicity, laterality, size, Fuhrman grade, sarcomatoid, and number of nodes removed were selected. Withal, only clear-cell, papillary, and chromophobe RCC were included. Patients with unknown cause of death or unreliable information on the aforementioned variables were excluded. Only cases with more than 1-month follow-up were included”</td></tr>
<tr><td>Concern that the included participants and setting do not match the review question</td><td>CONCERN:
(low/ high/ unclear)</td><td>Low</td><td>Low</td></tr>
<tr><td colspan="4">Rationale of applicability rating:
No major issues identified</td></tr>
</table>

<table>
<tr><th colspan="4">DOMAIN 2: Predictors</th></tr>
<tr><th colspan="4">A. Risk of Bias</th></tr>
<tr><td colspan="4">List and describe predictors included in the final model, e.g. definition and timing of assessment:

The predictors included in the model were: age, size, surgery, Fuhrman grade, histology, sarcomatoid, TN stage.

The predictors were measured before and after treatment</td></tr>
<tr><td colspan="2"></td><td>Dev</td><td>Val</td></tr>
<tr><td colspan="2">2.1 Were predictors defined and assessed in a similar way for all participants?</td><td>PY</td><td>PY</td></tr>
<tr><td colspan="2">2.2 Were predictor assessments made without knowledge of outcome data?</td><td>PY</td><td>PY</td></tr>
<tr><td colspan="2">2.3 Are all predictors available at the time the model is intended to be used?</td><td>PY</td><td>PY</td></tr>
<tr><td>Risk of bias introduced by predictors or their assessment</td><td>RISK:
(low/ high/ unclear)</td><td>Low</td><td>Low</td></tr>
<tr><td colspan="4">Rationale of bias rating:
The blinding of measurement is unknown but all the predictors are objective.</td></tr>
<tr><th colspan="4">B. Applicability</th></tr>
<tr><td>Concern that the definition, assessment or timing of predictors in the model do not match the review question</td><td>CONCERN:
(low/ high/ unclear)</td><td>Low</td><td>Low</td></tr>
<tr><td colspan="4">Rationale of applicability rating:
No major issues identified.</td></tr>
</table>

**DOMAIN 3: Outcome**			
**A. Risk of Bias**			
Describe the outcome, how it was defined and determined, and the time interval between predictor assessment and outcome determination:<br>The outcome was cancer specific survival at 3 and 5 years			
		Dev	Val
3.1 Was the outcome determined appropriately?		PY	PY
3.2 Was a pre-specified or standard outcome definition used?		Y	Y
3.3 Were predictors excluded from the outcome definition?		PY	PY
3.4 Was the outcome defined and determined in a similar way for all participants?		PY	PY
3.5 Was the outcome determined without knowledge of predictor information?		PY	PY
3.6 Was the time interval between predictor assessment and outcome determination appropriate?		Y	Y
**Risk of bias introduced by the outcome or its determination**	**RISK:**<br>*(low/ high/ unclear)*	Low	Low
Rationale of bias rating:<br>*No major issues identified*			
**B. Applicability**			
At what time point was the outcome determined:<br>3 and 5 years<br><br>*If a composite outcome was used, describe the relative frequency/distribution of each contributing outcome:*<br>*N/A*			
**Concern that the outcome, its definition, timing or determination do not match the review question**	**CONCERN:**<br>*(low/ high/ unclear)*	Low	Low
Rationale of applicability rating:<br>*The outcome of the primary study matches the outcome of interest of the review*			

**DOMAIN 4: Analysis**
**Risk of Bias**
Describe numbers of participants, number of candidate predictors, outcome events and events per candidate predictor:<br>*"A total of 5,791 cases with nonmetastatic T3a RCC and eligible data, collected between 2010 and 2017, were selected from the SEER database. According to the ratio of 7:3, 4,055 cases were randomly assigned to the training group, and the remaining 1,736 cases were assigned to the verification group"*<br><br>*A total of 28 predictors were included (Table 2)*<br><br>*N= 4055 (E= 491) to develop the model and 1736 to validate it (E= 203)*<br>*EPV develop the model= 491/28=17,53*<br>*EPV internal validation= 203/28=7,25*
Describe how the model was developed (for example in regards to modelling technique (e.g. survival or logistic modelling), predictor selection, and risk group definition):<br><br>*"The included cases were randomly stratified into the training and verification group, in the ratio of 7:3. The descriptive statistics were conducted to describe the general characteristics of the cases in the two groups. Continuous variables were described as medians (interquartile range [IQR]) and compared using the Mann−Whitney U test. Categorical variables were described as frequencies and percentages and compared using x2 or Fisher's exact test. Univariate and multivariate Cox regression analyses were conducted to identify the significant predictors for CSS in the training group"*
Describe whether and how the model was validated, either internally (e.g. bootstrapping, cross validation, random split sample) or externally (e.g. temporal validation, geographical validation, different setting, different type of participants):<br><br>*"Bootstraps with 1,000 resamples were conducted to calculate the C- indices and plot calibration curves"*
Describe the performance measures of the model, e.g. (re)calibration, discrimination, (re)classification, net benefit, and whether they were adjusted for optimism:<br><br>*"The discrimination, which depicted the ability of the nomogram to predict the outcomes between different cases, was evaluated by Harrell's concordance index (C- index) and time-dependent receiver operating characteristic (ROC) curve. Calibration curves were used to visualize the differences between predicted and actual risks"*<br>*"Decision Curve Analysis was used to evaluate the utility of model for decision- making"*
Describe any participants who were excluded from the analysis:<br><br>*"Only patients with restaged T3aN0-1M0 RCC were included. Patients who were ≥-18-years-old at diagnosis, underwent partial or radical nephrectomy (PN or RN, RX Summ—Surg Prim Site [1988+]: 30 and 50) and had available information on gender, ethnicity, laterality, size, Fuhrman grade, sarcomatoid, and number of nodes removed were selected. Withal, only clear-cell, papillary, and chromophobe RCC were included. Patients with unknown cause of death or unreliable information on the aforementioned variables were excluded. Only cases with more than 1-month follow-up were included"*

Describe missing data on predictors and outcomes as well as methods used for missing data:<br><br>*"Only patients with restaged T3aN0-1M0 RCC were included. Patients who were ≥-18-years-old at diagnosis, underwent partial or radical nephrectomy (PN or RN, RX Summ—Surg Prim Site [1988+]: 30 and 50) and had available information on gender, ethnicity, laterality, size, Fuhrman grade, sarcomatoid, and number of nodes removed were selected. Withal, only clear-cell, papillary, and chromophobe RCC were included. Patients with unknown cause of death or unreliable information on the aforementioned variables were excluded. Only cases with more than 1-month follow-up were included"*			
		Dev	Val
4.1 Were there a reasonable number of participants with the outcome?		N	Y
4.2 Were continuous and categorical predictors handled appropriately?		N	N
4.3 Were all enrolled participants included in the analysis?		PY	PY
4.4 Were participants with missing data handled appropriately?		N	N
4.5 Was selection of predictors based on univariable analysis avoided?		N	
4.6 Were complexities in the data (e.g. censoring, competing risks, sampling of controls) accounted for appropriately?		N	N
4.7 Were relevant model performance measures evaluated appropriately?		Y	Y
4.8 Were model overfitting and optimism in model performance accounted for?		Y	
4.9 Do predictors and their assigned weights in the final model correspond to the results from multivariable analysis?		PY	
**Risk of bias introduced by the analysis**	**RISK:**<br>*(low/ high/ unclear)*	High	High
Rationale of bias rating:<br>*They hadn ´t enough patients with the event. They did categorizations. They excluded patients with missing data. They selected the predictors based on univariable. They didn't use competing risks.*			

Step 4: Overall assessment

Use the following tables to reach overall judgements about risk of bias and concerns regarding applicability of the prediction model evaluation (development and/or validation) across all assessed domains.
Complete for each evaluation of a distinct model.

Reaching an overall judgement about risk of bias of the prediction model evaluation	
**Low risk of bias**	If all domains were rated low risk of bias.<br>If a prediction model was developed without any external validation, and it was rated as low risk of bias for all domains, consider downgrading to **high risk of bias**. Such a model can only be considered as low risk of bias, if the development was based on a very large data set and included some form of internal validation.
**High risk of bias**	If at least one domain is judged to be at **high risk of bias**.
**Unclear risk of bias**	If an unclear risk of bias was noted in at least one domain and it was low risk for all other domains.

Reaching an overall judgement about applicability of the prediction model evaluation	
**Low concerns regarding applicability**	If low concerns regarding applicability for all domains, the prediction model evaluation is judged to have **low concerns regarding applicability**.
**High concerns regarding applicability**	If high concerns regarding applicability for at least one domain, the prediction model evaluation is judged to have **high concerns regarding applicability**.
**Unclear concerns regarding applicability**	If unclear concerns (but no “high concern”) regarding applicability for at least one domain, the prediction model evaluation is judged to have **unclear concerns regarding applicability** overall.

Overall judgement about risk of bias and applicability of the prediction model evaluation		
**Overall judgement of risk of bias**	**RISK:**<br>*(low/ high/ unclear)*	High
Summary of sources of potential bias:<br>Analysis domain shows several major issues		
**Overall judgement of applicability**	**CONCERN:**<br>*(low/ high/ unclear)*	Low
Summary of applicability concerns:<br><br>No major issues		
